# Synthesis of α,γ-Chiral Trifluoromethylated Amines through the Stereospecific Isomerization of α-Chiral Allylic Amines

**DOI:** 10.1021/acs.orglett.2c01436

**Published:** 2022-05-19

**Authors:** Víctor García-Vázquez, Pablo Martínez-Pardo, Alexandru Postole, A. Ken Inge, Belén Martín-Matute

**Affiliations:** † Department of Organic Chemistry, Arrhenius Laboratory, Stockholm University, SE-106 91 Stockholm, Sweden; ‡ Department of Materials and Environmental Chemistry, Arrhenius Laboratory, Stockholm University, SE-106 91 Stockholm, Sweden

## Abstract

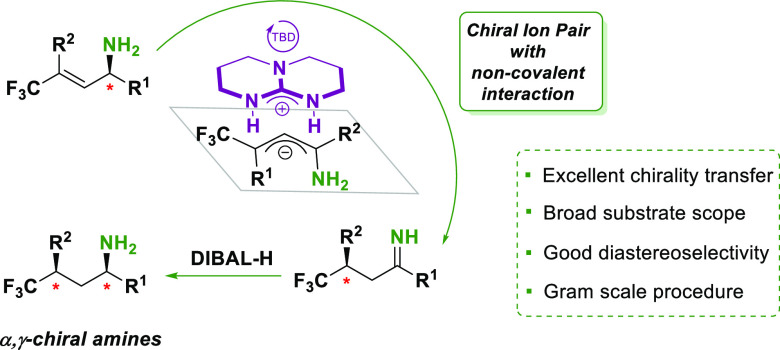

Chiral γ-branched aliphatic amines are present in a large number of pharmaceuticals and natural products. However, enantioselective methods to access these compounds are scarce and mainly rely on the use of designed chiral transition-metal complexes. Herein, we combined an organocatalytic method for the stereospecific isomerization of chiral allylic amines with a diastereoselective reduction of the chiral imine/enamine intermediates, leading to γ-trifluoromethylated aliphatic amines with two noncontiguous stereogenic centers, in excellent yields and high diastereo- and enantioselectivities. This approach has been used with primary amine substrates. This approach also provides a new synthetic pathway to chiral trifluoromethylated scaffolds, of importance in medicinal chemistry. Additionally, a gram-scale reaction demonstrates the applicability of this synthetic procedure.

Chiral primary amines are very valuable and versatile building blocks for the synthesis of amine-containing pharmaceuticals and natural products.^1^ Furthermore, chiral aliphatic amines bearing at least one stereogenic center are common substructures in natural products and pharmaceuticals, where the amine functional group is crucial for their biological activity (Figure 1).^2,3^ There are numerous synthetic methods that allow the stereochemistry of α- and β-chiral amines to be controlled.^3,4^ However, the synthesis of chiral amines with the stereogenic center at a remote position remains challenging.^5^

**Figure 1:**
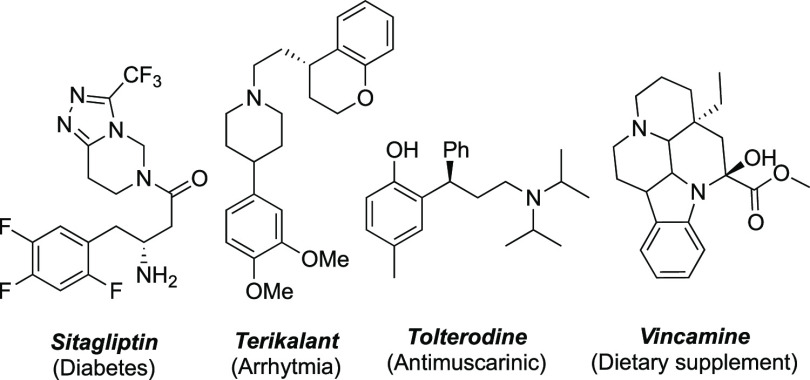
Relevant examples of chiral aliphatic amines.

An indirect approach would be to combine the diastereoselective 1,4-addition of organocopper reagents to α,β-unsaturated chiral sulfinyl imines, followed by reduction/deprotection steps (Figure 2a). The first step has been explored previously by Ellman and co-workers, enabling the addition of butyl and methyl organocopper reagents (Figure 2a).^6^ The stereoselective transformation of the resulting sulphinyl imines has not been reported to the best of our knowledge.

**Figure 2:**
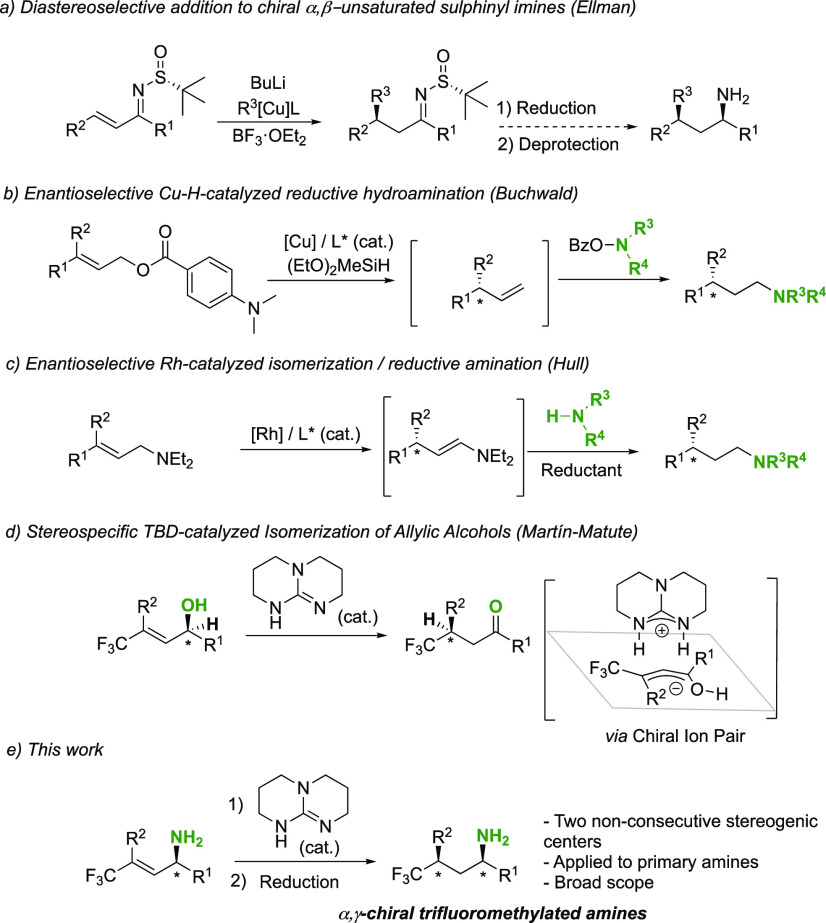
Enantioselective and enantiospecific strategies for the synthesis of chiral γ-branched aliphatic amines.

Buchwald and co-workers developed a copper(I)-catalyzed hydrocupration/β-alkoxide elimination reaction of allylic esters, followed by an anti-Markovnikov hydroamination of the olefin intermediate (Figure 2b).^7^ This method provides γ-chiral aliphatic amines with excellent enantioselectivities. However, it requires the use of specific electrophilic aminating reagents, and it is limited to tertiary amines. The Hull group contributed to this area with a Rh-catalyzed enantioselective isomerization/reductive amination of allylic diethyl amines (Figure 2c).^8^ In this case, the product of the redox-neutral isomerization process, a chiral enamine, reacts with amines in the presence of a reducing agent (NaBH_4_ or HCO_2_H) to give γ-chiral primary and secondary amine products with high enantioselectivities. Other examples reported in literature include the synthesis of protected γ-chiral substituted aliphatic amines through enantioselective Pd-catalyzed fluoroarylation,^9^ or direct hydrogenation of allyl amines.^10^ However, none of these methods tolerate further substitution at Cα or encompass primary amines.

The transition-metal-catalyzed isomerization of allylic alcohols or amines has been widely used to access γ-chiral carbonyl compounds and enamines (i.e., as in step 1 in Scheme 1b), respectively.^8,11−14^ Chirality is introduced by using metal complexes with specially designed chiral ligands. The synthesis of these ligands requires additional work, and the substrate scope of the reaction is dependent on the ligand used.^15^ An alternative method for the synthesis of carbonyl compounds with remote stereogenic centers is the stereospecific isomerization of α-chiral allylic alcohols, which are easily accessible α-chiral starting materials.^1,3,16,17^ These isomerization reactions can be catalyzed or mediated by achiral metal complexes,^18,19^ or by achiral bases,^20−24^ and take place through [1,3]-hydrogen shifts. The reaction takes place by a stepwise mechanism, so stereospecific examples are scarce.^18,19,22−24^ Our group has contributed to this field with the stereospecific isomerization of β-trifluoromethylated allylic alcohols, ethers, and halides mediated by catalytic amounts of the base 1,5,7-triazabicyclo[4.4.0]dec-5-ene (TBD; Figure 2c).^22−24^ Chirality is transferred from Cα to Cγ in a stepwise manner, through the formation of a tight-ion-pair intermediate with induced noncovalent chirality (Figure 2d). When it comes to the base-mediated isomerization of allylic amines yielding enamines, only one protocol has been reported to the best of our knowledge, which in this example is nonstereospecific.^25^ Other related recent contributions include an Ir-catalyzed asymmetric allylic substitution–isomerization strategy by the group of He.^26,27^

**Scheme 1:**
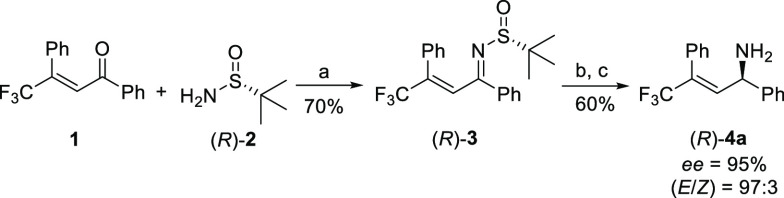
Synthesis of Chiral Trifluoromethylated Allylic Amines Reaction conditions: **1** (4 g, 14.5 mmol), (*R*)-**2** (2.6 g, 21.8 mmol, 1.5 equiv), Ti(OEt)_4_ (6.6 g, 29 mmol, 2 equiv), MW, 100 °C, 2 h (70%). (*R*)-**3** (3.8 g, 10.2 mmol), DIBAL-H (1 M in THF; 11 mL, 1.1 equiv), THF (10 mL, 1 M), 0 °C, 2 h. HCl (3M in H_2_O; 10 mL), THF (10 mL), rt, 18 h (60% over two steps).

In this work, we report a new method for the synthesis of chiral γ-aliphatic amines with two stereogenic centers in noncontiguous positions. The method relies on a stereospecific TBD-mediated isomerization of α-chiral allylic amines. As the reaction tolerates a further substituent at Cα, a subsequent reduction leads to functionalized aliphatic amines with two stereogenic centers, at Cα and at Cγ, starting from readily available chiral allylic amines (Figure 2d). Importantly, the reaction works on primary allylic amines, so it represents a direct method for the synthesis of α,γ-chiral primary amines. Further, this method also gives access to chiral trifluoromethylated building blocks,^28,29^ with high potential in medicinal chemistry.^30^

We started our investigations by designing an enantioselective synthesis of trifluoromethylated allylic amines (Scheme 1). Inspired by Guijarro’s work on the synthesis of *N-(tert*-butylsulfinyl)imines,^31^ we subjected enone **1** to a Ti (IV) mediated, microwave-assisted reaction with (*R*)-**2** to obtain the desired chiral sulfinimine (*R*)-**3** in 70% yield. A diastereoselective reduction with DIBAL-H and a final acidic deprotection gave trifluoromethylated chiral allylic amine (*R*)-**4a** with 95% *ee* and in 42% yield over three steps.

Having developed this enantioselective protocol for the synthesis of chiral trifluoromethylated allylic amines, we went on to examine the base-catalyzed stereospecific isomerization of γ-trifluoromethylated allylic amine **4a**. When allylic amine **4a** is treated with base, it undergoes isomerization to give a mixture of the primary enamine and the imine, as observed by NMR spectroscopy, which cannot be isolated. We therefore hydrolyzed this mixture by treatment with HCl (2 M) to give the corresponding chiral ketone, from which we could determine the efficiency of the chirality transfer.^22^ We found that when **4a** was treated with catalytic amounts of TBD, it underwent the isomerization reaction to yield ketone **5a** in quantitative yield and with high levels of chirality transfer (c.t.; Table 1, entry 1). The reaction also took place in the presence of a catalytic amount of 1,8-diazabicyclo[5.4.0]undec-7-ene (DBU) or MTBD (*N*-methyl TBD), although the yields were significantly lower (Table 1, entries 2 and 3 vs entry 1).^32^ Catalytic amounts of the more basic phosphazene P_4_-*t*-Bu did not yield **5a**. This result indicates that the reaction relies not only on the basicity of the catalyst but also on the ability of its conjugate acid to protonate the allylic anion intermediate (Table 1, entry 4). Decreasing the temperature to 60 °C did not show any significant effect on either the conversion or the chirality transfer (Table 1, entry 5), and the reaction did not work at room temperature (Table 1, entry 6). As expected, solvents that can be deprotonated did not give the product (Table 1, entry 7). In polar aprotic solvents such as 1,4-dioxane and ethyl acetate, the yields were similar to those obtained in toluene, but the chirality transfer was less efficient (Table 1, entries 8 and 9 vs entry 5). Finally, the effect of the catalyst loading was studied, and 5 mol % of TBD was found to be sufficient for the reaction to take place in high yield and, importantly, with an increased chirality transfer of 95% (Table 1, entry 10). Any further decrease in the catalyst loading was found to be detrimental to the reaction (Table 1, entry 11).

**Table 1:**
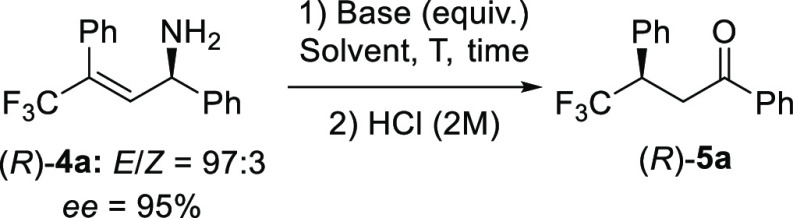
Optimization of the Stereospecific Isomerization Reaction of Chiral Allylic Amines^a^

Entry	Base (equiv)	Solvent	Temp [°C]	Yield [%]^b^	*c.t.* [%]^c^
**1**	TBD (0.1)	Toluene	120	>99	84
**2**	DBU (0.1)	Toluene	120	52	n.d.
**3**	MTBD (0.1)	Toluene	120	17	n.d.
**4**	P_4_-^*t*^Bu (0.1)^d^	Toluene	120	7	n.d.
**5**	TBD (0.1)	Toluene	60	>99	88
**6**	TBD (0.1)	Toluene	25	0	n.d.
**7**	TBD (0.1)	CHCl_3_	60	11	n.d.
**8**	TBD (0.1)	Dioxane	60	>99	86
**9**	TBD (0.1)	EtOAc	60	>99	84
**10**	TBD (0.05)	Toluene	60	>99	95
**11**	TBD (0.025)	Toluene	60	45	n.d.

a Reactions on **4a** (0.1 mmol) 0.02 M.

b Yield determined by ^19^F NMR spectroscopy.

c *c.t*. = (*ee*_product_/*ee*_SM_) × 100%.

d 0.8 M solution in hexane. *n.d*. = not determined.

Having optimized the reaction conditions for the stereospecific isomerization (Table 1, entry 11), we went on to study the reduction of the enamine intermediate to form α,γ-chiral trifluoromethylated aliphatic amine **6a** (Table 2). Compound *rac*-**4a** was subjected to the isomerization conditions as before, followed by treatment with a reducing agent in a two-step one-pot protocol. When the isomerization was completed, the temperature was adjusted before addition of the reductant. No further manipulations were done. When NaBH_4_ was used at room temperature, good yields were obtained, but the diastereoselectivity was poor (Table 2, entry 1). DIBAL-H showed moderate diastereoselectivity in favor of *syn*-**6a** at room temperature (Table 2, entry 2), which was improved at lower temperatures, and the major diastereomer (*syn*-**6a**) was obtained in a good 75% isolated yield at −90 °C (Table 2, entries 3–5). Other reducing agents such as l-selectride and lithium triethylborohydride gave lower conversions and complex reaction mixtures due to formation of defluorinated byproducts (see Table S1).

**Table 2:**
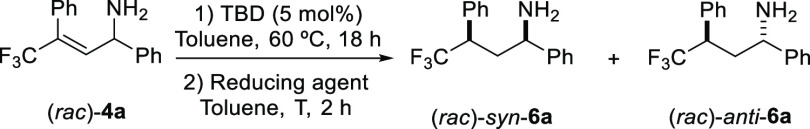
Optimization of the One-Pot Synthesis of (*rac*)-**6a**^a^

Entry	Reducing agent	Temp [°C]	**6a** [%]^b^	*d.r.* (*syn:anti*)^b^
1^c^	NaBH_4_	25	>99	50:50
2	DIBAL-H	25	65^d^	58:42
3	DIBAL-H	0	>99	65:35
4	DIBAL-H	–78	>99	70:30
5	DIBAL-H	–90	>99 (75)	75:25

a Reactions on **4a** (0.1 mmol) and reducing agent (2 equiv), 0.02 M.

b Yield and *d.r*. determined by ^19^F NMR spectroscopy. Isolated yield in parentheses.

c Toluene/MeOH (1:1).

d Different byproducts observed.

Having optimized the reaction conditions (Table 1, entry 11 for the stereospecific isomerization, and Table 2, entry 5 for the reduction), the scope and limitations were investigated (Scheme 2).

**Scheme 2:**
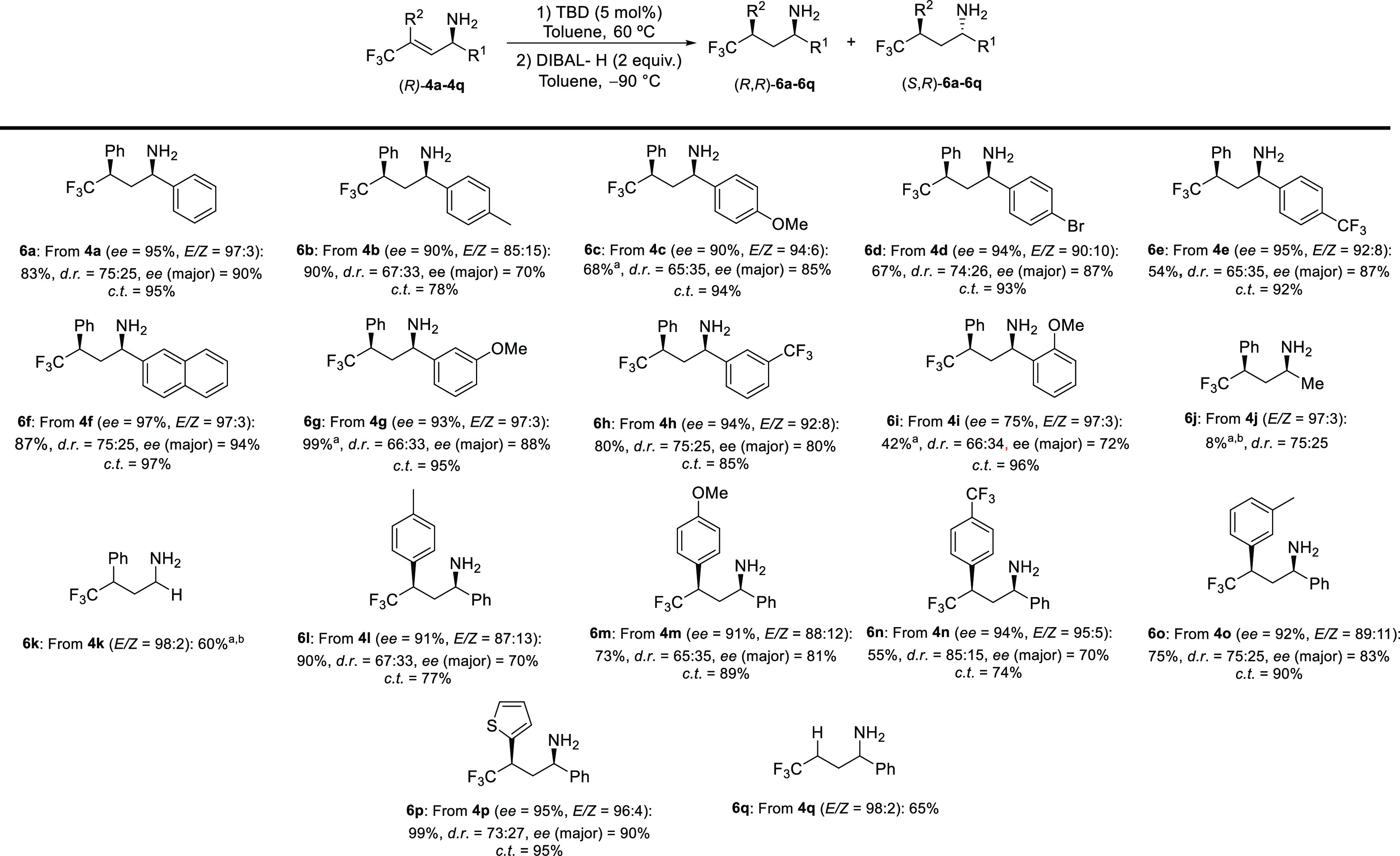
Scope of the TBD-Catalyzed Stereospecific Isomerization/Reduction Reaction of Allylic Amines Reaction conditions: (*R*)-**4a**–**4q** (0.25 mmol, 1 equiv), TBD (0.013 mmol, 0.05 equiv), toluene (12.5 mL, 0.02 M), 60 °C, 18 h. DIBAL-H (0.5 mL, 1 M in THF, 2 equiv), −90 °C, 2 h. Yield and *d.r.* determined by ^19^F NMR spectroscopy; isolated yields of each diastereomer are given in the Supporting Information. Chirality transfer (*c.t.*): (*ee*_product_/*ee*_SM_) × 100%. 120 °C. Not isolated.

The effect of different aryl groups at R^1^ were evaluated first. Substrates bearing electron-donating, electron-withdrawing, or electron-neutral groups at the *para* position of the aryl group reacted smoothly to give the desired products in high yields and with good chirality transfer (**6a**–**6e**). It is important to note that the *ee* of the final products (*R*,*R*)-**6** results from a combination of three factors: (a) the efficiency of the stereospecific isomerization; (b) the *E*/*Z* ratio of the starting allylic amines (**4**), and (c) the *ee* of their α-carbons (Scheme S4). Despite this complexity, the products are obtained in very good enantiomeric ratios. The bulkier naphthyl derivative gave **6f** in good yield with a chirality transfer of 97%. *Meta* and *ortho* substitution at R^1^ were also well tolerated; the diastereoselectivity was not compromised, and yields and chirality transfer levels were maintained (**6g**–**6i**). Replacing the aryl group by an alkyl chain resulted in a dramatic decrease in the yield (**6j**). When **6k** was used as a substrate (R^1^ = H), **6k** was formed in 60% yield. Variation of R^2^ was also studied, and aromatic groups with electron-donating groups in the *para* position gave good yields and good levels of chirality transfer, with moderate diastereoselectivities (**6l**–**6m**). *para*-Trifluoromethyl-substituted allylic amine **4n** gave aliphatic amine **6n** with a decreased efficiency in terms of yield and chirality transfer, but the diastereoselectivity was enhanced. *meta*-Methyl-substituted **6o** was also obtained in high yield with excellent levels of chirality transfer. Heteroaryl derivative **6p** was obtained in excellent yield with high levels of chirality transfer. Replacing the aryl substituent by H had a significant effect on the yield of the reaction, and **6q** was obtained in 65% yield.

A gram-scale experiment was carried out on amine **4d** (Scheme 3). Aliphatic amine **6d** was obtained in 73% yield, with excellent levels of chirality transfer (91%) and good levels of diastereoselectivity (72:28). In addition, the absolute configurations of both allylic amine **4d** and major diastereomer **6d** were determined by X-ray single crystal diffraction analysis of their Boc-derivatives (Scheme 3 and Figures S1–S2), and the absolute configuration of the other chiral amines (**6a**–**6q**) was assigned by analogy.

**Scheme 3:**
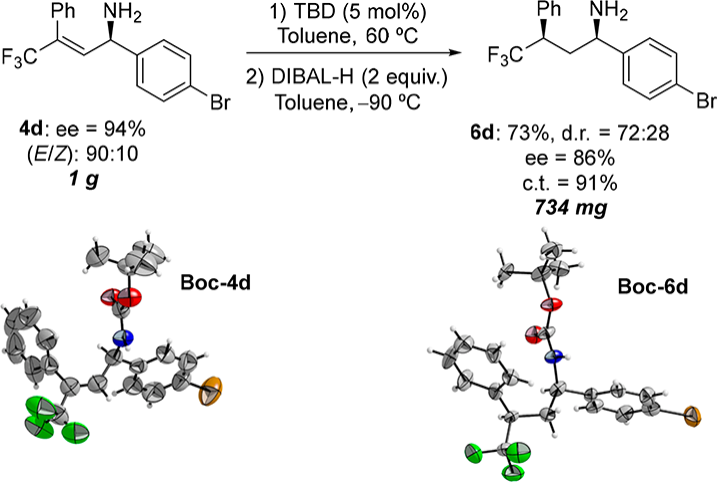
Gram-Scale Experiment and X-ray Single-Crystal Diffraction Structures

In conclusion, we have developed a method for the synthesis of γ-chiral aliphatic amines from easily accessible α-chiral allylic amines using a base catalyst. A subsequent diastereoselective reduction of the chiral imine/enamine intermediate leads to α,γ-chiral γ-trifluoromethylated amines in excellent yields and with high diastereo- and enantioselectivities. We have shown that the reaction has a broad scope, and the reaction has been run on a gram scale. Thus, this represents a straightforward approach to α,γ-chiral trifluoromethylated amines from accessible allylic amines. We have shown that the reaction has a broad scope, and the reaction has been run on a gram scale. Thus, this represents a straightforward approach to α,γ-chiral trifluoromethylated amines from accessible allylic amines.

## Supporting Information Available

Experimental procedures, characterization of compounds, and spectra (PDF)

## Accession Codes

Original NMR FIDs and HPLC data are available, free of charge open access, at Zenodo at: https://zenodo.org/record/6563011. CCDC 2129456–2129457 contain the supplementary crystallographic data for this paper. These data can be obtained free of charge via www.ccdc.cam.ac.uk/data_request/cif, or by emailing data_request@ccdc.cam.ac.uk, or by contacting The Cambridge Crystallographic Data Centre, 12 Union Road, Cambridge CB2 1EZ, UK; fax: +44 1223 336033.

## Notes

This article has been previously preprinted.^33^
