# Supplementary material for: Synthesis of α,γ-Chiral Trifluoromethylated Amines through the Stereospecific Isomerization of α-Chiral Allylic Amines
Source: Org Lett. 2022 May 19;24(21):3867–71. doi: 10.1021/acs.orglett.2c01436 (PMC9490871; doi:10.1021/acs.orglett.2c01436)
Supplement: Supplementary file 1 — ol2c01436_si_001.pdf [file ol2c01436_si_001.pdf]

Synthesis of α,γ-Chiral Trifluoromethylated Amines through the Stereospecific Isomerization of α-Chiral Allylic Amines

Víctor García-Vázquez,[‡,a] Pablo Martínez-Pardo,[‡,a] Alexandru Postole,[a] A. Ken Inge[b] and Belén Martín-Matute[*,a]

[a] Department of Organic Chemistry, Arrhenius Laboratory, Stockholm University, SE-106 91 Stockholm, Sweden.

[b] Department of Materials and Environmental Chemistry, Arrhenius Laboratory, Stockholm University, SE-106 91 Stockholm, Sweden.

Table of Contents

**General Methods**	S3
**Optimization of reduction conditions**	S4
**Control experiments**	S5
**General procedures and substrates that did not work**	S5
**Isomerization of allylic compounds and their possible products**	S6
**General procedures**	S7
**Characterization data of allylic amines 4 and 4'**	S7
**Characterization data of amines 6 and 6'**	S22
**NMR spectra and HPLC of compounds 4 and 4'**	S43
**NMR spectra and HPLC of compounds 6 and 6'**	S89
**Crystal structure determination of compounds 4d' and (1*R*,3*R*)-6d'**	S150

General methods

All reagents were utilized without any further purification as obtained from commercial sources. Flash chromatography was performed with 60 Å (35-70 μm) silica gel (GC 60A 35-70 Micron, DAVISIL). Analytical TLC was performed on aluminum plates pre-coated (0-25 mm) with silica gel (Merck, Silica Gel 60 F254). Compounds were detected by exposure to UV light or by revealing the plates in a solution of 5% $KMnO_4$ in water. Melting points were recorded in metal block and are uncorrected. ^1H, ^{13}C and ^{19}F NMR spectra were recorded at 400 MHz, 100 MHz and 376 MHz respectively on a Bruker Advance spectrometer. Chemical shifts (d) are shown in ppm, using as a reference the residual peaks of $CDCl_3$ (d_H 7.26 and d_C 77.00). Coupling constants (*J*) are given in Hz. NMR yields were calculated using 1 equiv. of 1,2,4,5-tetrachloronitrobenzene as internal standard. High resolution mass spectra (HRMS) were recorded on Bruker microTOF mass spectrometer using APCI ionization. Herein, are reported the MS when they were possible to be detected. Enantiomeric excesses were determined using HPLC analysis on an Agilent 1200-series instrument with an autosampler and UV detection and using Chiralcel OD-H, Chiralpak AD-H and IF and Phenomenex Lux Cellulose 5 columns. Optical rotations were recorded on a RUDOLPH AUTOPOL IV with an automatic polarimeter. Microwave reactions were performed in an Initiator Classic microwave reactor from Biotage.

Allylic amines (compounds **4a**-**4q**) decompose under HRMS conditions, we have identified the fragmentations of three of those compounds (**4a**, **4f**, **4h**).
Although products **6a**-**6q** could be identified by HRMS (**6a**, **6f**, **6h**), these compounds showed a higher stability when were Boc-protected. Therefore, we provide HRMS of all Boc-protected amines (both allylic amines **4'** and aliphatic amines **6'**) and NMR (^1H NMR, ^{13}C NMR and ^{19}F NMR) data for non-protected amines

Instead, full characterization of boc-protected amines **4'** and **6'** are reported.

Optimization of reduction conditions

Table S1. Optimization of the one pot isomerization/diastereoselective synthesis of γ-trifluoromethylated aliphatic amines.[a]

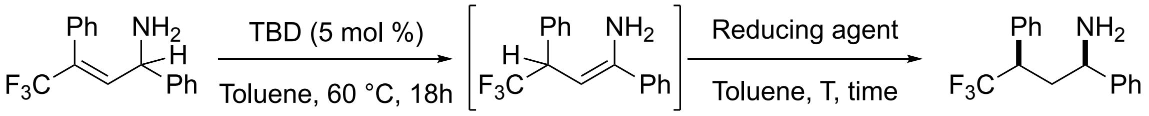

Entry	Reducing agent	Temp. (ºC)	Time (h)	Conversion (%)[b]	Yield (%)[b]	*d.r.*[b]
1[c]	$NaBH_4$	25	2	99	99	50:50
2	DIBAL-H	25	2	99		58:42
3	$BH_3 \cdot SMe_2$	25	18	0	0	-
4	$BH_3 \cdot THF$	25	18	0	0	-
5	$Et_3SiH/B(C_6F_5)_3$	25	18	0	0	-
6	DIBAL-H	0	2	99	99	65:35
7	DIBAL-H	–78 ºC	2	99	99	70:30
8	L-Selectride	–78 ºC	2	99	13	92:8
9[d]	L-Selectride	25	2	99	-	-
10	Super-hydride	–78 ºC	2	99	81	56:44
**11**	**DIBAL-H**	**–90 ºC**	**2**	**99**	**99% (75%)[e]**	**75:25**
12[f]	DIBAL-H	–90 ºC	2	99	10	-

a: Reactions were run using **4a** (0.1 mmol) and 2 equiv. of reducing agent, 0.02M. b: Conversion and *d.r.* determined by ^{19}F NMR spectroscopy. c: Toluene:MeOH (1:1) used as solvent. d: Decomposition of **4a** was observed. e: Isolated yield in parenthesis. f: 1 equiv. of DIBAL-H used, ketone **5a** observed as major product.

Control experiments performed on allylic amine 4a

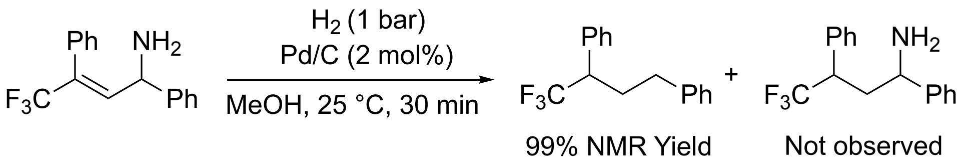

Scheme S1. Hydrogenation of **4a** with Pd/C.

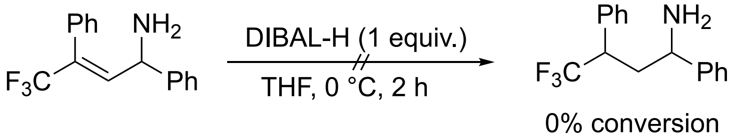

Scheme S2. Reduction of **4a** with DIBAL-H.

General procedure for the synthesis of enantioenriched allylic amines

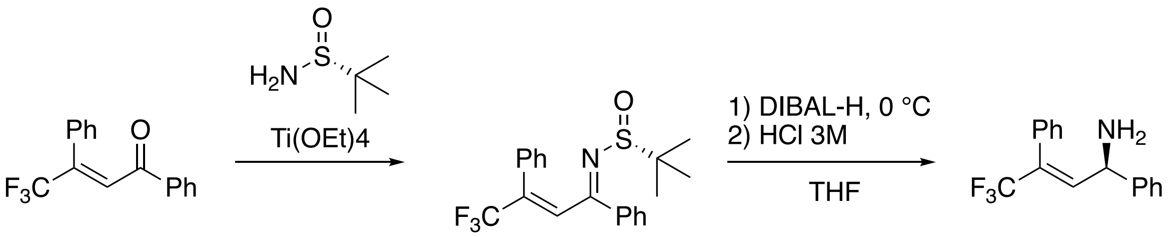

Scheme S3. Synthetic route for the synthesis of allylic amines **4**.

Allylic amines that could not be synthesized with this methodology

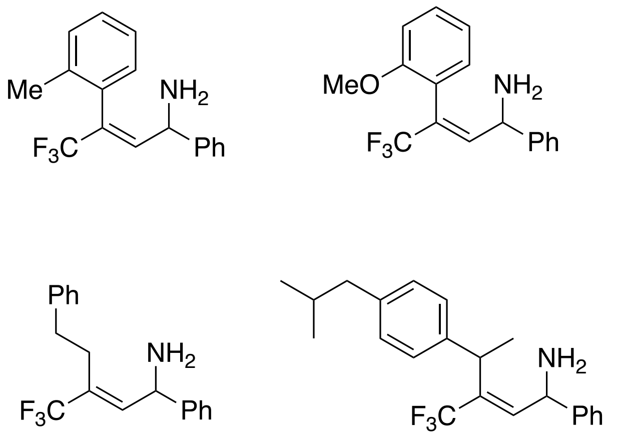

Figure S1. Allylic amines not accessible with the enantioselective synthetic pathway.

Allylic amines that do not work under the isomerization reaction conditions

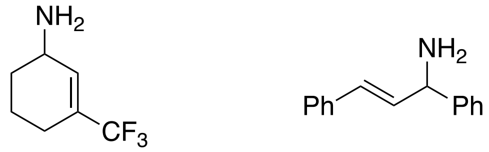

Figure S2. Allylic amines that don't undergo isomerization reaction.

Possible stereochemical outcome of the base-catalyzed isomerization of allylic amines

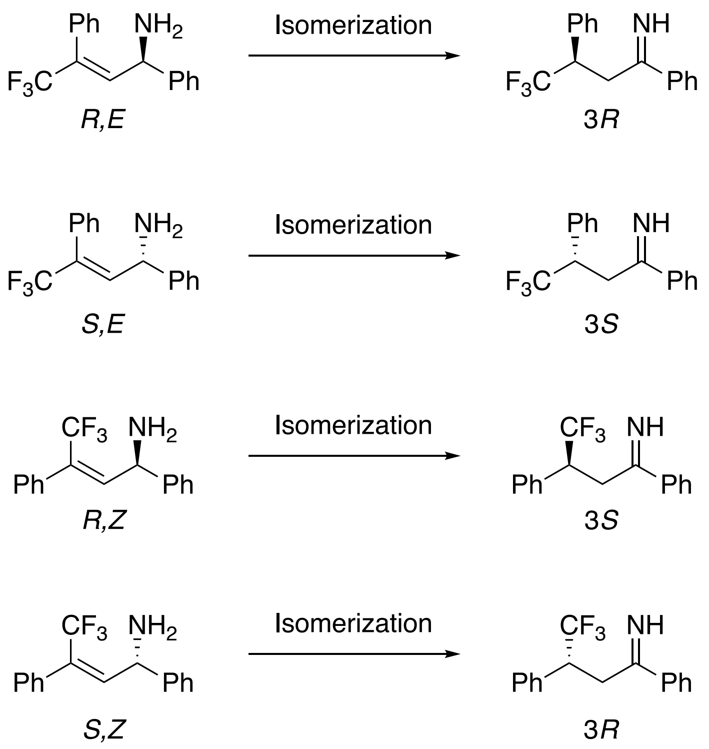

Scheme S4. Different stereosisomeric products accessible under the isomerization conditions.

Above is explained how the stereochemical information of the starting material affect the stereochemical outcome of the products. The stereospecificity of the isomerization reaction not only depends on the stereochemistry of the α-carbon of the allylic amine, but also on the stereochemistry of the alkene. Therefore, *R* amines lead to different stereoisomers than *S* amines, aswell as *Z* alkenes lead to different stereoisomers than *E* alkenes.

As a result of this stereodivergency, those substrates containing higher amounts of *Z* alkenes result in lower *ee* of the final chiral aliphatic amines.

General procedures

A Synthesis of allylic amines 4

The corresponding enone (5 mmol, 1 equiv.) was placed on a sealed MW vial with (*R*)-(+)-2-methyl-2-propanesulfinamide (7.5 mmol, 1.5 equiv.) and titanium(IV) ethoxide (10 mmol, 2 equiv.) and the reaction was stirred at 100 °C for 2 h under neat conditions in the MW reactor. After completion of the reaction, the resulting imine was purified with FCC (pentane:EtOAc 9:1) to afford the pure imine. The imine was dissolved in THF (1M) and the mixture was cooled to 0 °C, then DIBAL-H (5.5 mmol, 1.1 equiv.) was added dropwise. After the reaction was completed, a mixture THF:HCl 3M (50 mL, 1:1 *v/v,* 0.1M) was added and the reaction was stirred overnight at room temperature. The mixture was then basified adding NaOH (2M) slowly until reach pH = 14. The aqueous layer was extracted with EtOAc (3x100 mL), the organic layers were washed with brine and dried over $MgSO_4$. The solvent was evaporated under reduced pressure and the amine was purified by FCC (pentane:EtOAc 8:2 to 6:4) to afford the pure allylic amines.

(*rac*)-**4** were obtained using the same protocol with (*rac*)-2-methyl-2-propanesulfinamide.

B Amine protection

To a solution of the corresponding amine (0.3 mmol, 1 equiv.) in $CHCl_3$ (3 mL, 0.1 M) at 0 °C a solution of di-*tert*-butyl dicarbonate (0.36 mmol, 1.2 equiv.) in $CHCl_3$ (3mL, 0.1 M) was added and the reaction was stirred overnight. Then, the protected amine was purified by FCC (pentane:EtOAc 97:3 to 9:1) to obtain the desired compounds.

C Isomerization and reduction of the allylic amines 6

The allylic amine (0.25 mmol, 1 equiv.) and TBD (0.012 or 0.025 mmol, 5 or 10 mol %) were charged on a pressure vial and purged with Ar. Dry Toluene (12.5 mL, 0.02 M) was added and the reaction mixture was stirred for 18 h at 60 or 120 °C on an oil bath. Then, the reaction mixture was allowed to reach room temperature and cooled to -90 °C. DIBAL-H (0.5 mmol, 2 equiv.) was added and stirred at that temperature for an additional 4 h. The reaction was allowed to reach room temperature and a solution of Rochelle's salt was added and stirred for additional 30 min, the aqueous layer was extracted with EtOAc (3x15 mL), the organic layers were dried over $MgSO_4$, the solvent was removed under reduced pressure. The crude was then purified by FCC (pentane:EtOAc 7:3 to 0:1) to afford the pure amine.

Characterization of allylic amines 4 and 4'

(*R*,*E*)-4,4,4-Trifluoro-1,3-diphenylbut-2-en-1-amine (4a)

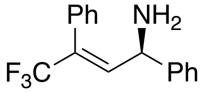

The title compound was obtained following GPA from (*R*,*E*)-4,4,4-trifluoro-1,3-diphenylbut-2-en-1-one (4.0 g, 14.5 mmol). The allylic amine was purified by FCC (pentane:EtOAc 7:3) as a yellow oil in 42% yield over 3 steps (1.69 g, 6.1 mmol).

^{1}H NMR (400 MHz. $CDCl_3$) δ 7.44 – 7.41 (m, 3H), 7.37 – 7.33 (m, 2H), 7.30 – 7.27 (m, 5H), 6.55 (dq, *J* = 9.5, 1.5 Hz, 1H), 4.48 (d, *J* = 9.5 Hz, 1H), 1.55 (bs, 2H).

^{13}C NMR (100 MHz, $CDCl_3$) δ 142.8, 139.0 (Cq, J_{CF} = 5.0 Hz), 131.7, 130.5 (Cq, J_{CF} = 30.0 Hz), 129.6, 128.82, 128.80, 128.5, 127.6, 126.4, 123.3 (Cq, J_{CF} = 273.5 Hz), 53.0.

^{19}F NMR (376 MHz, $CDCl_3$) δ – 66.24 (s, CF_3).

HRMS (ESI) *m/z*: Fragmentation observed: 261.0906 $[M - NH_2]^+$ corresponding to $C_{16}H_{12}F_3^+$, $C_{16}H_{12}F_3^+$ requires 261.0886.

***tert*-Butyl (*R*,*E*)-(4,4,4-trifluoro-1,3-diphenylbut-2-en-1-yl)carbamate (4a')**

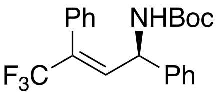

The title compound was obtained following GPB from (*R*,*E*)-4,4,4-trifluoro-1,3-diphenylbut-2-en-1-amine (100.0 mg, 0.36 mmol). The protected allylic amine was purified by FCC (pentane:EtOAc 95:5) as a white solid in 73% (100.0 mg, 0.26 mmol).

The enantiomeric excess (minor isomer: nd, major isomer 95%) was determined by HPLC (CHIRACEL® OD–H), hexane/iPrOH 98/2, 1 mL/min, (**1*R*,*E***) (major diastereomer): minor enantiomer t_r = 10.2 min, major enantiomer, t_r = 7.6 min.

$[\alpha]_D^{25}$: – 55.2 (c 1.00, $CHCl_3$, for the diastereomer mixture, d.r.: 97:3).

^{1}H NMR (400 MHz, $CDCl_3$) δ 7.41 – 7.39 (m, 3H), 7.33 – 7.27 (m, 5H), 7.17 – 7.15 (m, 2H), 6.54 (dd, *J* = 9.5, 2.0 Hz, 1H), 5.21 (bs, 1H), 4.96 (d, *J* = 7.5 Hz, 1H), 1.42 (s, 9H).

^{13}C NMR (100 MHz, $CDCl_3$) δ 154.5, 140.2, 135.3 (Cq, J_{CF} = 5.0 Hz), 132.3, 131.1, 129.5, 128.92, 128.89, 128.5, 127.9, 126.6, 123.2 (Cq, J_{CF} = 273.4 Hz), 80.0, 52.6, 28.3.

^{19}F NMR (376 MHz, $CDCl_3$) δ – 66.28 (s, CF_3).

HRMS (ESI) *m/z*: 400.1474 $[M+Na]^+$, $C_{21}H_{22}F_3NNaO_2^+$ requires 400.1495.

(*R*,*E*)-4,4,4-Trifluoro-3-phenyl-1-(*p*-tolyl)but-2-en-1-amine (4b)

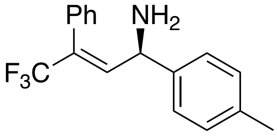

The title compound was obtained following GPA from (*R*,*E*)-4,4,4-trifluoro-3-phenyl-1-(*p*-tolyl)but-2-en-1-one (1.3 g, 4.5 mmol). The allylic amine was purified by FCC (pentane:EtOAc 7:3) as a yellow oil in 40% yield over 3 steps (0.52 g, 1.8 mmol).

^{1}H NMR (400 MHz. $CDCl_3$) δ 7.43 – 7.40 (m, 3H), 7.34 – 7.32 (m, 1H), 7.28-7.25 (m, 2H); 7.16 (s, 3H), 6.53 (dq, *J* = 10.0, 1.5 Hz, 1H), 4,44 (d, *J* = 10.0 Hz, 1H), 2.35 (s, 3H), 1.51 (bs, 2H).

^{13}C NMR (100 MHz, $CDCl_3$) δ 139.9, 139.1 (Cq, J_{CF} = 5.0 Hz), 137.4, 131.7, 129.6, 129.5, 128.8, 128.5, 128.2, 126.6, 123.3 (Cq, J_{CF} = 273.0 Hz), 52.8, 21.0.

^{19}F NMR (376 MHz, $CDCl_3$) δ – 66.21 (s, CF_3).

***tert*-Butyl (*R*,*E*)-(4,4,4-trifluoro-3-phenyl-1-(*p*-tolyl)but-2-en-1-yl)carbamate (4b')**

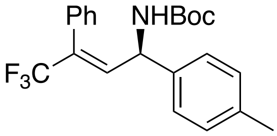

The title compound was obtained following GPB from (*R*,*E*)-4,4,4-trifluoro-3-phenyl-1-(*p*-tolyl)but-2-en-1-amine (58.3 mg, 0.20 mmol). The protected allylic amine was purified by FCC (pentane:EtOAc 95:5) as a white solid in 74% (57.9 mg, 0.15 mmol).

The enantiomeric excess (minor isomer: 96%, major isomer 90%) was determined by HPLC (CHIRACEL® OD–H), hexane/iPrOH 98/2, 1 mL/min, (**1*R*,*Z***) (minor diastereomer): minor enantiomer, t_r = 13.0 min, major enantiomer, t_r = 9.6 min, (**1*R*,*E***) (major diastereomer): minor enantiomer t_r = 6.0 min, major enantiomer, t_r = 5.5 min.

$[\alpha]_D^{25}$: – 49.9 (c 0.85, $CHCl_3$, for the diastereomer mixture, d.r.: 84:16).

^{1}H NMR (400 MHz, $CDCl_3$) δ 7.41 – 7.36 (m, 3H), 7.28 – 7.25 (m, 2H), 7.14 (d, *J* = 7.9 Hz, 2H), 7.05 (d, *J* = 7.9 Hz, 2H), 6.53 (dd, *J* = 9.5, 1.5 Hz, 1H), 5.16 (bs, 1H), 4.91 (d, *J* = 7.5 Hz, 1H), 2.34 (s, 3H), 1.42 (bs, 9H).

^{13}C NMR (100 MHz, $CDCl_3$) δ 154.5, 137.7, 137.2, 135.5 (Cq, J_{CF} = 5.0 Hz), 131.2, 129.63, 129.57, 128.9, 128.5, 128.3, 126.5, 123.2 (Cq, J_{CF} = 273.5 Hz), 79.9, 52.4, 28.3, 21.0.

^{19}F NMR (376 MHz, $CDCl_3$) δ – 66.22 (s, CF_3).

HRMS (ESI) *m/z*: 414.1619 $[M+Na]^+$, $C_{22}H_{24}F_3NNaO_2^+$ requires 414.1651.

(*R*,*E*)-4,4,4-Trifluoro-1-(4-methoxyphenyl)-3-phenylbut-2-en-1-amine (4c)

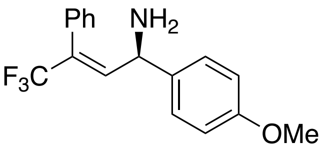

The title compound was obtained following GPA from (*R*,*E*)-4,4,4-trifluoro-1-(4-methoxyphenyl)-3-phenylbut-2-en-1-one (1.3 g, 4.2 mmol). The allylic amine was purified by FCC (pentane:EtOAc 7:3) as a yellow oil in 31% yield over 3 steps (0.4 g, 1.3 mmol).

^{1}H NMR (400 MHz. $CDCl_3$) δ 7.43 – 7.41 (m, 3H), 7.27 – 7.24 (m, 2H), 7.19 (d, *J* = 8.7 Hz, 2H), 6.88 (d, *J* = 8.7 Hz, 2H), 6.52 (dq, J = 9.6, 1.6 Hz, 1H), 4.42 (d, *J* = 9.6 Hz, 1H), 3.80 (s, 3H), 1.50 (bs, 2H).

^{13}C NMR (100 MHz, $CDCl_3$) δ 159.0, 139.3 (Cq, J_{CF} = 5.0 Hz), 135.0, 131.8, 130.1 (J_{CF} = 30.0 Hz) 129.7, 128.8, 128.5, 127.5, 123.3 (Cq, J_{CF} = 273.5 Hz), 114.2, 55.3, 52.5.

^{19}F NMR (376 MHz, $CDCl_3$) δ – 66.20 (s, CF_3).

***tert*-Butyl (*R*,*E*)-(4,4,4-trifluoro-1-(4-methoxyphenyl)-3-phenylbut-2-en-1-yl)carbamate (4c')**

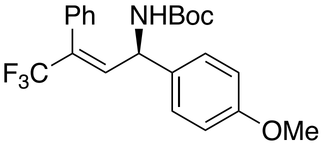

The title compound was obtained following GPB from (*R*,*E*)-4,4,4-trifluoro-1-(4-methoxyphenyl)-3-phenylbut-2-en-1-amine (61.5 mg, 0.20 mmol). The protected allylic amine was purified by FCC (pentane:EtOAc 95:5) as a white solid in 66% (53.8 mg, 0.13 mmol).

The enantiomeric excess (minor isomer: nd, major isomer 94%) was determined by HPLC (CHIRACEL® OD–H), hexane/iPrOH 98/2, 1 mL/min, (**1*R*,*E***) (major diastereomer): minor enantiomer t_r = 8.4 min, major enantiomer, t_r = 7.8 min.

$[\alpha]_D^{25}$: – 74.6 (c 1.01, $CHCl_3$, for the diastereomer mixture, d.r.: 94:6).

^{1}H NMR (400 MHz, $CDCl_3$) δ 7.39 – 7.35 (m, 3H), 7.27 – 7.25 (m, 2H), 7.06 (d, *J* = 9.0 Hz, 2H), 6.85 (d, *J* = 9.0 Hz, 2H), 6.52 (dq, *J* = 9.5, 1.5 Hz, 1H), 5.13 (bs, 1H), 4.88 (d, *J* = 7.5 Hz, 1H), 3.79 (s, 3H), 1.41 (s, 9H).

^{13}C NMR (100 MHz, $CDCl_3$) δ 159.2, 154.5, 135.6 (Cq, J_{CF} = 5.5 Hz), 132.3 131.2, 129.5, 128.9, 128.4, 127.8, 127.3, 123.2 (Cq, J_{CF} = 273.5 Hz), 114.3, 79.9, 55.2, 52.1, 28.3.

^{19}F NMR (376 MHz, $CDCl_3$) δ – 66.20 (s, CF_3).

HRMS (ESI) *m/z*: 430.1606 $[M+Na]^+$, $C_{22}H_{24}F_3NNaO_3^+$ requires 430.1600.

(*R*,*E*)-1-(4-Bromophenyl)-4,4,4-trifluoro-3-phenylbut-2-en-1-amine (4d)

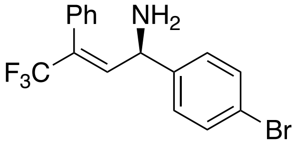

The title compound was obtained following GPA from (*R*,*E*)-1-(4-bromophenyl)-4,4,4-trifluoro-3-phenylbut-2-en-1-one (5.0 g, 14.1 mmol). The allylic amine was purified by FCC (pentane:EtOAc 7:3) as a yellow oil in 55% yield over 3 steps (2.7 g, 7.7 mmol).

^{1}H NMR (400 MHz. $CDCl_3$) δ 7.47 – 7.42 (m, 5H), 7.26 – 7.23 (m, 2H), 7.14 (d, *J* = 8.4 Hz, 2H), 6.47 (dd, *J* = 9.7, 1.7 Hz, 1H), 4.44 (d, *J* = 9.7 Hz, 1H), 1.51 (bs, 2H).

^{13}C NMR (100 MHz, $CDCl_3$) δ 141.8, 138.5 (Cq, J_{CF} = 5.5 Hz), 131.9, 131.5, 129.5, 128.9, 128.6, 128.2, 127.2, 123.1 (Cq, J_{CF} = 273.5 Hz), 119.0, 52.6.

^{19}F NMR (376 MHz, $CDCl_3$) δ – 66.37 (s, CF_3).

***tert*-Butyl (*R*,*E*)-(1-(4-bromophenyl)-4,4,4-trifluoro-3-phenylbut-2-en-1-yl)carbamate (4d')**

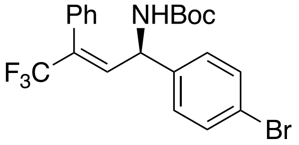

The title compound was obtained following GPB from (*R*,*E*)-1-(4-bromophenyl)-4,4,4-trifluoro-3-phenylbut-2-en-1-amine (71.2 mg, 0.20 mmol). The protected allylic amine was purified by FCC (pentane:EtOAc 95:5) as a white solid in 90% (82.3 mg, 0.18 mmol) [m.p.: 110 – 112 °C].

The enantiomeric excess (minor isomer: 92, major isomer 97%) was determined by HPLC (CHIRACEL® OD–H), hexane/iPrOH 98/2, 1 mL/min, (**1*R*,*Z***) (minor diastereomer): minor enantiomer,

t_r = 16.8 min, major enantiomer, t_r = 12.7 min, (**1*R*,*E***) (major diastereomer): minor enantiomer t_r = 7.6 min, major enantiomer, t_r = 6.8 min.

$[\alpha]_D^{25}$: – 96.8 (c 1.00, $CHCl_3$, for the diastereomer mixture, d.r.: 90:10).

^{1}H NMR (400 MHz, $CDCl_3$) δ 7.44 (d, J = 8.0 Hz, 2H), 7.41 – 7.40 (m, 3H), 7.26 – 7.24 (m, 2H), 7.02 (d, J = 8.0 Hz, 2H), 6.47 (d, J = 9.0 Hz, 1H), 5.15 (bs, 1H), 4.97 (bs, 1H), 1.40 (s, 9H).

^{13}C NMR (100 MHz, $CDCl_3$) δ 154.4, 139.3, 134.5 (Cq, J_{CF} = 5.5 Hz), 132.1, 132.0, 130.9, 129.4, 129.1, 128.6, 128.2, 123.0 (Cq, J_{CF} = 273.6 Hz), 121.8, 80.2, 52.2, 28.2.

^{19}F NMR (376 MHz, $CDCl_3$) δ – 66.38 (s, CF_3).

HRMS (ESI) *m/z*: 480.0637 $[M+Na]^+$, $C_{21}H_{21}BrF_3NNaO_2^+$ requires 480.0580.

(*R*,*E*)-4,4,4-Trifluoro-3-phenyl-1-(4-(trifluoromethyl)phenyl)but-2-en-1-amine (4e)

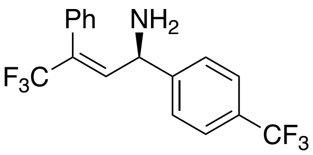

The title compound was obtained following GPA from (*E*)-4,4,4-trifluoro-3-phenyl-1-(4-(trifluoromethyl)phenyl)but-2-en-1-one (3.7 g, 10.8 mmol). The allylic amine was purified by FCC (pentane:EtOAc 7:3) as a yellow oil in 54% yield over 3 steps (2.0 g, 5.8 mmol).

^{1}H NMR (400 MHz. $CDCl_3$) δ 7.61 (d, J = 8.0 Hz, 2H), 7.46 – 7.44 (m, 3H), 7.40 (d, J = 8.0 Hz, 2H), 7.28 – 7.27 (m, 2H), 6.50 (dd, J = 9.5, 1.5 Hz, 1H), 4.55 (d, J = 9.5 Hz, 1H), 1.56 (bs, 2H).

^{13}C NMR (100 MHz, $CDCl_3$) δ 146.7, 138.2, (Cq, J_{CF} = 5.0 Hz), 131.5 (J_{CF} = 30.0 Hz), 131.4, 129.9 (J_{CF} = 32.4 Hz), 129.5, 129.0, 128.7, 126.9, 125.7 (Cq, J_{CF} = 4.0 Hz), 124.0 (Cq, J_{CF} = 272.0 Hz), 123.1 (Cq, J_{CF} = 273.5 Hz), 52.8.

^{19}F NMR (376 MHz, $CDCl_3$) δ – 66.55 (s, CF_3), – 66.46 (s, CF_3).

***tert*-Butyl (*R*,*E*)-(4,4,4-trifluoro-3-phenyl-1-(4-(trifluoromethyl)phenyl)but-2-en-1-yl)carbamate (4e')**

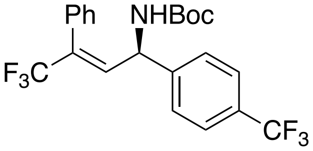

The title compound was obtained following GPB from (*R*,*E*)-4,4,4-trifluoro-3-phenyl-1-(4-(trifluoromethyl)phenyl)but-2-en-1-amine (86.3 mg, 0.25 mmol). The protected allylic amine was purified by FCC (pentane:EtOAc 95:5) as a white solid in 88% (98.1 mg, 0.22 mmol).

The enantiomeric excess (minor isomer: nd, major isomer 95%) was determined by HPLC (CHIRACEL® OD–H), hexane/iPrOH 98/2, 1 mL/min, (**1*R*,*E***) (major diastereomer): minor enantiomer t_r = 7.7 min, major enantiomer, t_r = 13.9 min.

$[\alpha]_D^{25}$: – 63.1 (c 0.99, $CHCl_3$, for the diastereomer mixture, d.r.: 91:9).

^{1}H NMR (400 MHz, $CDCl_3$) δ 7.60 (d, *J* = 8.0 Hz, 2H), 7.44 – 7.72 (m, 3H), 7.30 – 7.26 (m, 4H), 6.51 (dd, *J* = 9.5, 1.5 Hz, 1H), 5.28 (bs, 1H), 5.04 (bs, 1H), 1.42 (s, 9H).

^{13}C NMR (100 MHz, $CDCl_3$) δ 154.4, 144.3, 134.1 (Cq, J_{CF} = 5.5 Hz), 130.8, 130.1 (Cq, J_{CF} = 32.5 Hz), 129.4, 129.2, 128.7, 128.3 (Cq, J_{CF} = 32.2 Hz), 126.9, 125.9 (Cq, J_{CF} = 4.0 Hz), 123.9 (Cq, J_{CF} = 272.0 Hz), 123.0 (Cq, J_{CF} = 273.5 Hz), 80.4, 52.3, 28.2.

^{19}F NMR (376 MHz, $CDCl_3$) δ – 62.64 (s, CF_3), – 66.50 (s, CF_3).

HRMS (ESI) *m/z*: 468.1336 $[M+Na]^+$, $C_{22}H_{21}F_6NNaO_2^+$ requires 468.1369.

(*R*,*E*)-4,4,4-Trifluoro-1-(naphthalen-2-yl)-3-phenylbut-2-en-1-amine (4f)

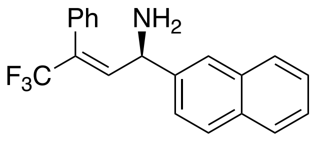

The title compound was obtained following GPA from (*E*)-4,4,4-trifluoro-1-(naphthalen-2-yl)-3-phenylbut-2-en-1-one (2.3 g, 7.1 mmol). The allylic amine was purified by FCC (pentane:EtOAc 7:3) as a yellow oil in 35% yield over 3 steps (0.8 g, 2.4 mmol).

^{1}H NMR (400 MHz. $CDCl_3$) δ 7.85 – 7.82 (m, 3H), 7.72 (s, 1H), 7.53 – 7.49 (m, 2H), 7.47 – 7.45 (m, 3H), 7.43 – 7.38 (m, 1H), 7.33 – 7.32 (m, 2H), 6.65 (dd, *J* = 9.7, 1.7 Hz, 1H), 4.66 (d, *J* = 9.7 Hz, 1H), 1.69 (bs, 2H).

^{13}C NMR (100 MHz, $CDCl_3$) δ 140.2, 138.9 (Cq, *J* = 5.2 Hz), 133.4, 132.9, 131.7, 130.8 (*J* = 30.0 Hz), 129.7, 128.9, 128.6, 127.9, 127.6, 127.3, 126.3, 126.0, 124.9, 124.7, 123.2 (*J* = 273.5 Hz), 53.2.

^{19}F NMR (376 MHz, $CDCl_3$) δ – 66.16 (s, CF3).

HRMS (ESI) *m/z*: Fragmentation observed: 311.0988 $[M - NH_2]^+$ corresponding to $C_{20}H_{14}F_3^+$, $C_{20}H_{14}F_3^+$ requires 311.1042.

***tert*-Butyl (*R*,*E*)-(4,4,4-trifluoro-1-(naphthalen-2-yl)-3-phenylbut-2-en-1-yl)carbamate (4f')**

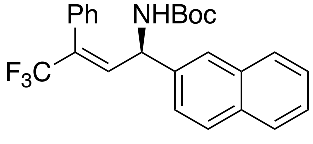

The title compound was obtained following GPB from (*R*,*E*)-4,4,4-trifluoro-1-(naphthalen-2-yl)-3-phenylbut-2-en-1-amine (60.0 mg, 0.18 mmol). The protected allylic amine was purified by FCC (pentane:EtOAc 95:5) as a white solid in 83% (65.2 mg, 0.15 mmol).

The enantiomeric excess (minor isomer: nd, major isomer 97%) was determined by HPLC (CHIRACEL® OD–H), hexane/iPrOH 98/2, 1 mL/min, (**1*R*,*E***) (major diastereomer): minor enantiomer t_r = 13.7 min, major enantiomer, t_r = 9.6 min.

$[\alpha]_D^{25}$: – 176.8 (c 1.00, $CHCl_3$, for the diastereomer mixture, d.r.: 97:3).

^{1}H NMR (400 MHz, $CDCl_3$) δ 7.84 – 7.79 (m, 3H), 7.58 (s, 1H), 7.51 – 7.49 (m, 2H), 7.41 – 7.38 (m, 3H), 7.30 – 7.26 (m, 3H), 6.64 (d, *J* = 9.2 Hz, 1H), 5.38 (bs, 1H), 5.04 (d, *J* = 7.6 Hz, 1H), 1.43 (s, 9H).

^{13}C NMR (100 MHz, $CDCl_3$) δ 154.5, 137.6, 135.2 (Cq, J = 5.7 Hz), 133.3, 132.9, 131.1, 129.6, 129.0, 128.9, 128.6, 127.9, 127.6, 126.4, 126.3, 125.4, 124.5, 123.2 (Cq, J = 273.5 Hz), 80.0, 52.8, 28.3.

^{19}F NMR (376 MHz, $CDCl_3$) δ – 66.20 (s, CF_3).

HRMS (ESI) *m/z*: 450.1653 $[M+Na]^+$, $C_{25}H_{24}F_3NNaO_2^+$ requires 450.1651.

(*R*,*E*)-4,4,4-Trifluoro-1-(3-methoxyphenyl)-3-phenylbut-2-en-1-amine (4g)

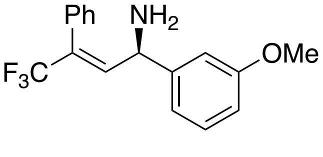

The title compound was obtained following GPA from (*E*)-4,4,4-trifluoro-1-(3-methoxyphenyl)-3-phenylbut-2-en-1-one (2.2 g, 7.2 mmol). The allylic amine was purified by FCC (pentane:EtOAc 7:3) as a yellow oil in 54% yield over 3 steps (1.2 g, 3.9 mmol).

^{1}H NMR (400 MHz. $CDCl_3$) δ 7.45 – 7.44 (m, 3H), 7.32 – 7.26 (m, 3H), 6.88 – 6.83 (m, 3H), 6.55 (dq, J = 9.9, 1.6 Hz, 1H), 4.47 (d, J = 9.9 Hz, 1H), 3.82 (s, 3H), 1.54 (bs, 2H).

^{13}C NMR (100 MHz, $CDCl_3$) δ 159.9, 144.4, 138.9 (Cq, J_{CF} = 5.2 Hz), 131.6, 130.5 (Cq, J_{CF} = 30.0 Hz), 129.8, 129.6, 128.8, 128.5, 123.2 (Cq, J_{CF} = 273.3 Hz), 118.6, 112.9, 112.1, 55.2, 53.0.

^{19}F NMR (376 MHz, $CDCl_3$) δ – 66.20 (s, CF_3).

***tert*-Butyl (*R*,*E*)-(4,4,4-trifluoro-1-(3-methoxyphenyl)-3-phenylbut-2-en-1-yl)carbamate (4g')**

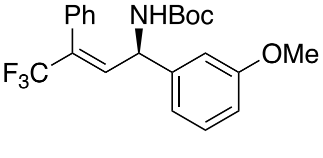

The title compound was obtained following GPB from (*R*,*E*)-4,4,4-trifluoro-1-(3-methoxyphenyl)-3-phenylbut-2-en-1-amine (76.8 mg, 0.25 mmol). The protected allylic amine was purified by FCC (pentane:EtOAc 95:5) as a white solid in 91% (92.5 mg, 0.23 mmol).

The enantiomeric excess (minor isomer: nd, major isomer 94%) was determined by HPLC (CHIRACEL® OD–H), hexane/iPrOH 98/2, 1 mL/min, (**1*R*,*E***) (major diastereomer): minor enantiomer t_r = 8.0 min, major enantiomer, t_r = 6.6 min.

$[\alpha]_D^{25}$: – 36.4 (c 0.77, $CHCl_3$, for the diastereomer mixture, d.r.: 97:3).

^{1}H NMR (400 MHz, $CDCl_3$) δ 7.40 – 7.35 (m, 3H), 7.28 – 7.22 (m, 3H), 6.81 (dd, J = 8.3, 2.5 Hz, 1H), 6.74 (d, J = 7.7 Hz, 1H), 6.67 (s, 1H), 6.50 (dd, J = 9.3, 1.9, 1H), 5.16 (bs, 1H), 4.93 (d, J = 7.4 Hz, 1H), 3.77 (s, 3H), 1.41 (s, 9H).

^{13}C NMR (100 MHz, $CDCl_3$) δ 159.9, 154.5, 141.7, 135.2 (Cq, J_{CF} = 5.3 Hz), 131.1, 130.0, 129.6, 128.9, 128.5, 123.1 (Cq, J_{CF} = 273.6 Hz), 119.1, 118.6, 113.2, 112.4, 79.9, 55.2, 52.6, 28.3.

^{19}F NMR (376 MHz, $CDCl_3$) δ – 66.28 (s, CF_3).

HRMS (ESI) *m/z*: 430.1595 $[M+Na]^+$, $C_{22}H_{24}F_3NNaO_3^+$ requires 430.1600.

(*R*,*E*)-4,4,4-Trifluoro-3-phenyl-1-(3-(trifluoromethyl)phenyl)but-2-en-1-amine (4h)

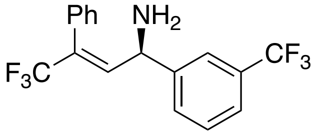

The title compound was obtained following GPA from (*E*)-4,4,4-trifluoro-3-phenyl-1-(3-(trifluoromethyl)phenyl)but-2-en-1-one (1.2 g, 3.5 mmol). The allylic amine was purified by FCC (pentane:EtOAc 7:3) as a yellow oil in 66% yield over 3 steps (0.8 g, 2.3 mmol).

^{1}H NMR (400 MHz. $CDCl_3$) δ 7.57 – 7.53 (m, 2H), 7.45 – 7.44 (m, 5H), 7.26 – 7.25 (m, 2H), 6.49 (dd, *J* = 9.6, 1.7 Hz, 1H), 4.55 (d, *J* = 9.6, 1H), 1.54 (bs, 2H).

^{13}C NMR (100 MHz, $CDCl_3$) δ 143.8, 138.3 (Cq, J_{CF} = 5.2 Hz), 131.5 (Cq, J_{CF} = 30.3 Hz), 131.4, 131.1 (J_{CF} = 32.3 Hz), 130.0, 129.5, 129.2, 129.1, 128.7, 124.5 (J_{CF} = 3.8 Hz), 123.4 (J_{CF} = 3.8 Hz), 124.0 (J_{CF} = 272.4 Hz), 123.1 (J_{CF} = 273.4 Hz), 52.8.

^{19}F NMR (376 MHz, $CDCl_3$) δ – 62.65 (s, CF3), – 66.50 (s, CF3).

HRMS (ESI) *m/z*: Fragmentation observed: 329.0884 $[M - NH_2]^+$ corresponding to $C_{17}H_{11}F_6^+$, $C_{17}H_{11}F_6^+$ requires 329.0759.

***tert*-Butyl (*R*,*E*)-(4,4,4-trifluoro-3-phenyl-1-(3-(trifluoromethyl)phenyl)but-2-en-1-yl)carbamate (4h')**

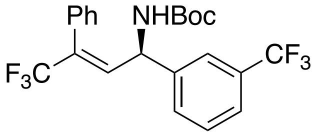

The title compound was obtained following GPB from (*R*,*E*)-4,4,4-trifluoro-3-phenyl-1-(3-(trifluoromethyl)phenyl)but-2-en-1-amine (86.3 mg, 0.25 mmol). The protected allylic amine was purified by FCC (pentane:EtOAc 95:5) as a white solid in 91% (93.5 mg, 0.21 mmol).

The enantiomeric excess (minor isomer: 99%, major isomer 94%) was determined by HPLC (CHIRACEL® OD–H), hexane/iPrOH 98/2, 1 mL/min, (**1*R*,*Z***) (minor diastereomer): minor enantiomer, t_r = 13.3 min, major enantiomer, t_r = 9.1 min, (**1*R*,*E***) (major diastereomer): minor enantiomer t_r = 6.5 min, major enantiomer, t_r = 5.6 min.

$[\alpha]_D^{25}$: – 50.8 (c 1.00, $CHCl_3$, for the diastereomer mixture, d.r.: 92:8).

^{1}H NMR (400 MHz, $CDCl_3$) δ 7.58 (d, *J* = 8.1 Hz, 2H), 7.42 – 7.41 (m, 3H), 7.28 – 7.25 (m, 4H), 6.49 (d, *J* = 9.3 Hz, 1H), 5.25 (bs, 1H), 5.00 (bs, 1H), 1.41 (s, 9H).

^{13}C NMR (100 MHz, $CDCl_3$) δ 154.4, 144.3, 134.1 (Cq, J_{CF} = 5.4 Hz), 130.8, 130.1 (Cq, J_{CF} = 32.4 Hz), 129.4, 129.2, 128.7, 128.3 (Cq, J_{CF} = 3.7 Hz), 126.92, 126.85, 125.9 (Cq, J_{CF} = 3.7 Hz), 123.3 (Cq, J_{CF} = 3.8 Hz), 122.9 (Cq, J_{CF} = 273.6 Hz), 80.4, 52.3, 28.2.

^{19}F NMR (376 MHz, $CDCl_3$) δ – 62.73(s, CF_3), – 66.54 (s, CF_3).

HRMS (ESI) *m/z*: 468.1364 $[M+Na]^+$, $C_{22}H_{21}F_6NNaO_2^+$ requires 468.1369.

(*R*,*E*)-4,4,4-Trifluoro-1-(2-methoxyphenyl)-3-phenylbut-2-en-1-amine (4i)

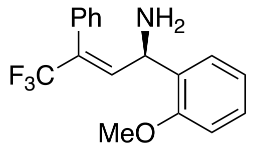

The title compound was obtained following GPA from (*E*)-4,4,4-trifluoro-1-(2-methoxyphenyl)-3-phenylbut-2-en-1-one (2.7 g, 8.7 mmol). The allylic amine was purified by FCC (pentane:EtOAc 7:3) as a yellow oil in 49% yield over 3 steps (1.3 g, 4.26 mmol).

^{1}H NMR (400 MHz. $CDCl_3$) δ 7.41 – 7.39 (m, 3H), 7.24 – 7.23 (m, 3H), 7.07 (dd, *J* = 7.5, 1.3 Hz, 1H), 6.91 (t, *J* = 7.8 Hz, 1H), 6.87 (d, *J* = 8.3 Hz, 1H), 6.76 (dd, *J* = 9.6, 1,2 Hz, 1H), 4.54 (d, *J* = 9.7 Hz, 1H), 3.79 (s, 3H), 1.77 (bs, 2H).

^{13}C NMR (100 MHz, $CDCl_3$) δ 156.9, 138.5 (Cq, J_{CF} = 5.3 Hz), 132.0, 131.1, 130.1 (Cq, J_{CF} = 29.7 Hz), 129.8, 128.63, 128.57, 128.3, 127.8, 123.5 (Cq, J_{CF} = 273.3 Hz), 120.9, 110.9, 55.1, 50.7.

^{19}F NMR (376 MHz, $CDCl_3$) δ – 66.06 (S, CF_3).

***tert*-Butyl (*R*,*E*)-(4,4,4-trifluoro-1-(2-methoxyphenyl)-3-phenylbut-2-en-1-yl)carbamate (4i')**

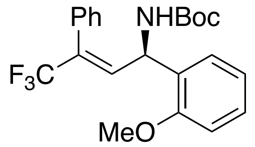

The title compound was obtained following GPB from (*R*,*E*)-4,4,4-trifluoro-1-(2-methoxyphenyl)-3-phenylbut-2-en-1-amine (76.8 mg, 0.25 mmol). The protected allylic amine was purified by FCC (pentane:EtOAc 95:5) as a white solid in 99% (100.5 mg, 0.25 mmol).

The enantiomeric excess (minor isomer: 94%, major isomer 75%) was determined by HPLC (CHIRACEL® IC), hexane/iPrOH 98/2, 1 mL/min, (**1*R*,*Z***) (minor diastereomer): minor enantiomer, t_r = 12.8 min, major enantiomer, t_r = 10.9 min, (**1*R*,*E***) (major diastereomer): minor enantiomer tr = 9.3 min, major enantiomer, tr = 8.6 min.

$[\alpha]_D^{25}$: – 24.3 (c 0.76, $CHCl_3$, for the diastereomer mixture, d.r.: 97:3).

^{1}H NMR (400 MHz, $CDCl_3$) δ 7.41 – 7.40 (m, 3H), 7.24 – 7.20 (m, 3H), 6.87 (d, *J* = 8.1 Hz, 1H), 6.82 – 6.75 (m, 3H), 5.59 (bs, 1H), 5.30 (bs, 1H), 3.84 (s, 3H), 1.42 (s, 9H).

^{13}C NMR (100 MHz, $CDCl_3$) δ 156.9, 154.4, 136.0, 131.4, 131.2 (Cq, J_{CF} = 30.1 Hz), 129.8, 129.2, 128.7, 128.3, 127.9, 123.3 (Cq, J_{CF} = 273.3 Hz), 120.9, 111.1, 79.5, 55.3, 51.5, 28.3.

^{19}F NMR (376 MHz, $CDCl_3$) δ – 66.40 (s, CF_3).

HRMS (ESI) *m/z*: 430.1595 $[M+Na]^+$, $C_{22}H_{24}F_3NNaO_3^+$ requires 430.1600.

(*S*,*E*)-5,5,5-Trifluoro-4-phenylpent-3-en-2-amine (4j)

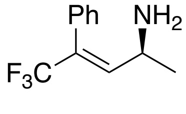

The title compound was obtained following GPA from (*E*)-5,5,5-trifluoro-4-phenylpent-3-en-2-one (2.0 g, 9.3 mmol). The allylic amine was purified by FCC (pentane:EtOAc 7:3) as a yellow oil in 30% yield over 3 steps (0.6 g, 2.8 mmol).

^{1}H NMR (400 MHz. $CDCl_3$) δ 7.41 – 7.37 (m, 3H), 7.24 – 7.22 (m, 2H), 6.26 (dq, *J* = 9.5, 1.6Hz, 1H), 3.44 (dt, *J* = 13.1, 6.6 Hz, 1H), 1.31 (bs, 2H), 1.14 (d, *J* = 6.6 Hz, 3H).

^{13}C NMR (100 MHz, $CDCl_3$) δ 141.3 (Cq, J_{CF} = 5.1 Hz), 131.9, 129.8 (Cq, J_{CF} = 29.9 Hz), 129.5, 128.6, 128.5, 123.3 (Cq, J_{CF} = 273.1 Hz), 44.8, 23.1.

^{19}F NMR (376 MHz, $CDCl_3$) δ – 66.30 (s, CF_3).

***tert*-Butyl (*S*,*E*)-(5,5,5-Trifluoro-4-phenylpent-3-en-2-yl)carbamate (4j')**

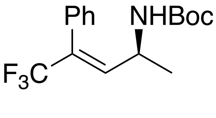

The title compound was obtained following GPB from (*S*,*E*)-5,5,5-trifluoro-4-phenylpent-3-en-2-amine (53.8 mg, 0.25 mmol). The protected allylic amine was purified by FCC (pentane:EtOAc 95:5) as a white solid in 99% (69.4 mg, 0.22 mmol).

The enantiomeric excess (minor isomer: nd, major isomer 28%) was determined by HPLC (CHIRACEL® OD–H), hexane/iPrOH 98/2, 1 mL/min, (**1*S*,*E***) (major diastereomer): minor enantiomer t_r = 9.17 min, major enantiomer, t_r = 6.85 min.

^{1}H NMR (400 MHz, $CDCl_3$) δ 7.41 – 7.39 (m, 3H), 7.32 – 7.31 (m, 2H), 6.25 (d, *J* = 8.8 Hz, 1H), 4.47 (bs, 1H), 4.17 (bs, 1H), 1.42 (s, 9H), 1.14 (d, *J* = 6.6 Hz, 3H).

^{13}C NMR (100 MHz, $CDCl_3$) δ 154.6, 137.9, 131.4, 129.5, 128.7, 128.5, 127.2, 123.2 (Cq, J_{CF} = 273.2 Hz), 44.9, 28.3, 21.0.

^{19}F NMR (376 MHz, $CDCl_3$) δ – 66.46 (s, CF_3).

HRMS (ESI) *m/z*: 338.1360 $[M+Na]^+$, $C_{16}H_{20}F_3NNaO_2^+$ requires 338.1338.

(*E*)-4,4,4-Trifluoro-3-phenylbut-2-en-1-amine (4k)

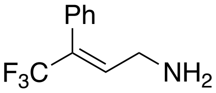

The title compound was obtained following GPA from (*E*)-4,4,4-trifluoro-3-phenylbut-2-enal (0.7 g, 3.5 mmol). The allylic amine was purified by FCC (pentane:EtOAc 7:3) as a yellow oil in 49% yield over 3 steps (0.35 g, 1.7 mmol).

^{1}H NMR (400 MHz. $CDCl_3$) δ 7.42 – 7.38 (m, 3H), 7.24 – 7.22 (m, 2H), 6.47 (t, *J* = 6.7 Hz, 1H), 3.27 (dd, *J* = 6.9, 2.2 Hz, 2H), 1.25 (bs, 2H).

^{13}C NMR (100 MHz, $CDCl_3$) δ 137.0 (Cq, J_{CF} = 5.3 Hz), 131.7, 131.3 (Cq, J_{CF} = 29.6 Hz), 129.4, 128.7, 128.5, 123.3 (Cq, J_{CF} = 273.1 Hz), 39.8.

^{19}F NMR (376 MHz, $CDCl_3$) δ – 66.01 (s, CF_3).

(*R*,*E*)-4,4,4-Trifluoro-1-phenyl-3-(*p*-tolyl)but-2-en-1-amine (4l)

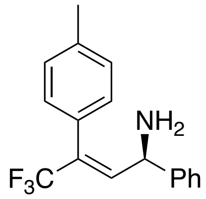

The title compound was obtained following GPA from (*E*)-4,4,4-trifluoro-1-phenyl-3-(*p*-tolyl)but-2-en-1-one (1.6 g, 5.5 mmol). The allylic amine was purified by FCC (pentane:EtOAc 7:3) as a yellow oil in 56% yield over 3 steps (0.9 g, 3.1 mmol).

^{1}H NMR (400 MHz. $CDCl_3$) δ 7.34 – 7.33 (m, 2H), 7.30 – 7.27 (m, 3H), 7.24 (d, *J* = 7.9 Hz, 2H), 7.16 (d, *J* = 7.9 Hz, 2H), 6.51 (dd, *J* = 9.8, 1.7 Hz, 1H), 4.50 (d, *J* = 9.8 Hz, 1H), 2.40 (s, 3H), 1.56 (bs, 2H).

^{13}C NMR (100 MHz, $CDCl_3$) δ 142.9, 138.8 (Cq, J_{CF} = 5.2 Hz), 138.7, 130.5 (Cq, J_{CF} = 29.8 Hz), 129.5, 129.2, 128.8, 128.7, 127.6, 126.4, 123.3 (Cq, J_{CF} = 273.3 Hz), 53.0, 21.3.

^{19}F NMR (376 MHz, $CDCl_3$) δ – 66.30 (s, CF_3).

***tert*-Butyl (*R*,*E*)-(4,4,4-trifluoro-1-phenyl-3-(*p*-tolyl)but-2-en-1-yl)carbamate (4l')**

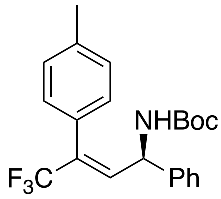

The title compound was obtained following GPB from (*R*,*E*)-4,4,4-trifluoro-1-phenyl-3-(*p*-tolyl)but-2-en-1-amine (72.8 mg, 0.25 mmol). The protected allylic amine was purified by FCC (pentane:EtOAc 95:5) as a white solid in 99% (93.2 mg, 0.24 mmol).

The enantiomeric excess (minor isomer: nd, major isomer 90%) was determined by HPLC (CHIRACEL® OD–H), hexane/iPrOH 98/2, 1 mL/min, (**1*R*,*E***) (major diastereomer): minor enantiomer t_r = 6.9 min, major enantiomer, t_r = 5.4 min.

$[\alpha]_D^{25}$: – 53.1 (c 0.98, $CHCl_3$, for the diastereomer mixture, d.r.: 87:13).

^{1}H NMR (400 MHz, $CDCl_3$) δ 7.38 – 7.28 (m, 3H), 7.21 – 7.15 (m, 6H), 6.49 (dq, *J* = 9.4, 1.6 Hz, 1H), 5.22 (bs, 1H), 4.90 (d, *J* = 7.2 Hz, 1H), 2.38 (s, 3H), 1.41 (s, 9H).

^{13}C NMR (100 MHz, $CDCl_3$) δ 154.5, 140.3, 138.8, 134.9 (Cq, J_{CF} = 4.7 Hz), 129.4, 129.2, 128.9, 128.1, 127.9, 126.5, 123.2 (Cq, J_{CF} = 273.6 Hz), 80.0, 52.6, 28.3, 21.3.

^{19}F NMR (376 MHz, $CDCl_3$) δ – 66.32 (s, CF_3).

HRMS (ESI) *m/z*: 414.1623 $[M+Na]^+$, $C_{22}H_{24}F_3NNaO_2^+$ requires 414.1651.

(*R*,*E*)-4,4,4-Trifluoro-3-(4-methoxyphenyl)-1-phenylbut-2-en-1-amine (4m)

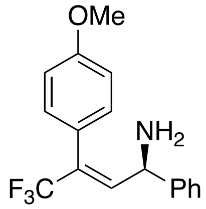

The title compound was obtained following GPA from (*E*)-4,4,4-trifluoro-3-(4-methoxyphenyl)-1-phenylbut-2-en-1-one (1.6 g, 5.5 mmol). The allylic amine was purified by FCC (pentane:EtOAc 7:3) as a yellow oil in 56% yield over 3 steps (0.9 g, 3.1 mmol).

^{1}H NMR (400 MHz. $CDCl_3$) δ 7.37 – 7.33 (m, 2H), 7.29 – 7.27 (m, 3H), 7.19 (d, *J* = 8.6 Hz, 1H), 6.94 (d, *J* = 8.6 Hz, 1H), 6.51 (d, *J* = 9.7, 1.5 Hz, 1H), 4.50 (d, *J* = 9.7 Hz, 1H), 3.85 (s, 3H), 1.55 (bs, 2H).

^{13}C NMR (100 MHz, $CDCl_3$) δ 159.9, 143.0, 138.9 (Cq, *J* = 5.1 Hz), 130.9, 130.1 (Cq, *J* = 29.9 Hz), 128.8, 127.6, 126.4, 123.7, 123.4 (Cq, *J* = 273.3 Hz), 113.9, 55.2, 53.1.

^{19}F NMR (376 MHz, $CDCl_3$) δ – 66.40 (s, CF_3).

***tert*-Butyl (*R*,*E*)-(4,4,4-trifluoro-3-(4-methoxyphenyl)-1-phenylbut-2-en-1-yl)carbamate (4m')**

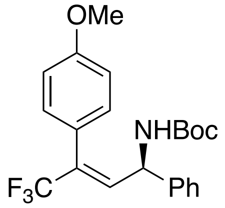

The title compound was obtained following GPB from (*R*,*E*)-4,4,4-trifluoro-3-(4-methoxyphenyl)-1-phenylbut-2-en-1-amine (76.8 mg, 0.25 mmol). The protected allylic amine was purified by FCC (pentane:EtOAc 95:5) as a white solid in 99% (86.2 mg, 0.21 mmol).

The enantiomeric excess (minor isomer: 98%, major isomer 93%) was determined by HPLC (CHIRACEL® OD–H), hexane/iPrOH 98/2, 1 mL/min, (**1*R*,*Z***) (minor diastereomer): minor enantiomer, t_r = 19.5 min, major enantiomer, t_r = 12.0 min, (**1*R*,*E***) (major diastereomer): minor enantiomer t_r = 8.4 min, major enantiomer, t_r = 6.3 min.

$[\alpha]_D^{25}$: – 60.0 (c 0.98, $CHCl_3$, for the diastereomer mixture, d.r.: 88:12).

^{1}H NMR (400 MHz, $CDCl_3$) δ 7.38 – 7.27 (m, 4H), 7.21 – 7.17 (m, 3H), 6.92 (d, *J* = 8.7 Hz, 2H), 6.50 (d, *J* = 9.2 Hz, 1H), 5.24 (bs, 1H), 5.04 (bs, 1H), 3.82 (s, 3H), 1.42 (s, 9H).

^{13}C NMR (100 MHz, $CDCl_3$) δ 159.9, 154.5, 140.3, 135.1 (Cq, J_{CF} = 5.5 Hz), 130.8, 128.9, 127.8, 127.3, 126.5, 123.2 (Cq, J_{CF} = 273.5 Hz), 123.2, 113.9, 79.9, 55.1, 52.6, 28.3.

^{19}F NMR (376 MHz, $CDCl_3$) δ – 66.36 (s, CF_3).

HRMS (ESI) *m/z*: 430.1562 $[M+Na]^+$, $C_{21}H_{22}F_3NNaO_2^+$ requires 430.1600.

(*R*,*E*)-4,4,4-Trifluoro-1-phenyl-3-(4-(trifluoromethyl)phenyl)but-2-en-1-amine (4n)

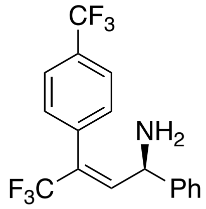

The title compound was obtained following GPA from (*E*)-4,4,4-trifluoro-1-phenyl-3-(4-(trifluoromethyl)phenyl)but-2-en-1-one (0.8 g, 2.3 mmol). The allylic amine was purified by FCC (pentane:EtOAc 7:3) as a yellow oil in 52% yield over 3 steps (0.4 g, 1.2 mmol).

^{1}H NMR (400 MHz. $CDCl_3$) δ 7.60 (d, *J* = 8.1 Hz, 2H), 7.45 – 7.44 (m, 3H), 7.40 (d, *J* = 8.1 Hz, 2H), 7.27 – 7.26 (m, 2H), 6.50 (dd, *J* = 9.7, 1.7 Hz, 1H), 4.55 (d, *J* = 9.7 Hz, 1H), 1.56 (bs, 2H).

^{13}C NMR (100 MHz, $CDCl_3$) δ 146.7, 138.2 (Cq, J_{CF} = 5.2 Hz), 131.5 (Cq, J_{CF} = 30.2 Hz), 131.4, 129.9 (Cq, J_{CF} = 32.5 Hz), 129.5, 129.0, 128.7, 126.9, 125.7 (Cq, J_{CF} = 3.8 Hz), 124.0 (Cq, J_{CF} = 272.0 Hz), 123.1 (Cq, J_{CF} = 273.4 Hz), 52.8.

^{19}F NMR (376 MHz, $CDCl_3$) δ – 66.55 (s, CF_3), – 66.46 (s, CF_3).

***tert*-Butyl (*R*,*E*)-(4,4,4-trifluoro-1-phenyl-3-(4-(trifluoromethyl)phenyl)but-2-en-1-yl)carbamate (4n')**

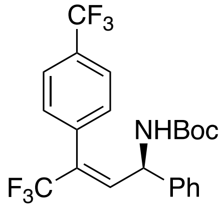

The title compound was obtained following GPB from (*R*,*E*)-4,4,4-trifluoro-1-phenyl-3-(4-(trifluoromethyl)phenyl)but-2-en-1-amine (86.3 mg, 0.25 mmol). The protected allylic amine was purified by FCC (pentane:EtOAc 95:5) as a white solid in 82% (91.3 mg, 0.21 mmol).

The enantiomeric excess (minor isomer: nd, major isomer 94%) was determined by HPLC (CHIRACEL® OD–H), hexane/iPrOH 98/2, 1 mL/min, (**1*R*,*E***) (major diastereomer): minor enantiomer t_r = 6.9 min, major enantiomer, t_r = 5.1 min.

$[\alpha]_D^{25}$: – 32.8 (c 0.78, $CHCl_3$, for the diastereomer mixture, d.r.: 95:5).

^{1}H NMR (400 MHz, $CDCl_3$) δ 7.66 (d, *J* = 8.1 Hz, 2H), 7.41 (d, *J* = 7.9 Hz, 2H), 7.36 – 7.30 (m, 3H), 7.15 – 7.12 (m, 2H), 6.61 (dd, *J* = 9.4, 1.8 Hz, 1H), 5.13 (bs, 1H), 4.90 (d, *J* = 7.4 Hz, 1H), 1.42 (s, 9H).

^{13}C NMR (100 MHz, $CDCl_3$) δ 154.5, 139.5, 136.6 (Cq, J_{CF} = 4.4 Hz), 134.9, 131.13 (Cq, J_{CF} = 32.5 Hz), 131.06 (Cq, J_{CF} = 30.3 Hz), 130.2, 129.1, 128.2, 126.6, 125.5 (Cq, J_{CF} = 3.8 Hz), 123.9 (Cq, J_{CF} = 272.3 Hz), 122.8 (Cq, J_{CF} = 273.5 Hz), 80.2, 52.7, 28.3.

^{19}F NMR (376 MHz, $CDCl_3$) δ – 62.85 (s, CF_3), – 66.14 (s, CF_3).

HRMS (ESI) *m/z*: 468.1369 $[M+Na]^+$, $C_{22}H_{21}F_6NNaO_2^+$ requires 468.1369.

(*R*,*E*)-4,4,4-Trifluoro-1-phenyl-3-(*m*-tolyl)but-2-en-1-amine (4o)

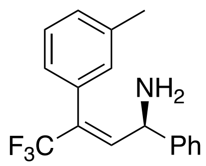

The title compound was obtained following GPA from (*E*)-4,4,4-trifluoro-1-phenyl-3-(*m*-tolyl)but-2-en-1-one (2.0 g, 6.9 mmol). The allylic amine was purified by FCC (pentane:EtOAc 7:3) as a yellow oil in 50% yield over 3 steps (1.0 g, 3.4 mmol).

^{1}H NMR (400 MHz. $CDCl_3$) δ 7.38 – 7.27 (m, 6H), 7.25 – 7.23 (m, 1H), 7.08 – 7.06 (m, 2H), 6.53 (dq, *J* = 9.7, 1.6 Hz, 1H), 4.49 (d, *J* = 9.7 Hz, 1H), 2.39 (s, 3H), 1.56 (bs, 2H).

^{13}C NMR (100 MHz, $CDCl_3$) δ 142.9, 138.8 (Cq, J_{CF} = 5.2 Hz), 138.2, 131.6, 130.6 (Cq, J_{CF} = 29.9 Hz), 130.2, 129.6, 128.8, 128,4, 127.6, 126.7, 126.5, 123.3 (Cq, J_{CF} = 273.3 Hz), 53.0, 21.4.

^{19}F NMR (376 MHz, $CDCl_3$) δ – 66.18 (s, CF_3).

***tert*-Butyl (*R*,*E*)-(4,4,4-trifluoro-1-phenyl-3-(*m*-tolyl)but-2-en-1-yl)carbamate (4o')**

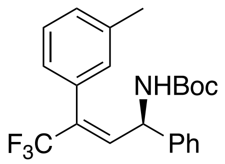

The title compound was obtained following GPB from (*R*,*E*)-4,4,4-trifluoro-1-phenyl-3-(4-(trifluoromethyl)phenyl)but-2-en-1-amine (72.8 mg, 0.25 mmol). The protected allylic amine was purified by FCC (pentane:EtOAc 95:5) as a white solid in 94% (92.3 mg, 0.24 mmol).

The enantiomeric excess (minor isomer: 93%, major isomer 89%) was determined by HPLC (CHIRACEL® OD–H), hexane/iPrOH 98/2, 1 mL/min, (**1*R*,*Z***) (minor diastereomer): minor enantiomer, t_r = 9.92 min, major enantiomer, t_r = 6.87 min, (**1*R*,*E***) (major diastereomer): minor enantiomer t_r = 5.78 min, major enantiomer, t_r = 4.93 min.

$[\alpha]_D^{25}$: – 54.2 (c 1.00, $CHCl_3$, for the diastereomer mixture, d.r.: 89:11).

^{1}H NMR (400 MHz, $CDCl_3$) δ 7.38 – 7.27 (m, 4H), 7.22 – 7. 20 (m, 1H), 7.17 – 7.15 (m, 2H), 7.08 – 7.05 (m, 2H), 6.52 (dd, *J* = 9.3, 1.9 Hz, 1H), 5.21 (bs, 1H), 4.99 (d, *J* = 7.6 Hz, 1H), 2.35 (s, 3H), 1.42 (s, 9H).

^{13}C NMR (100 MHz, $CDCl_3$) δ 154.4, 140.4, 138.1, 135.1, 132.4 (Cq, J_{CF} = 29.7 Hz), 131.0, 130.2, 129.7, 128.8, 128.3, 127.8, 126.6, 126.5, 123.2 (Cq, J_{CF} = 273.5 Hz), 80.0, 52.6, 28.3, 21.3.

^{19}F NMR (376 MHz, $CDCl_3$) δ – 66.16 (s, CF_3).

HRMS (ESI) *m/z*: 414.1652 $[M+Na]^+$, $C_{21}H_{22}F_3NNaO_2^+$ requires 414.1651.

(*R*,*Z*)-4,4,4-Trifluoro-1-phenyl-3-(thiophen-2-yl)but-2-en-1-amine (4p)

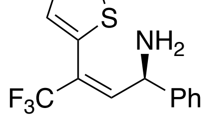

The title compound was obtained following GPA from (*Z*)-4,4,4-trifluoro-1-phenyl-3-(thiophen-2-yl)but-2-en-1-one (1.5 g, 5.3 mmol). The allylic amine was purified by FCC (pentane:EtOAc 7:3) as a yellow oil in 47% yield over 3 steps (0.7 g, 2.5 mmol).

^{1}H NMR (400 MHz. $CDCl_3$) δ 7.44 (d, *J* = 4.8 Hz, 1H), 7.40 – 7.29 (m, 5H), 7.11 – 7.09 (m, 2H), 6.62 (d, *J* = 9.7 Hz, 1H), 4.80 (d, *J* = 9.7 Hz, 1H), 1.60 (bs, 2H).

^{13}C NMR (100 MHz, $CDCl_3$) δ 142.4, 141.3 (Cq, J_{CF} = 4.9 Hz), 130.9, 129.5, 128.8, 127.7, 127.5, 127.2, 126.5, 123.7 (Cq, J_{CF} = 31.2 Hz), 122.7 (Cq, J_{CF} = 273.5 Hz), 53.1.

^{19}F NMR (376 MHz, $CDCl_3$) δ – 66.58 (s, CF_3).

***tert*-Butyl (R,Z)-(4,4,4-trifluoro-1-phenyl-3-(thiophen-2-yl)but-2-en-1-yl)carbamate (4p')**

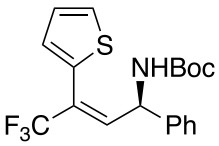

The title compound was obtained following GPB from (*R*,*E*)-4,4,4-trifluoro-1-phenyl-3-(4-(trifluoromethyl)phenyl)but-2-en-1-amine (70.8 mg, 0.25 mmol). The protected allylic amine was purified by FCC (pentane:EtOAc 95:5) as a white solid in 98% (94.4 mg, 0.24 mmol).

The enantiomeric excess (minor isomer: nd, major isomer 95%) was determined by HPLC (CHIRACEL® OD–H), hexane/iPrOH 98/2, 1 mL/min, (**1*R*,*E***) (major diastereomer): minor enantiomer t_r = 9.2 min, major enantiomer, t_r = 7.3 min.

$[\alpha]_D^{25}$: – 49.5 (c 1.00, $CHCl_3$, for the diastereomer mixture, d.r.: 96:4).

^{1}H NMR (400 MHz, $CDCl_3$) δ 7.42 (dd, *J* = 5.0, 1.3 Hz, 1H), 7.38 – 7.28 (m, 3H), 7.24 – 7.23 (m, 2H), 7.09 (bs, 1H), 7.06 (dd, *J* = 5.1, 3.6 Hz, 1H), 6.58 (dd, *J* = 9.2, 0.8 Hz, 1H), 5.52 (bs, 1H), 4.95 (dd, *J* = 7.7 Hz, 1H), 1.42 (s, 9H).

^{13}C NMR (100 MHz, $CDCl_3$) δ 154.5, 139.9, 137.0 (Cq, J_{CF} = 5.5 Hz), 130.4, 129.8, 129.0, 128.1, 127.8, 127.1, 126.7, 126.6, 122.7 (Cq, J_{CF} = 273.9 Hz), 80.2, 52.7, 28.3.

^{19}F NMR (376 MHz, $CDCl_3$) δ – 65.51 (s, CF_3).

HRMS (ESI) *m/z*: 406.1024 $[M+Na]^+$, $C_{19}H_{20}F_3NNaO_2S^+$ requires 406.1059.

4,4,4-Trifluoro-1-phenylbut-2-en-1-amine (4q)

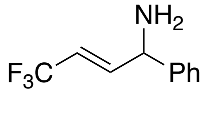

The title compound was obtained following GPA from (*E*)-4,4,4-trifluoro-1-phenylbut-2-en-1-one (0.7 g, 3.5 mmol). The allylic amine was purified by FCC (pentane:EtOAc 7:3) as a yellow oil in 33% yield over 3 steps (0.3 g, 1.2 mmol).

^{1}H NMR (400 MHz. $CDCl_3$) δ 7.39 – 7.36 (m, 2H), 7.32 – 7.29 (m, 3H), 6.55 (ddq, *J* = 15.7, 5.5, 2.1 Hz, 1H), 5.92 (dqd, *J* = 15.7, 6.4, 1,7 Hz, 1H), 4.65 (dp, *J* = 4.5, 2.2 Hz, 1H), 1.59 (bs, 2H).

^{13}**C NMR** (100 MHz, $CDCl_3$) δ 143.3 (Cq, J_{CF} = 6.1 Hz), 142.3, 128.9, 127.9, 126.7, 123.3 (Cq, J_{CF} = 269.4 Hz), 117.6 (Cq, J_{CF} = 33.4 Hz), 56.2.

^{19}**F NMR** (376 MHz, $CDCl_3$) δ – 63.79 (dt, J = 6.4, 2.1 Hz, CF_3).

Characterization of amines 6 and 6'

HRMS of **6a-6q** could not be obtained due to decomposition of the compounds. Instead, the HRMS of the **6a'-6p'** (protected amines) are reported. **6q** HRMS could not be given, a fragmentation is observed. HPLC analysis and α values of **6a-6p** is not reported here. Instead, the HPLC analysis and α values of **6a'-6p'** is reported.

<u>4,4,4-Trifluoro-1,3-diphenylbutan-1-amine (6a)</u>

The titled compound was obtained following GPC from **4a** (69.3 mg, 0.25 mmol) as a colourless oil (83% NMR yield). The diastereomers were purified by FCC using (pentane:EtOAc 8:2 to 6:4). The diastereomers were obtained in a ratio 75:25 in 80% isolated yield. Minor diastereomer **(1*S*,3*R*)-6a** isolated 20 mg (0.07 mmol) 28% yield, major diastereomer **(1*R*,3*R*)-6a** isolated 36 mg (0.13 mmol) 52% yield.

Minor diastereomer (configuration 1*S*, 3*R*)-6a

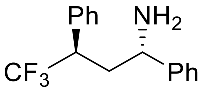

^{1}**H NMR** (400 MHz, $CDCl_3$) δ 7.42 – 7.33 (m, 5H), 7.32 – 7.30 (m, 2H), 7.24 – 7.19 (m, 3H), 3.79 – 3.71 (m, 1H), 3.56 (t, J = 7.6 Hz, 1H), 2.24 – 2.50 (m, 2H), 1.40 (bs, 2H).

^{13}**C NMR** (100 MHz, $CDCl_3$) δ 146.4, 134.4 (Cq, J_{CF} = 2,1 Hz), 129.2, 128.8, 128.7, 128.3, 127.2, 125.7, 124.3 (Cq, J_{CF} = 272.8 Hz), 52.5, 47.3 (Cq, J_{CF} = 26.7 Hz), 38.3 (Cq, J_{CF} = 2.1 Hz).

^{19}**F NMR** (376 MHz, $CDCl_3$) δ – 69.47 (d, J = 9.7 Hz, CF_3).

Major diastereomer (configuration 1*R*, 3*R*)-6a

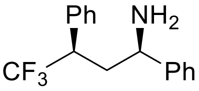

^{1}**H NMR** (400 MHz, $CDCl_3$) δ 7.41 – 7.34 (m, 5H), 7.32 – 7.28 (m, 1H), 7.24 – 7.22 (m, 2H), 7.19 – 7.17 (m, 2H), 3.65 – 3.61 (m, 1H), 3.04 – 2.93 (m, 1H), 2.39 – 2.32 (m, 2H), 1.52 (bs, 2H).

^{13}**C NMR** (100 MHz, $CDCl_3$) δ 144.4, 134.3 (Cq, J_{CF} = 2.0 Hz), 129.2, 128.8, 128.7, 128.3, 127.7, 126.8 (Cq, J_{CF} = 278.0 Hz), 126.6, 53.6, 47.4 (Cq, J_{CF} = 26.8 Hz), 37.9.

^{19}**F NMR** (376 MHz, $CDCl_3$) δ – 69.91 (d, J = 9.5 Hz, CF_3).

HRMS (ESI) *m/z*: 280.1336 $[M+H]^+$, $C_{16}H_{17}F_3N^+$ requires 280.1308.

***tert*-Butyl 4,4,4-trifluoro-1,3-diphenylbutyl)carbamate (6a')**

The titled compound was obtained following GPB from **6a** and purified by FCC using (pentane:EtOAc 95:5). Minor diastereomer **(1*S*,3*R*)-6a'**, isolated yield 57% (15.1 mg, 0.04 mmol) from 20 mg (0.07 mmol) of **(1*S*,3*R*)-6a**. Major diastereomer **(1*R*,3*R*)-6a'**, isolated yield 72% (34.1 mg, 0.09 mmol) from 36 mg (0.13 mmol) of **(1*R*,3*R*)-6a**.

The enantiomeric excess (minor isomer: 94%, major isomer 90%) was determined by HPLC (CHIRACEL® OD–H), hexane/iPrOH 98/2, 1 mL/min, λ = 210 nm, **1*S*,3*R*** (minor diastereomer): minor enantiomer, t_r = 7.2 min, major enantiomer, t_r = 5.9 min, **1*R*,3*R*** (major diastereomer): minor enantiomer t_r = 6.1 min, major enantiomer, t_r = 7.8 min.

Minor diastereomer (configuration 1*S*, 3*R*)-6a'

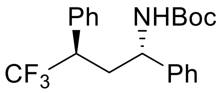

$[\alpha]_D^{25}$: – 5.4 (c 0.5, $CHCl_3$, ee 94%).

^{1}H NMR (400 MHz, $CDCl_3$) δ (*Rotamers are observed*) 7.38 – 7.29 (m, 8H), 7.16 – 7.14 (m, 2H), 4.99 – 4.76 (m, 1H), 4.50 – 4.28 (m, 1H), 3.47 (bs, 1H), 2.39 – 2.31 (m, 2H), 1.44 – 1.27 (m, 9H).

^{13}C NMR (100 MHz, $CDCl_3$) δ 154.9, 142.2, 134.0, 129.1, 128.8, 128.7, 128.3, 127.5, 126,9 (Cq, J_{CF} = 279.9 Hz), 125.9, 79.7, 51.5, 47.3 (Cq, J_{CF} = 26.7 Hz), 36.8, 28.3.

^{19}F NMR (376 MHz, $CDCl_3$) δ – 69.31 (d, J = 9.7 Hz, CF_3).

Major diastereomer (configuration 1*R*, 3*R*)-6a'

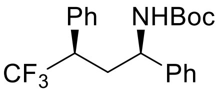

$[\alpha]_D^{25}$: – 1.4 (c 0.71, $CHCl_3$, *ee* 90%).

^{1}H NMR (400 MHz, $CDCl_3$) δ 7.39 – 7.31 (m, 6H), 7.23 – 7.21 (m, 2H), 7.13 – 7.11 (m, 2H), 4.71 (d, J = 6.3 Hz, 1H), 4.36 (bs, 1H), 3.00 – 2.89 (m, 1H), 2.62 (bs, 1H), 2.35 (ddd, J = 13.7, 10.3, 3.6 Hz, 1H), 1.37 (bs, 9H).

^{13}C NMR (100 MHz, $CDCl_3$) δ 154.6, 140.3, 133.3 (Cq, J_{CF} = 2.1 Hz), 129.3, 129.0, 128.8, 128.5, 128.1, 126.6 (Cq, J_{CF} = 278.0 Hz), 126.8, 79.6, 53.0, 47.2 (Cq, J_{CF} = 27.9 Hz), 34.6, 28.3.

^{19}F NMR (376 MHz, $CDCl_3$) δ – 70.20 (bs, CF_3).

HRMS (ESI) *m/z*: 402.1647 $[M+Na]^+$, $C_{21}H_{24}F_3NNaO_2^+$ requires 402.1651.

4,4,4-Trifluoro-3-phenyl-1-(*p*-tolyl)butan-1-amine (6b)

The titled compound was obtained following GPC from **4b** (72.8 mg, 0.25 mmol) as a colourless oil (90% NMR yield). The diastereomers were purified by FCC using (pentane:EtOAc 8:2 to 6:4). The diastereomers were obtained in a ratio 67:33 in 61% yield. Minor diastereomer could not be purified. Major diastereomer **(1*R*,3*R*)-6b** isolated 45 mg (0.15 mmol) 61% yield.

Major diastereomer (configuration 1*R*, 3*R*)-6b

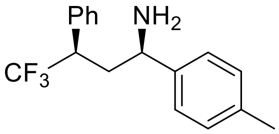

^{1}H NMR (400 MHz, $CDCl_3$) δ 7.38 – 7.35 (m, 3H), 7.23 – 7.22 (m, 2H), 7.16 (d, *J* = 7.9 Hz, 2H), 7.06 (d, *J* = 7.9 Hz, 2H), 3.57 (dd, *J* = 8.7, 6.4 Hz, 1H), 3.02 – 2.91 (m, 1H), 2.36 – 2.31 (m, 5H), 1.51 (bs, 2H).

^{13}C NMR (100 MHz, $CDCl_3$) δ 141.4, 137.3, 134.4 (q, *J* = 2.1 Hz), 129.5, 129.2, 128.7, 128.3, 126.8 (q, *J* = 278.0 Hz), 126.5, 53.3, 47.4 (Cq, J_{CF} = 26.8 Hz), 37.9, 21.1.

^{19}F NMR (376 MHz, $CDCl_3$) δ – 69.91 (d, *J* = 9.5 Hz, CF_3).

***tert*-Butyl (4,4,4-trifluoro-3-phenyl-1-(*p*-tolyl)butyl)carbamate (6b')**

The titled compound was obtained following GPB from **6b** and purified by FCC using (pentane:EtOAc 95:5). Major diastereomer **(1*R*,3*R*)-6b'**, isolated yield 56% (32.4 mg, 0.09 mmol) from 45 mg (0.15 mmol) of **6b**.

The enantiomeric excess (minor isomer: nd, major isomer 67%) was determined by HPLC (CHIRALPAK® IF), hexane/iPrOH 98/2, 1 mL/min, λ = 210 nm, **1*R*,3*R*** (major diastereomer): minor enantiomer tr = 7.1 min, major enantiomer, tr = 6.5 min.

Major diastereomer (configuration 1*R*, 3*R*)-6b'

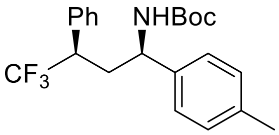

$[\alpha]_D^{25}$: – 7.8 (c 0.63, $CHCl_3$, *ee* 67%).

^{1}H NMR (400 MHz, $CDCl_3$) δ 7.40 – 7.34 (m, 3H), 7.26 – 7.23 (m, 2H), 7.16 (d, *J* = 7.8 Hz, 2H), 7.00 (d, *J* = 7.8 Hz, 2H), 4.68 (bs, 1H), 4.31 (bs, 1H), 2.99 – 2.88 (m, 1H), 2.62 (bs, 1H), 2.36 – 2.29 (m, 4H), 1.37 (s, 9H).

^{13}C NMR (100 MHz, $CDCl_3$) δ 154.7, 137.8, 137.3, 133.4, 130.8, 129.6, 129.3, 128.8, 128.4, 126.7, 126.65 (Cq, *J* = 279.8 Hz), 79.5, 52.7, 47.2 (Cq, *J* = 26.9 Hz), 34.6, 28.3, 21.1.

^{19}F NMR (376 MHz, $CDCl_3$) δ – 70.18 (bs, CF_3).

HRMS (ESI) *m/z*: 416.1871 $[M+Na]^+$, $C_{22}H_{26}F_3NNaO_2^+$ requires 416.1871.

4,4,4-Trifluoro-1-(4-methoxyphenyl)-3-phenylbutan-1-amine (6c)

The titled compound was obtained following GPC from **4c** (76.8 mg, 0.25 mmol) as a colourless oil (68% NMR yield). The diastereomers were purified by FCC using (pentane:EtOAc 8:2 to 6:4). The diastereomers were obtained in a ratio 65:35 in 35% yield. Minor diastereomer could not be purified. Major diastereomer **(1*R*,3*R*)-6c** isolated 27 mg (0.09 mmol) 35% yield.

Major diastereomer (configuration 1*R*, 3*R*)-6c

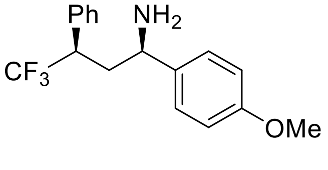

^{1}H NMR (400 MHz, $CDCl_3$) δ 7.40 – 7.33 (m, 3H), 7.23 – 7.21 (m, 2H), 7.09 (d, *J* = 8.7 Hz, 2H), 6.89 (d, *J* = 8.7 Hz, 2H), 3.82 (s, 3H), 3.57 (dd, *J* = 9.6, 5.4 Hz, 1H), 2.94 (ddq, *J* = 14.2, 9.6, 4.8 Hz, 1H), 2.34 (m, 2H), 1.50 (bs, 2H).

^{13}C NMR (100 MHz, $CDCl_3$) δ 159.0, 136.3, 134.3 (Cq, *J* = 1.9 Hz), 129.2, 128.7, 128.3, 127.7, 126.8 (Cq, *J* = 279.7 Hz), 114.2, 55.3, 52.9, 47.4 (Cq, *J* = 26.8 Hz), 38.0.

^{19}F NMR (376 MHz, $CDCl_3$) δ – 69.92 (d, *J* = 9.5 Hz, CF_3).

***tert*-Butyl (4,4,4-trifluoro-1-(4-methoxyphenyl)-3-phenylbutyl)carbamate (6c')**

The titled compound was obtained following GPB from **6c** and purified by FCC using (pentane:EtOAc 95:5). Major diastereomer **(1*R*,3*R*)-6c'**, isolated yield 85% (30.0 mg, 0.08 mmol) from 27 mg (0.15 mmol) of **6c**.

The enantiomeric excess (minor isomer: nd, major isomer 90%) was determined by HPLC (CHIRACEL® OD–H), hexane/iPrOH 98/2, 1 mL/min, λ = 210 nm, **1*R*,3*R*** (major diastereomer): minor enantiomer t_r = 9.2 min, major enantiomer, t_r = 10.4 min.

Major diastereomer (configuration 1*R*, 3*R*)-6c'

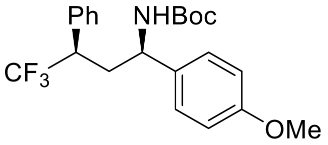

$[\alpha]_D^{25}$: – 16.8 (c 0.70, $CHCl_3$, *ee* 90%).

^{1}H NMR (400 MHz, $CDCl_3$) δ 7.40 – 7.33 (m, 3H), 7.22 – 7.02 (m, 2H), 7.03 (d, *J* = 8.5 Hz, 2H), 6.88 7.03 (d, *J* = 8.5 Hz, 2H), 4.65 (bs, 1H), 4.28 (bs, 1H), 3.82 (s, 3H), 2.97 – 2.87 (m, 1H), 2.62 (bs, 1H), 2.30 (ddd, *J* = 13.6, 10.7, 3.3 Hz, 1H), 1.37 (s, 9H).

^{13}C NMR (100 MHz, $CDCl_3$) δ 159.3, 154.6, 133.3, 132.3, 129.3, 128.8, 128.4, 128.0, 126.7 (Cq, *J* = 279.6 Hz), 114.3, 79.6, 55.3, 52.3, 47.2 (Cq, *J* = 26.4 Hz), 34.5, 28.3.

^{19}F NMR (376 MHz, $CDCl_3$) δ – 70.18 (d, *J* = 9.3 Hz, CF_3).

HRMS (ESI) *m/z*: 432.1757 $[M+Na]^+$, $C_{22}H_{26}F_3NNaO_3^+$ requires 432.1785.

1-(4-Bromophenyl)-4,4,4-trifluoro-3-phenylbutan-1-amine (6d)

The titled compound was obtained following GPC from **4d** (89 mg, 0.25 mmol) as a colourless oil (67% NMR yield). The diastereomers were purified by FCC using (pentane:EtOAc 8:2 to 6:4). The diastereomers were obtained in a ratio 74:26 in 67% yield. Minor diastereomer could not be purified. Major diastereomer **(1*R*,3*R*)-6d** isolated 39.6 mg (0.09 mmol) 44% yield.

Reaction 1g scale

The titled compound was obtained following GPC from **4d** (1.0 g, 2.81 mmol) as a colourless oil (73% NMR yield). The diastereomers were purified by FCC using (pentane:EtOAc 8:2 to 6:4). The diastereomers were obtained in a ratio 70:30 in 68% yield. Minor diastereomer could not be purified. Major diastereomer **(1*R*,3*R*)-6d** isolated 387.5 mg (1.08 mmol) 38% yield.

Major diastereomer (configuration 1*R*, 3*R*)-6d

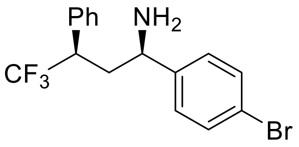

^{1}H NMR (400 MHz, $CDCl_3$) δ 7.48 (d, *J* = 8.4 Hz, 2H), 7.40 – 7.36 (m, 3H), 7.22 – 7.20 (m, 2H), 7.05 (d, *J* = 8.4 Hz, 2H), 3.62 (dd, *J* = 9.7, 5.3 Hz, 1H), 2.94 (ddp, *J* = 18.6, 9.5, 4.8, 4.3 Hz, 1H), 2.31 (dqt, *J* = 23.0, 9.5, 4.7 Hz, 1H), 1.51 (bs, 2H).

^{13}C NMR (100 MHz, $CDCl_3$) δ 143.3, 134.0 (Cq, *J* = 2.0 Hz), 131.9, 129.1, 128.8, 128.41, 128.37, 126.6 (Cq, *J* = 279.8 Hz), 121.4, 53.1, 47.3 (q, *J* = 26.9 Hz), 38.8.

^{19}F NMR (376 MHz, $CDCl_3$) δ – 69.98 (d, *J* = 9.5 Hz, CF_3).

***tert*-Butyl (1-(4-bromophenyl)-4,4,4-trifluoro-3-phenylbutyl)carbamate (6d')**

The titled compound was obtained following GPB from **6d** and purified by FCC using (pentane:EtOAc 95:5). Major diastereomer **(1*R*,3*R*)-6d'**, isolated yield 91% (46.1 mg, 0.10 mmol) from 39.6 mg (0.11 mmol) of **6d**.

The titled compound was obtained following GPB from **6d** and purified by FCC using (pentane:EtOAc 95:5). Major diastereomer **(1*R*,3*R*)-6d'**, isolated yield 87% (432.1 mg, 0.94 mmol) from 387.5 mg (1.08 mmol) of **6d**.

The enantiomeric excess (minor isomer: nd, major isomer 86%) was determined by HPLC (CHIRACEL® OD–H), hexane/iPrOH 98/2, 1 mL/min, λ = 210 nm, **1*R*,3*R*** (major diastereomer): minor enantiomer t_r = 9.0 min, major enantiomer, t_r = 12.4 min.

Major diastereomer (configuration 1*R*, 3*R*)-6d'

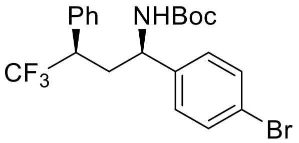

$[\alpha]_D^{25}$: – 37.1 (c 0.93, $CHCl_3$, *ee* 86%).

^{1}H NMR (400 MHz, $CDCl_3$) δ 7.48 (d, *J* = 8.4 Hz, 2H), 7.41 – 7.37 (m, 3H), 7.22 – 7.20 (m, 2H), 7.00 (d, *J* = 8.1 Hz, 2H), 4.70 (d, *J* = 7.4 Hz, 1H), 4.32 (bs, 1H), 2.92 (dtd, *J* = 18.3, 9.2, 4.6 Hz, 1H), 2.55 (bs, 1H), 2.30 (ddd, *J* = 13.7, 10.2, 3.6 Hz, 1H), 1.36 (s, 9H).

^{13}C NMR (100 MHz, $CDCl_3$) δ 154.5, 139.6, 133.1, 132.1, 129.2, 128.9, 128.6, 128.5, 126.5 (Cq, *J* = 279.8 Hz), 121.9, 79.9, 52.4, 47.15 (Cq, *J* = 29.4 Hz), 34.6, 28.3.

^{19}F NMR (376 MHz, $CDCl_3$) δ – 70.22 (d, *J* = 9.2 Hz, CF_3).

HRMS (ESI) *m/z*: 480.0733 $[M+Na]^+$, $C_{21}H_{23}F_3BrNNaO_2^+$ requires 480.0756.

4,4,4-Trifluoro-3-phenyl-1-(4-(trifluoromethyl)phenyl)butan-1-amine (6e)

The titled compound was obtained following GPC from **4e** (86.3 mg, 0.25 mmol) as a colourless oil (60% NMR yield). The diastereomers were purified by FCC using (pentane:EtOAc 8:2 to 6:4). The diastereomers were purified by FCC using (pentane:EtOAc 8:2 to 6:4). The diastereomers were obtained

in a ratio 65:35 in 54% yield. Minor diastereomer could not be purified. Major diastereomer **(1*R*,3*R*)-6e** isolated 28.0 mg (0.08 mmol) 32% yield.

Major diastereomer (configuration 1*R*, 3*R*)-6e

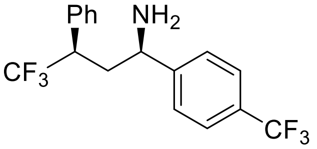

^{1}H NMR (400 MHz, $CDCl_3$) δ 7.61 (d, *J* = 8.0 Hz, 2H), 7.40 – 7.37 (m, 3H), 7.30 (d, *J* = 8.0 Hz, 2H), 7.23 – 7.21 (m, 2H), 3.75 (dd, *J* = 8.8, 6.1 Hz, 1H), 2.97 (pd, *J* = 9.4, 5.3 Hz, 1H), 2.42 – 2.30 (m, 2H), 1.54 (bs, 2H).

^{13}C NMR (100 MHz, $CDCl_3$) δ 148.4, 134.0 (Cq, *J* = 1.8 Hz), 130.0 (Cq, *J* = 32.4 Hz), 129.1, 128.9, 128.5, 127.0, 126.6, 125.8 (Cq, *J* = 3.7 Hz), 124.0 (d, *J* = 272.1 Hz), 53.3, 47.3 (d, *J* = 27.1 Hz), 37.9.

^{19}F NMR (376 MHz, $CDCl_3$) δ – 62.51 (s, CF_3), – 70.00 (d, *J* = 9.2 Hz, CF_3).

***tert*-Butyl (4,4,4-trifluoro-3-phenyl-1-(4-(trifluoromethyl)phenyl)butyl)carbamate (6e')**

The titled compound was obtained following GPB from **5e** and purified by FCC using (pentane:EtOAc 95:5). Major diastereomer **(1*R*,3*R*)-6e'**, isolated yield 78% (27.8 mg, 0.106 mmol) from 28.0 mg (0.08 mmol) of **6e**.

The enantiomeric excess (minor isomer: nd, major isomer 87%) was determined by HPLC (CHIRACEL® OD–H), hexane/iPrOH 98/2, 1 mL/min, λ = 210 nm, **1*R*,3*R*** (major diastereomer): minor enantiomer t_r = 10.8 min, major enantiomer, t_r = 7.8 min.

Major diastereomer (configuration 1*R*, 3*R*)-6e'

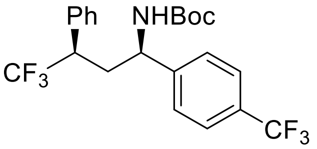

$[\alpha]_D^{25}$: – 16.0 (c 0.45, $CHCl_3$, *ee* 87%).

^{1}H NMR (400 MHz, $CDCl_3$) δ 7.62 (d, *J* = 8.0 Hz, 2H), 7.42 – 7.38 (m, 3H), 7.26 – 7.21 (m, 4H), 4.75 (d, *J* = 7.5 Hz, 1H), 4.46 (bs, 1H), 2.95 (dtd, *J* = 18.4, 9.2, 3.7 Hz, 1H), 2.56 (bs, 1H), 2.36 (ddd, *J* = 13.6, 9.7, 3.8 Hz, 1H), 1.36 (s, 9H).

^{13}C NMR (100 MHz, $CDCl_3$) δ 154.6, 144.8, 133.2, 130.3 (Cq, *J* = 32.6 Hz), 129.1, 129.0, 128.7, 127.1, 126.4 (d, *J* = 279.8 Hz), 125.9 (Cq, *J* = 3.6 Hz), 123.9 (d, *J* = 272.1 Hz), 80.0, 52.7, 47.2 (Cq, *J* = 27.8 Hz), 34.8, 28.3.

^{19}F NMR (376 MHz, $CDCl_3$) δ – 62.56 (s, CF3), – 70.23 (bs, CF_3).

HRMS (ESI) *m/z*: 470.1551 $[M+Na]^+$, $C_{22}H_{23}F_6NNaO_2^+$ requires 470.1525.

4,4,4-Trifluoro-1-(naphthalen-2-yl)-3-phenylbutan-1-amine (6f)

The titled compound was obtained following GPC from **4f** (81.8 mg, 0.25 mmol) as a colourless oil (86% NMR yield). The diastereomers were purified by FCC using (pentane:EtOAc 8:2 to 6:4). The

diastereomers were purified by FCC using (pentane:EtOAc 8:2 to 6:4). The diastereomers were obtained in a ratio 75:25 in 87% yield. Minor diastereomer **(1*S*,3*R*)-6f** isolated 23 mg (0.07 mmol) 28 % yiled. Major diastereomer **(1*R*,3*R*)-6f** isolated 46.0 mg (0.14 mmol) 56% yield.

Minor diastereomer (configuration 1*S*, 3*R*)-6f

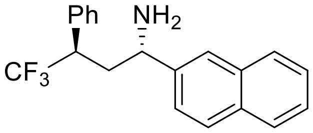

^{1}H NMR (400 MHz, $CDCl_3$) δ 7.83 – 7.78 (m, 3H), 7.64 (s, 1H), 7.50 – 7.46 (m, 2H), 7.41 – 7.37 (m, 5H), 7.33 (dd, J = 8.6, 1.8 Hz, 1H), 3.82 – 3.73 (m, 2H), 2.32 (dd, J = 8.2, 6.5 Hz, 2H), 1.49 (bs, 2H).

^{13}C NMR (100 MHz, $CDCl_3$) δ 143.7, 134.5 (Cq, J = 2.0 Hz), 133.4, 132.3, 129.2, 128.8, 128.5, 128.3, 127.7, 127.6, 126.2, 125.8, 124.2, 124.1, 52.6, 47.3 (Cq, J = 26.7 Hz), 38.2.

^{19}F NMR (376 MHz, $CDCl_3$) δ – 69.39 (d, J = 9.6 Hz, CF_3).

Major diastereomer (configuration 1*R*, 3*R*)-6f

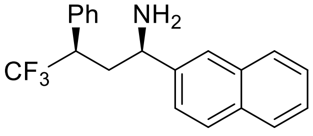

^{1}H NMR (400 MHz, $CDCl_3$) δ 7.89 – 7.79 (m, 3H), 7.53 – 7.48 (m, 3H), 7.41 – 7.38 (m, 4H), 7.26 – 7.23 (m, 2H), 3.81 (t, J = 7.5 Hz, 1H), 2.99 (pd, J = 9.3, 6.6 Hz, 1H), 2.45 (dd, J = 8.4, 6.7 Hz, 2H), 1.61 (bs, 2H).

^{13}C NMR (100 MHz, $CDCl_3$) δ 141.6, 134.3 (Cq, J = 1.9 Hz), 133.3, 133.0, 129.2, 128.9, 128.7, 128.3, 127.8, 127.7, 126.7 (Cq, J = 279.8 Hz), 126.3, 126.0, 125.8, 124.1, 53.7, 47.4 (Cq, J = 26.8 Hz), 37.7.

^{19}F NMR (376 MHz, $CDCl_3$) δ – 69.93 (d, J = 9.6 Hz, CF_3).

HRMS (ESI) *m/z*: 330.1482 $[M+H]^+$, $C_{20}H_{19}F_3N^+$ requires 330.1464.

***tert*-Butyl (4,4,4-trifluoro-1-(naphthalen-2-yl)-3-phenylbutyl)carbamate (6f')**

The titled compound was obtained following GPB from **5f** and purified by FCC using (pentane:EtOAc 95:5). Minor diastereomer **(1*S*,3*R*)-6f'**, isolated yield 25% (7.5 mg, 0.06 mmol) from 23.0 mg (0.08 mmol) of **5f**. Major diastereomer **(1*R*,3*R*)-6f'**, isolated yield 55% (33.1 mg, 0.08 mmol) from 46.0 mg (0.14 mmol) of **6f**.

The enantiomeric excess (minor isomer: 93%, major isomer 95%) was determined by HPLC (CHIRACEL® OD–H), hexane/iPrOH 98/2, 1 mL/min, λ = 210 nm, ***1S*,*3R*** (minor diastereomer): minor enantiomer, t_r = 21.5 min, major enantiomer, t_r = 16.7 min, ***1R*,*3R*** (major diastereomer): minor enantiomer t_r = 13.2 min, major enantiomer, t_r = 10.9 min.

Minor diastereomer (configuration 1*S*, 3*R*)-6f'

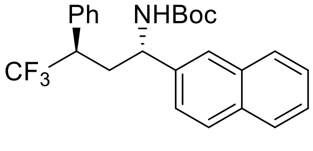

$[\alpha]_D^{25}$: – 21.0 (c 0.20, $CHCl_3$, *ee* 93%).

^{1}H NMR (400 MHz, $CDCl_3$) δ 7.82 – 7.77 (m, 3H), 7.60 (s, 1H), 7.49 – 7.46 (m, 2H), 7.38 – 7.34 (m, 5H), 7.26 – 7.25 (m, 1H), 5.00 – 4.85 (m, 1H), 4.66 – 4.43 (m, 1H), 3.51 (bs, 1H), 2.46 – 2.40 (m, 2H), 1.44 – 1.27 (m, 9H).

^{13}C NMR (100 MHz, $CDCl_3$) δ 155.0, 139.5, 134.0, 133.3, 132.7, 129.1, 128.9, 128.6, 128.5, 127.8, 127.6, 126.9 (Cq, *J* = 278.0 Hz), 126.3, 126.0, 125.5, 124.5, 124.2, 79.8, 51.7, 47.4 (Cq, *J* = 27.0 Hz), 36.6, 28.3.

^{19}F NMR (376 MHz, $CDCl_3$) δ – 69.25 (d, *J* = 9.6 Hz, CF_3).

Major diastereomer (configuration 1*R*, 3*R*)-6f'

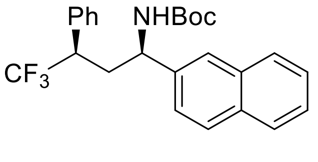

$[\alpha]_D^{25}$: – 43.5 (c 1.00, $CHCl_3$, *ee* 95%).

^{1}H NMR (400 MHz, $CDCl_3$) δ 7.89 – 7.84 (m, 2H), 7.80 – 7.78 (m, 1H), 7.52 – 7.50 (m, 3H), 7.40 – 7.39 (m, 3H), 7.28 (d, *J* = 8.4 Hz, 1H), 7.23 – 7.21 (m, 2H), 4.86 (d, *J* = 6.6 Hz, 1H), 4.54 (bs, 1H), 2.95 (ddt, *J* = 18.5, 12.7, 6.3 Hz, 1H), 2.68 (bs, 1H), 2.46 (ddd, *J* = 13.7, 10.2, 3.6 Hz, 1H), 1.37 (s, 9H).

^{13}C NMR (100 MHz, $CDCl_3$) δ 154.7, 137.6, 133.3, 133.2, 133.0, 129.3, 129.0, 128.8, 128.5, 128.0, 127.9, 127.7, 126.6 (Cq, *J* = 279.9 Hz), 126.4, 126.2, 124.1, 79.6, 53.1, 47.2 (Cq, *J* = 26.7 Hz), 35.5, 28.3.

^{19}F NMR (376 MHz, $CDCl_3$) δ – 70.16 (s, CF_3).

HRMS (ESI) *m/z*: 452.1826 $[M+Na]^+$, $C_{25}H_{26}F_3NNaO_2^+$ requires 452.1808.

<u>4,4,4-Trifluoro-1-(3-methoxyphenyl)-3-phenylbutan-1-amine (6g)</u>

The titled compound was obtained following GPC from **4g** (76.8 mg, 0.25 mmol) as a colourless oil (99% NMR yield). The diastereomers were purified by FCC using (pentane:EtOAc 8:2 to 6:4). The diastereomers were purified by FCC using (pentane:EtOAc 8:2 to 6:4). The diastereomers were obtained in a ratio 66:33 in 99% yield. Minor diastereomer **(1*S*,3*R*)-6g** isolated 24 mg (0.08 mmol) 31 % yiled. Major diastereomer **(1*R*,3*R*)-6g** isolated 48.0 mg (0.16 mmol) 62% yield.

Minor diastereomer (configuration 1*S*, 3*R*)-6g

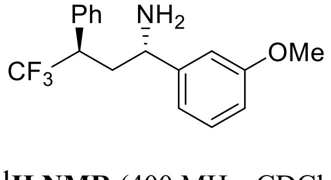

^{1}H NMR (400 MHz, $CDCl_3$) δ 7.41 – 7.33 (m, 5H), 7.23 (t, *J* = 7.9 Hz, 1H), 6.80 – 6.73 (m, 3H), 3.80 (s, 3H), 3.75 – 3.51 (m, 1H), 2.23 – 2.19 (m, 2H), 1.49 (bs, 2H).

^{13}C NMR (100 MHz, $CDCl_3$) δ 159.9, 148.2, 134.4 (Cq, *J* = 1.9 Hz), 129.7, 129.2, 128.8, 128.3, 127.1 (Cq, *J* = 279.6 Hz), 118.0, 112.2, 111.7, 55.2, 52.5, 47.3 (Cq, *J* = 26.7 Hz), 38.2.

^{19}**F NMR** (376 MHz, CDCl$_3$) δ – 69.47 (d, *J* = 9.6 Hz, CF$_3$).

Major diastereomer (configuration 1*R*, 3*R*)-6g

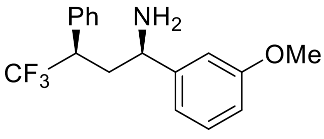

^{1}**H NMR** (400 MHz, CDCl$_3$) δ 7.40 – 7.36 (m, 3H), 7.29 – 7.23 (m, 3H), 6.84 (dd, *J* = 8.3, 2.5 Hz, 1H), 6.75 (d, *J* = 7.6 Hz, 1H), 6.71 (bs, 1H), 3.81 (s, 3H), 3.61 – 3.58 (m, 1H), 3.06 – 2.95 (m, 1H), 2.36 – 2.32 (m, 2H), 1.68 (bs, 2H).

^{13}**C NMR** (100 MHz, CDCl$_3$) δ 159.9, 146.0, 134.3 (Cq, *J* = 1.9 Hz), 129.9, 129.2, 128.7, 128.1, 126.7 (Cq, *J* = 279.7 Hz), 118.8, 113.1, 112.1, 55.2, 53.6, 47.3 (Cq, *J* = 26.8 Hz), 37.8.

^{19}**F NMR** (376 MHz, CDCl$_3$) δ – 69.92 (d, *J* = 9.6 Hz, CF$_3$).

***tert*-Butyl (4,4,4-trifluoro-1-(3-methoxyphenyl)-3-phenylbutyl)carbamate (6g')**

The titled compound was obtained following GPB from **6g** and purified by FCC using (pentane:EtOAc 95:5). Minor diastereomer **(1*S*,3*R*)-6g'**, isolated yield 60% (19.7 mg, 0.05 mmol) from 24.0 mg (0.08 mmol) of **6g**. Major diastereomer **(1*R*,3*R*)-6g'**, isolated yield 88% (57.7 mg, 0.14 mmol) from 48.0 mg (0.16 mmol) of **6g**.

The enantiomeric excess (minor isomer: 86%, major isomer 89%) was determined by HPLC (CHIRACEL® OD–H), hexane/iPrOH 98/2, 1 mL/min, λ = 210 nm, **1*R*,3*R*** (major diastereomer): minor enantiomer t_r = 9.5 min, major enantiomer, t_r = 7.7 min.

Minor diastereomer (configuration 1*S*, 3*R*)-6g'

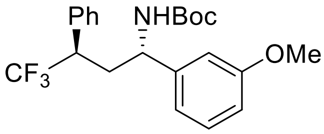

$[\alpha]_D^{25}$: – 43.5 (c 1.00, CHCl$_3$, *ee* 95%).

^{1}**H NMR** (400 MHz, CDCl$_3$) δ 7.41 – 7.37 (m, 3H), 7.31 – 7.30 (m, 2H), 7.22 (t, *J* = 7.9 Hz, 1H), 6.78 (dd, *J* = 8.3, 2.4 Hz, 1H), 6.74 (d, *J* = 7.7 Hz, 1H), 6.68 (s, 1H), 4.71 (d, *J* = 9.3 Hz, 1H), 4.41 (bs, 1H), 3.77 (s, 3H), 3.46 (bs, 1H), 2.37 – 2.27 (m, 2H), 1.43 (s, 9H).

^{13}**C NMR** (100 MHz, CDCl$_3$) δ 159.8, 154.9, 143.9, 134,0, 129.8, 129.1, 128.8, 128.5, 126.9 (Cq, *J* = 279.3 Hz), 118.1, 112.7, 112.0, 79.7, 55.2, 51.5, 47.3 (Cq, *J* = 27.0 Hz), 36.7, 28.3.

^{19}**F NMR** (376 MHz, CDCl$_3$) δ – 69.31 (d, *J* = 9.5 Hz, CF$_3$).

Major diastereomer (configuration 1*R*, 3*R*)-6g'

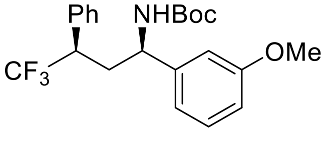

$[\alpha]_D^{25}$: – 0.8 (c 1.00, $CHCl_3$, *ee* 89%).

^{1}H NMR (400 MHz, $CDCl_3$) δ 7.41 – 7.36 (m, 3H), 7.29 – 7.23 (m, 3H), 6.85 (dd, *J* = 8.3, 2.4 Hz, 1H), 6.72 (d, *J* = 7.5 Hz, 1H), 6.63 (s, 1H), 4.74 (d, *J* = 7.3 Hz, 1H), 4.33 (bs, 1H), 3.78 (s, 3H), 2.97 (ddt, *J* = 18.7, 12.6, 6.3 Hz, 1H), 2.62 (bs, 1H), 2.33 (ddd, *J* = 13.7, 10.3, 3.5 Hz, 1H), 1.38 (s, 9H).

^{13}C NMR (100 MHz, $CDCl_3$) δ 159.9, 154.7, 141.9, 133.3, 130.1, 129.3, 128.8, 128.5, 126.6 (Cq, *J* = 279.8 Hz), 118.8, 113.5, 112.5, 79.6, 55.2, 52.9, 47.1 (Cq, *J* = 26.8 Hz), 35.6, 28.3.

^{19}F NMR (376 MHz, $CDCl_3$) δ – 70.19 (s, CF_3).

HRMS (ESI) *m/z*: 432.1761 $[M+Na]^+$, $C_{22}H_{26}F_3NNaO_3^+$ requires 432.1757.

4,4,4-Trifluoro-3-phenyl-1-(3-(trifluoromethyl)phenyl)butan-1-amine (6h)

The titled compound was obtained following GPC from **4h** (86.3 mg, 0.25 mmol) as a colourless oil (80% NMR yield). The diastereomers were purified by FCC using (pentane:EtOAc 8:2 to 6:4). The diastereomers were purified by FCC using (pentane:EtOAc 8:2 to 6:4). The diastereomers were obtained in a ratio 75:25 in 80% yield. Minor diastereomer **(1*S*,3*R*)-6h** isolated 15 mg (0.04 mmol) 17 % yiled. Major diastereomer **(1*R*,3*R*)-6h** isolated 45.0 mg (0.13 mmol) 52% yield.

Minor diastereomer (configuration 1*S*, 3*R*)-6h

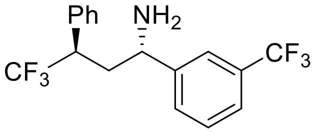

^{1}H NMR (400 MHz, $CDCl_3$) δ 7.49 (d, *J* = 7.6 Hz, 1H), 7.45 – 7.33 (m, 8H), 3.77 – 3.67 (m, 1H), 3.65 – 3.61 (m, 1H), 2.24 – 2.20 (m, 2H), 1.51 (bs, 2H).

^{13}C NMR (100 MHz, $CDCl_3$) δ 147.3, 134.0 (Cq, *J* = 1.9 Hz), 131.0 (Cq, *J* = 32.1 Hz), 129.3, 129.1, 128.9, 128.6, 128.4, 127.0 (Cq, *J* = 276.8 Hz), 126.7 (Cq, *J* = 279.6 Hz), 124.1 (Cq, *J* = 3.8 Hz), 122.6 (Cq, *J* = 3.8 Hz), 52.4, 47.3 (Cq, *J* = 26.9 Hz), 38.2.

^{19}F NMR (376 MHz, $CDCl_3$) δ – 62.59 (s, CF_3), – 69.54 (d, *J* = 9.6 Hz, CF_3).

HRMS (ESI) *m/z*: 348.1137 $[M+H]^+$, $C_{17}H_{16}F_6N^+$ requires 348.1181.

Major diastereomer (configuration 1*R*, 3*R*)-6h

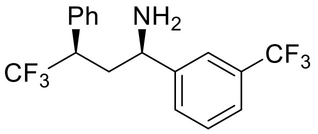

^{1}H NMR (400 MHz, $CDCl_3$) δ 7.56 (d, *J* = 7.6 Hz, 1H), 7.48 (t, *J* = 7.7 Hz, 1H), 7.42 – 7.36 (m, 5H), 7.22 – 7.20 (m, 2H), 3.76 (dd, *J* = 9.2, 5.6 Hz, 1H), 2.96 (pd, *J* = 9.4, 4.7 Hz, 1H), 2.42 – 2.29 (m, 2H), 1.56 (bs, 2H).

^{13}C NMR (100 MHz, $CDCl_3$) δ 145.4, 134.0 (Cq, *J* = 1.9 Hz), 131.0 (Cq, *J* = 32.2 Hz), 129.9, 129.3, 129.1, 128.9, 128.5, 126.6 (d, *J* = 279.8 Hz), 124.0 (Cq, *J* = 272.3 Hz), 124.6 (Cq, *J* = 3.8 Hz), 123.7 (Cq, *J* = 3.8 Hz), 53.4, 47.3 (Cq, *J* = 26.9 Hz), 38.0.

^{19}F NMR (376 MHz, $CDCl_3$) δ – 62.67 (s, CF_3), – 69.99 (d, J = 9.4 Hz, CF_3).

***tert*-Butyl (4,4,4-trifluoro-3-phenyl-1-(3-(trifluoromethyl)phenyl)butyl)carbamate (6h')**

The titled compound was obtained following GPB from **6h** and purified by FCC using (pentane:EtOAc 95:5). Minor diastereomer **(1*S*,3*R*)-6h'**, isolated yield 96% (17.1 mg, 0.04 mmol) from 15.0 mg (0.04 mmol) of **6h**. Major diastereomer **(1*R*,3*R*)-6h'**, isolated yield 93% (54.1 mg, 0.12 mmol) from 45.0 mg (0.13 mmol) of **6h**.

The enantiomeric excess (minor isomer: 77%, major isomer 90%) was determined by HPLC (CHIRACEL® OD–H), hexane/iPrOH 98/2, 1 mL/min, λ = 210 nm, **1*S*,3*R*** (minor diastereomer): minor enantiomer, t_r = 7.6 min, major enantiomer, t_r = 10.8 min, **1*R*,3*R*** (major diastereomer): minor enantiomer t_r = 16.5 min, major enantiomer, t_r = 10.3 min.

Minor diastereomer (configuration 1*S*, 3*R*)-6h'

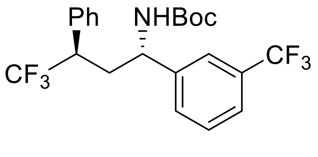

$[\alpha]_D^{25}$: – 9.9 (c 0.63, $CHCl_3$, *ee* 77%).

^{1}H NMR (400 MHz, $CDCl_3$) δ 7.50 (d, J = 7.7 Hz, 1H), 7.44 – 7.26 (m, 8H), 4.77 (bs, 1H), 4.51 (bs, 1H), 3.48 (bs, 1H), 2.37 – 2.27 (m, 2H), 1.43 – 1.26 (m, 9H).

^{13}C NMR (100 MHz, $CDCl_3$) δ 154.8, 143.4, 133.5, 131.1 (Cq, J = 27.6 Hz), 129.54, 129.47, 129.23, 129.16, 129.0, 128.7, 124.4 (Cq, J = 3.7 Hz), 122.6, 80.2, 51.3, 47.4 (Cq, J = 27.6 Hz), 36.5, 28.2.

^{19}F NMR (376 MHz, $CDCl_3$) δ – 62.66 (s, CF_3), – 69.38 (d, J = 9.5 Hz, CF_3).

Major diastereomer (configuration 1*R*, 3*R*)-6h'

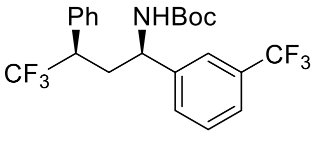

$[\alpha]_D^{25}$: – 7.8 (c 0.90, $CHCl_3$, *ee* 90%).

^{1}H NMR (400 MHz, $CDCl_3$) δ 7.58 (d, J = 7.7 Hz, 1H), 7.49 (t, J = 7.7 Hz, 1H), 7.40 – 7.33 (m, 5H), 7.21 – 7.20 (m, 2H), 4.76 (d, J = 7.4 Hz, 1H), 4.46 (bs, 1H), 2.99 – 2.88 (m, 1H), 2.56 (bs, 1H), 2.35 (ddd, J = 13.6, 9.6, 3.9 Hz, 1H), 1.36 (s, 9H).

^{13}C NMR (100 MHz, $CDCl_3$) δ 147.6, 141.8, 133.1, 131.18 (Cq, J = 32.3 Hz), 130.0, 129.5, 129.0, 128.7, 127.8, 126.44 (Cq, J = 279.9 Hz), 124.86 (Cq, J = 3.6 Hz), 123.9 (d, J = 272.5 Hz), 123.7, 80.0, 52.9, 47.2 (d, J = 25.8 Hz), 34.9, 28.2.

^{19}F NMR (376 MHz, $CDCl_3$) δ – 62.70 (s, CF_3), 70.22 (s, CF_3).

HRMS (ESI) *m/z*: 470.1531 $[M+Na]^+$, $C_{22}H_{23}F_6NNaO_2^+$ requires 470.1525.

4,4,4-Trifluoro-1-(2-methoxyphenyl)-3-phenylbutan-1-amine (6i)

The titled compound was obtained following GPC from **4i** (76.8 mg, 0.25 mmol) as a colourless oil (78% NMR yield). The diastereomers were purified by FCC using (pentane:EtOAc 8:2 to 6:4). The diastereomers were purified by FCC using (pentane:EtOAc 8:2 to 6:4). The diastereomers were obtained in a ratio 66:34 in 56% yield. Minor diastereomer **(1*S*,3*R*)-6i** isolated 18.6 mg (0.06 mmol) 24 % yiled. Major diastereomer **(1*R*,3*R*)-6i** isolated 20.2 mg (0.17 mmol) 26% yield.

Minor diastereomer (configuration 1*S*, 3*R*)-6i

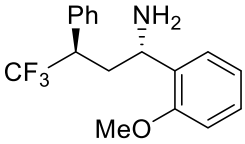

^{1}H NMR (400 MHz, $CDCl_3$) δ 7.40 – 7.33 (m, 5H), 7.20 (t, *J* = 7.8 Hz, 1H), 7.15 (d, *J* = 7.4 Hz, 1H), 6.91 (t, *J* = 7.4 Hz, 1H), 6.81 (d, *J* = 8.2 Hz, 1H), 3.85 – 3.73 (m, 5H), 2.32 – 2.19 (m, 2H), 1.59 (bs, 2H).

^{13}C NMR (100 MHz, $CDCl_3$) δ 156.6, 134.5 (Cq, *J* = 1.9 Hz), 134.4, 129.5, 128.4, 128.0, 127.9, 127.3 (Cq, *J* = 277.6 Hz), 126.1, 120.6, 110.6, 55.0, 48.6, 47.3 (q, *J* = 26.5 Hz), 35.8.

^{19}F NMR (376 MHz, $CDCl_3$) δ – 69.58 (d, *J* = 9.7 Hz, CF_3).

Major diastereomer (configuration 1*R*, 3*R*)-6i

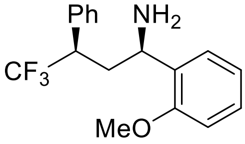

^{1}H NMR (400 MHz, $CDCl_3$) δ 7.36 – 7.31 (m, 3H), 7.27 – 7.19 (m, 3H), 6.97 – 6.88 (m, 3H), 3.81 – 3.74 (m, 4H), 3.02 (pd, *J* = 9.8, 3.7 Hz, 1H), 2.63 (ddd, *J* = 13.6, 9.8, 3.8 Hz, 1H), 2.32 (ddd, *J* = 13.5, 11.1, 5.5 Hz, 1H), 1.83 (m, 2H).

^{13}C NMR (100 MHz, $CDCl_3$) δ 157.3, 134.7 (Cq, *J* = 1.8 Hz), 131.9, 129.3, 128.5, 128.4, 128.1, 126.9 (Cq, *J* = 277.7 Hz), 120.7, 111.0, 55.1, 50.8, 47.8 (Cq, *J* = 26.6 Hz), 35.9.

^{19}F NMR (376 MHz, $CDCl_3$) δ – 69.84 (d, *J* = 9.4 Hz, CF_3).

***tert*-Butyl (4,4,4-trifluoro-1-(2-methoxyphenyl)-3-phenylbutyl)carbamate (6i')**

The titled compound was obtained following GPB from **6i** and purified by FCC using (pentane:EtOAc 95:5). Minor diastereomer **(1*S*,3*R*)-6i'**, isolated yield 84% (20.6 mg, 0.05 mmol) from 18.6 mg (0.06 mmol) of **6i**. Major diastereomer **(1*R*,3*R*)-6i'**, isolated yield 89% (25.5 mg, 0.06 mmol) from 20.2 mg (0.07 mmol) of **6i**.

The enantiomeric excess (minor isomer: 69%, major isomer 72%) was determined by HPLC (CHIRACEL® OD–H), hexane/iPrOH 98/2, 1 mL/min, λ = 210 nm, **1*S*,3*R*** (minor diastereomer): minor enantiomer, tr = 7.9 min, major enantiomer, tr = 7.3 min, **1*R*,3*R*** (major diastereomer): minor enantiomer tr = 9.6 min, major enantiomer, tr = 8.6 min.

Minor diastereomer (configuration 1*S*, 3*R*)-6i'

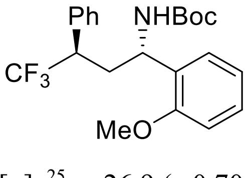

$[\alpha]_D^{25}$: – 26.9 (c 0.70, $CHCl_3$, *ee* 69%).

^{1}H NMR (400 MHz, $CDCl_3$) δ (*Rotamers are observed*) 7.40 – 7.28 (m, 5H), 7.20 (t, *J* = 7.6 Hz, 1H), 6.98 (d, *J* = 7.2 Hz, 1H), 6.87 – 6.80 (m, 2H), 5.33 (d, *J* = 10.0 Hz, 1H), 4.59 (td, *J* = 10.2, 3.7 Hz, 1H), 3.78, 3.52 – 3.40 (m, 1H), 2.48 – 2.42 (m, 1H), 2.30 – 2.23 (m, 1H), 1.43 – 1.26 (m, 9H).

^{13}C NMR (100 MHz, $CDCl_3$) δ 156.8. 155.0, 134.1, 129.8, 129.4, 128.6, 128.5, 128.2, 127.9, 120.7, 110.9, 79.3, 55.1, 47.5 (Cq, *J* = 28.2 Hz), 35.3, 28.4.

^{19}F NMR (376 MHz, $CDCl_3$) δ – 69.43 (d, *J* = 9.8 Hz, CF_3).

Major diastereomer (configuration 1*R*, 3*R*)-6i'

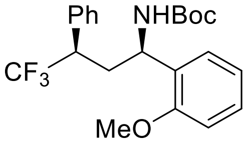

$[\alpha]_D^{25}$: +8.7 (c 0.50, $CHCl_3$, *ee* 72%).

^{1}H NMR (400 MHz, $CDCl_3$) (*Rotamers are observed*) δ 7.39 – 7.34 (m, 3H), 7.30 – 7.26 (m, 1H), 7.22 – 7.21 (m, 2H), 6.91 (d, *J* = 8.2 Hz, 1H), 6.86 (t, *J* = 7.4 Hz, 1H), 6.79 (d, *J* = 7.0 Hz, 1H), 5.42 (d, *J* = 8.7 Hz, 1H), 4.57 (q, *J* = 8.8 Hz, 1H), 3.86 (s, 3H), 2.95 – 2.86 (m, 1H), 2.65 – 2.59 (m, 1H), 2.60 – 2.42 (m, 1H), 1.39 (s, 9H).

^{13}C NMR (100 MHz, $CDCl_3$) δ 157.3, 154.9, 133.6, 130.0, 129.4, 129.1, 128.6, 128.3, 127.4, 126.8 (Cq, *J* = 279.9 Hz), 120.7, 111.1, 79.2, 55.3, 51.7, 47.6 (q, *J* = 27.0 Hz), 33.1, 28.4.

^{19}F NMR (376 MHz, $CDCl_3$) δ – 70.28 (d, *J* = 9.5 Hz, CF_3).

HRMS (ESI) *m/z*: 432.1736 $[M+Na]^+$, $C_{22}H_{26}F_3NNaO_3^+$ requires 432.1757.

5,5,5-Trifluoro-4-phenylpentan-2-amine (6j)

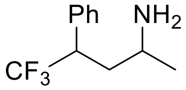

The final compound was not isolated due to low conversion. Conversion to the mixture of E and Z diastereomers was determined by integration of the CF_3 of both starting material and product by ^{19}F NMR (Shown below). ^{19}F NMR (376 MHz, $CDCl_3$) δ – 66.28 (s, CF_3, Allylic amine **4j**), – 69.55 - – 70.01 (d, CF_3, diastereomeric mixture of **6j**).

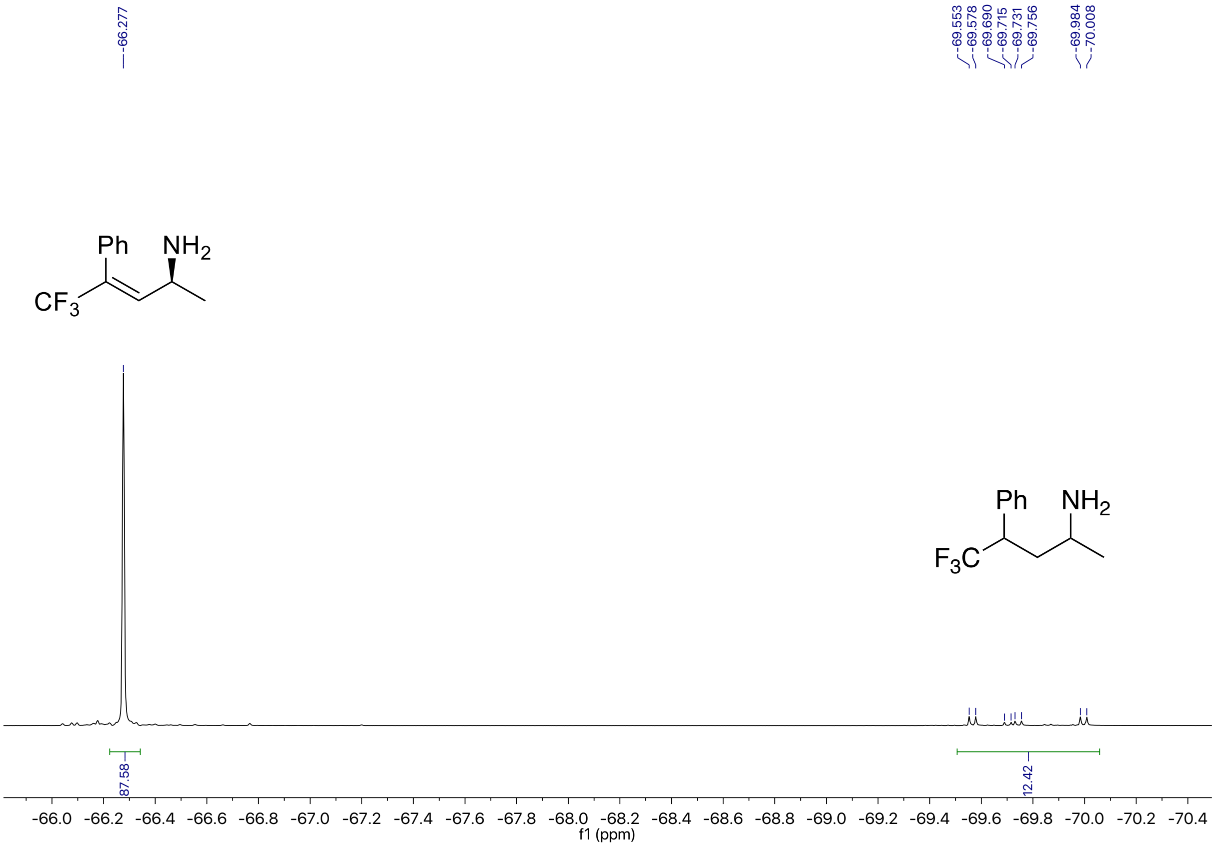

4,4,4-Trifluoro-3-phenylbutan-1-amine (6k)

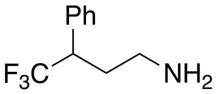

The final compound could not be separated from the starting material. The conversion to the mixture of E and Z diastereomers was determined by integration of the CF_3 of both starting material and product by ^{19}F NMR (Shown below). ^{19}F NMR (376 MHz, $CDCl_3$) δ – 66.00 - – 66.04 (s, CF_3, Allylic amine **4k**), – 69.63 - – 69.66 (s, CF_3, **6k**).

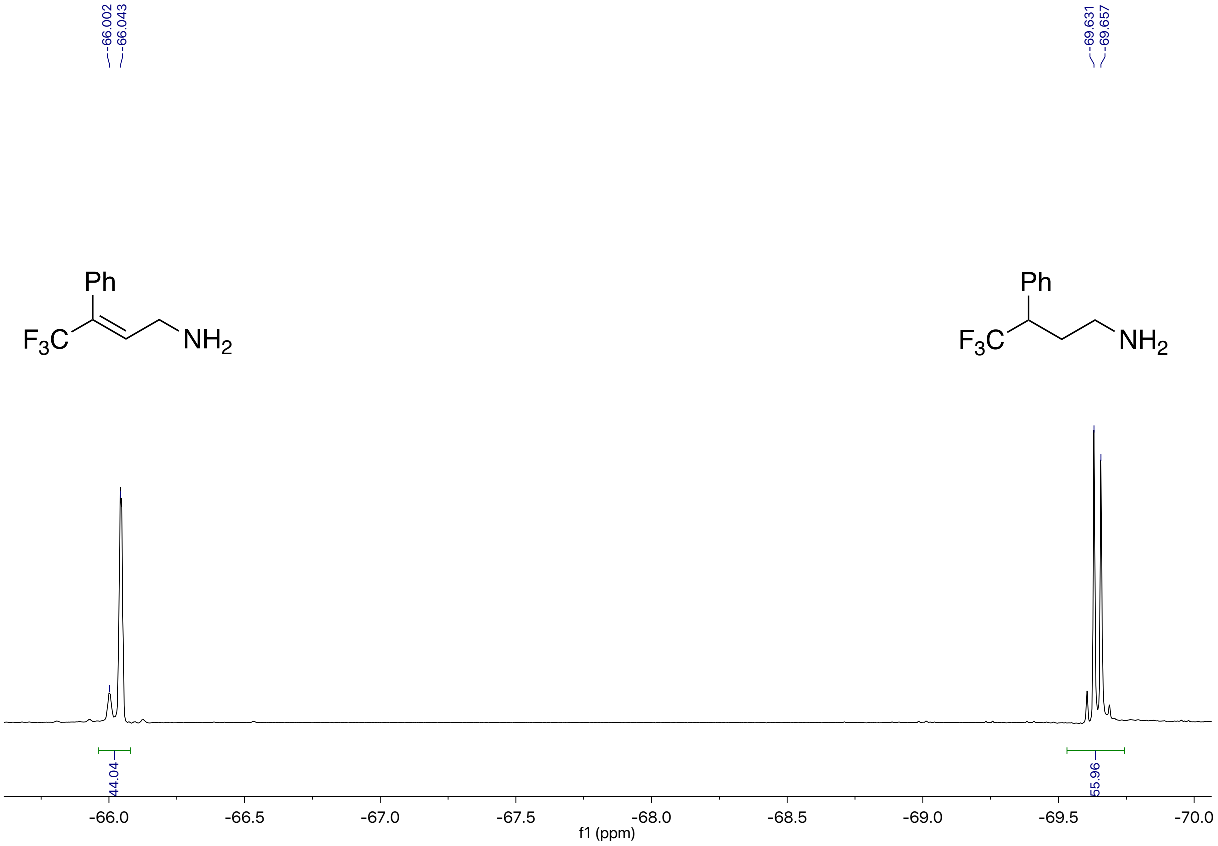

4,4,4-Trifluoro-1-phenyl-3-(*p*-tolyl)butan-1-amine (6l)

The titled compound was obtained following GPC from **4l** (72.8 mg, 0.25 mmol) as a colourless oil (90% NMR yield). The diastereomers were purified by FCC using (pentane:EtOAc 8:2 to 6:4). The diastereomers were obtained in a ratio 67:33 in 90% yield. Minor diastereomer could not be isolated. Major diastereomer **(1*R*,3*R*)-6l** isolated 44.0 mg (0.15 mmol) 60% yield.

Major diastereomer (configuration 1*R*, 3*R*)-6l

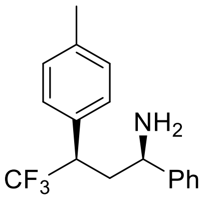

^{1}H NMR (400 MHz, $CDCl_3$) δ 7.37 – 7.27 (m, 3H), 7.19 – 7.17 (m, 4H), 7.11 (d, *J* = 7.9 Hz, 2H), 3.64 – 3.61 (m, 1H), 2.93 (dq, *J* = 18.5, 9.3 Hz, 1H), 2.37 (s, 3H), 2.35 – 2.31 (m, 2H), 1.55 (bs, 2H).

^{13}C NMR (100 MHz, $CDCl_3$) δ 144.5, 138.1, 131.22 (Cq, *J* = 1.9 Hz), 129.4, 129.0, 128.8, 127.7, 126.6, 126.8 (Cq, *J* = 279.7 Hz), 53.6, 47.0 (Cq, *J* = 26.8 Hz), 37.9, 21.1.

^{19}F NMR (376 MHz, $CDCl_3$) δ – 70.09 (d, *J* = 9.6 Hz, CF_3).

***tert*-Butyl (4,4,4-trifluoro-1-phenyl-3-(*p*-tolyl)butyl)carbamate (6l')**

The titled compound was obtained following GPB from **6l** and purified by FCC using (pentane:EtOAc 95:5). Major diastereomer **(1*R*,3*R*)-6l'**, isolated yield 87% (51.3 mg, 0.13 mmol) from 44.0 mg (0.15 mmol) of **6l**.

The enantiomeric excess (minor isomer: nd, major isomer 70%) was determined by HPLC (CHIRACEL® OD–H), hexane/iPrOH 98/2, 1 mL/min, λ = 210 nm, **1*R*,3*R*** (major diastereomer): minor enantiomer t_r = 6.9 min, major enantiomer, t_r = 5.1 min.

Major diastereomer (configuration 1*R*, 3*R*)-6l'

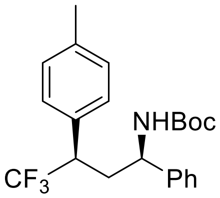

$[\alpha]_D^{25}$: – 15.8 (c 0.70, $CHCl_3$, *ee* 70%).

^{1}H NMR (400 MHz, $CDCl_3$) δ 7.37 – 7.29 (m, 3H), 7.19 (d, *J* = 7.8 Hz, 2H), 7.13 – 7.10 (m, 4H), 4.72 (d, *J* = 6.5 Hz, 1H), 4.35 (bs, 1H), 2.95 – 2.85 (m, 1H), 2.59 (bs, 1H), 2.37 – 2.29 (m, 4H), 1.38 (s, 9H).

^{13}C NMR (100 MHz, $CDCl_3$) δ 154.6, 140.4, 138.2, 130.2, 129.5, 129.1, 128.9, 128.0, 126.8, 126.7 (Cq, *J* = 279.7 Hz), 79.5, 52.9, 46.7 (Cq, *J* = 27.4 Hz), 34.7, 28.3, 21.1.

^{19}F NMR (376 MHz, $CDCl_3$) δ – 70.33 (d, *J* = 9.4 Hz, CF_3).

HRMS (ESI) *m/z*: 416.1871 $[M+Na]^+$, $C_{22}H_{26}F_3NNaO_2^+$ requires 416.1808.

4,4,4-Trifluoro-3-(4-methoxyphenyl)-1-phenylbutan-1-amine (6m)

The titled compound was obtained following GPC from **4m** (76.8 mg, 0.25 mmol) as a colourless oil (73% NMR yield). The diastereomers were purified by FCC using (pentane:EtOAc 8:2 to 6:4). The diastereomers were obtained in a ratio 65:35 in 73% yield. Minor diastereomer could not be isolated. Major diastereomer **(1*R*,3*R*)-6m** isolated 35.0 mg (0.11 mmol) 45% yield.

Major diastereomer (configuration 1*R*, 3*R*)-6m

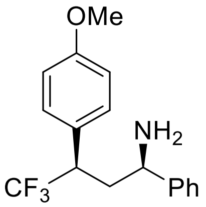

^{1}H NMR (400 MHz, $CDCl_3$) δ 7.38 – 7.33 (m, 2H), 7.31 – 7.27 (m, 1H), 7.18 – 7.13 (m, 4H), 6.90 (d, *J* = 8.8 Hz, 2H), 3.83 (s, 3H), 3.64 – 3.61 (m, 1H), 2.96 – 2.85 (m, 1H), 2.33 – 2.30 (m, 2H), 1.62 (bs, 2H).

^{13}C NMR (100 MHz, $CDCl_3$) δ 159.5, 144.4, 130.2, 128.8, 127.7, 126.8 (Cq, *J* = 278.0 Hz), 126.6, 126.2 (Cq, *J* = 2.2 Hz), 114.1, 55.2, 53.6, 46.6 (Cq, *J* = 26.9 Hz), 37.9.

^{19}F NMR (376 MHz, $CDCl_3$) δ – 70.34 (d, *J* = 9.4 Hz, CF_3).

***tert*-Butyl (4,4,4-trifluoro-3-(4-methoxyphenyl)-1-phenylbutyl)carbamate (6m')**

The titled compound was obtained following GPB from **6m** and purified by FCC using (pentane:EtOAc 95:5). Major diastereomer **(1*R*,3*R*)-6m'**, isolated yield 64% (28.8 mg, 0.07 mmol) from 35.0 mg (0.11 mmol) of **6m**.

The enantiomeric excess (minor isomer: nd, major isomer 81%) was determined by HPLC (CHIRACEL® OD–H), hexane/iPrOH 98/2, 1 mL/min, λ = 210 nm, **1*R*,3*R*** (major diastereomer): minor enantiomer t_r = 10.4 min, major enantiomer, t_r = 6.9 min.

Major diastereomer (configuration 1*R*, 3*R*)-6m'

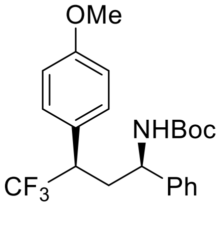

$[\alpha]_D^{25}$: – 22.9 (c 0.85, $CHCl_3$, *ee* 81%).

^{1}H NMR (400 MHz, $CDCl_3$) δ 7.37 – 7.29 (m, 3H), 7.15 – 7.11 (m, 4H), 6.91 (d, *J* = 8.6 Hz, 2H), 4.72 (d, *J* = 4.8 Hz, 1H), 4.34 (bs, 1H), 3.83 (s, 3H), 2.92 – 2.28 (m, 1H), 2.59 (bs, 1H), 2.31 (ddd, *J* = 13.6, 10.6, 3.4 Hz, 1H), 1.38 (s, 9H).

^{13}C NMR (100 MHz, $CDCl_3$) δ 159.6, 154.7, 140.3, 130.3, 129.0, 128.1, 126.9, 126.7 (Cq, *J* = 279.7 Hz), 125.1, 114.2, 79.5, 55.2, 52.9, 46.3 (Cq, *J* = 26.4 Hz), 34.6, 28.3.

^{19}F NMR (376 MHz, $CDCl_3$) δ – 70.58 (s, CF_3).

HRMS (ESI) *m/z*: 432.1764 $[M+Na]^+$, $C_{22}H_{26}F_3NNaO_3^+$ requires 432.1757.

4,4,4-Trifluoro-1-phenyl-3-(4-(trifluoromethyl)phenyl)butan-1-amine (6n)

The titled compound was obtained following GPC from **4n** (86.3 mg, 0.25 mmol) as a colourless oil (55% NMR yield). The diastereomers were purified by FCC using (pentane:EtOAc 8:2 to 6:4). The diastereomers were obtained in a ratio 85:15 in 55% yield. Minor diastereomer could not be isolated. Major diastereomer **(1*R*,3*R*)-6n** isolated 37.0 mg (0.11 mmol) 43% yield.

Major diastereomer (configuration 1*R*, 3*R*)-6n

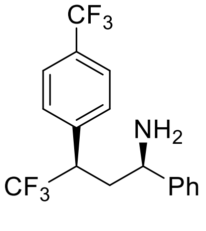

^{1}H NMR (400 MHz, $CDCl_3$) δ 7.64 (d, *J* = 8.0 Hz, 2H), 7.38 – 7.29 (m, 5H), 7.15 (d, *J* = 7.3 Hz, 2H), 3.60 (dd, *J* = 9.1, 6.0 Hz, 1H), 3.09 (pd, *J* = 9.4, 4.9 Hz, 1H), 2.43 – 2.31 (m, 2H), 1.56 (bs, 2H).

^{13}C NMR (100 MHz, $CDCl_3$) δ 148.4, 134.0, 130.0 (Cq, *J* = 32.4 Hz), 129.1, 128.9, 128.5, 127.0, 126.6 (Cq, J_{CF} = 279.8 Hz), 125.8 (Cq, J_{CF} = 3.7 Hz), 124.0 (Cq, J_{CF} = 272.0 Hz), 53.3, 47.3, 37.9.

^{19}F NMR (376 MHz, $CDCl_3$) δ – 62.71 (s, CF_3), – 69.73 (d, *J* = 9.1 Hz, CF_3).

***tert*-Butyl (4,4,4-trifluoro-1-phenyl-3-(4-(trifluoromethyl)phenyl)butyl)carbamate (6n')**

The titled compound was obtained following GPB from **6n** and purified by FCC using (pentane:EtOAc 95:5). Major diastereomer **(1*R*,3*R*)-6n'**, isolated yield 82% (40.4 mg, 0.09 mmol) from 37.0 mg (0.11 mmol) of **6n**.

The enantiomeric excess (minor isomer: nd, major isomer 70%) was determined by HPLC (CHIRACEL® OD–H), hexane/iPrOH 98/2, 1 mL/min, λ = 210 nm, **1*R*,3*R*** (major diastereomer): minor enantiomer t_r = 9.0 min, major enantiomer, t_r = 6.1 min.

Major diastereomer (configuration 1*R*, 3*R*)-6n'

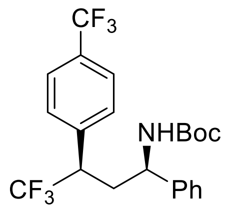

$[\alpha]_D^{25}$: – 1.8 (c 0.55, $CHCl_3$, *ee* 70%).

^{1}H NMR (400 MHz, $CDCl_3$) δ 7.66 (d, *J* = 8.1 Hz, 2H), 7.39 – 7.33 (m, 5H), 7.11 (dd, *J* = 7.8, 1.7 Hz, 2H), 4.68 (d, *J* = 7.0 Hz, 1H), 4.31 (bs, 1H), 3.02 (ddq, *J* = 18.2, 9.1, 4.5, 3.4 Hz, 1H), 2.69 (bs, 1H), 2.39 (ddd, *J* = 13.7, 10.3, 3.5 Hz, 1H), 1.38 (s, 9H).

^{13}C NMR (100 MHz, $CDCl_3$) δ 154.7, 139.8, 137.4, 130.8 (Cq, *J* = 32.6 Hz), 129.8, 129.2, 128.4, 126.8, 126.2 (Cq, *J* = 278.0 Hz), 125.8 (Cq, *J* = 3.7 Hz), 123.9 (Cq, *J* = 272.2 Hz), 79.8, 53.0, 47.1 (Cq, *J* = 27.2 Hz), 34.2, 28.3.

^{19}F NMR (376 MHz, $CDCl_3$) δ – 62.73 (s, CF_3), – 70.09 (d, *J* = 8.7 Hz, CF_3).

HRMS (ESI) *m/z*: 470.1548 $[M+Na]^+$, $C_{22}H_{23}F_6NNaO_2^+$ requires 470.1525.

4,4,4-Trifluoro-1-phenyl-3-(*m*-tolyl)butan-1-amine (6o)

The titled compound was obtained following GPC from **4o** (72.8 mg, 0.25 mmol) as a colourless oil (81% NMR yield). The diastereomers were purified by FCC using (pentane:EtOAc 8:2 to 6:4). The diastereomers were obtained in a ratio 75:25 in 75% yield. Minor diastereomer could not be isolated. Major diastereomer **(1*R*,3*R*)-6o** isolated 39.0 mg (0.13 mmol) 53% yield.

Major diastereomer (configuration 1*R*, 3*R*)-6o

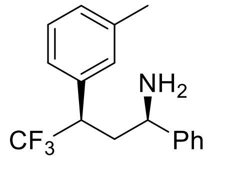

^{1}H NMR (400 MHz, $CDCl_3$) δ 7.38 – 7.34 (m, 2H), 7.31 – 7.29 (m, 1H), 7.28 – 7.24 (m, 1H), 7.19 – 7.16 (m, 3H), 7.04 – 7.00 (m, 2H), 3.63 (dd, *J* = 8.2, 6.8 Hz, 1H), 2.95 (dq, *J* = 18.6, 9.4 Hz, 1H), 2.37 (s, 3H), 2.35 – 2.32 (m, 2H), 1.62 (bs, 2H).

^{13}C NMR (100 MHz, $CDCl_3$) δ 144.5, 138.4, 134.3 (Cq, *J* = 1.9 Hz), 129.7, 129.0, 128.8, 128.5, 128.2, 127.7, 126.8 (Cq, *J* = 278.0 Hz), 126.6, 126.4, 53.6, 47.3 (Cq, *J* = 26.7 Hz), 38.0, 21.4.

^{19}F NMR (376 MHz, $CDCl_3$) δ – 69.83 (d, *J* = 9.5 Hz, CF_3).

***tert*-Butyl (4,4,4-trifluoro-1-phenyl-3-(*m*-tolyl)butyl)carbamate (6o')**

The titled compound was obtained following GPB from **6o** and purified by FCC using (pentane:EtOAc 95:5). Major diastereomer **(1*R*,3*R*)-6o'**, isolated yield 71% (36.3 mg, 0.09 mmol) from 39.0 mg (0.11 mmol) of **6o**.

The enantiomeric excess (minor isomer: nd, major isomer 83%) was determined by HPLC (CHIRACEL® OD–H), hexane/iPrOH 98/2, 1 mL/min, λ = 210 nm, **1*R*,3*R*** (major diastereomer): minor enantiomer t_r = 6.8 min, major enantiomer, t_r = 5.2 min.

Major diastereomer (configuration 1*R*, 3*R*)-6o'

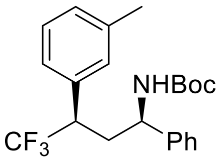

$[\alpha]_D^{25}$: – 1.1 (c 0.90, $CHCl_3$, *ee* 70%).

^{1}H NMR (400 MHz, $CDCl_3$) δ 7.40 – 7.31 (m, 3H), 7.27 – 7.25 (m, 1H), 7.18 – 7.13 (m, 3H), 7.03 (d, *J* = 7.7 Hz, 1H), 6.99 (s, 1H), 4.70 (bs, 1H), 4.38 (bs, 1H), 2.98 – 2.88 (m, 1H), 2.55 (bs, 1H), 2.37 – 2.31 (m, 4H), 1.37 (s, 9H).

^{13}C NMR (100 MHz, $CDCl_3$) δ 154.7, 140.6, 138.4, 133.4, 130.0, 129.2, 128.7, 128.0, 126.8, 126.2, 79.5, 53.0, 47.0, 35.0, 28.3, 21.4.

^{19}F NMR (376 MHz, $CDCl_3$) δ – 70.06 (s, CF_3).

HRMS (ESI) *m/z*: 416.1803 $[M+Na]^+$, $C_{22}H_{26}F_3NNaO_2^+$ requires 416.1808.

4,4,4-Trifluoro-1-phenyl-3-(thiophen-2-yl)butan-1-amine (6p)

The titled compound was obtained following GPC from **4p** (72.8 mg, 0.25 mmol) as a colourless oil (99% NMR yield). The diastereomers were purified by FCC using (pentane:EtOAc 8:2 to 6:4). The diastereomers were obtained in a ratio 73:27 in 99% yield. Minor diastereomer **(1*S*,3*S*)-6p** isolated 19.0 mg (0.07 mmol) 27% yield. Major diastereomer **(1*R*,3*S*)-6p** isolated 46.0 mg (0.16 mmol) 64% yield.

Minor diastereomer (configuration 1*S*, 3*S*)-6p

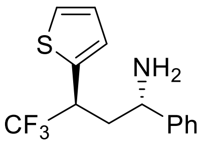

^{1}H NMR (400 MHz, $CDCl_3$) δ 7.35 – 7.31 (m, 3H), 7.26 – 7.22 (m, 3H), 7.09 (d, *J* = 2.9 Hz, 1H), 7.05 (dd, *J* = 5.1, 3.5 Hz, 1H), 4.12 (dqd, *J* = 10.8, 9.2, 4.1 Hz, 1H), 3.67 (dd, *J* = 10.2, 4.1 Hz, 1H), 2.24 – 2.11 (m, 2H), 1.51 (bs, 2H).

^{13}C NMR (100 MHz, $CDCl_3$) δ 146.3, 136.34 (Cq, *J* = 2.1 Hz), 128.7, 127.9, 127.2, 127.0, 126.3 (Cq, *J* = 279.5 Hz), 125.7, 125.6, 52.4, 42.8 (Cq, *J* = 28.4 Hz), 39.7.

^{19}F NMR (376 MHz, $CDCl_3$) δ – 70.67 (d, *J* = 8.9 Hz, CF_3).

Major diastereomer (configuration 1*R*, 3*S*)-6p

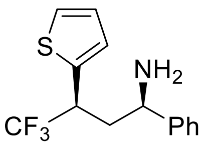

^{1}H NMR (400 MHz, $CDCl_3$) δ 7.39 – 7.29 (m, 4H), 7.24 – 7.22 (m, 2H), 7.03 (dd, *J* = 5.1, 3.5 Hz, 1H), 6.96 (d, *J* = 3.3 Hz, 1H), 3.75 (dd, *J* = 10.0, 5.1 Hz, 1H), 3.28 (dtt, *J* = 17.8, 8.9, 4.5 Hz, 1H), 2.40 – 2.25 (m, 2H), 1.58 (bs, 2H).

^{13}C NMR (100 MHz, $CDCl_3$) δ 144.1, 136.4 (q, *J* = 2.0 Hz), 128.9, 127.8, 127.7, 127.3, 126.9, 125.9 (Cq, *J* = 279.7 Hz), 125.7, 53.6, 42.9 (q, *J* = 28.5 Hz), 39.3.

^{19}F NMR (376 MHz, $CDCl_3$) δ – 71.09 (d, *J* = 8.9 Hz, CF_3).

***tert*-Butyl (4,4,4-trifluoro-1-phenyl-3-(thiophen-2-yl)butyl)carbamate (6p')**

The titled compound was obtained following GPB from **6p** and purified by FCC using (pentane:EtOAc 95:5). Minor diastereomer **(1*S*,3*R*)-6p'**, isolated yield 99% (27.0 mg, 0.07 mmol) from 19.0 mg (0.07 mmol) of **6p**. Major diastereomer **(1*R*,3S)-6p'**, isolated yield 65% (40.1 mg, 0.10 mmol) from 46.0 mg (0.16 mmol) of **6p**.

The enantiomeric excess (minor isomer: 86%, major isomer 90%) was determined by HPLC (CHIRACEL® OD–H), hexane/iPrOH 98/2, 1 mL/min, λ = 210 nm, **1*S*,3*S*** (minor diastereomer): minor enantiomer, t_r = 6.1 min, major enantiomer, t_r = 7.5 min, **1*R*,3*S*** (major diastereomer): minor enantiomer t_r = 8.3 min, major enantiomer, t_r = 6.1 min.

Minor diastereomer (configuration 1*S*, 3*S*)-6p'

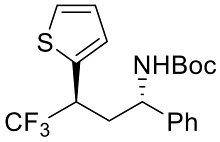

$[\alpha]_D^{25}$: – 37.0 (c 0.30, $CHCl_3$, *ee* 86%).

^{1}H NMR (400 MHz, $CDCl_3$) δ 7.34 – 7.30 (m, 3H), 7.27 – 7.24 (m, 1H), 7.19 (d, *J* = 7.3 Hz, 1H), 7.06 – 7.04 (m, 2H), 4.77 (d, *J* = 9.4 Hz, 1H), 4.60 (bs, 1H), 3.83 (bs, 1H), 2.35 (t, *J* = 11.2 Hz, 1H), 2.25 (t, *J* = 11.5 Hz, 1H), 1.43 (s, 9H).

^{13}C NMR (100 MHz, $CDCl_3$) δ 155.0, 142.1, 135.6 (Cq, *J* = 2.0 Hz), 128.8, 128.5, 127.5, 127.0, 126.0, 125.8, 79.8, 51.5, 42.9 (Cq, *J* = 28.3 Hz), 38.2, 28.3.

^{19}F NMR (376 MHz, $CDCl_3$) δ – 70.63 (d, *J* = 9.0, CF_3).

Major diastereomer (configuration 1*R*, 3*S*)-6p'

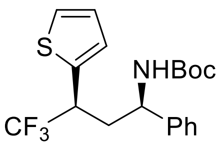

$[\alpha]_D^{25}$: +5.3 (c 0.94, $CHCl_3$, *ee* 90%).

^{1}H NMR (400 MHz, $CDCl_3$) δ 7.39 – 7.30 (m, 4H), 7.18 (d, *J* = 7.2 Hz, 2H), 7.05 – 7.00 (m, 2H), 4.75 (d, *J* = 4.8 H, 1H), 4.49 (bs, 1H), 3.24 (tt, *J* = 11.9, 8.8 Hz, 1H), 2.55 (bs, 1H), 2.37 (ddd, *J* = 13.5, 10.3, 3.3 Hz, 1H), 1.38 (s, 9H).

^{13}C NMR (100 MHz, $CDCl_3$) δ 154.6, 140.2, 135.2, 129.1, 128.2, 127.9, 127.2, 127.0, 126.8, 126.0, 125.8 (Cq, *J* = 279.8 Hz), 79.7, 53.0, 42.6 (Cq, *J* = 28.7 Hz), 36.0, 28.3.

^{19}F NMR (376 MHz, $CDCl_3$) δ – 71.25 (d, *J* = 8.9 Hz, CF_3).

HRMS (ESI) *m/z*: 408.1197 $[M+Na]^+$, $C_{19}H_{22}F_3NNaO_2S^+$requires 408.1216.

4,4,4-Trifluoro-1-phenylbutan-1-amine (6q)

The titled compound was obtained following GPC from **4q** (50.3 mg, 0.25 mmol) as a colourless oil (99% NMR yield). The diastereomers were purified by FCC using (pentane:EtOAc 8:2 to 6:4).

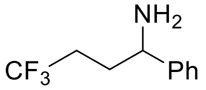

Isolated yield 86% (40.1 mg, 0.22 mmol).

^{1}H NMR (400 MHz, $CDCl_3$) δ 7.38 – 7.34 (m, 2H), 7.29 – 7.25 (m, 3H), 3.94 (t, *J* = 6.8 Hz, 1H), 2.19 – 1.87 (m, 4H), 1.50 (bs. 2H).

^{13}C NMR (100 MHz, $CDCl_3$) δ 145.1, 128.8, 127.4, 127.2 (Cq, *J* = 274.3 Hz), 126.1, 55.1, 31.4 (Cq, *J* = 2.5 Hz), 30.86 (Cq, *J* = 28.7 Hz).

^{19}F NMR (376 MHz, $CDCl_3$) δ – 66.19 (t, *J* = 10.7 Hz, CF_3).

HRMS (ESI) *m/z*: 187.0735 $[M+H]^+$, $C_{10}H_{10}F_3^+$ requires 187.0729.

NMR of allylic amines 4 and 4’

(*R*,*E*)-4,4,4-Trifluoro-1,3-diphenylbut-2-en-1-amine (4a)

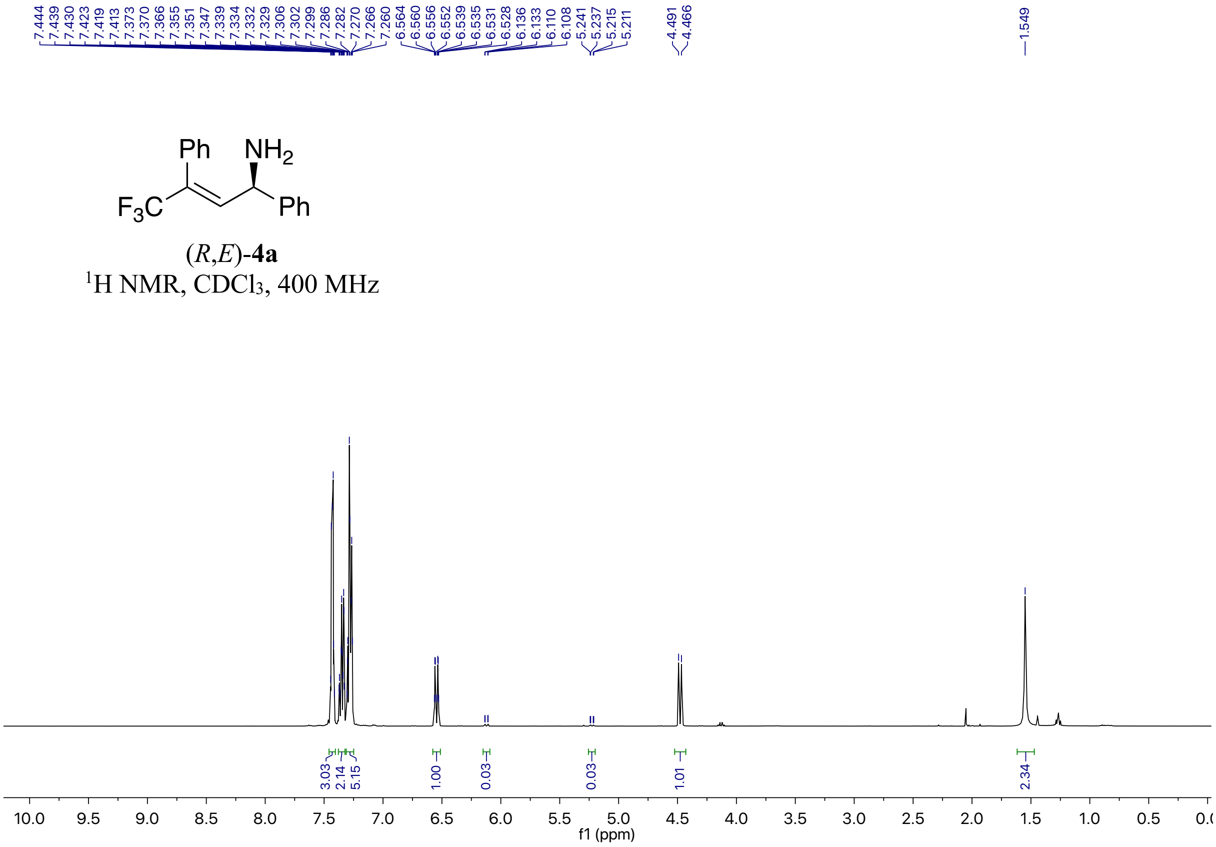

(*R*,*E*)-**4a**
^{1}H NMR, $CDCl_3$, 400 MHz

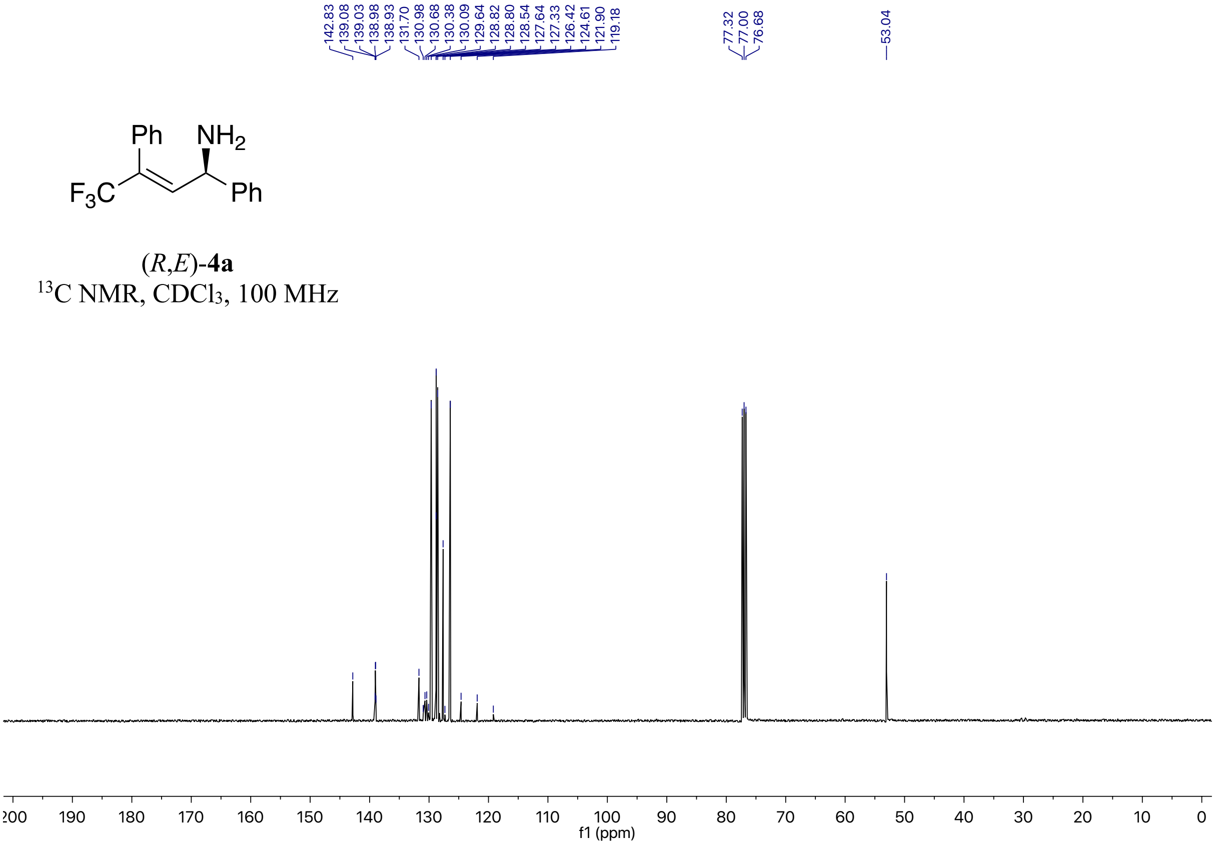

(*R*,*E*)-**4a**
^{13}C NMR, $CDCl_3$, 100 MHz

tert-Butyl (R,E)-(4,4,4-trifluoro-1,3-diphenylbut-2-en-1-yl)carbamate (4a')

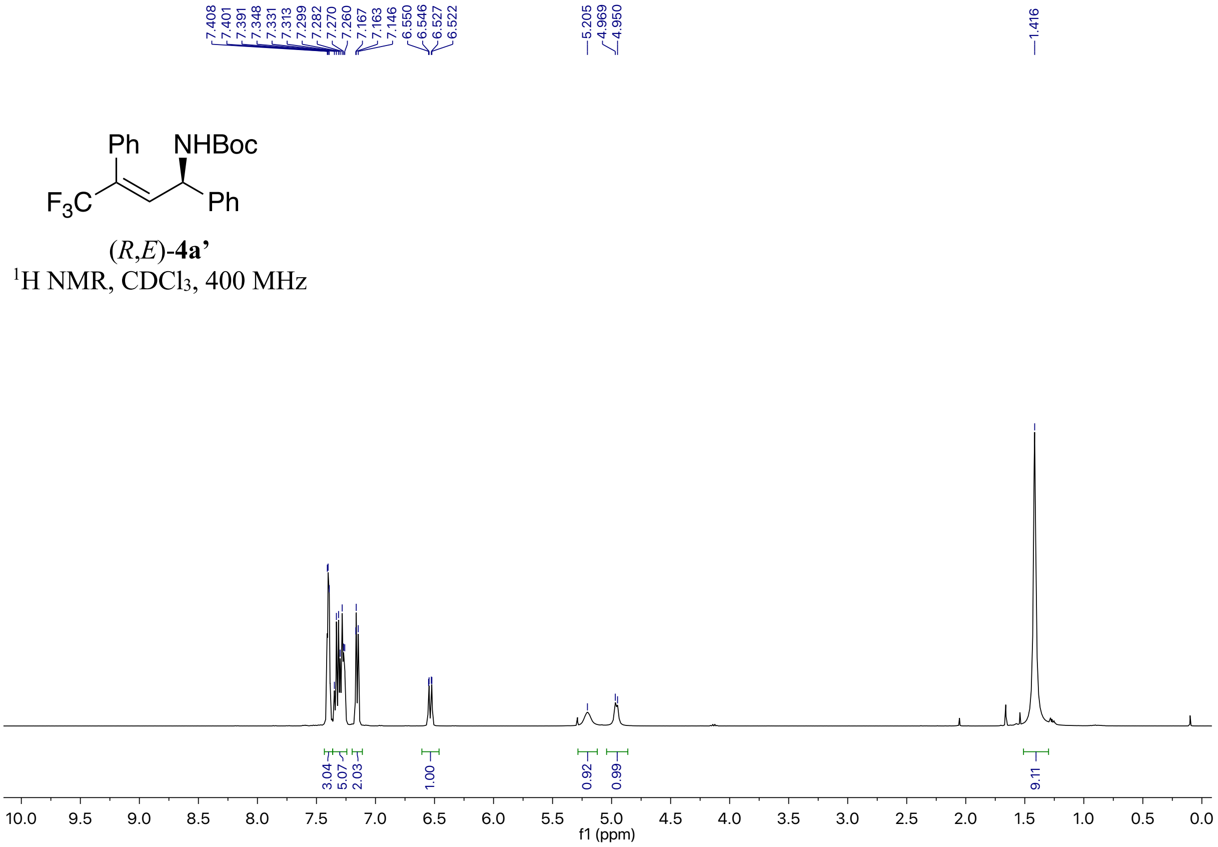

(*R*,*E*)-**4a'**
^{1}H NMR, $CDCl_3$, 400 MHz

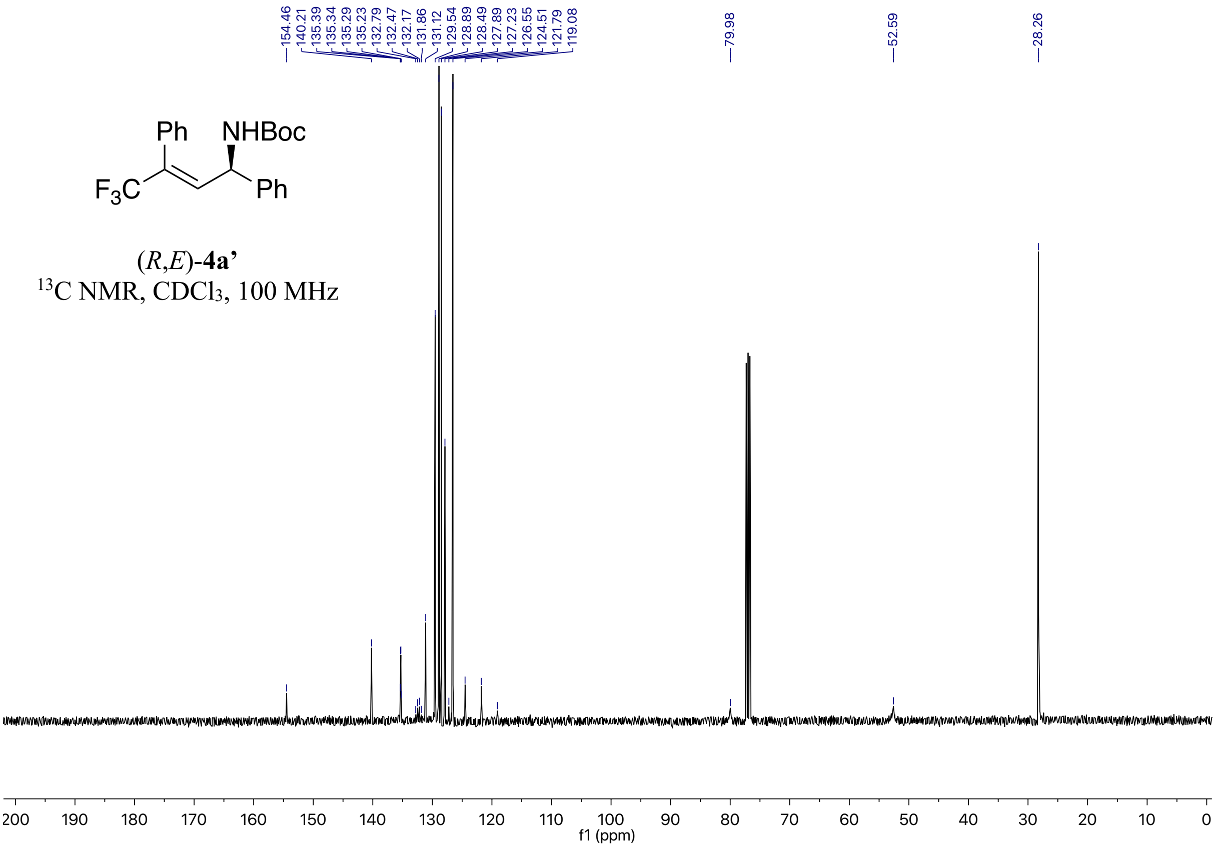

(*R*,*E*)-**4a'**
^{13}C NMR, $CDCl_3$, 100 MHz

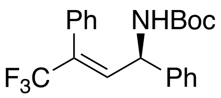

(*R*,*E*)-4a'

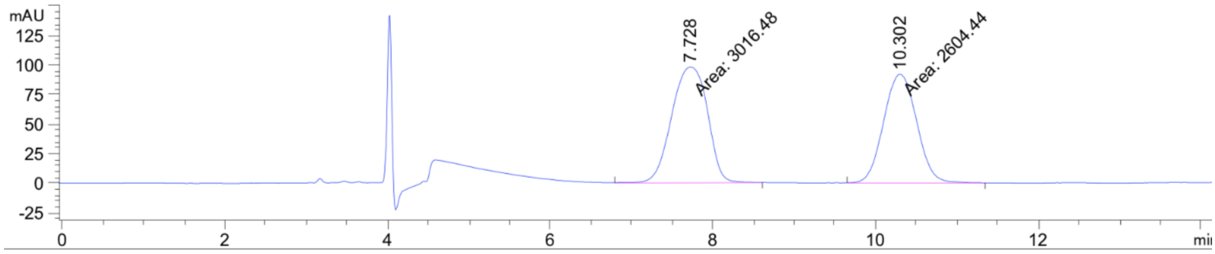

Peak #	RetTime [min]	Type	Width [min]	Area [mAU*s]	Height [mAU]	Area %
1	7.728	MM	0.5120	3016.48022	98.18989	53.6652
2	10.302	MM	0.4714	2604.44067	92.07738	46.3348

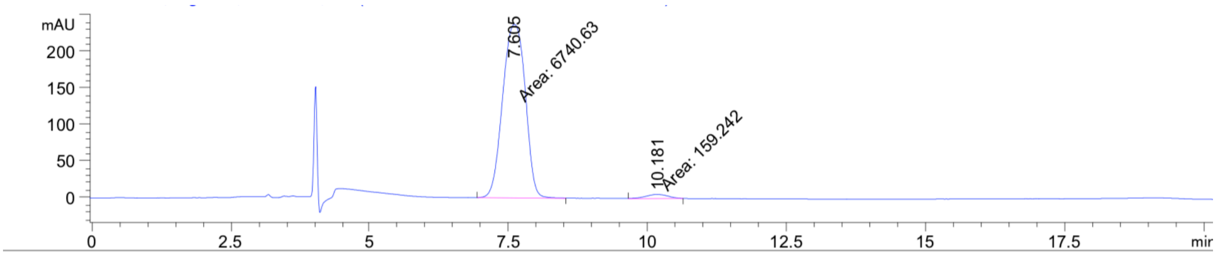

Peak #	RetTime [min]	Type	Width [min]	Area [mAU*s]	Height [mAU]	Area %
1	7.605	MM	0.4723	6740.62793	237.87270	97.6921
2	10.181	MM	0.4703	159.24196	5.64284	2.3079

(*R*,*E*)-4,4,4-Trifluoro-3-phenyl-1-(*p*-tolyl)but-2-en-1-amine (4b)

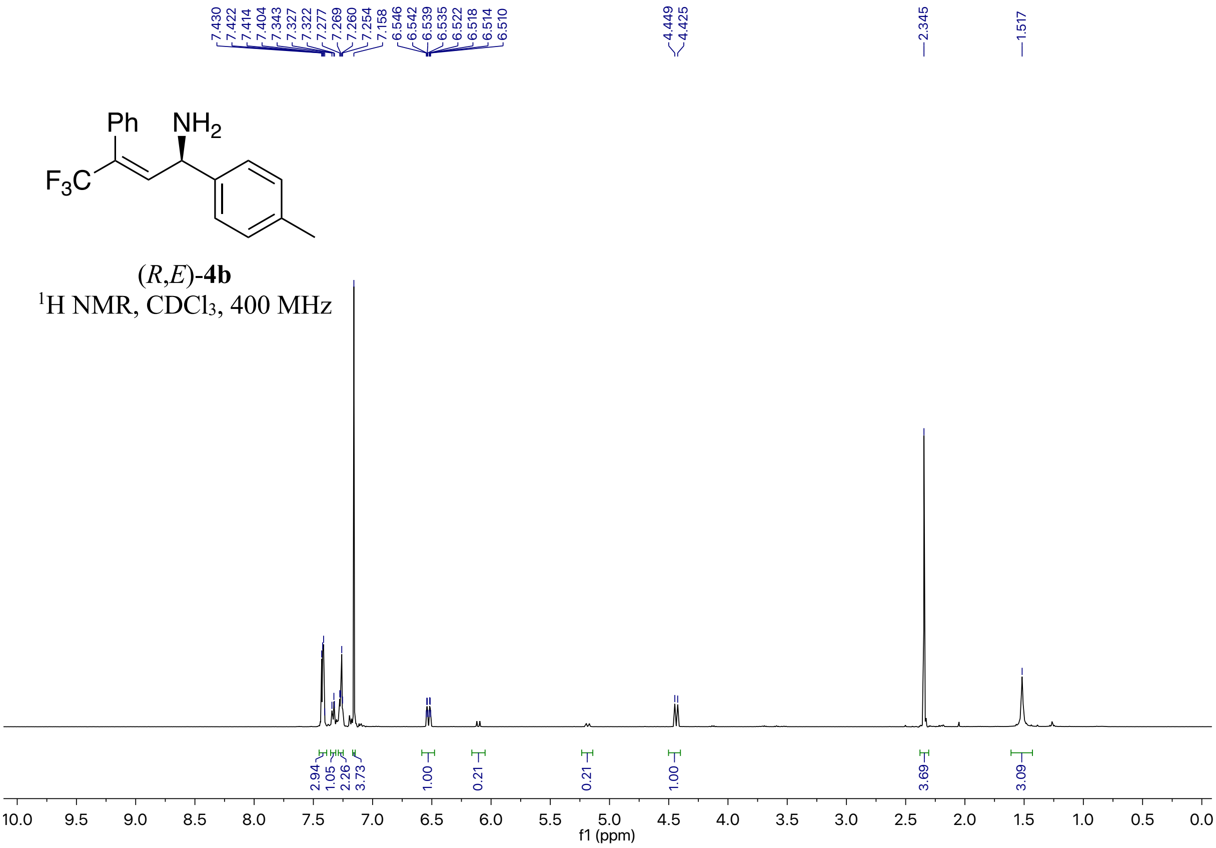

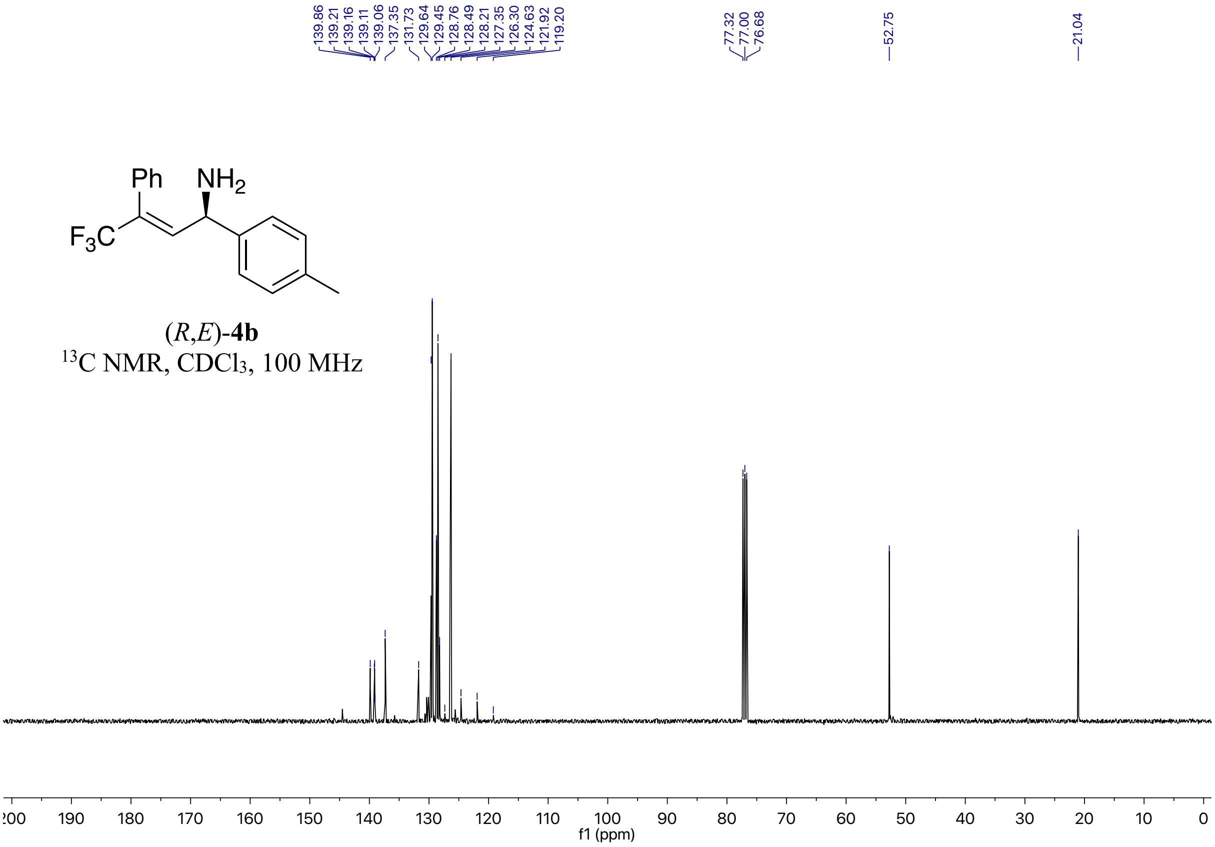

***tert*-Butyl (*R*,*E*)-(4,4,4-trifluoro-3-phenyl-1-(*p*-tolyl)but-2-en-1-yl)carbamate (4b')**

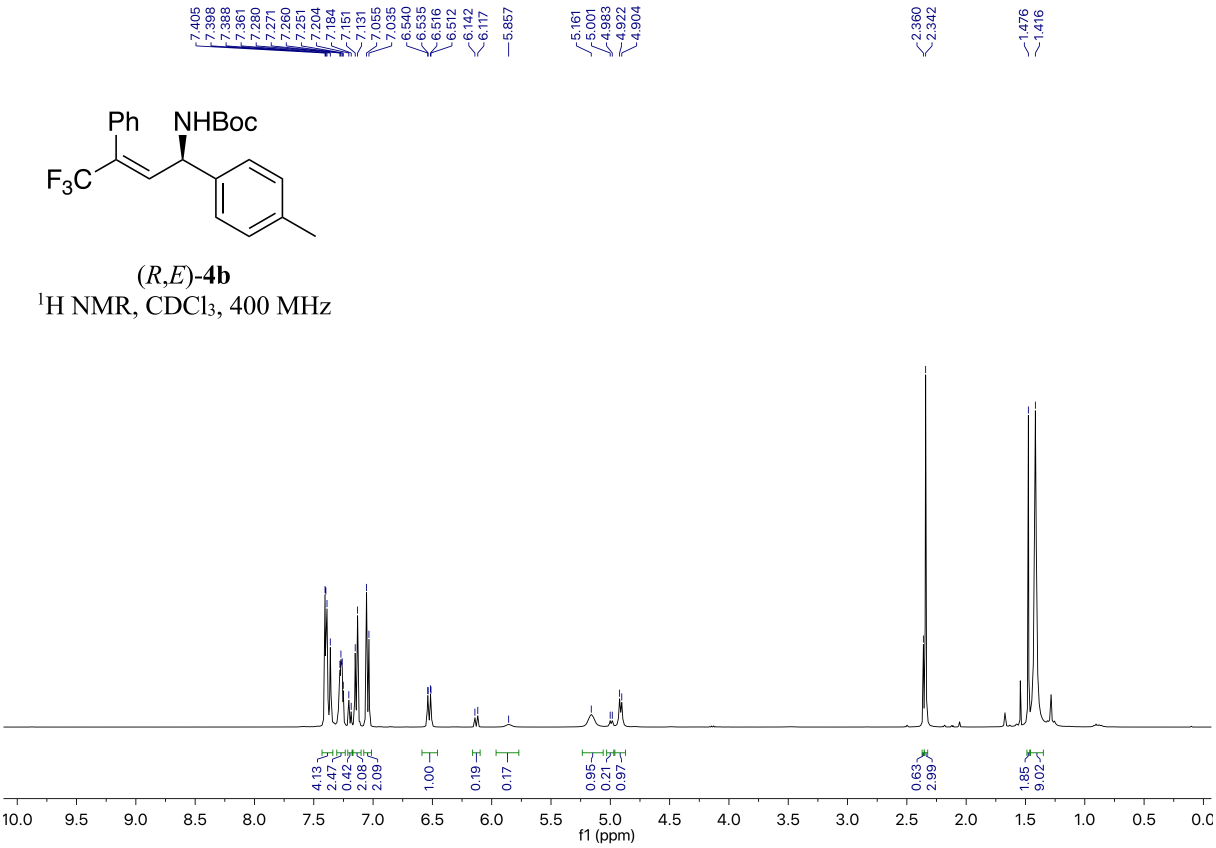

(*R*,*E*)-**4b**
^{1}H NMR, $CDCl_3$, 400 MHz

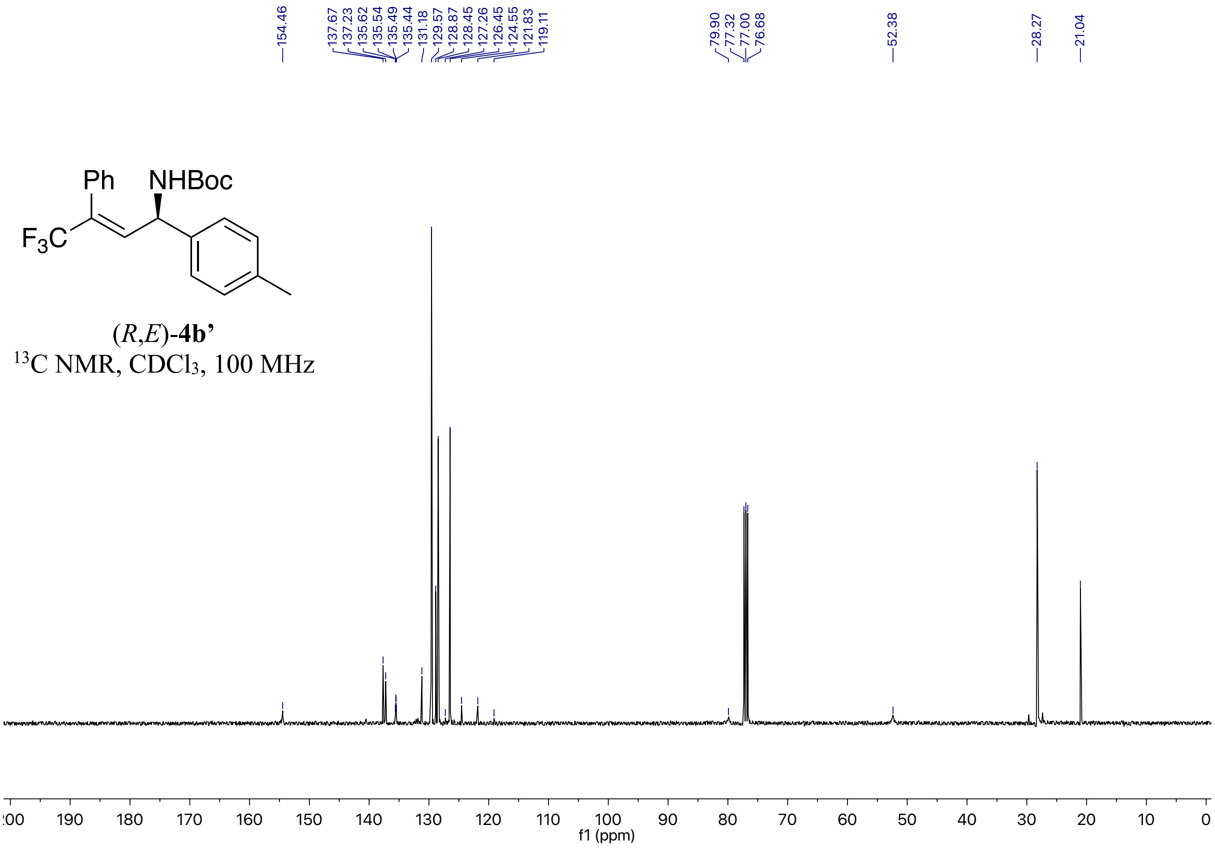

(*R*,*E*)-**4b'**
^{13}C NMR, $CDCl_3$, 100 MHz

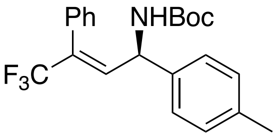

(*R*,*E*)-4b'

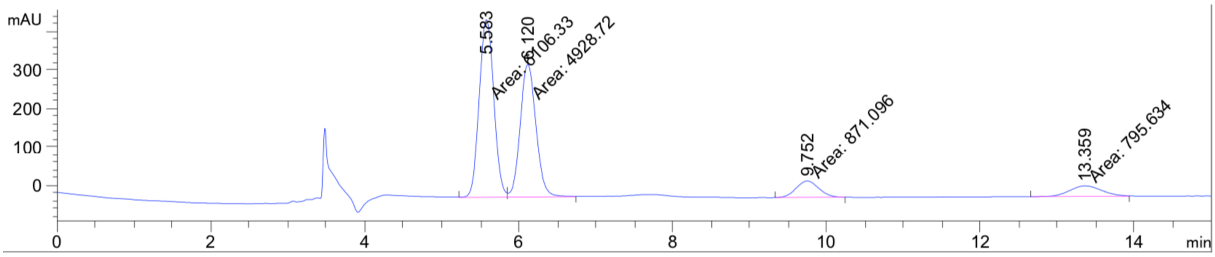

Peak #	RetTime [min]	Type	Width [min]	Area [mAU*s]	Height [mAU]	Area %
1	5.583	MF	0.2203	6106.32861	461.86719	48.0746
2	6.120	FM	0.2382	4928.72266	344.89444	38.8034
3	9.752	MM	0.3417	871.09570	42.49142	6.8581
4	13.359	MM	0.4886	795.63434	27.13961	6.2640

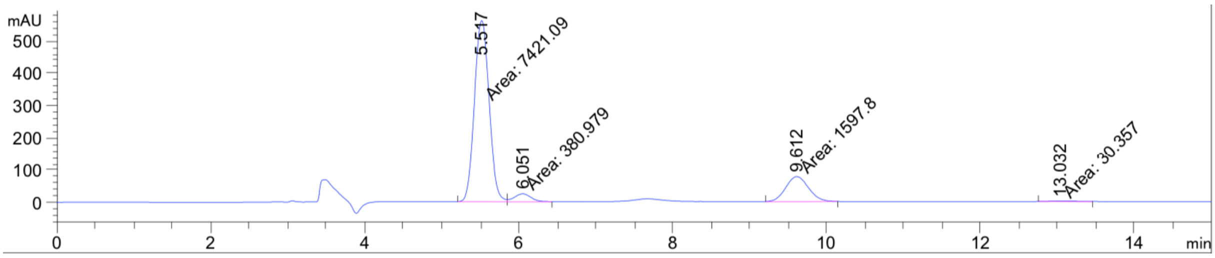

Peak #	RetTime [min]	Type	Width [min]	Area [mAU*s]	Height [mAU]	Area %
1	5.517	MF	0.2208	7421.09424	560.15454	78.6947
2	6.051	FM	0.2563	380.97888	24.77318	4.0400
3	9.612	MM	0.3436	1597.80359	77.50440	16.9434
4	13.032	MM	0.3993	30.35698	1.26698	0.3219

(*R*,*E*)-4,4,4-Trifluoro-1-(4-methoxyphenyl)-3-phenylbut-2-en-1-amine (4c)

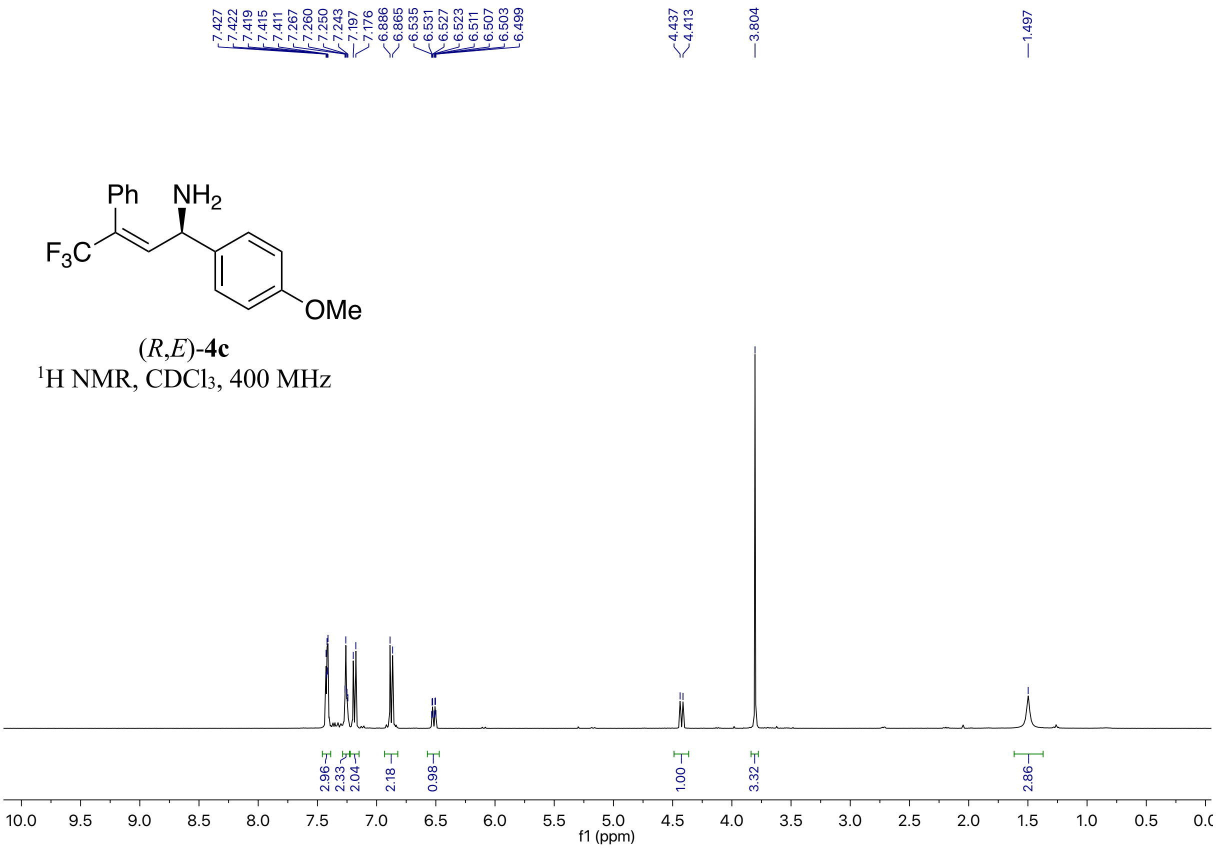

(*R*,*E*)-**4c**
^{1}H NMR, $CDCl_3$, 400 MHz

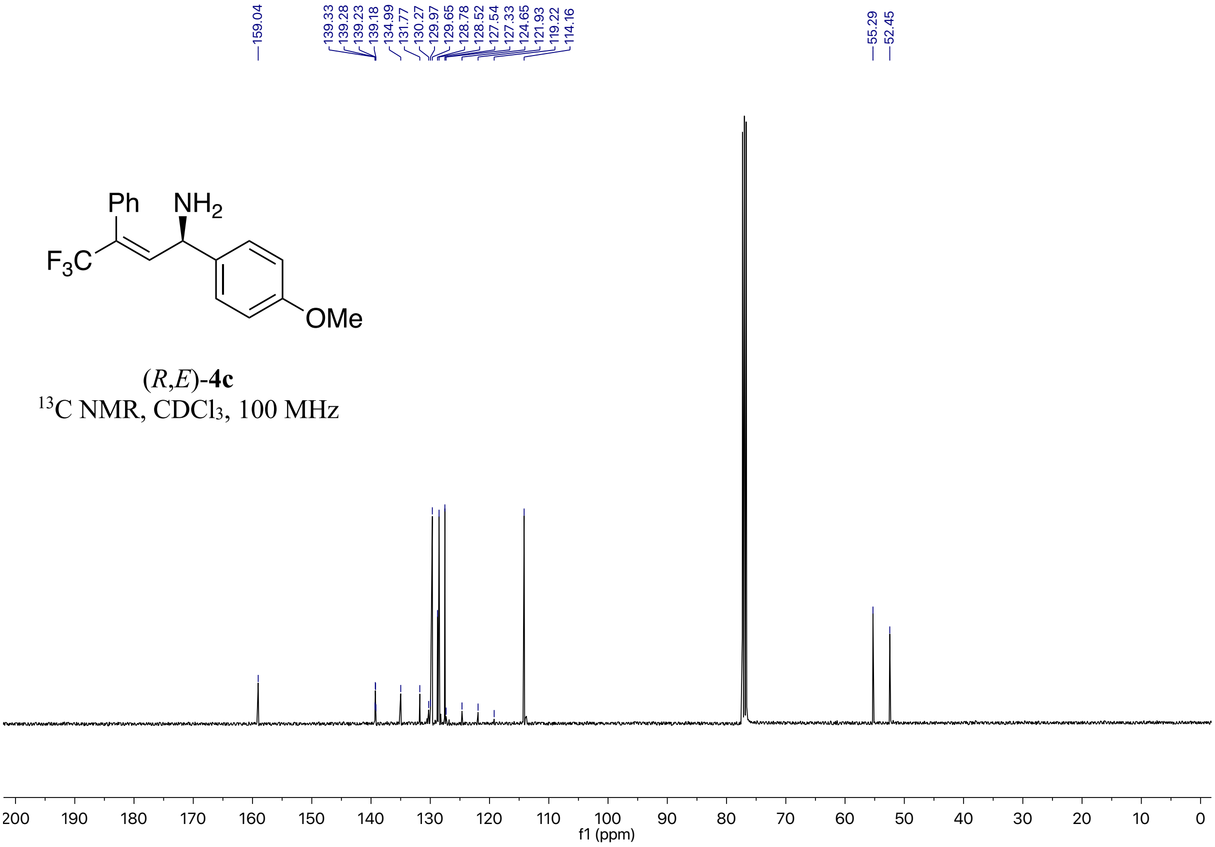

(*R*,*E*)-**4c**
^{13}C NMR, $CDCl_3$, 100 MHz

tert-Butyl (_R_,_E_)-(4,4,4-trifluoro-1-(4-methoxyphenyl)-3-phenylbut-2-en-1-yl)carbamate (4c')

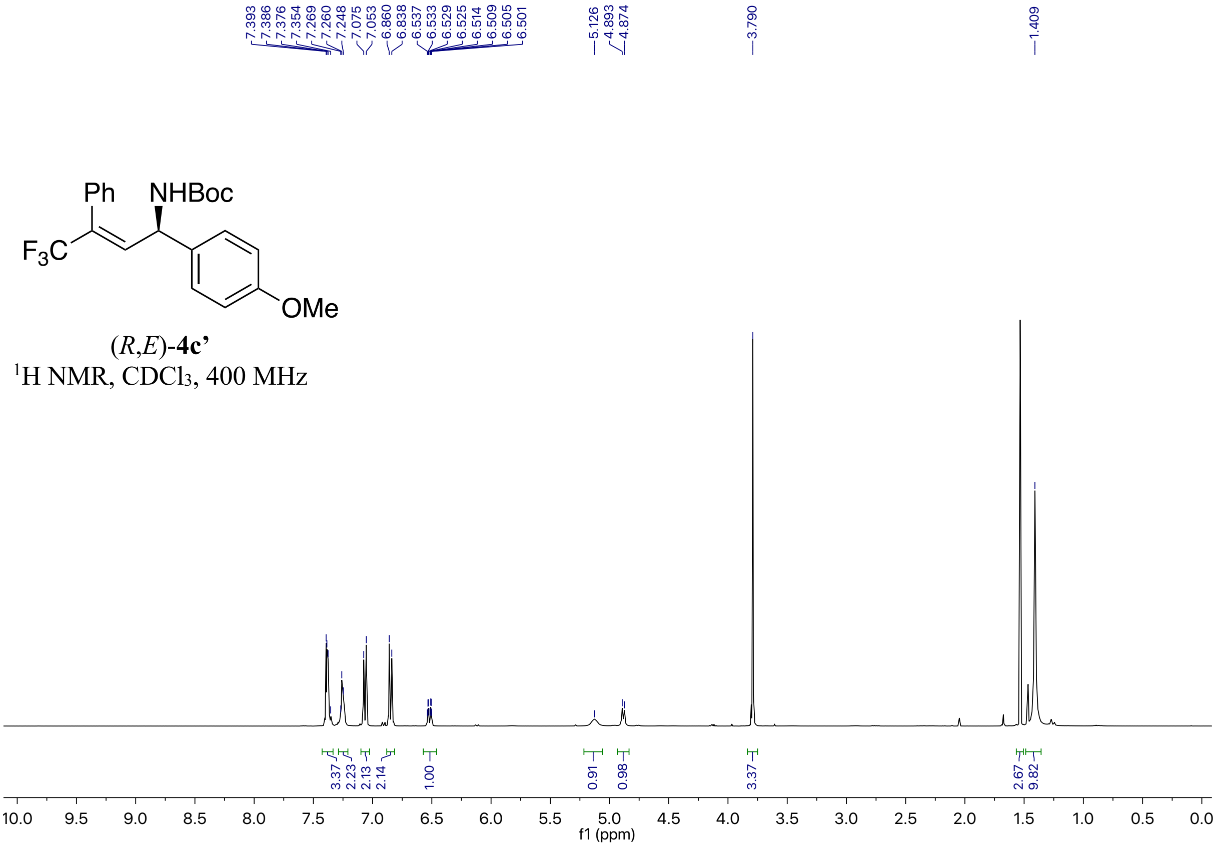

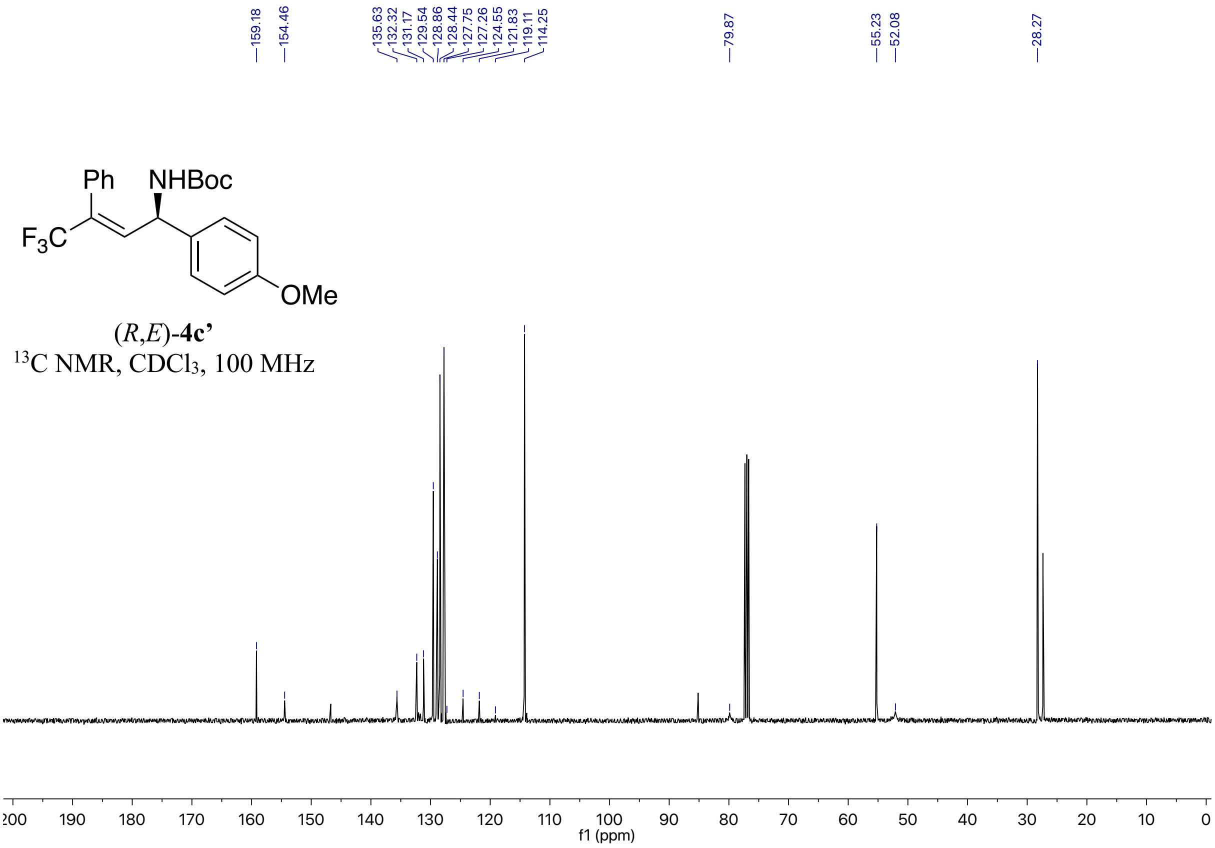

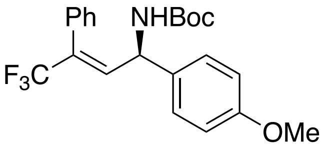

(*R*,*E*)-4c'

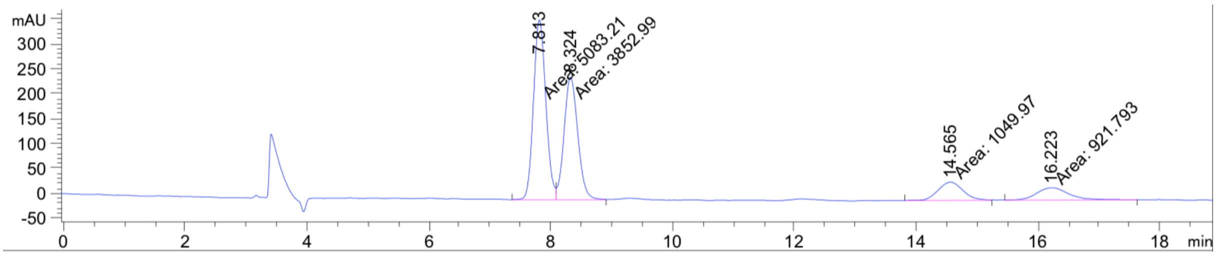

Peak #	RetTime [min]	Type	Width [min]	Area [mAU*s]	Height [mAU]	Area %
1	7.813	MF	0.2349	5083.21289	360.73505	46.6009
2	8.324	FM	0.2616	3852.99146	245.44814	35.3227
3	14.565	MM	0.4774	1049.96777	36.65691	9.6257
4	16.223	MM	0.6235	921.79266	24.63990	8.4506

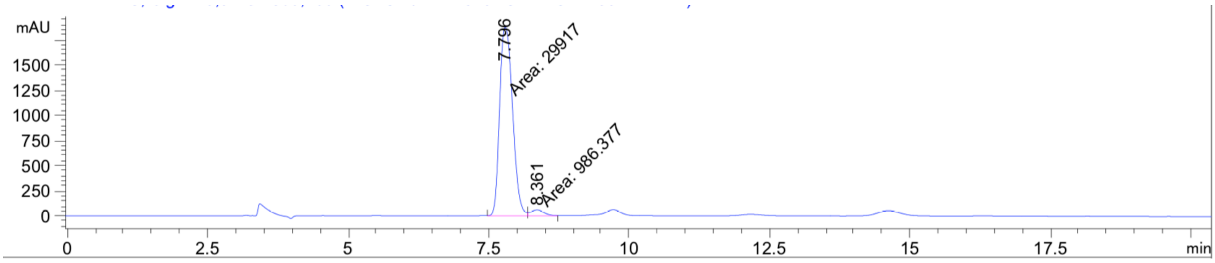

Peak #	RetTime [min]	Type	Width [min]	Area [mAU*s]	Height [mAU]	Area %
1	7.796	MF	0.2657	2.99170e4	1876.32739	96.8082
2	8.361	FM	0.2770	986.37738	59.34005	3.1918

(*R*,*E*)-1-(4-Bromophenyl)-4,4,4-trifluoro-3-phenylbut-2-en-1-amine 4d

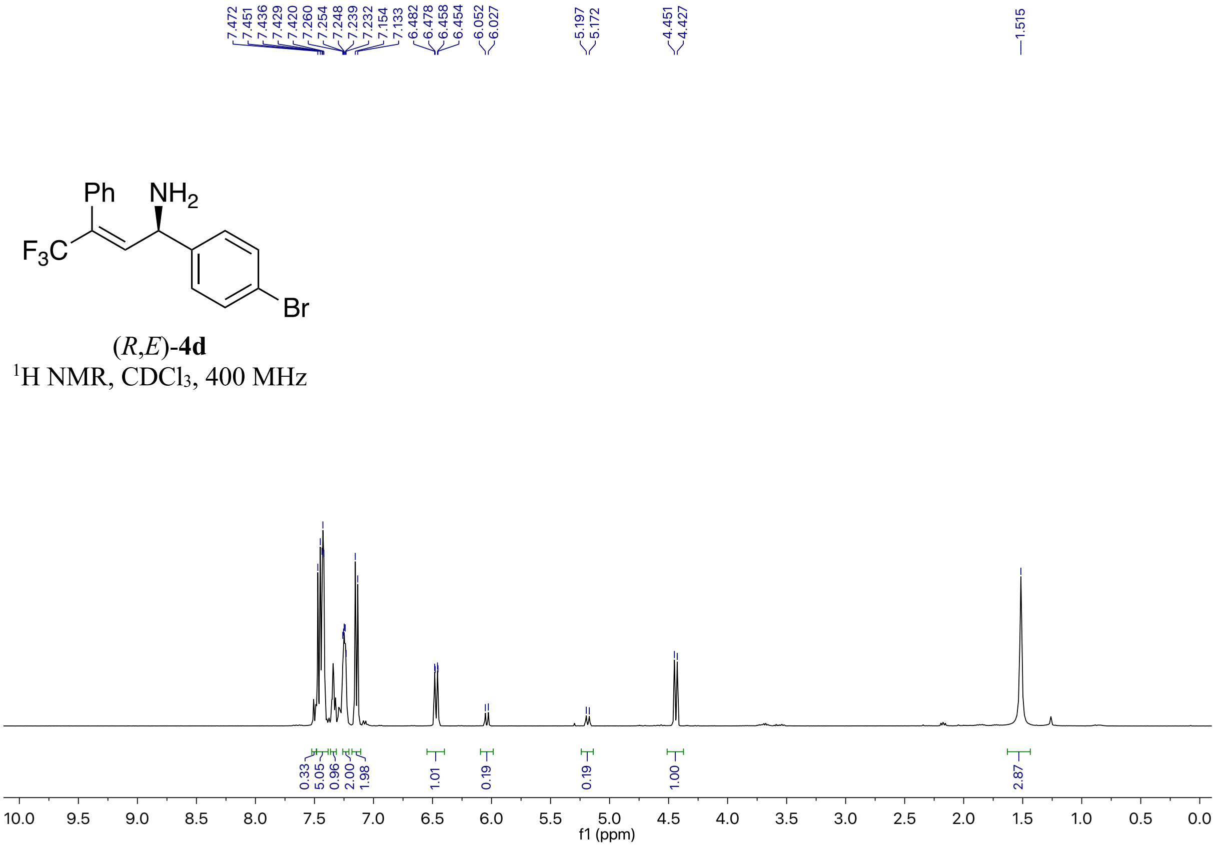

(*R*,*E*)-**4d**
^{1}H NMR, $CDCl_3$, 400 MHz

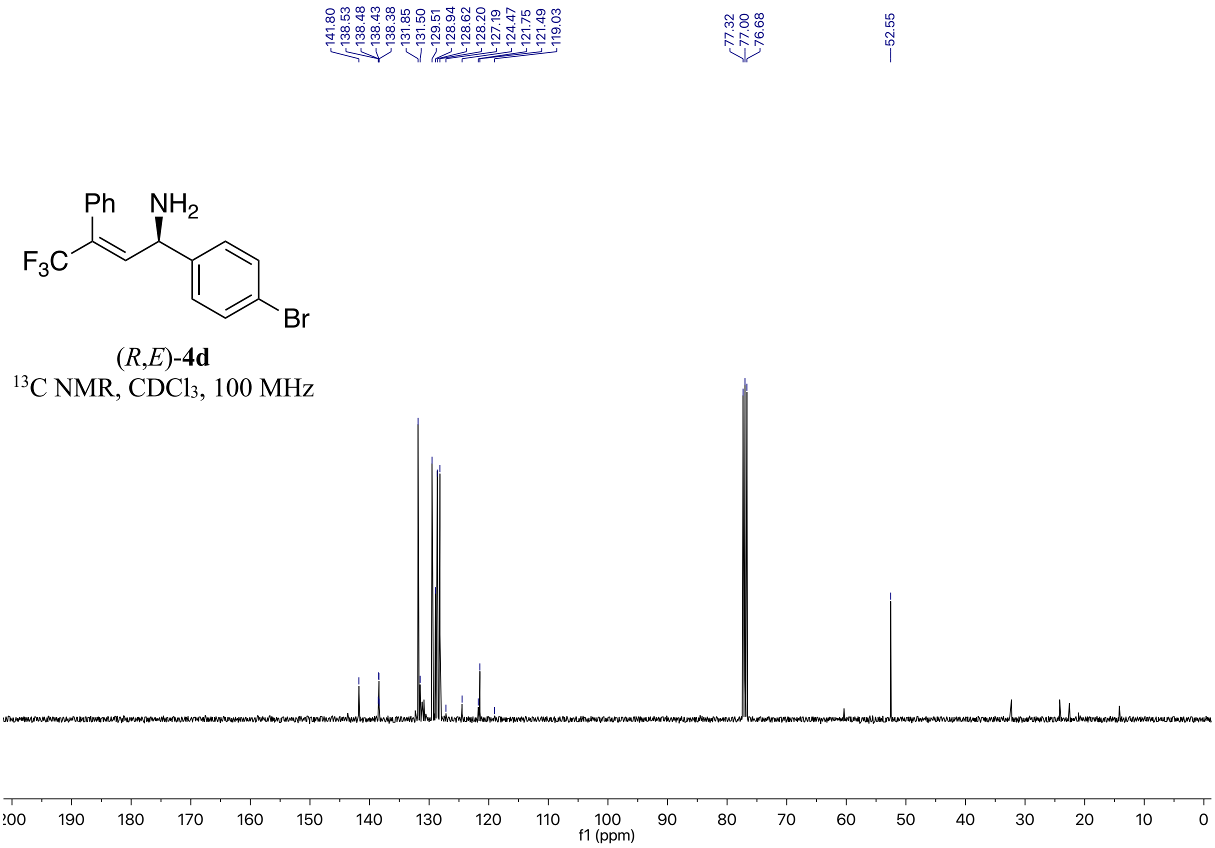

(*R*,*E*)-**4d**
^{13}C NMR, $CDCl_3$, 100 MHz

tert-Butyl (_R_,_E_)-(1-(4-bromophenyl)-4,4,4-trifluoro-3-phenylbut-2-en-1-yl)carbamate (4d')

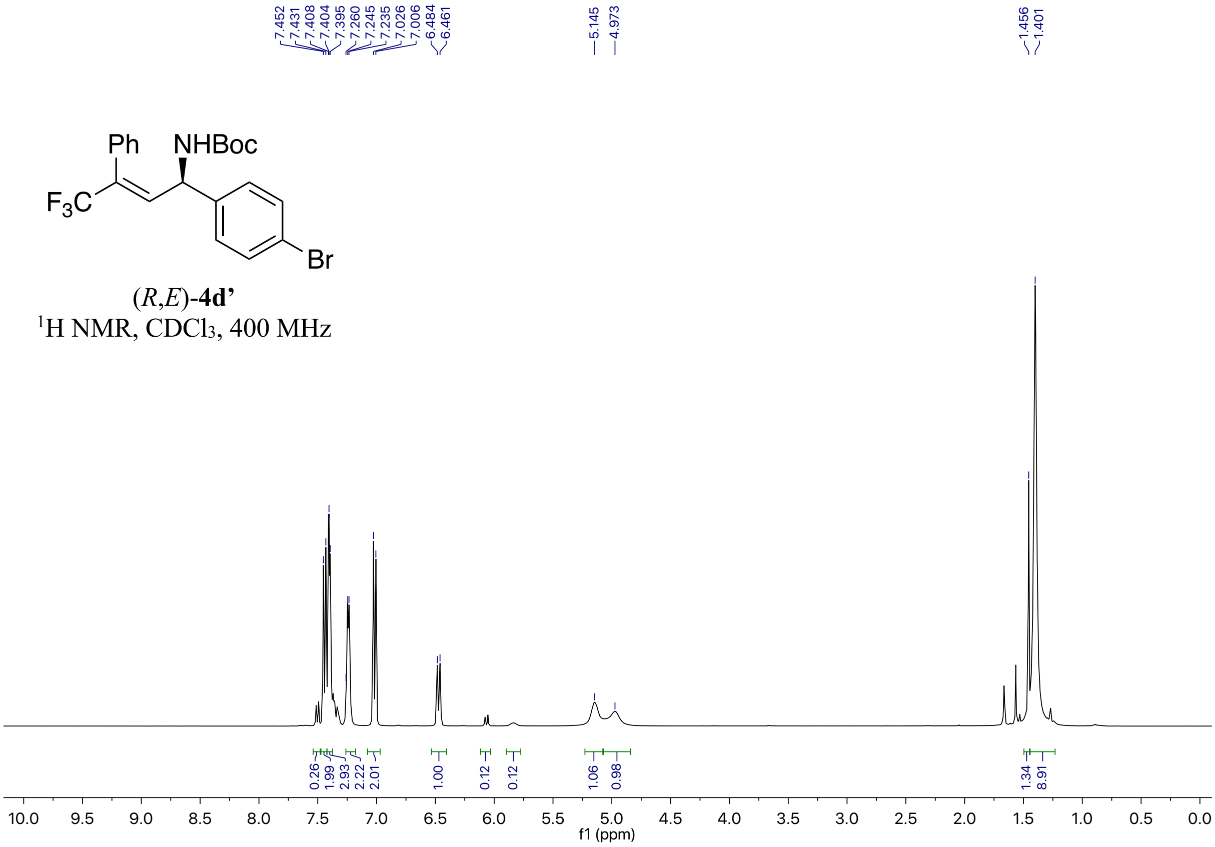

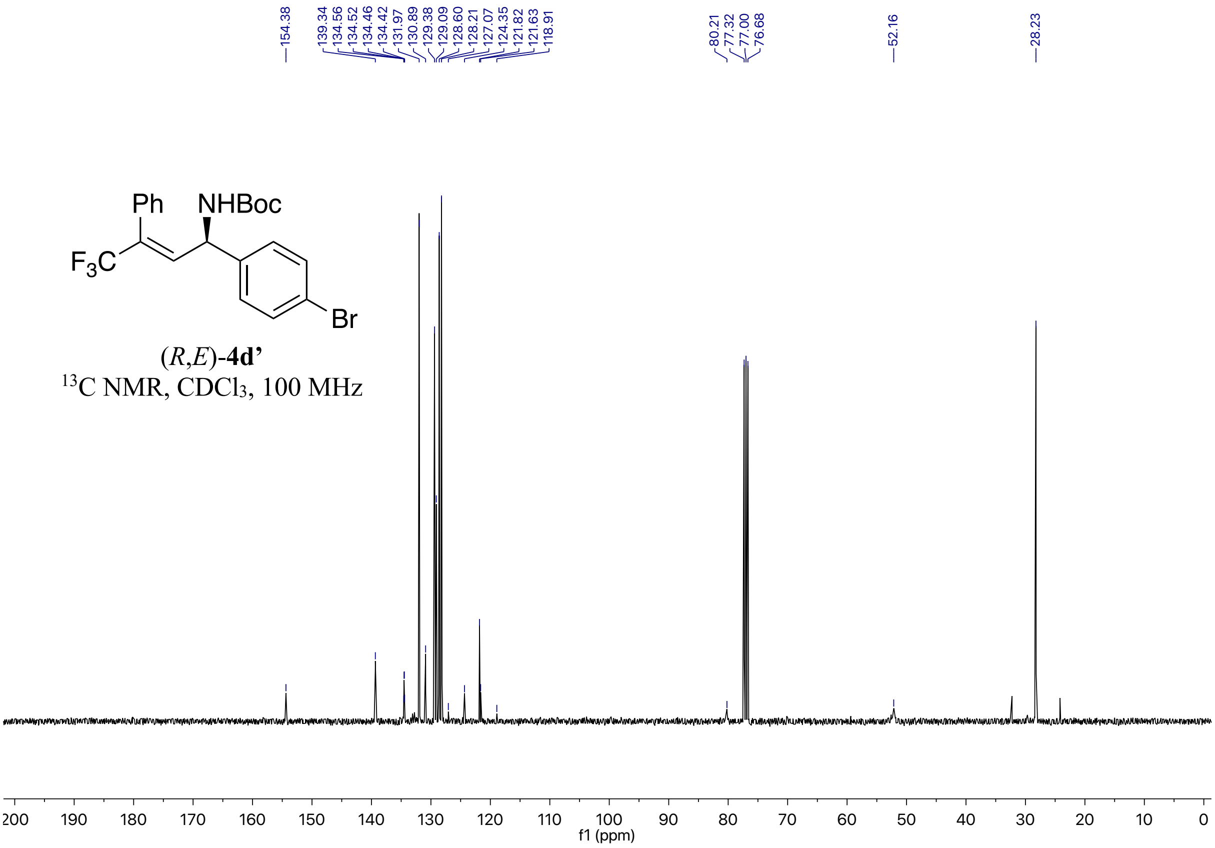

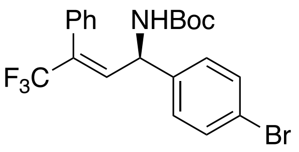

(*R*,*E*)-4d’

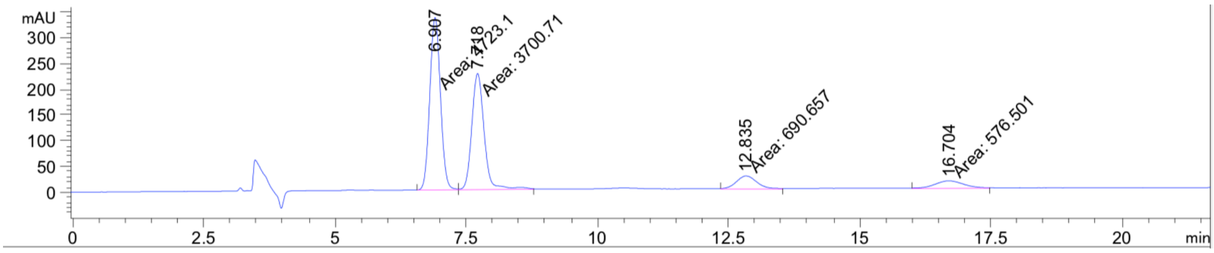

Peak #	RetTime [min]	Type	Width [min]	Area [mAU*s]	Height [mAU]	Area %
1	6.907	MF	0.2355	4723.09619	334.29535	48.7371
2	7.718	FM	0.2738	3700.70605	225.22873	38.1872
3	12.835	MM	0.4564	690.65741	25.22265	7.1268
4	16.704	MM	0.6539	576.50079	14.69383	5.9489

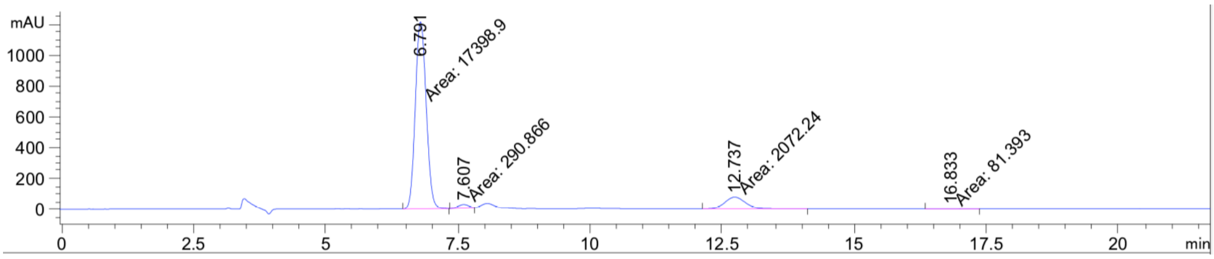

Peak #	RetTime [min]	Type	Width [min]	Area [mAU*s]	Height [mAU]	Area %
1	6.791	MM	0.2388	1.73989e4	1214.15039	87.6811
2	7.607	MM	0.2176	290.86615	22.28024	1.4658
3	12.737	MM	0.4546	2072.23926	75.97820	10.4430
4	16.833	MM	0.8601	81.39301	1.57716	0.4102

(*R*,*E*)-4,4,4-Trifluoro-3-phenyl-1-(4-(trifluoromethyl)phenyl)but-2-en-1-amine (4e)

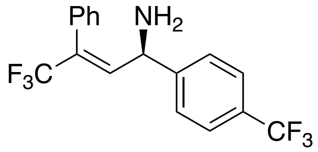

(*R*,*E*)-**4e**

^{1}H NMR, $CDCl_3$, 400 MHz

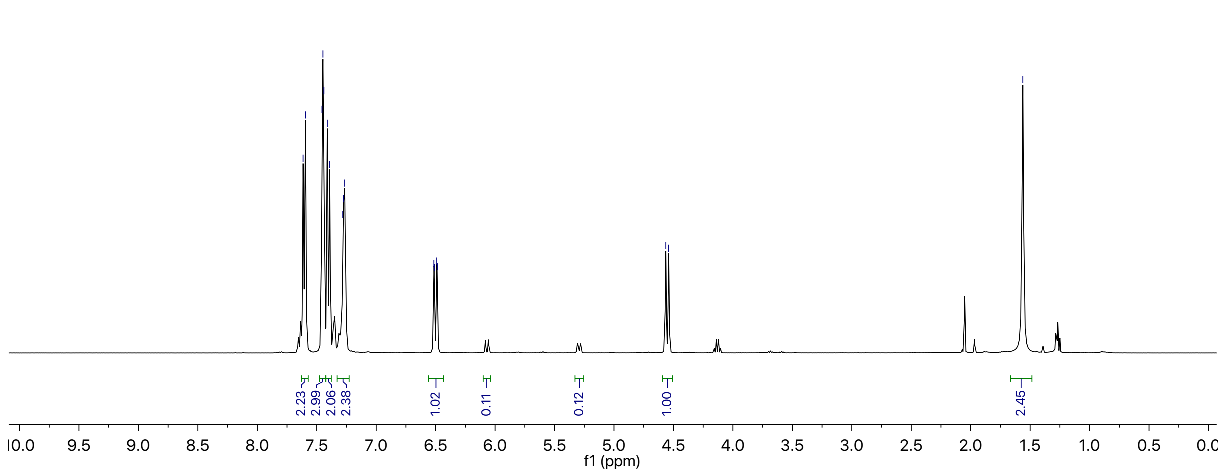

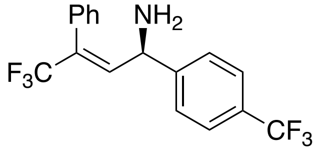

(*R*,*E*)-**4e**

^{13}C NMR, $CDCl_3$, 100 MHz

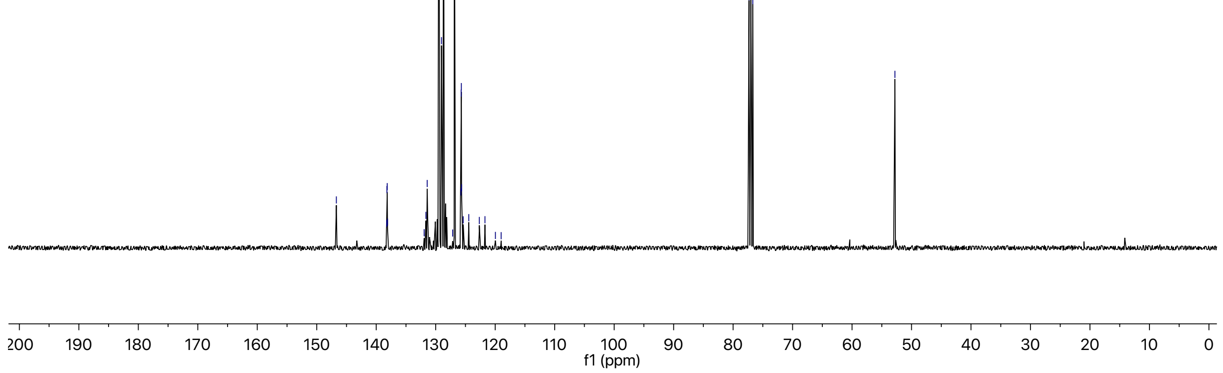

***tert*-Butyl (*R*,*E*)-(4,4,4-trifluoro-3-phenyl-1-(4-(trifluoromethyl)phenyl)but-2-en-1-yl)carbamate (4e')**

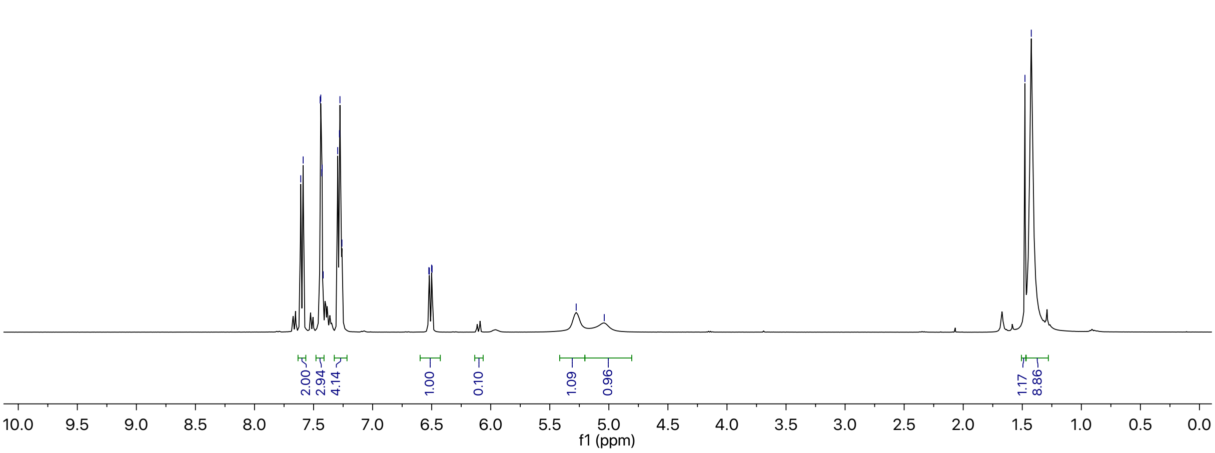

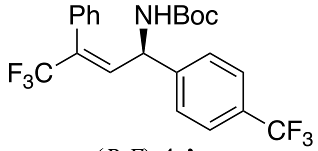

(*R*,*E*)-**4e'**
^{1}H NMR, $CDCl_3$, 400 MHz

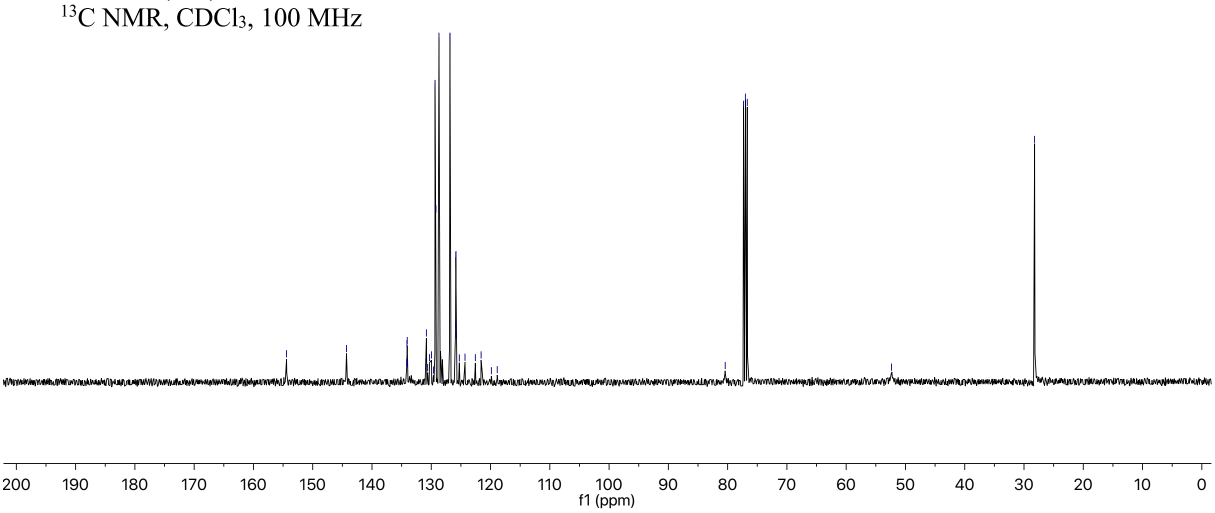

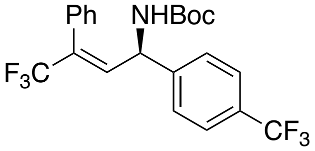

(*R*,*E*)-**4e'**
^{13}C NMR, $CDCl_3$, 100 MHz

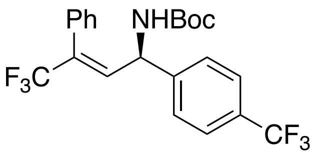

(*R*,*E*)-4e’

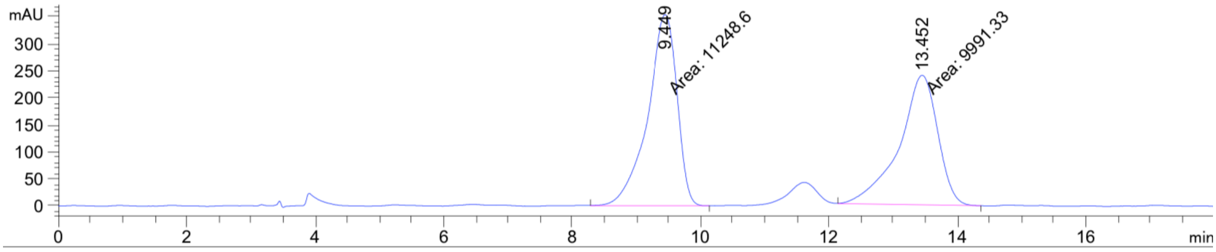

Peak #	RetTime [min]	Type	Width [min]	Area [mAU*s]	Height [mAU]	Area %
1	9.449	MM	0.5296	1.12486e4	354.01556	52.9596
2	13.452	MM	0.6940	9991.32813	239.93785	47.0404

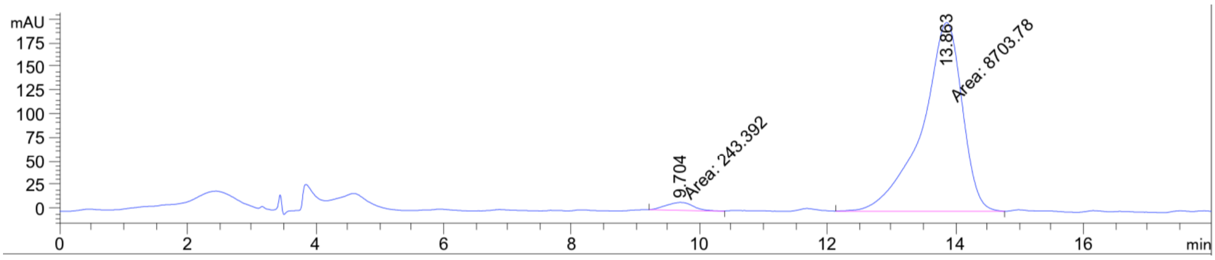

Peak #	RetTime [min]	Type	Width [min]	Area [mAU*s]	Height [mAU]	Area %
1	9.704	MM	0.4881	243.39159	8.31013	2.7203
2	13.863	MM	0.7281	8703.77832	199.24216	97.2797

(*R*,*E*)-4,4,4-Trifluoro-1-(naphthalen-2-yl)-3-phenylbut-2-en-1-amine (4f)

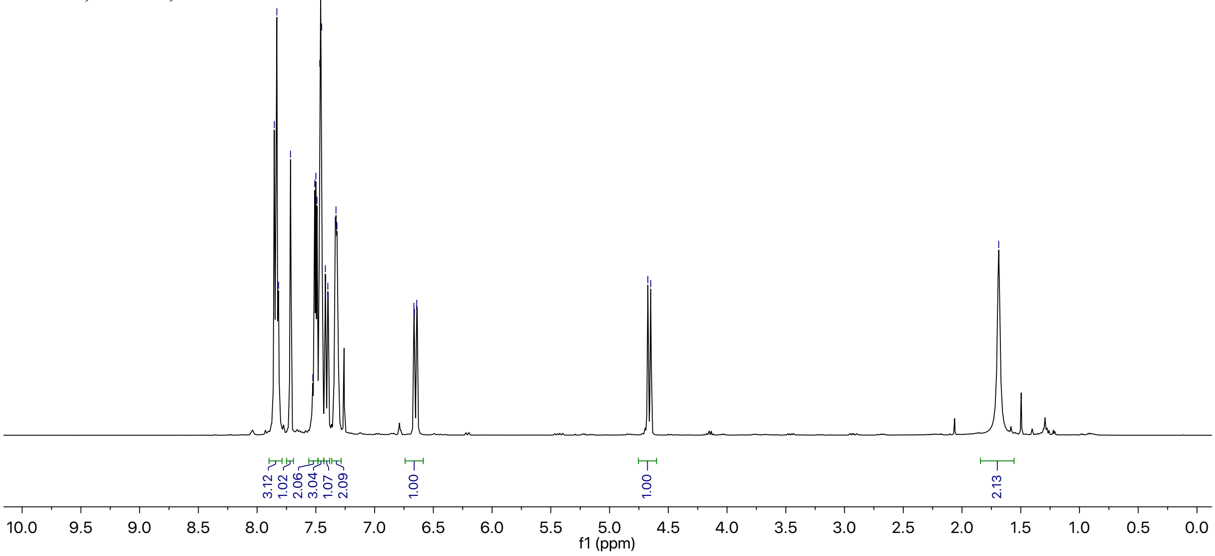

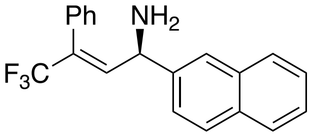

(*R*,*E*)-**4f**

^{1}H NMR, CDCl$_3$, 400 MHz

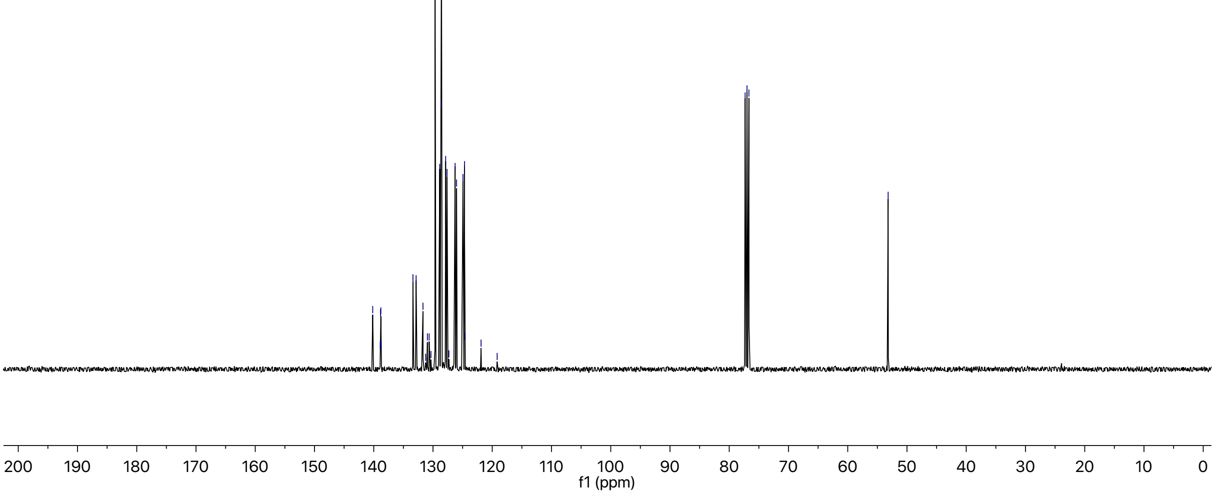

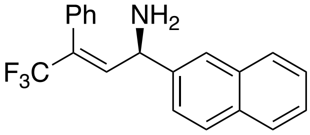

(*R*,*E*)-**4f**

^{13}C NMR, CDCl$_3$, 100 MHz

***tert*-Butyl (*R*,*E*)-(4,4,4-trifluoro-1-(naphthalen-2-yl)-3-phenylbut-2-en-1-yl)carbamate (4f’)**

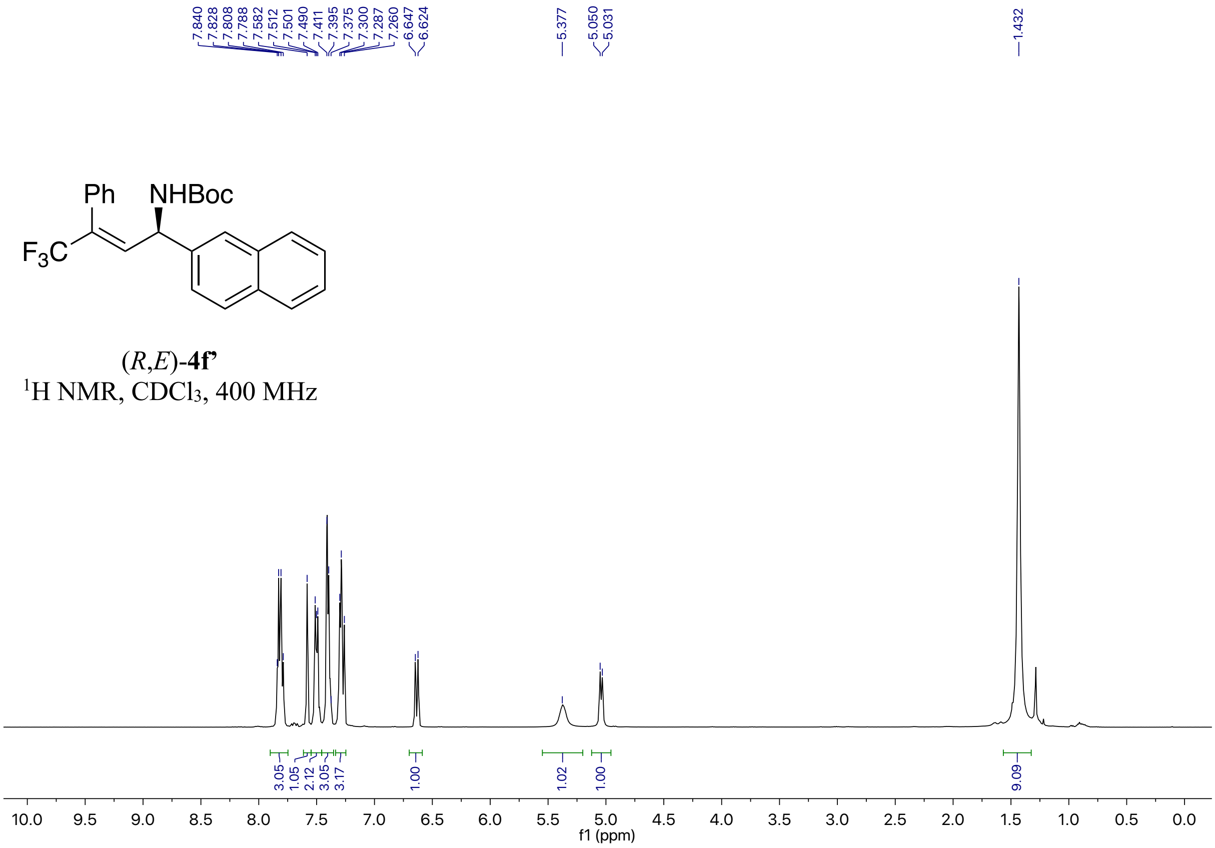

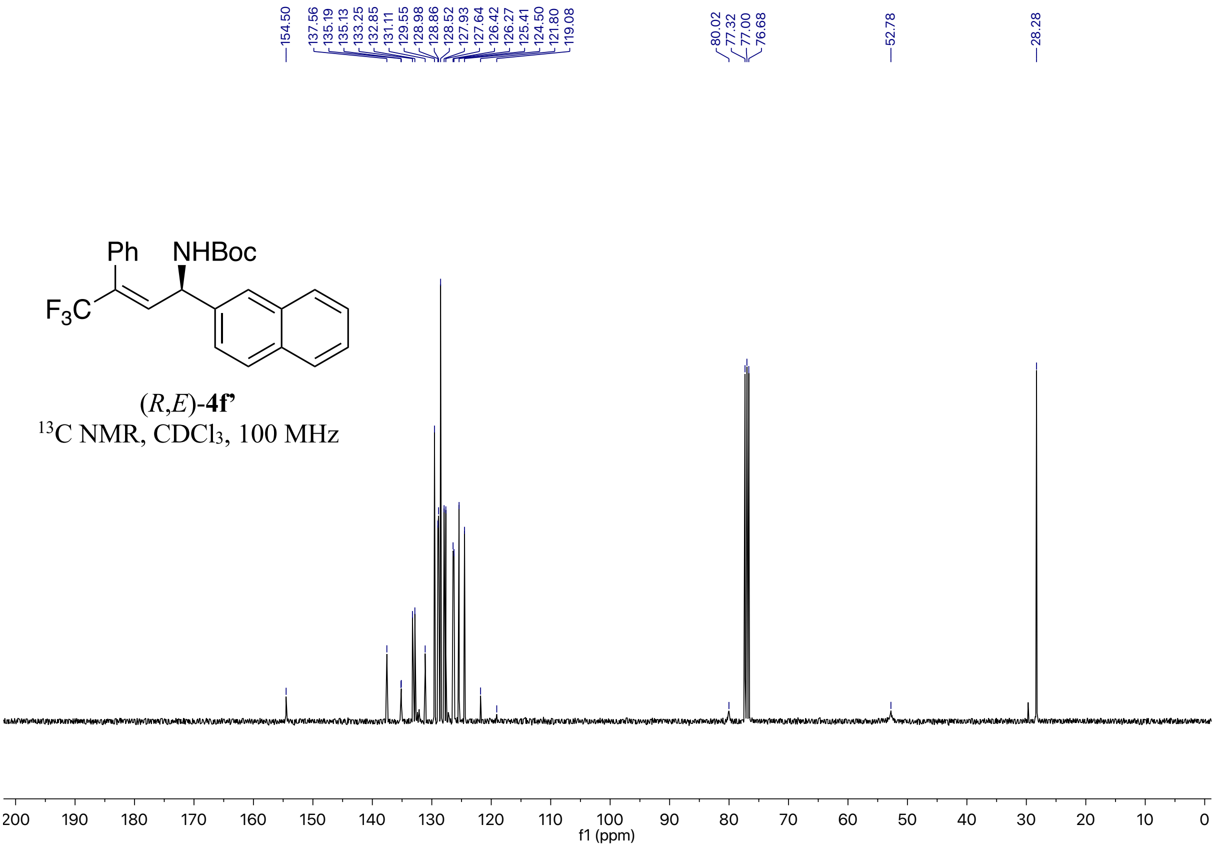

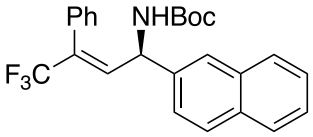

(*R*,*E*)-4f'

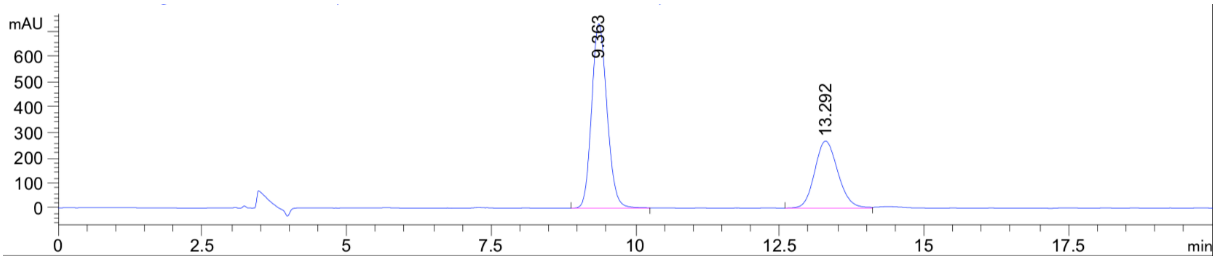

Peak #	RetTime [min]	Type	Width [min]	Area [mAU*s]	Height [mAU]	Area %
1	9.363	BV	0.2899	1.35227e4	730.63855	64.6200
2	13.292	VV	0.4288	7403.80908	265.95807	35.3800

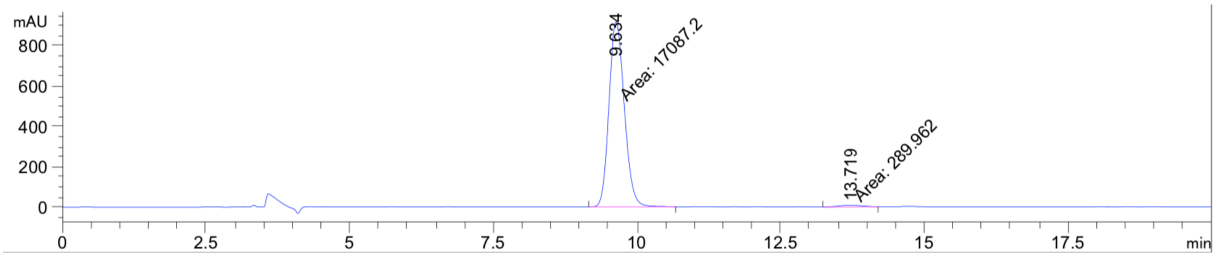

Peak #	RetTime [min]	Type	Width [min]	Area [mAU*s]	Height [mAU]	Area %
1	9.634	MM	0.3103	1.70872e4	917.83441	98.3314
2	13.719	MM	0.5318	289.96222	9.08709	1.6686

(*R*,*E*)-4,4,4-Trifluoro-1-(3-methoxyphenyl)-3-phenylbut-2-en-1-amine (4g)

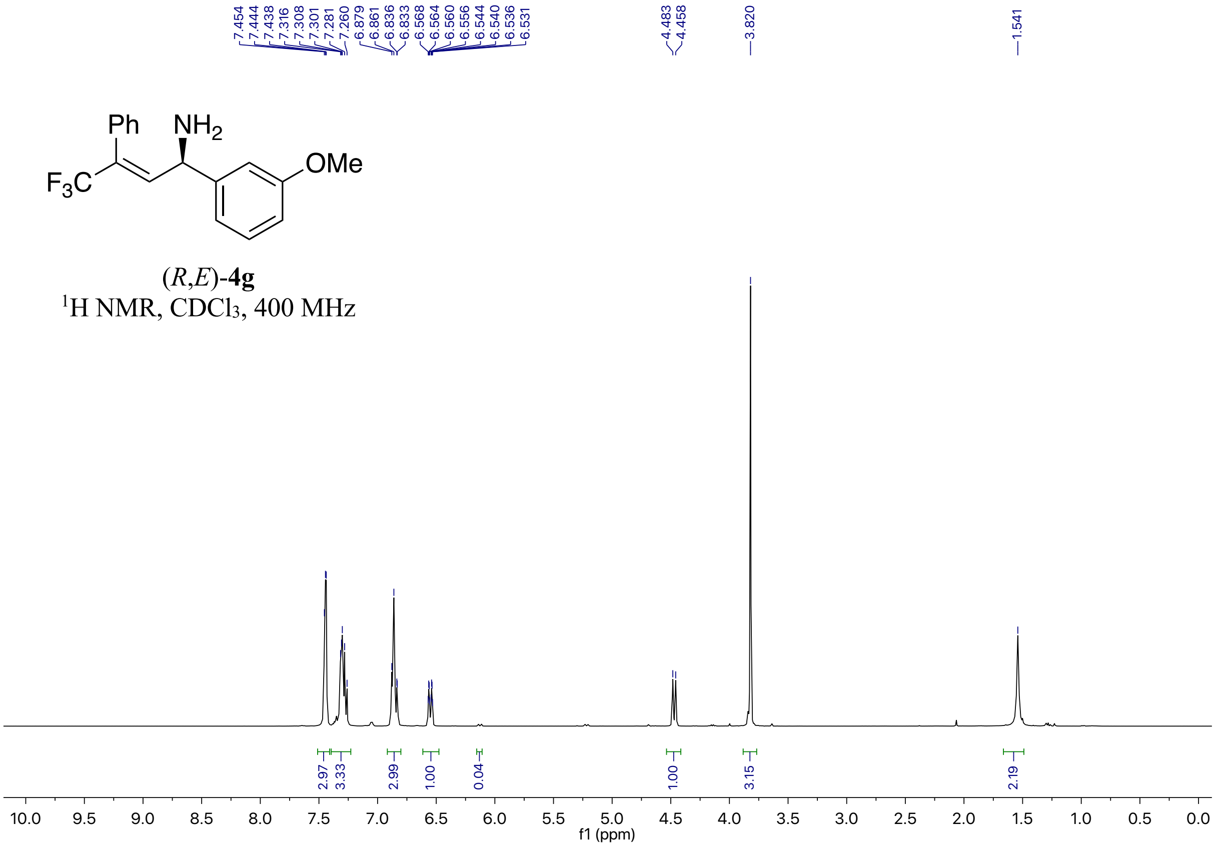

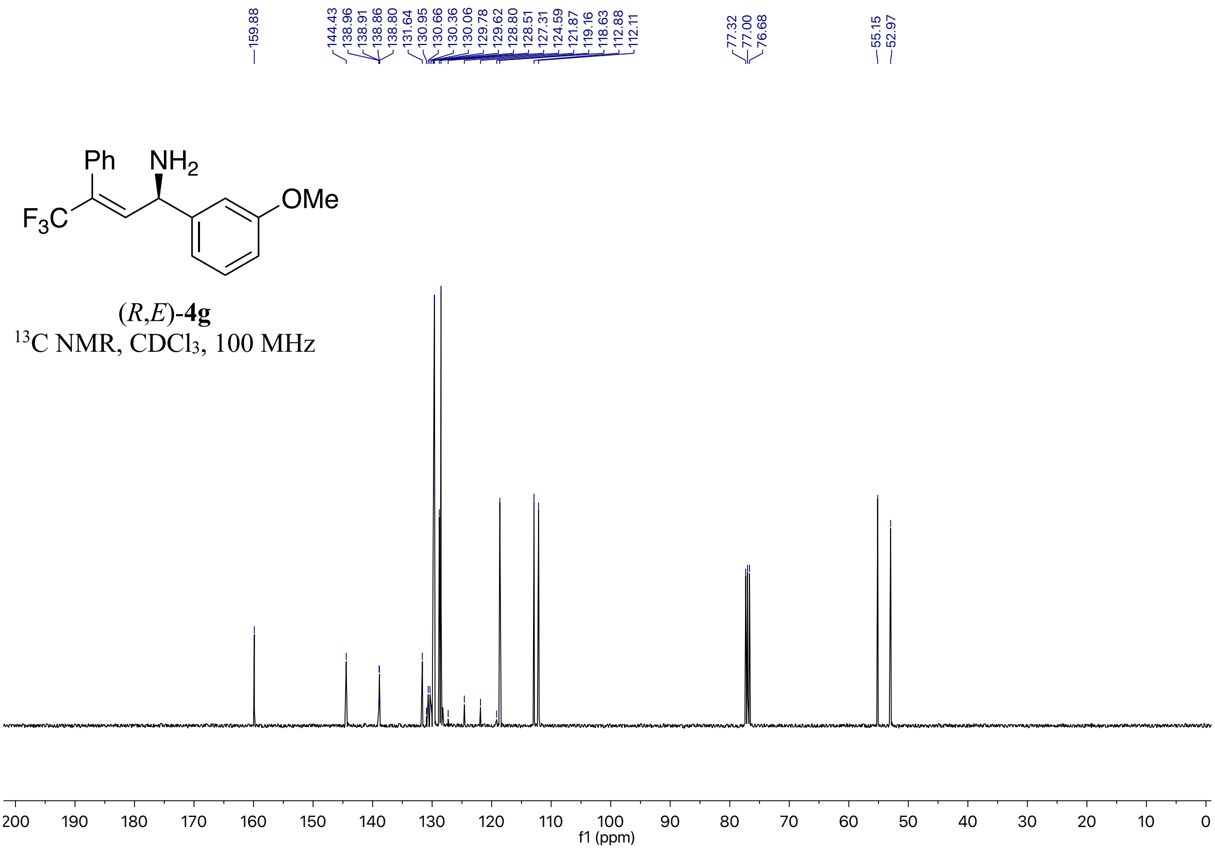

tert-Butyl (R,E)-(4,4,4-trifluoro-1-(3-methoxyphenyl)-3-phenylbut-2-en-1-yl)carbamate (4g')

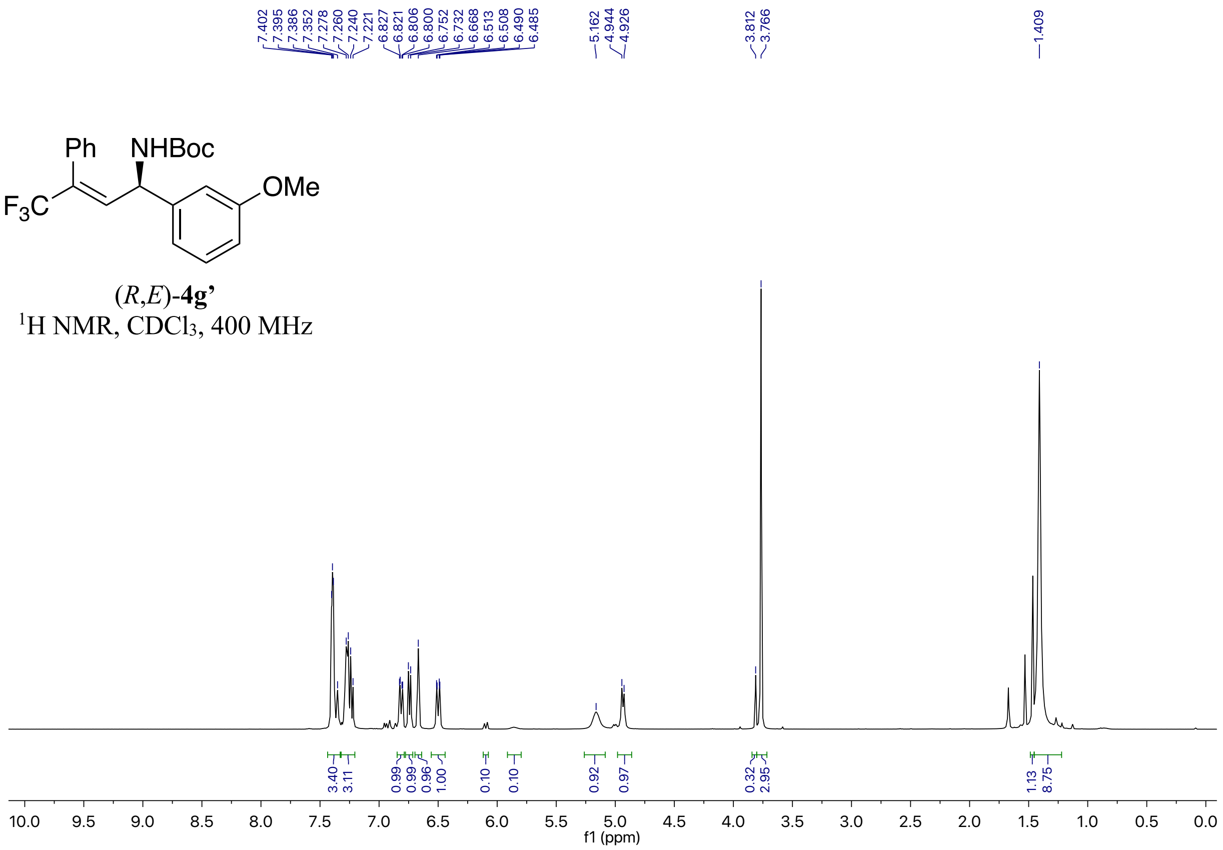

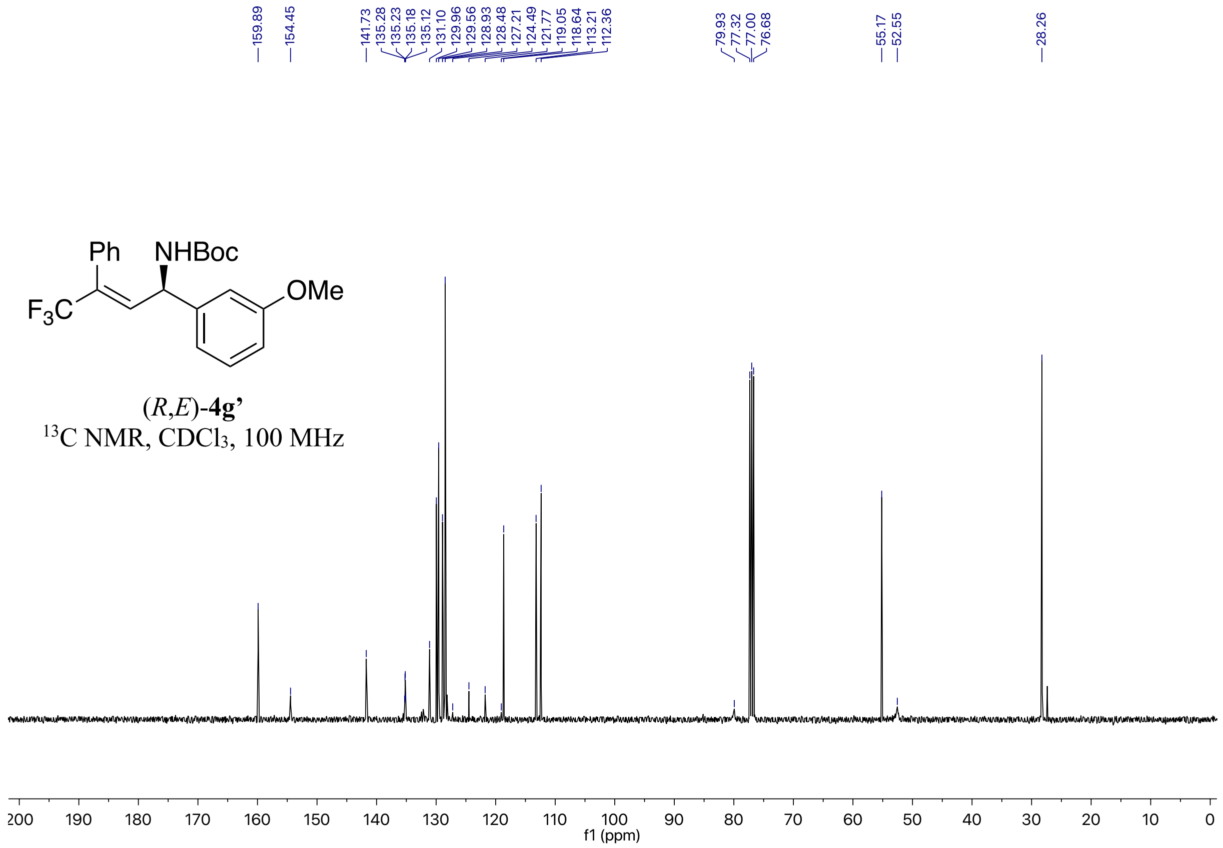

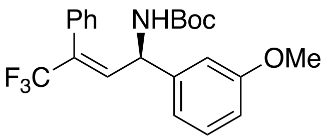

(*R*,*E*)-4g'

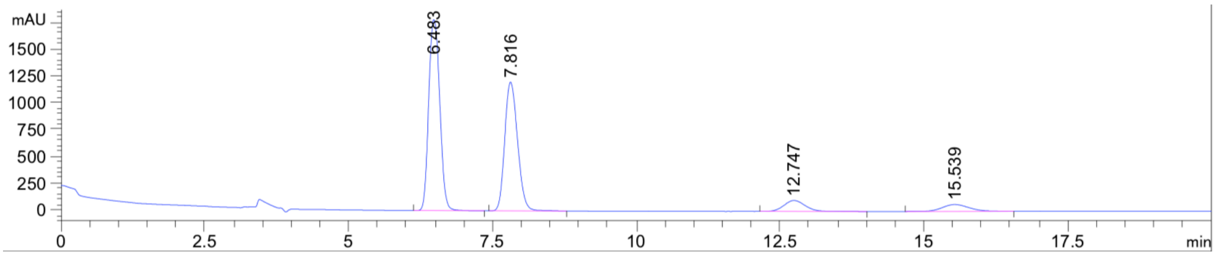

Peak #	RetTime [min]	Type	Width [min]	Area [mAU*s]	Height [mAU]	Area %
1	6.483	BB	0.2139	2.37795e4	1779.48376	50.3735
2	7.816	VB	0.2431	1.86578e4	1201.85327	39.5240
3	12.747	VV	0.3940	2637.63428	101.88622	5.5875
4	15.539	VV	0.4991	2131.39990	64.23135	4.5151

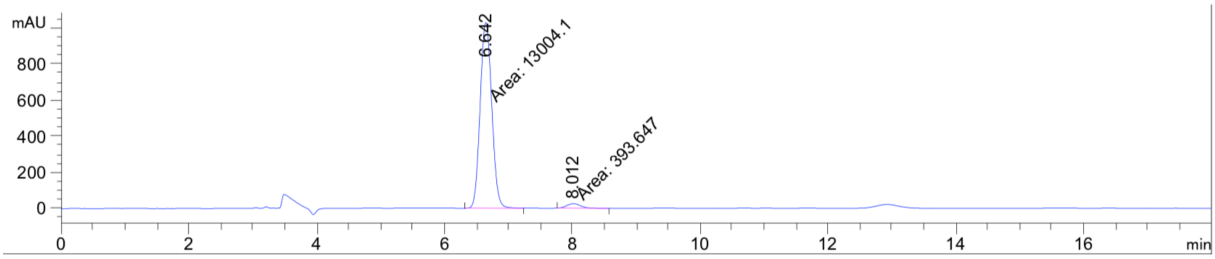

Peak #	RetTime [min]	Type	Width [min]	Area [mAU*s]	Height [mAU]	Area %
1	6.642	MM	0.2102	1.30041e4	1031.17188	97.0618
2	8.012	MM	0.2600	393.64725	25.23112	2.9382

(*R*,*E*)-4,4,4-Trifluoro-3-phenyl-1-(3-(trifluoromethyl)phenyl)but-2-en-1-amine (4h)

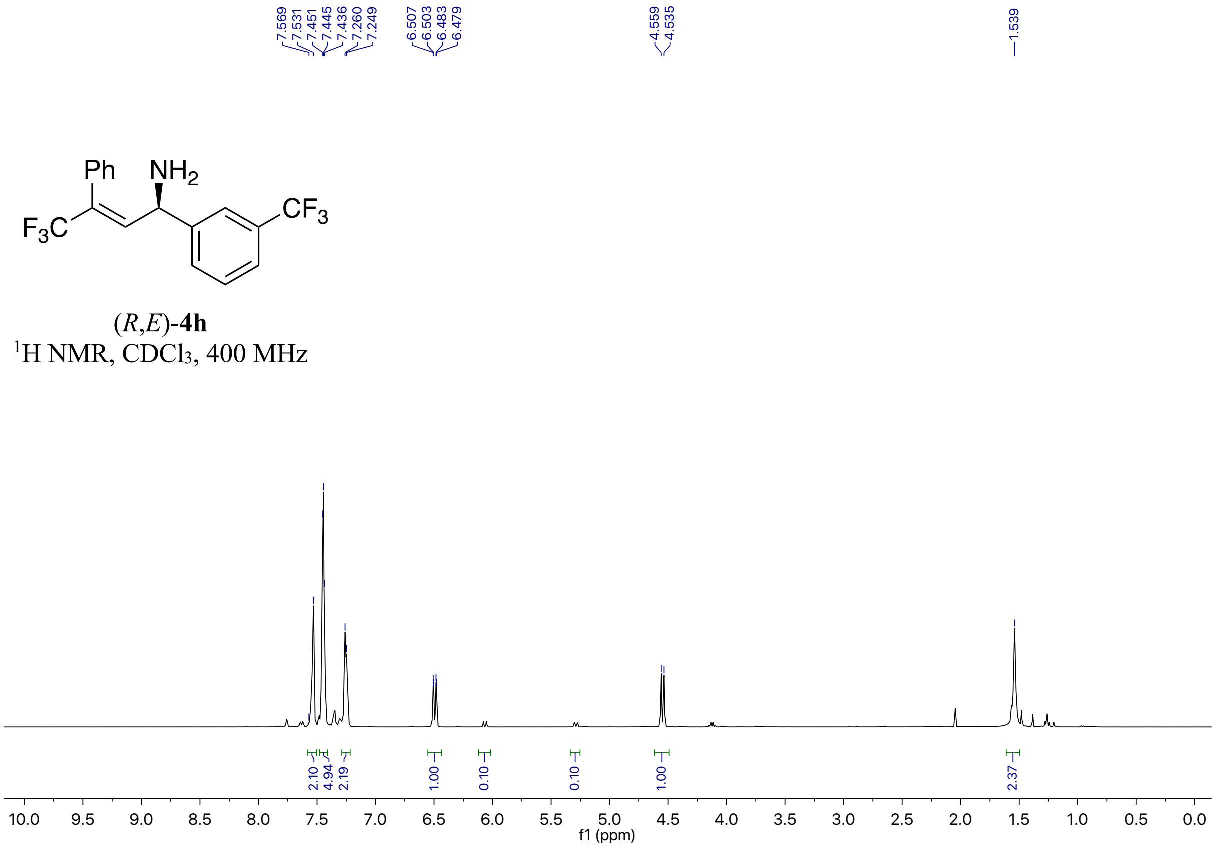

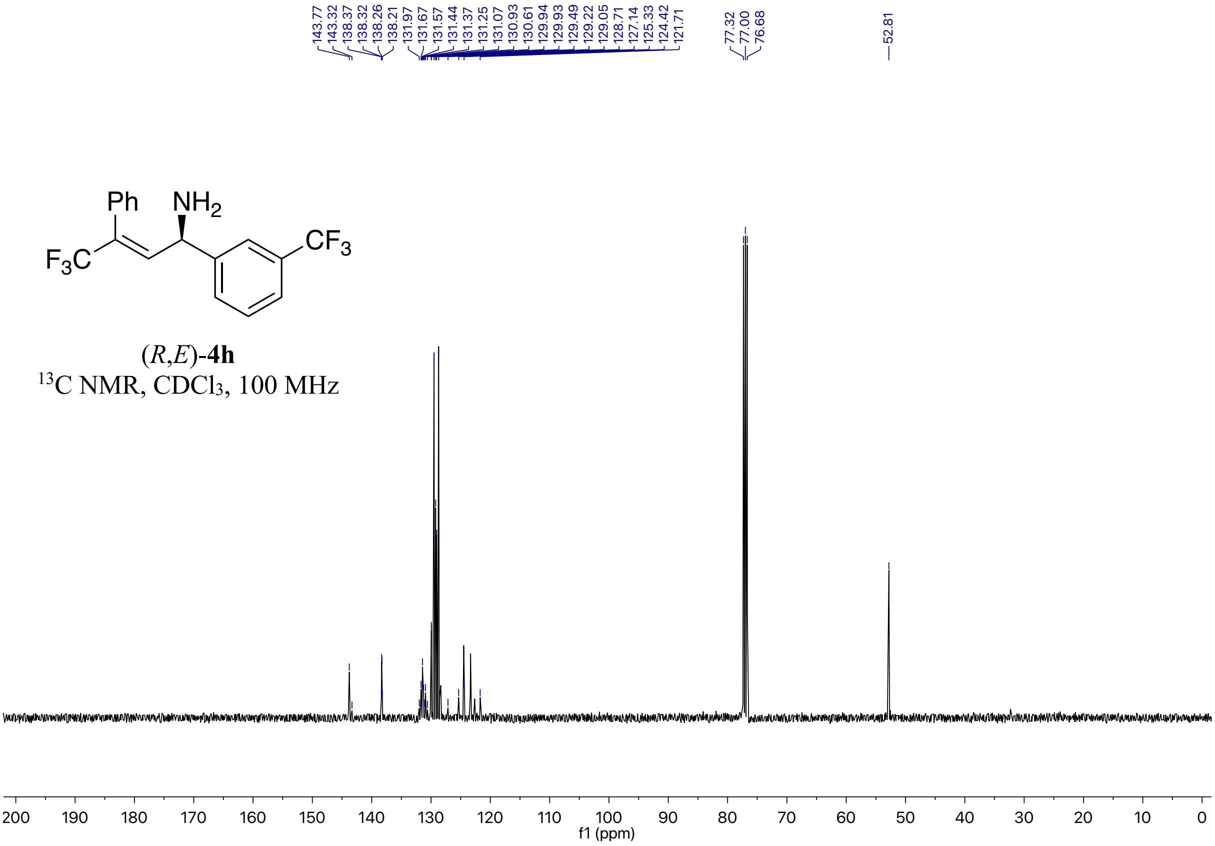

***tert*-Butyl (*R*,*E*)-(4,4,4-trifluoro-3-phenyl-1-(3-(trifluoromethyl)phenyl)but-2-en-1-yl)carbamate (4h')**

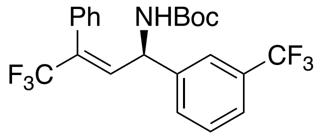

(*R*,*E*)-**4h'**
^{1}H NMR, $CDCl_3$, 400 MHz

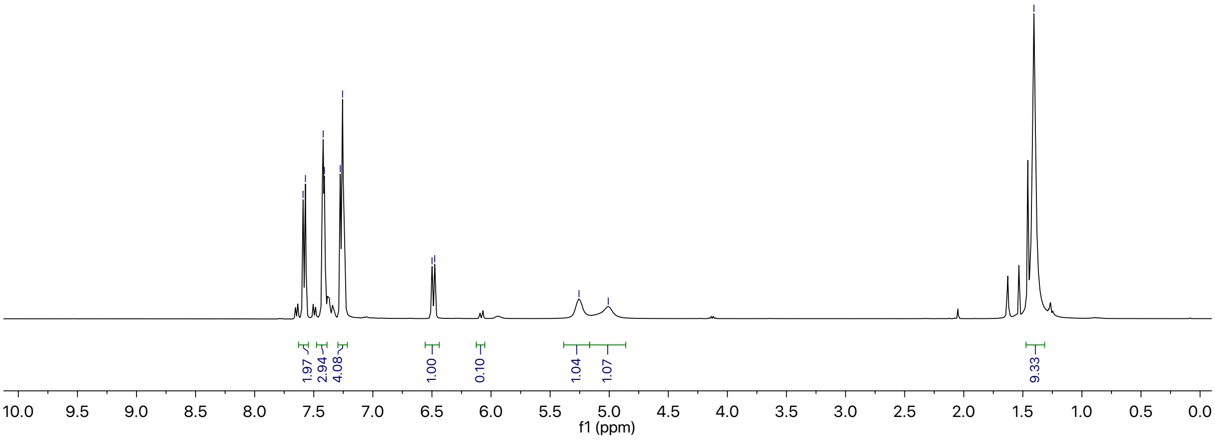

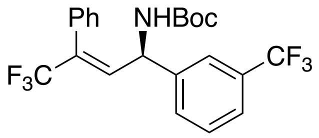

(*R*,*E*)-**4h'**
^{13}C NMR, $CDCl_3$, 100 MHz

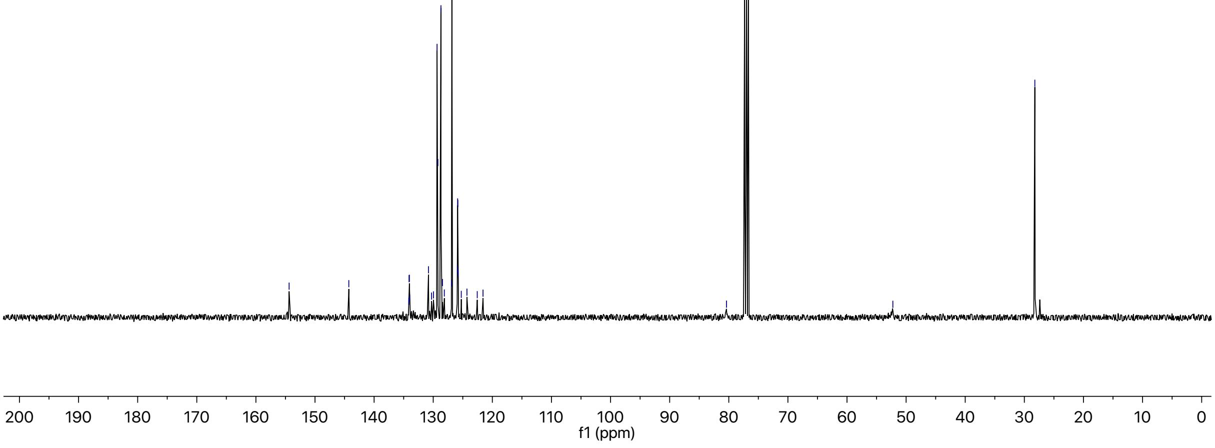

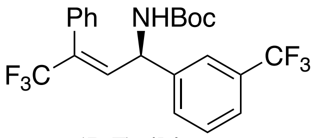

(*R*,*E*)-4h’

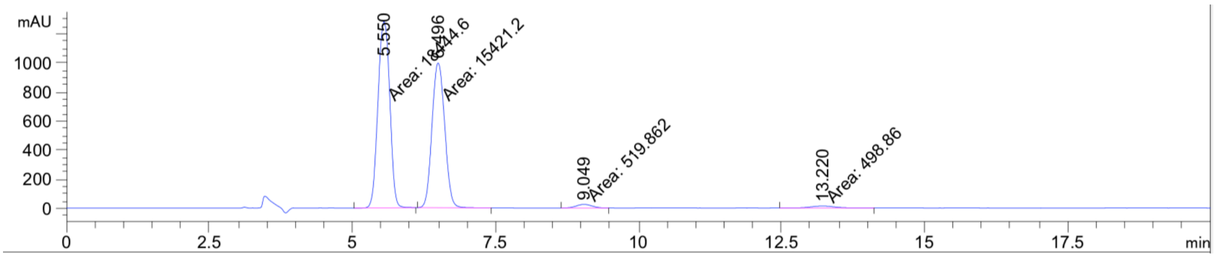

Peak #	RetTime [min]	Type	Width [min]	Area [mAU*s]	Height [mAU]	Area %
1	5.550	MM	0.2397	1.84446e4	1282.27478	52.8734
2	6.496	MM	0.2578	1.54212e4	996.95721	44.2064
3	9.049	MM	0.3394	519.86237	25.53016	1.4902
4	13.220	MM	0.5746	498.86026	14.47086	1.4300

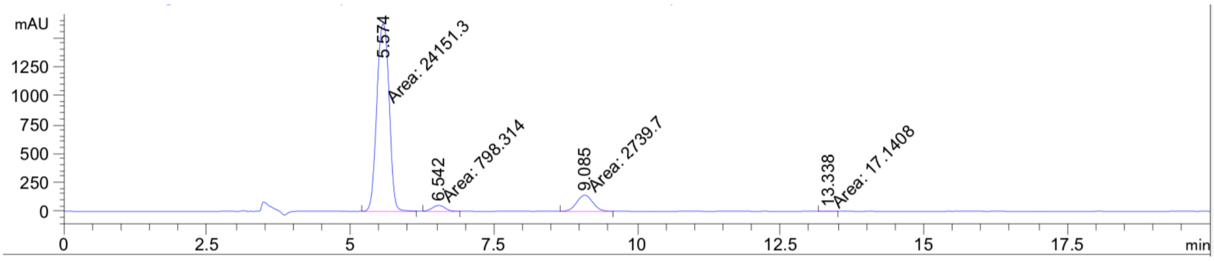

Peak #	RetTime [min]	Type	Width [min]	Area [mAU*s]	Height [mAU]	Area %
1	5.574	MM	0.2491	2.41513e4	1615.83179	87.1685
2	6.542	MM	0.2618	798.31378	50.81420	2.8813
3	9.085	MM	0.3276	2739.70117	139.37032	9.8883
4	13.338	MM	0.2488	17.14081	1.14808	0.0619

(*R*,*E*)-4,4,4-Trifluoro-1-(2-methoxyphenyl)-3-phenylbut-2-en-1-amine (4i)

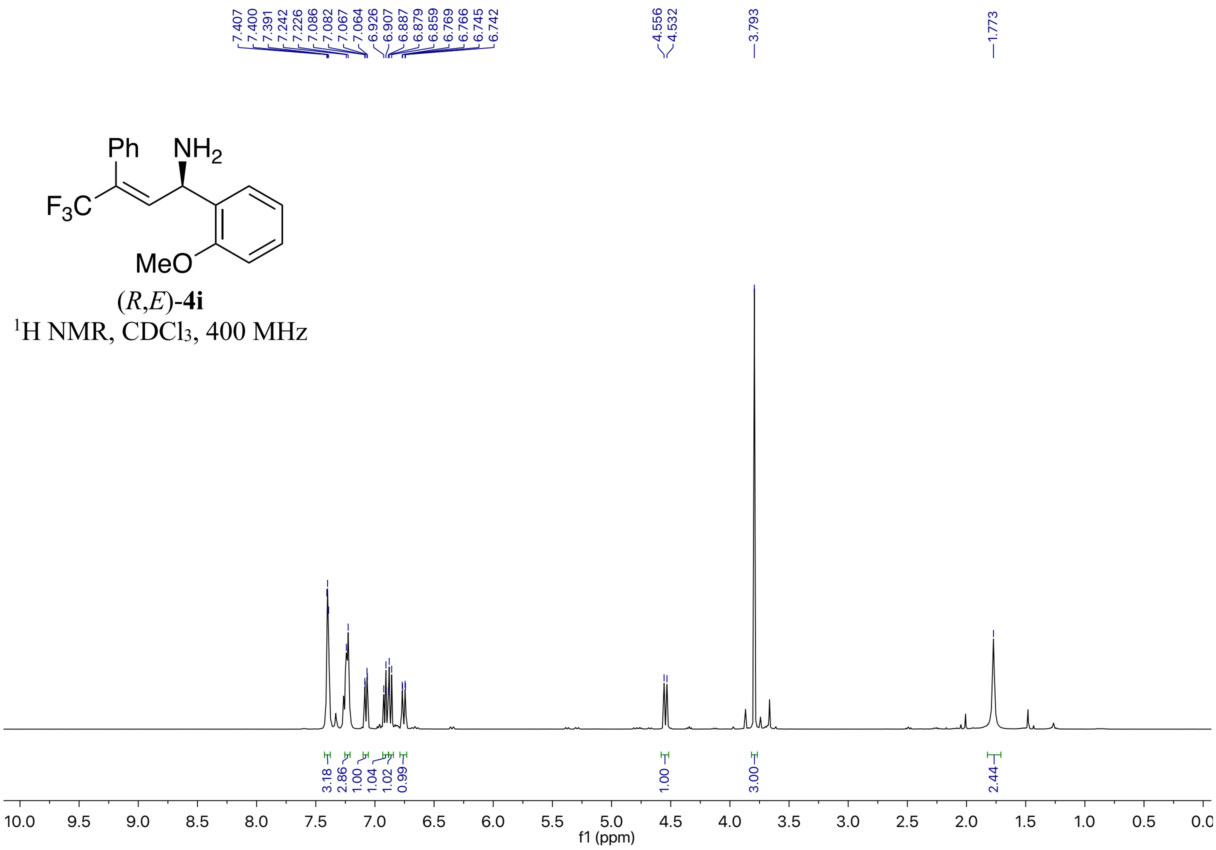

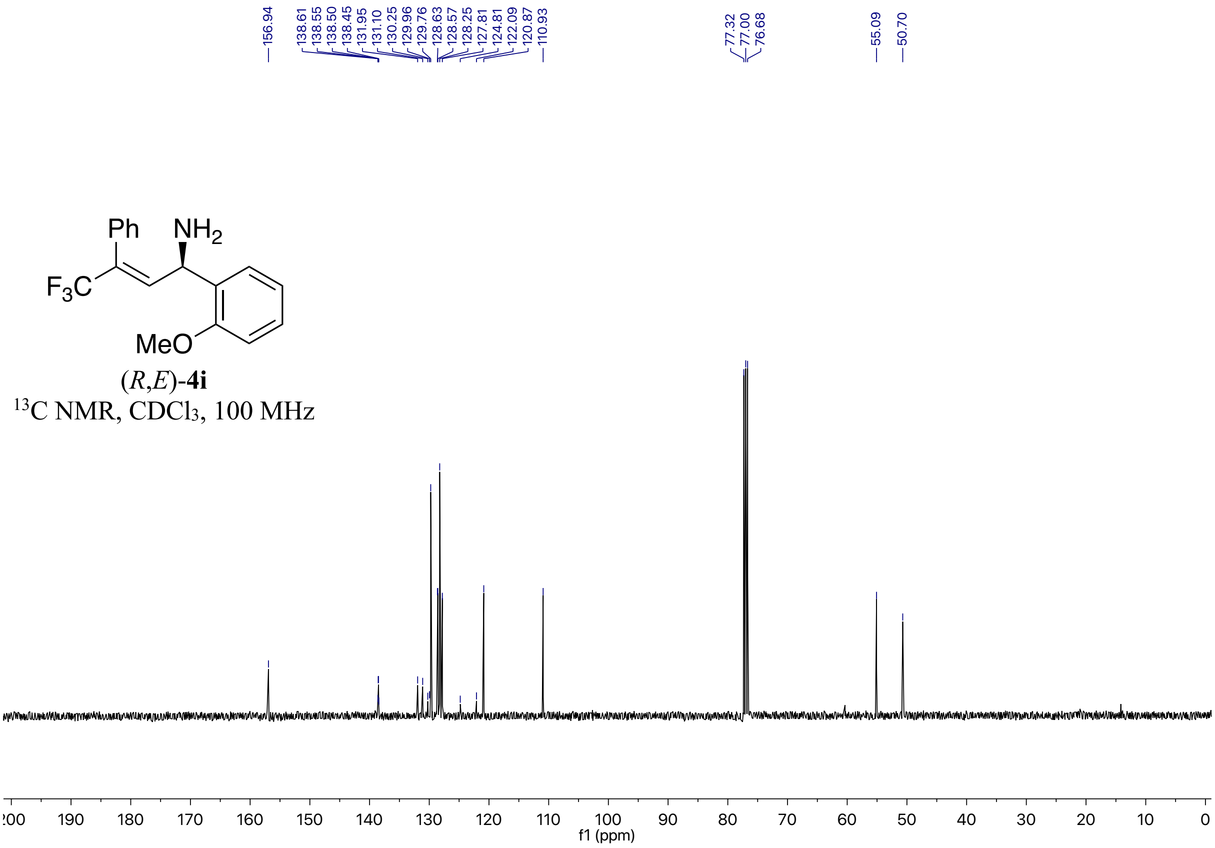

tert-Butyl (R,E)-(4,4,4-trifluoro-1-(2-methoxyphenyl)-3-phenylbut-2-en-1-yl)carbamate (4i')

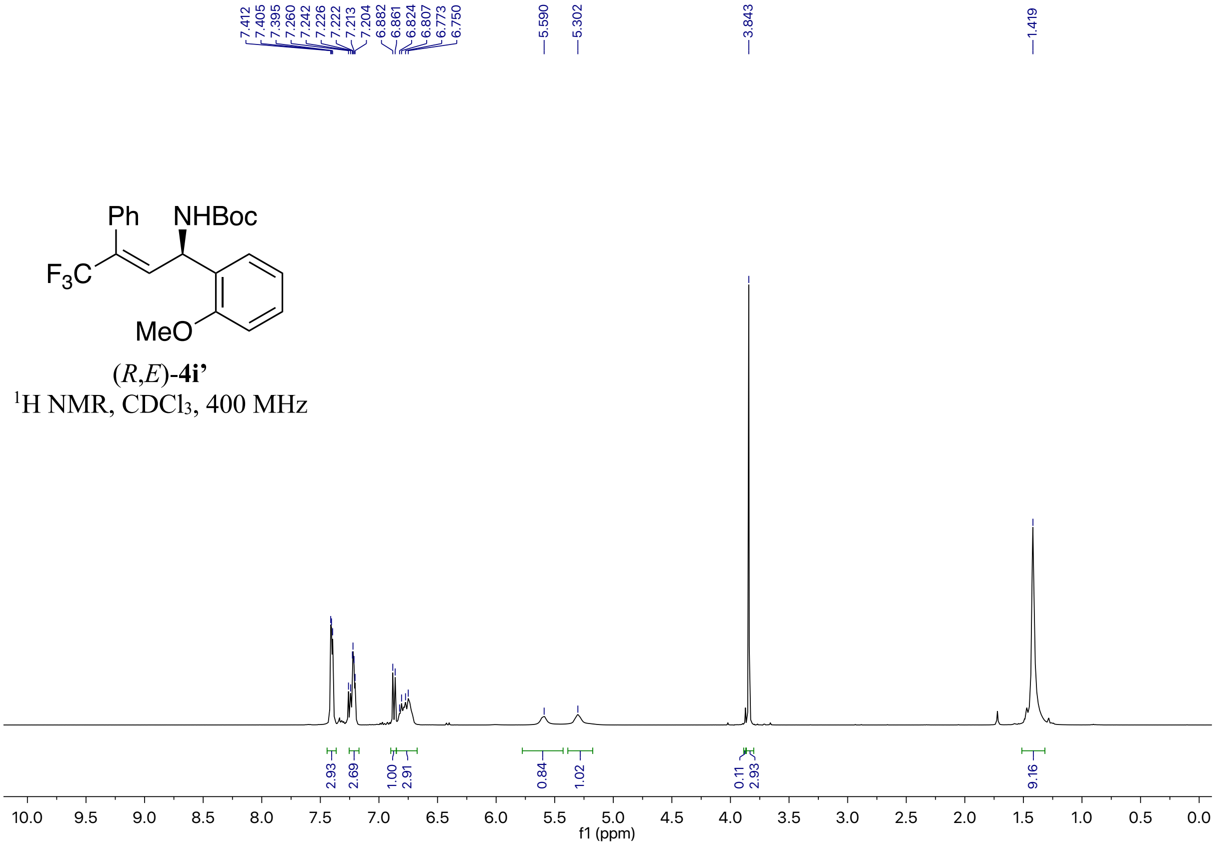

(*R*,*E*)-**4i'**
^{1}H NMR, CDCl$_3$, 400 MHz

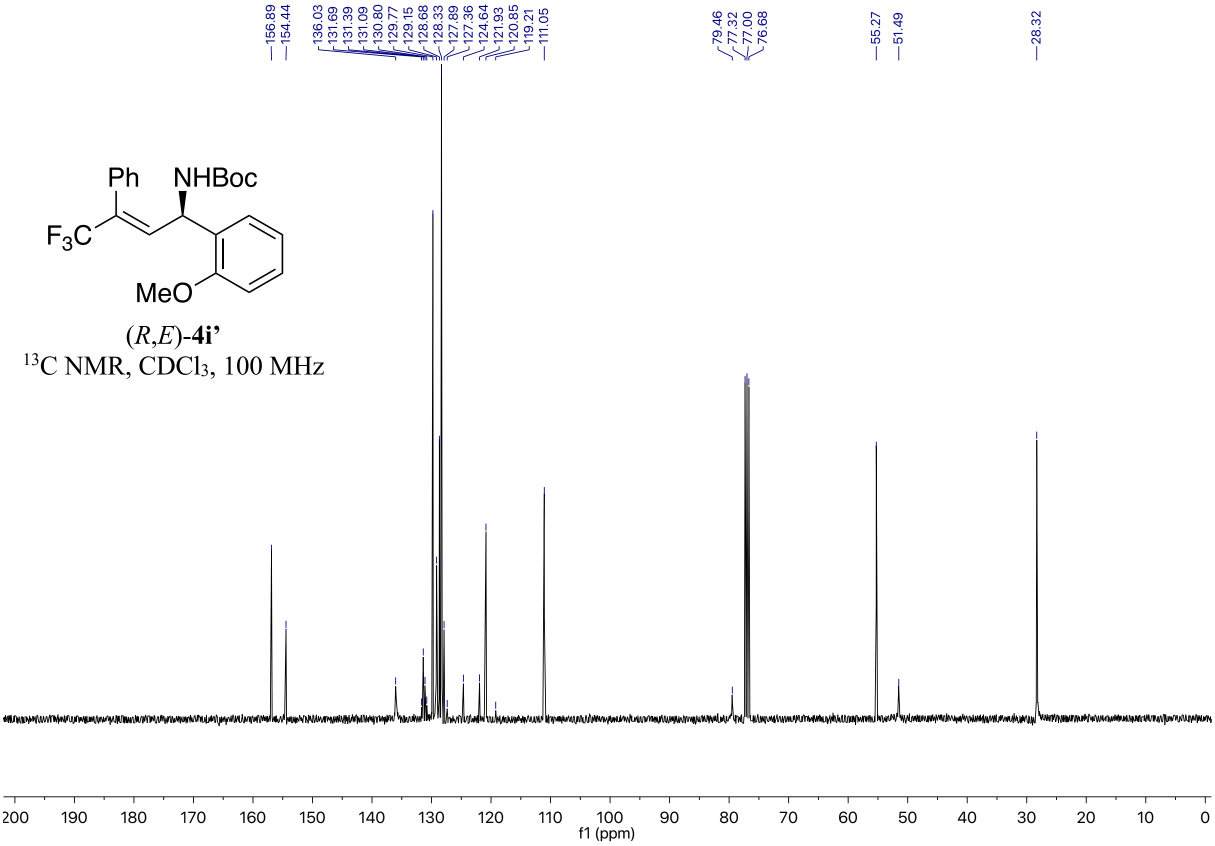

(*R*,*E*)-**4i'**
^{13}C NMR, CDCl$_3$, 100 MHz

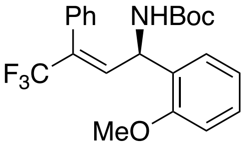

(***R***,***E***)-**4i'**

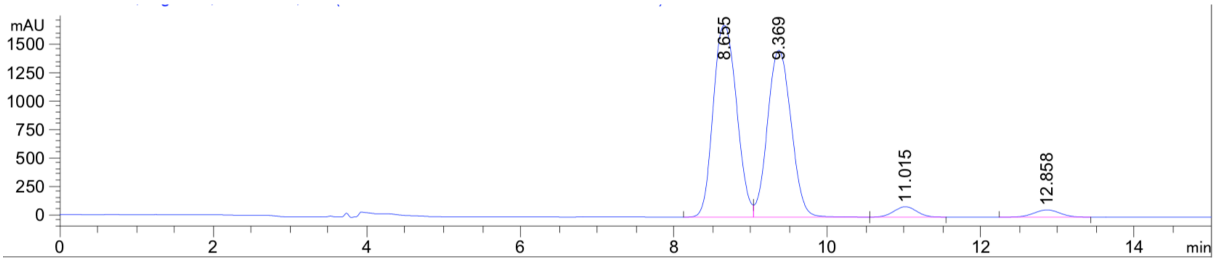

Peak #	RetTime [min]	Type	Width [min]	Area [mAU*s]	Height [mAU]	Area %
1	8.655	BV	0.3412	3.56850e4	1683.90942	50.6715
2	9.369	VB	0.3437	3.13718e4	1465.68506	44.5469
3	11.015	BV	0.3274	1889.33801	90.56397	2.6828
4	12.858	BB	0.3687	1478.06836	62.77741	2.0988

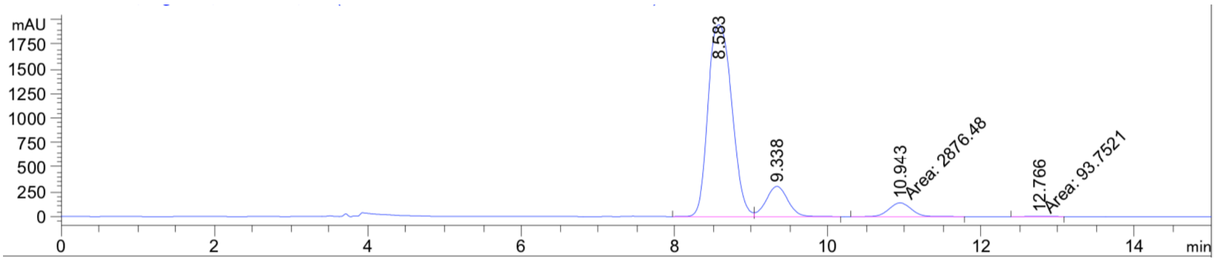

Peak #	RetTime [min]	Type	Width [min]	Area [mAU*s]	Height [mAU]	Area %
1	8.583	BV	0.3468	4.19100e4	1949.85486	82.3745
2	9.338	VB	0.3039	5997.18994	307.02896	11.7875
3	10.943	MM	0.3415	2876.47998	140.36908	5.6537
4	12.766	MM	0.3976	93.75205	3.92988	0.1843

(*S*,*E*)-5,5,5-Trifluoro-4-phenylpent-3-en-2-amine (4j)

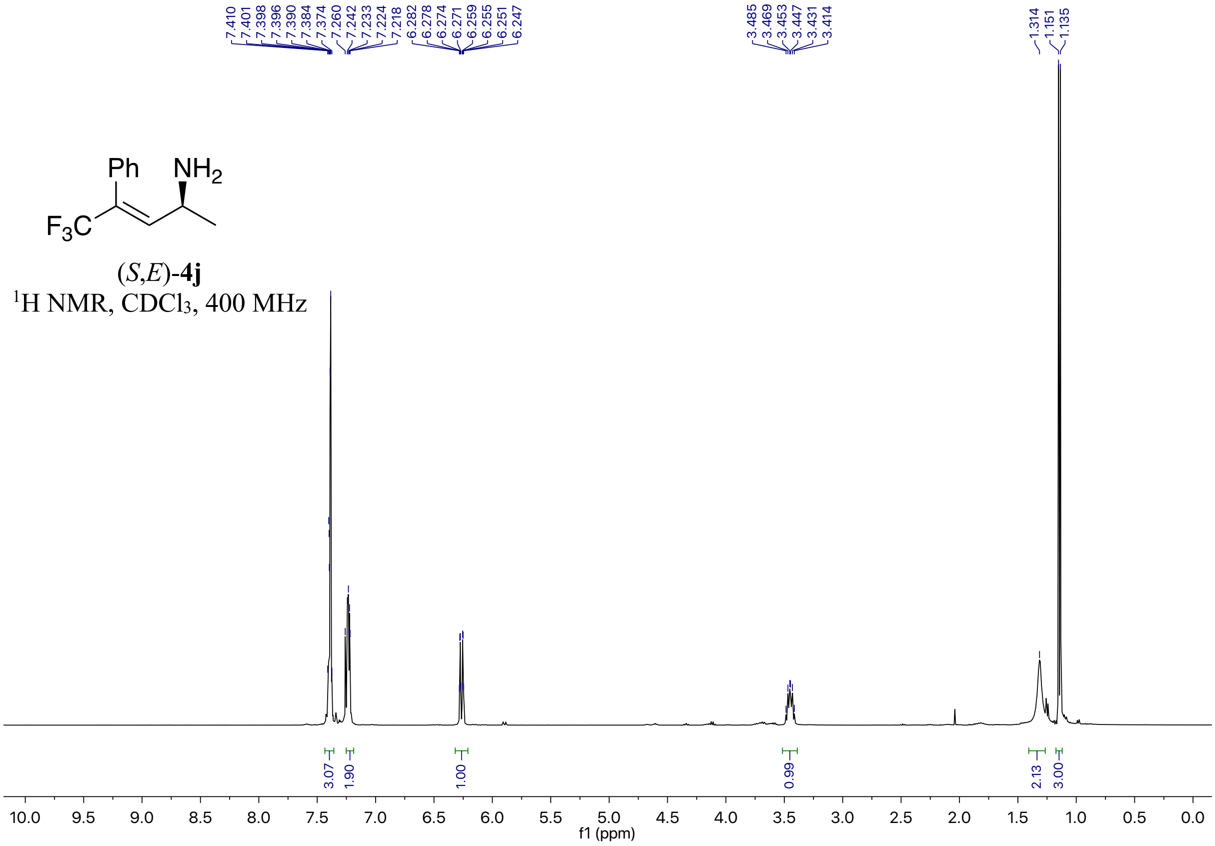

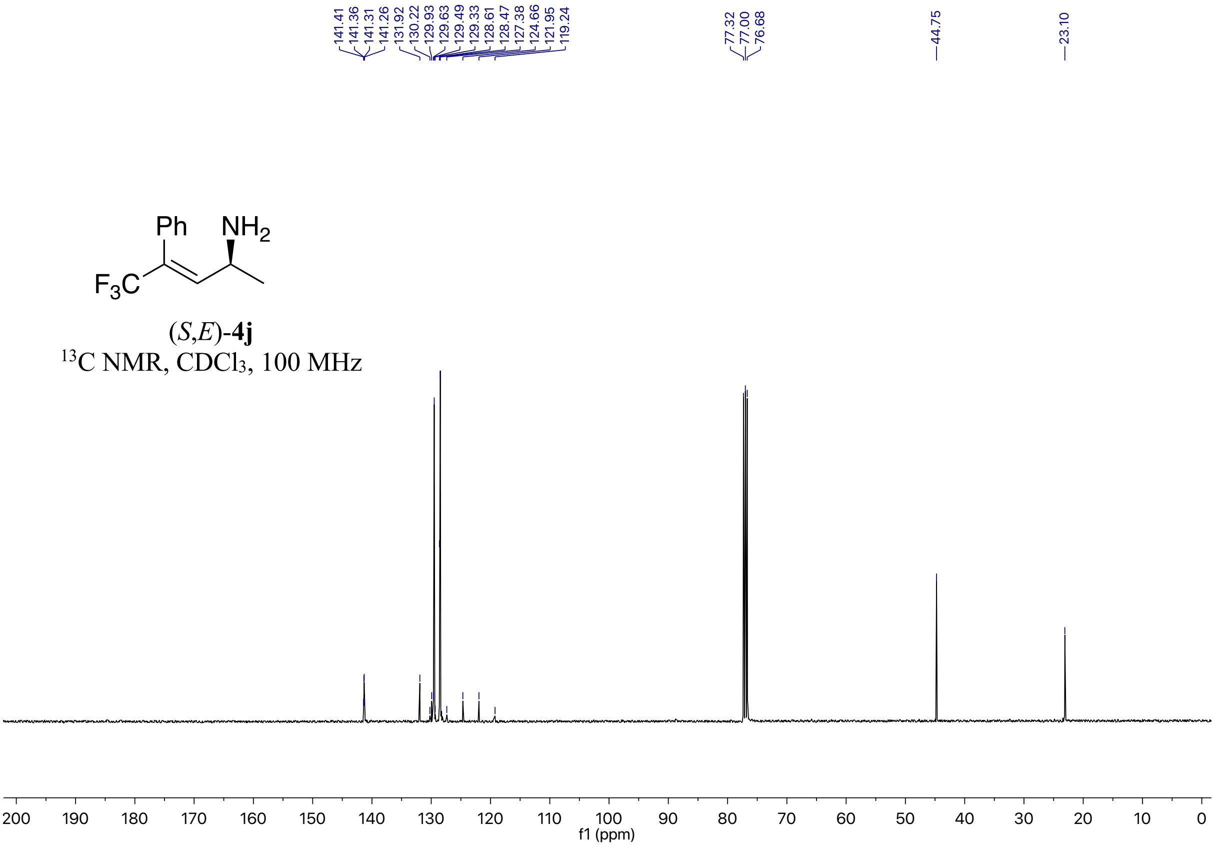

***tert*-Butyl (*S*,*E*)-(5,5,5-Trifluoro-4-phenylpent-3-en-2-yl)carbamate (4j')**

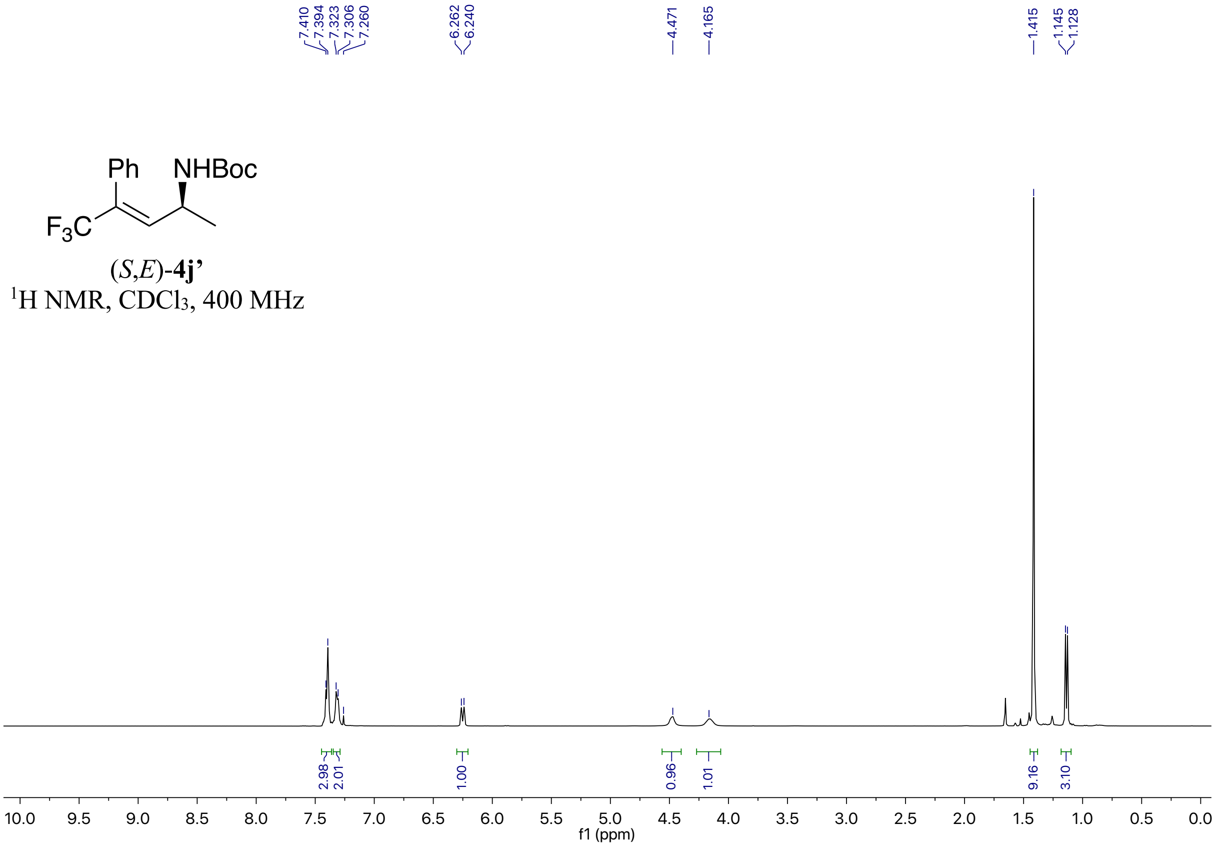

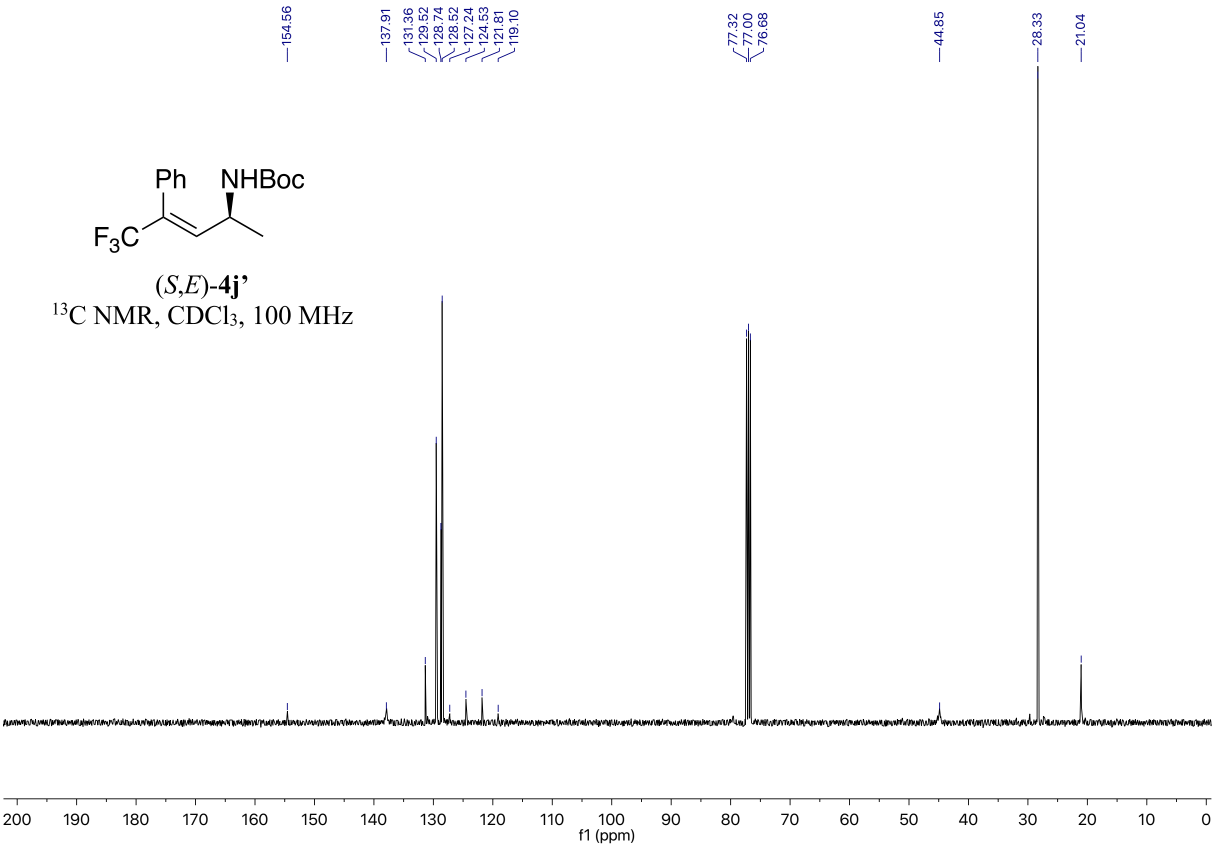

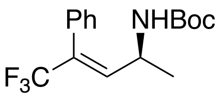

(*S*,*E*)-4j'

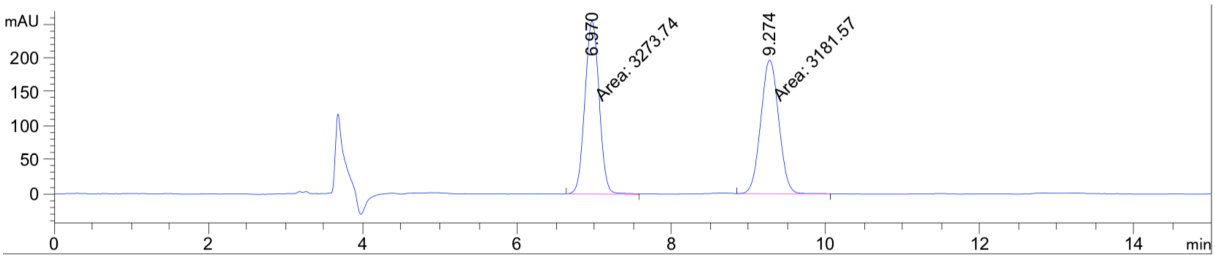

Peak #	RetTime [min]	Type	Width [min]	Area [mAU*s]	Height [mAU]	Area %
1	6.970	MM	0.2142	3273.73828	254.76917	50.7139
2	9.274	MM	0.2696	3181.57300	196.70164	49.2861

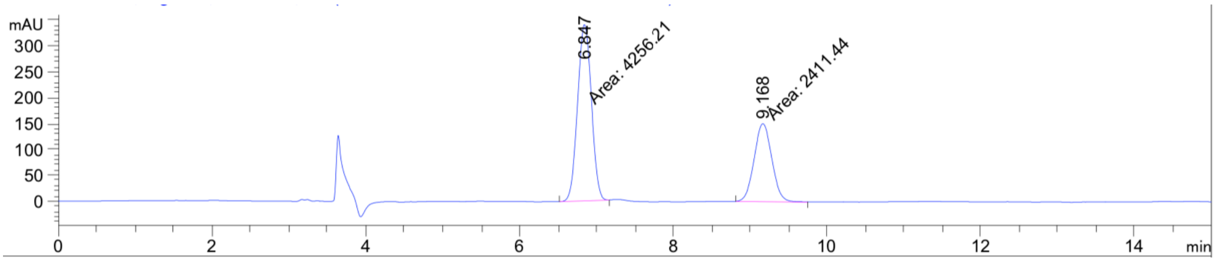

Peak #	RetTime [min]	Type	Width [min]	Area [mAU*s]	Height [mAU]	Area %
1	6.847	MM	0.2083	4256.21240	340.51993	63.8337
2	9.168	MM	0.2666	2411.43994	150.74600	36.1663

(E)-4,4,4-Trifluoro-3-phenylbut-2-en-1-amine (4k)

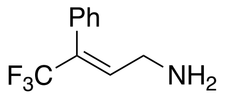

(R,E)-**4k**

^{1}H NMR, $CDCl_3$, 400 MHz

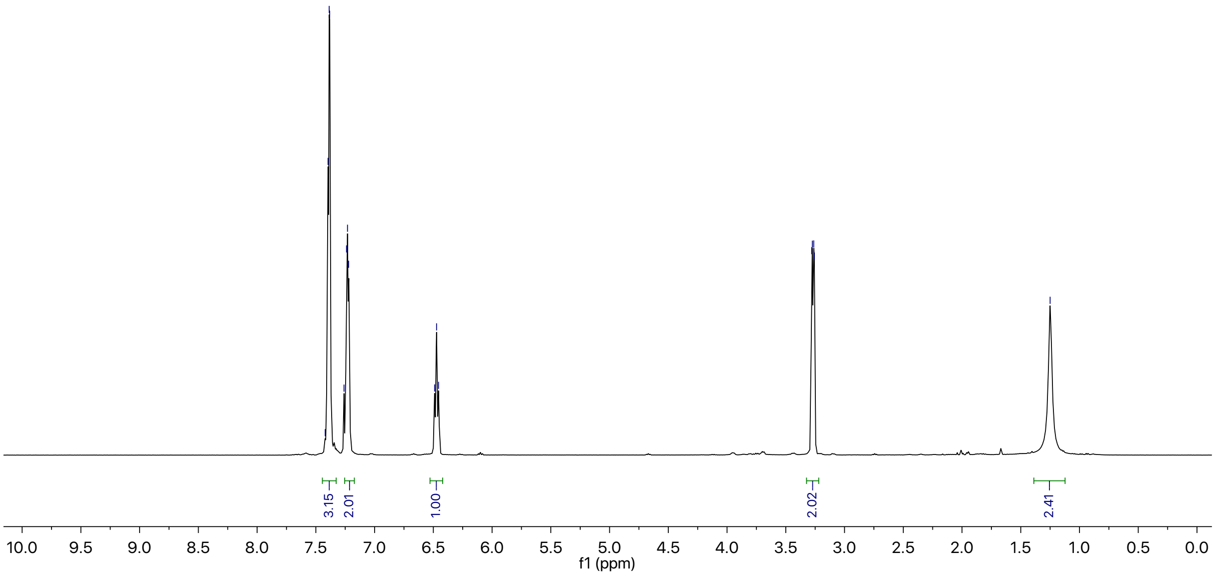

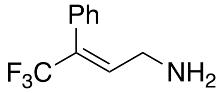

(R,E)-**4k**

^{13}C NMR, $CDCl_3$, 100 MHz

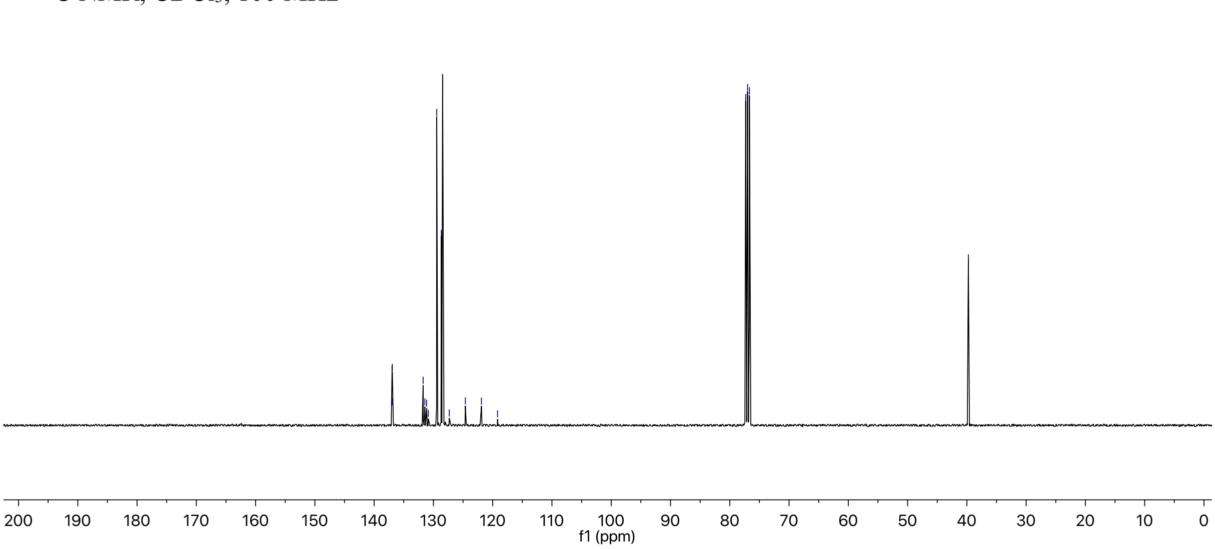

(*R*,*E*)-4,4,4-Trifluoro-1-phenyl-3-(*p*-tolyl)but-2-en-1-amine (4l)

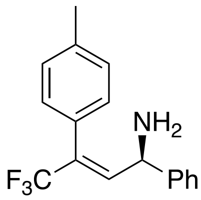

(*R*,*E*)-**4l**

^{1}H NMR, $CDCl_3$, 400 MHz

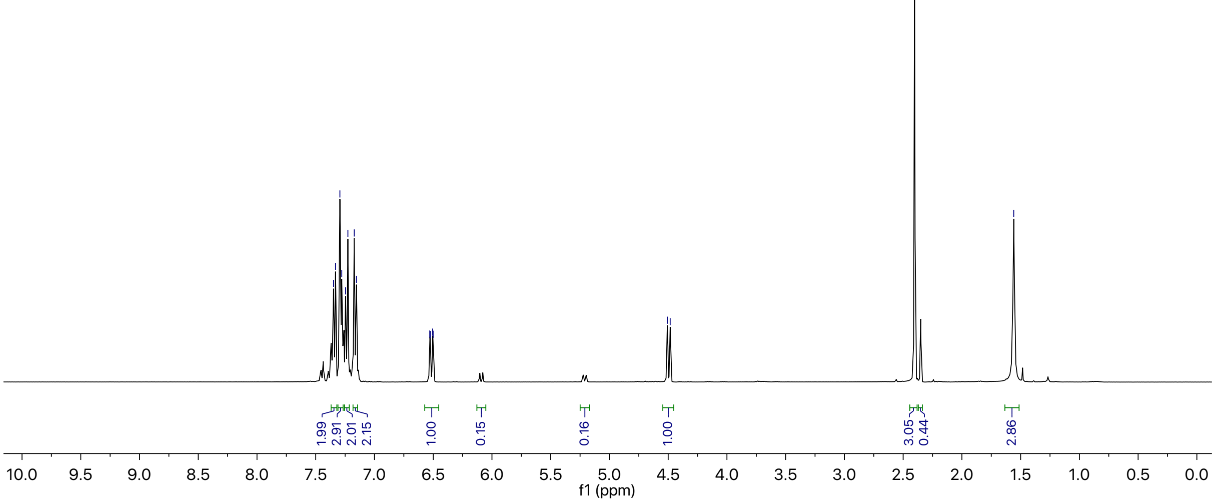

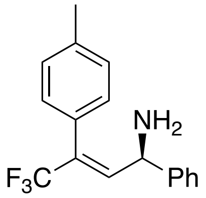

(*R*,*E*)-**4l**

^{13}C NMR, $CDCl_3$, 100 MHz

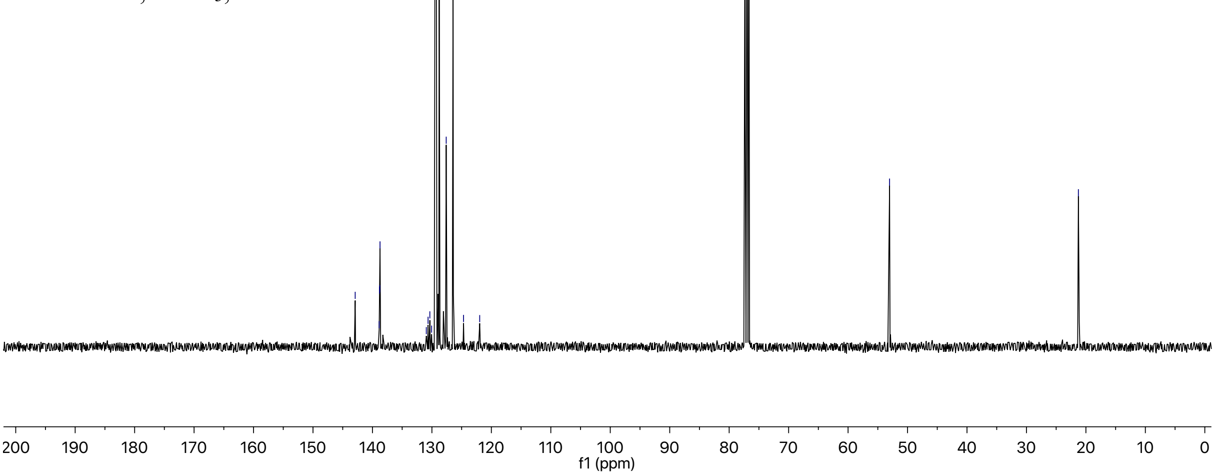

***tert*-Butyl (*R*,*E*)-(4,4,4-trifluoro-1-phenyl-3-(*p*-tolyl)but-2-en-1-yl)carbamate (4l')**

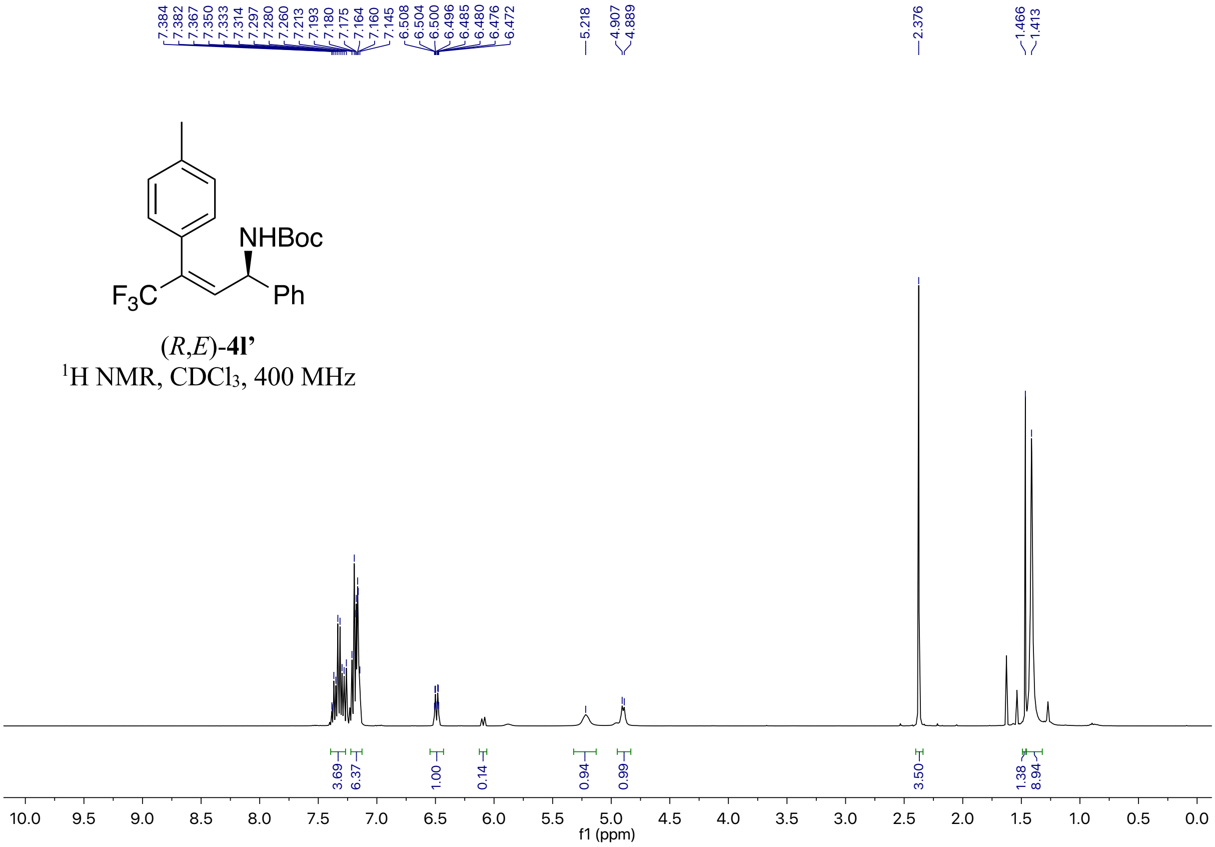

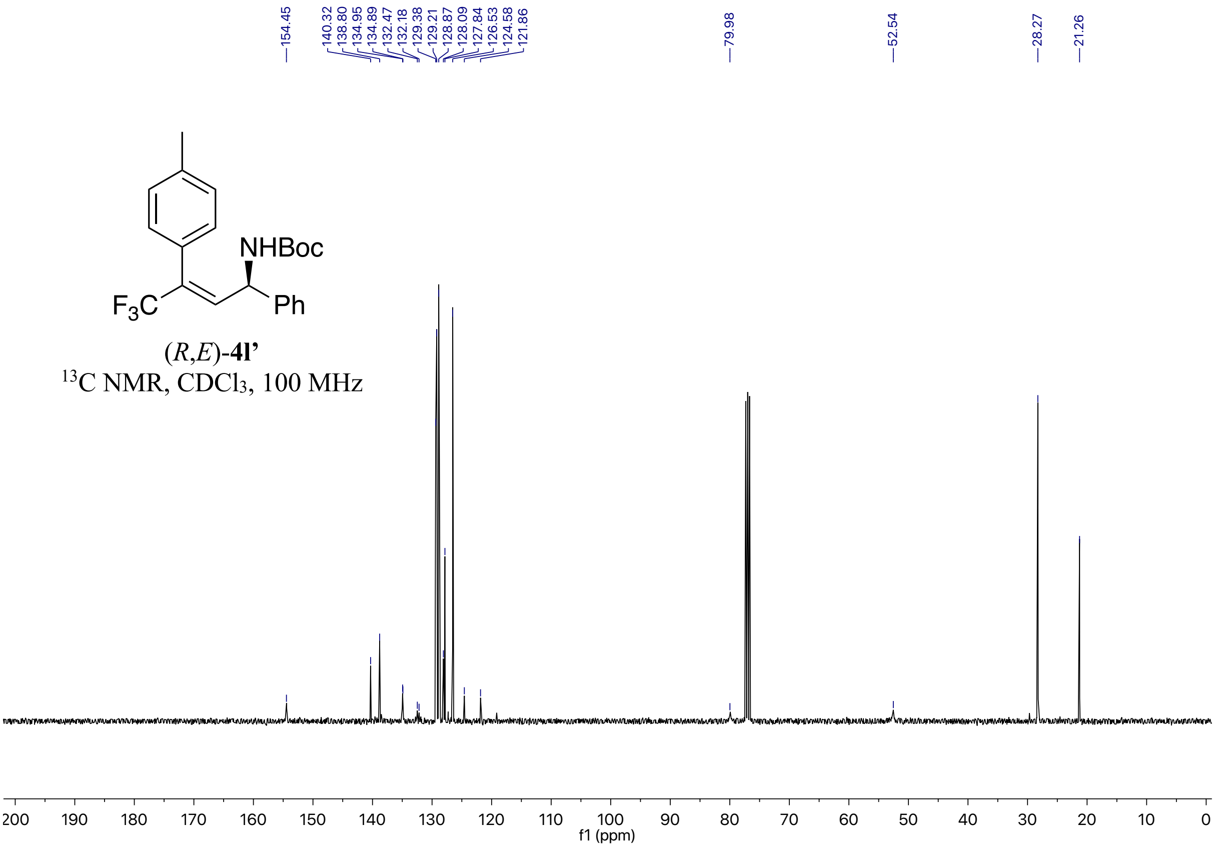

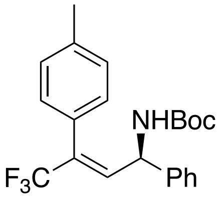

(*R*,*E*)-4l'

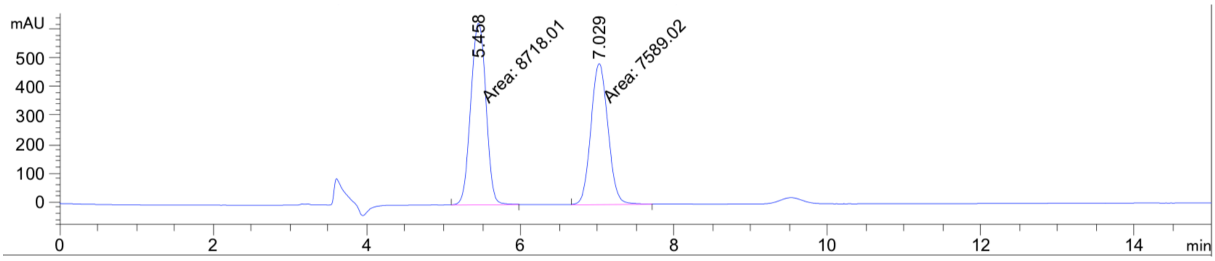

Peak #	RetTime [min]	Type	Width [min]	Area [mAU*s]	Height [mAU]	Area %
1	5.458	MM	0.2300	8718.00586	631.83197	53.4617
2	7.029	MM	0.2578	7589.01953	490.53333	46.5383

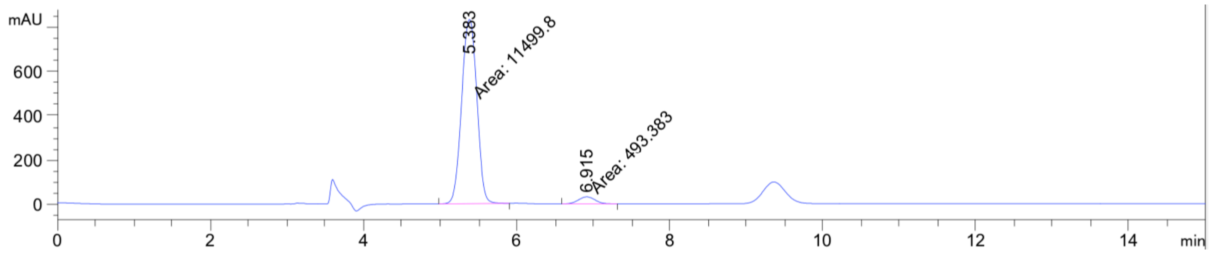

Peak #	RetTime [min]	Type	Width [min]	Area [mAU*s]	Height [mAU]	Area %
1	5.383	MM	0.2299	1.14998e4	833.57446	95.8861
2	6.915	MM	0.2567	493.38300	32.03526	4.1139

(*R*,*E*)-4,4,4-Trifluoro-3-(4-methoxyphenyl)-1-phenylbut-2-en-1-amine (4m)

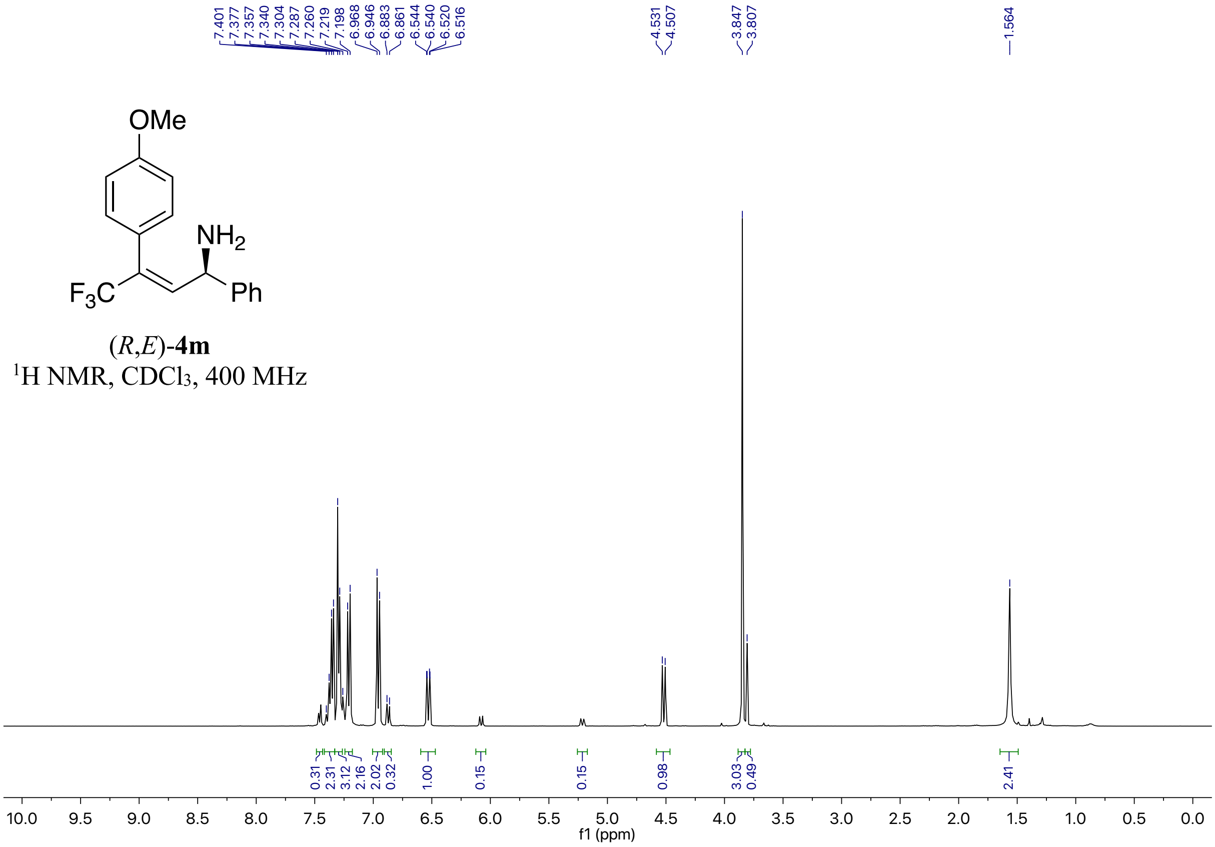

(*R*,*E*)-**4m**
^{1}H NMR, $CDCl_3$, 400 MHz

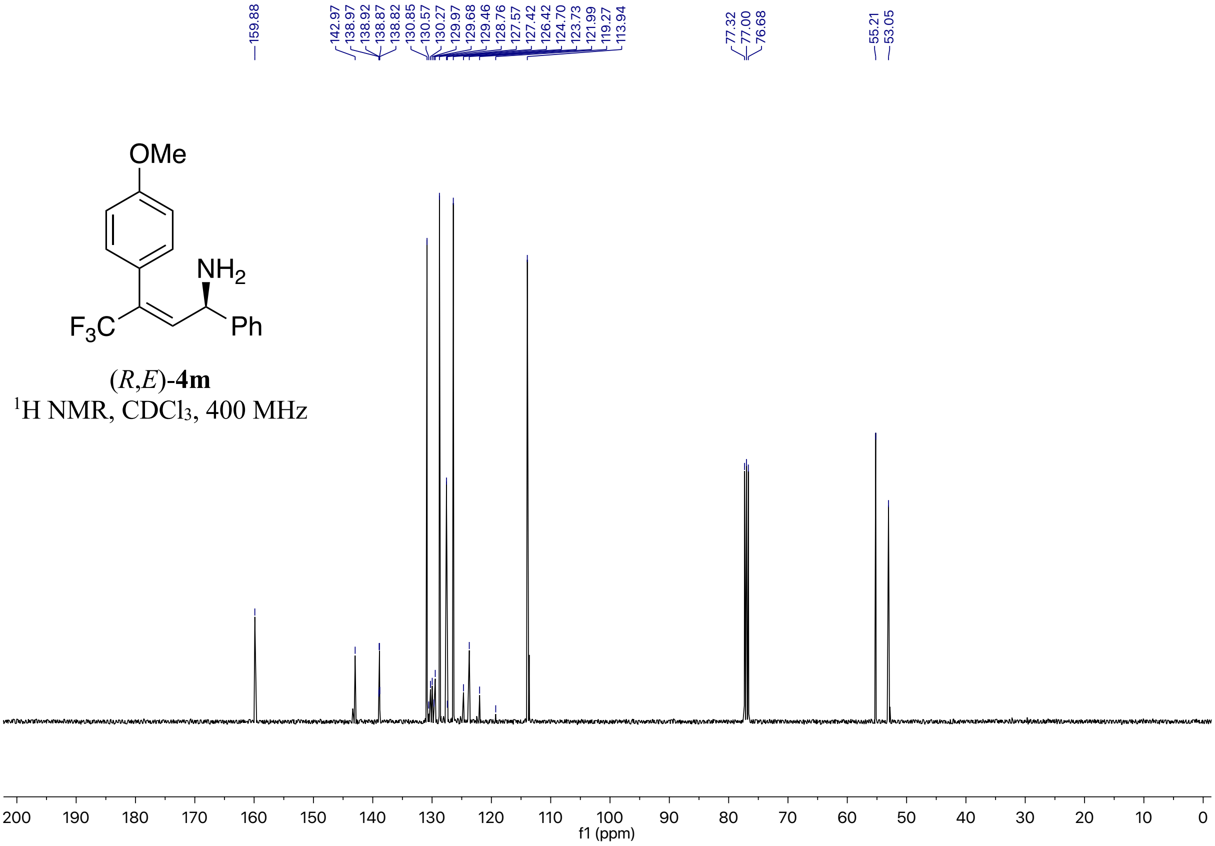

(*R*,*E*)-**4m**
^{1}H NMR, $CDCl_3$, 400 MHz

tert-Butyl (_R_,_E_)-(4,4,4-trifluoro-3-(4-methoxyphenyl)-1-phenylbut-2-en-1-yl)carbamate (4m')

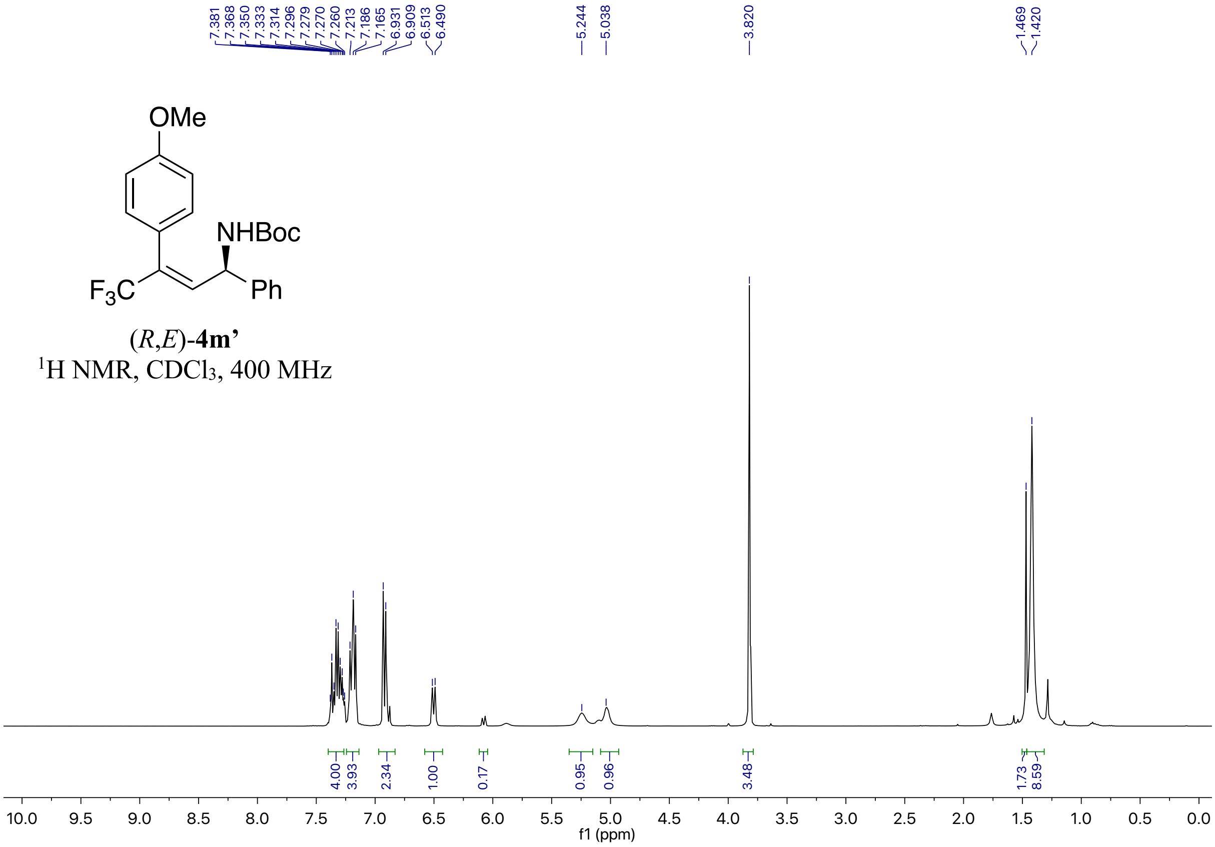

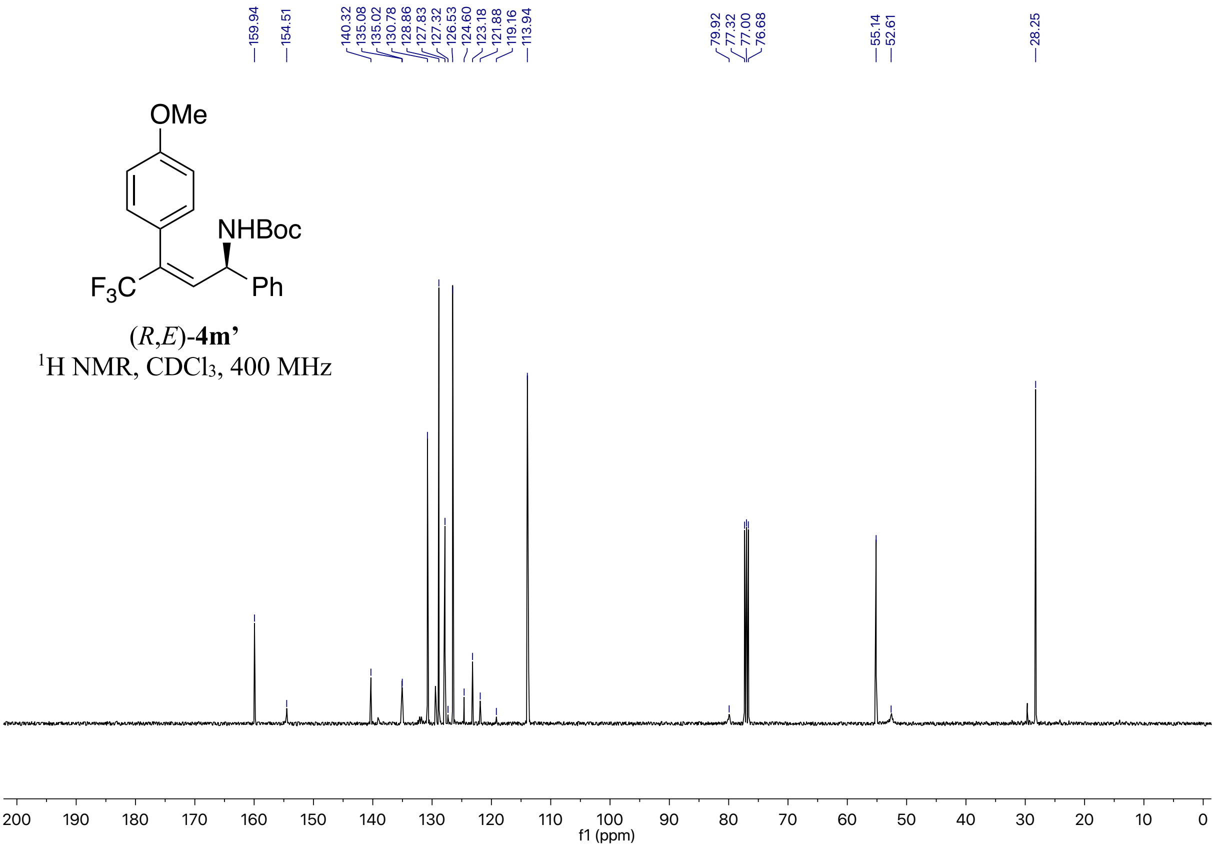

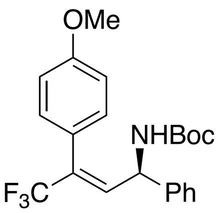

(*R*,*E*)-4m'

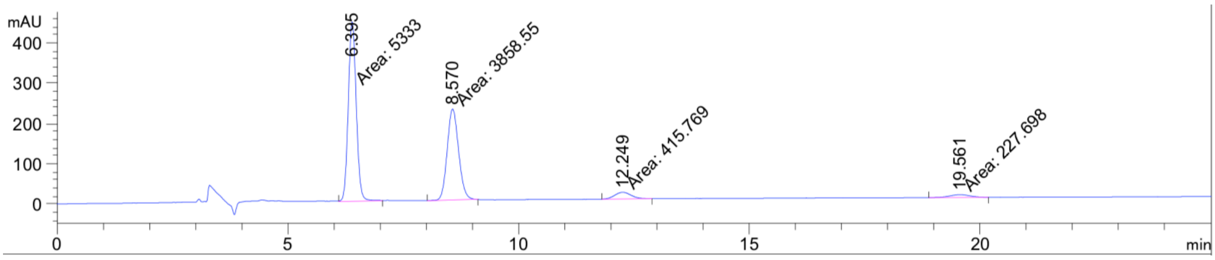

Peak #	RetTime [min]	Type	Width [min]	Area [mAU*s]	Height [mAU]	Area %
1	6.395	MM	0.2002	5333.00146	444.01639	54.2246
2	8.570	MM	0.2857	3858.55322	225.07327	39.2328
3	12.249	MM	0.4176	415.76917	16.59385	4.2274
4	19.561	MM	0.5349	227.69830	7.09520	2.3152

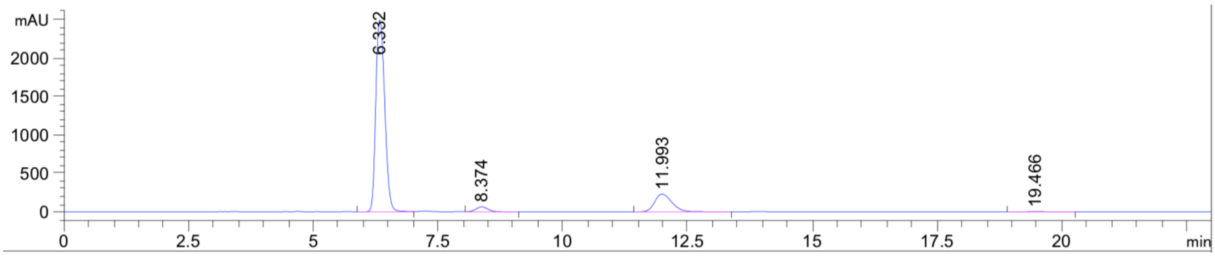

Peak #	RetTime [min]	Type	Width [min]	Area [mAU*s]	Height [mAU]	Area %
1	6.332	BV	0.2001	3.11489e4	2487.38428	82.3838
2	8.374	VB	0.2597	1075.94678	64.13140	2.8457
3	11.993	BB	0.3727	5527.08301	229.74226	14.6182
4	19.466	VB	0.4127	57.56136	1.72752	0.1522

(*R*,*E*)-4,4,4-Trifluoro-1-phenyl-3-(4-(trifluoromethyl)phenyl)but-2-en-1-amine (4n)

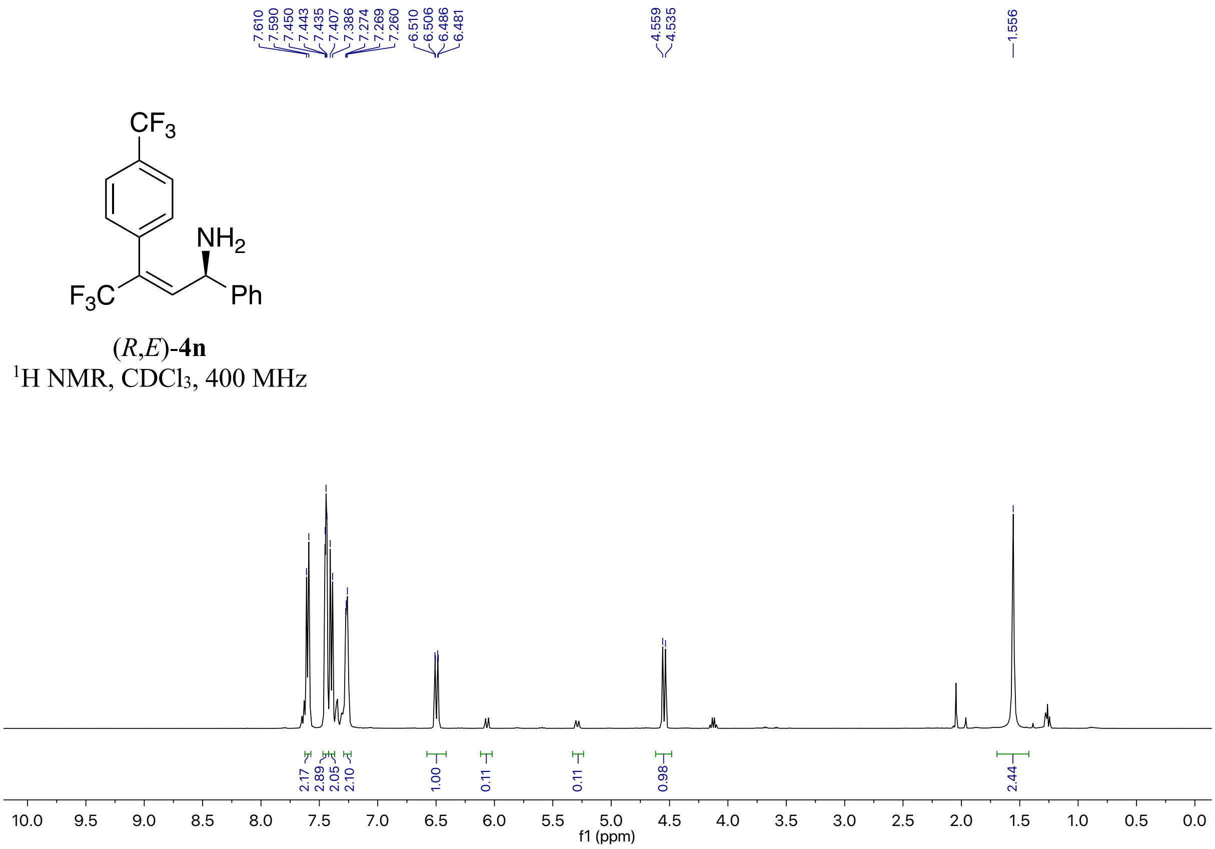

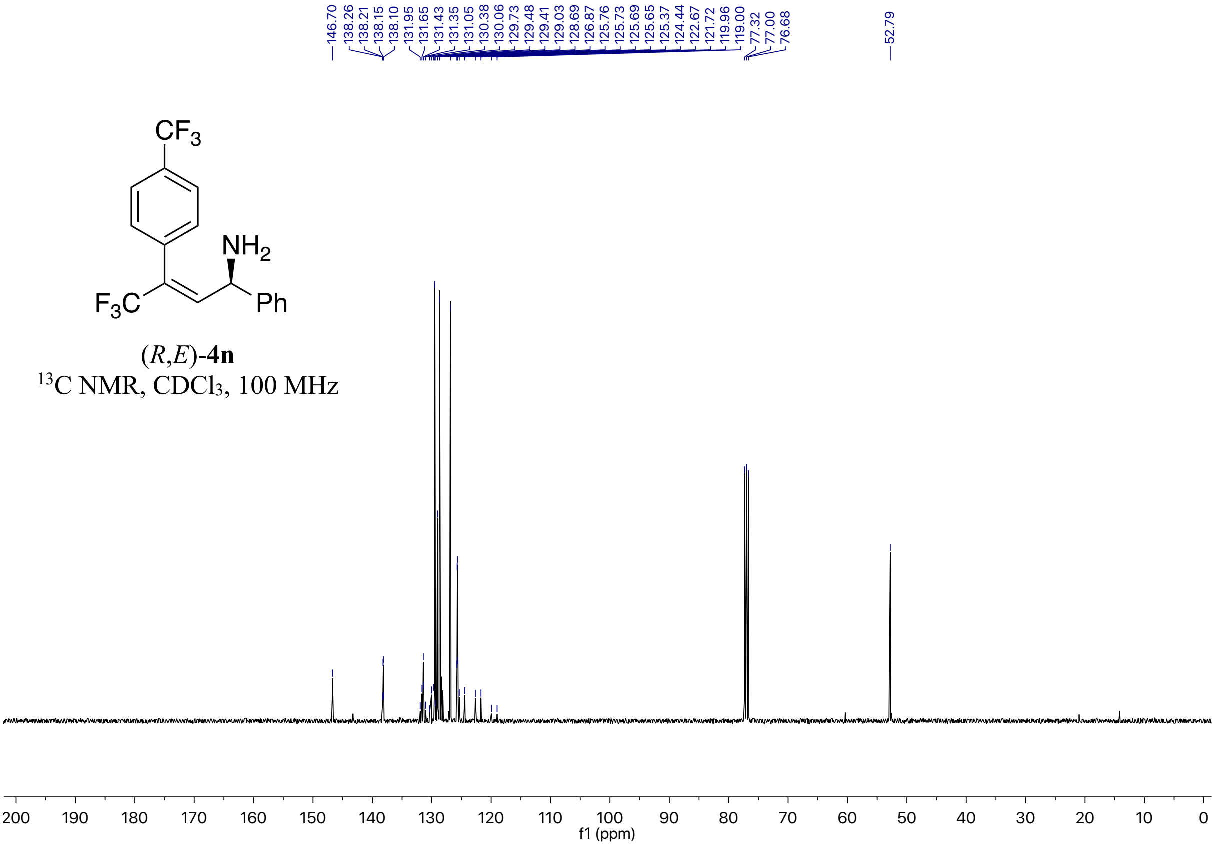

tert-Butyl (_R_,_E_)-(4,4,4-trifluoro-1-phenyl-3-(4-(trifluoromethyl)phenyl)but-2-en-1-yl)carbamate (4n')

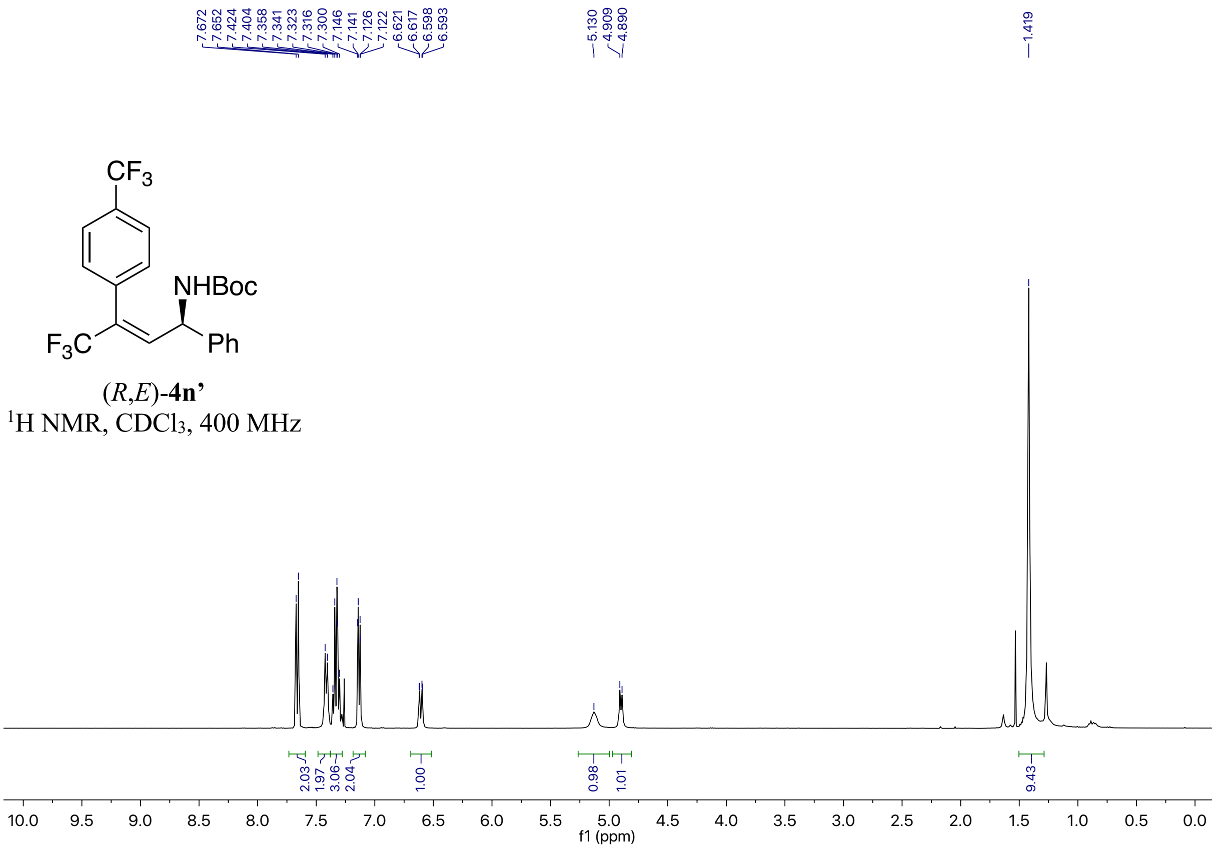

(_R_,_E_)-**4n'**
^{1}H NMR, $CDCl_3$, 400 MHz

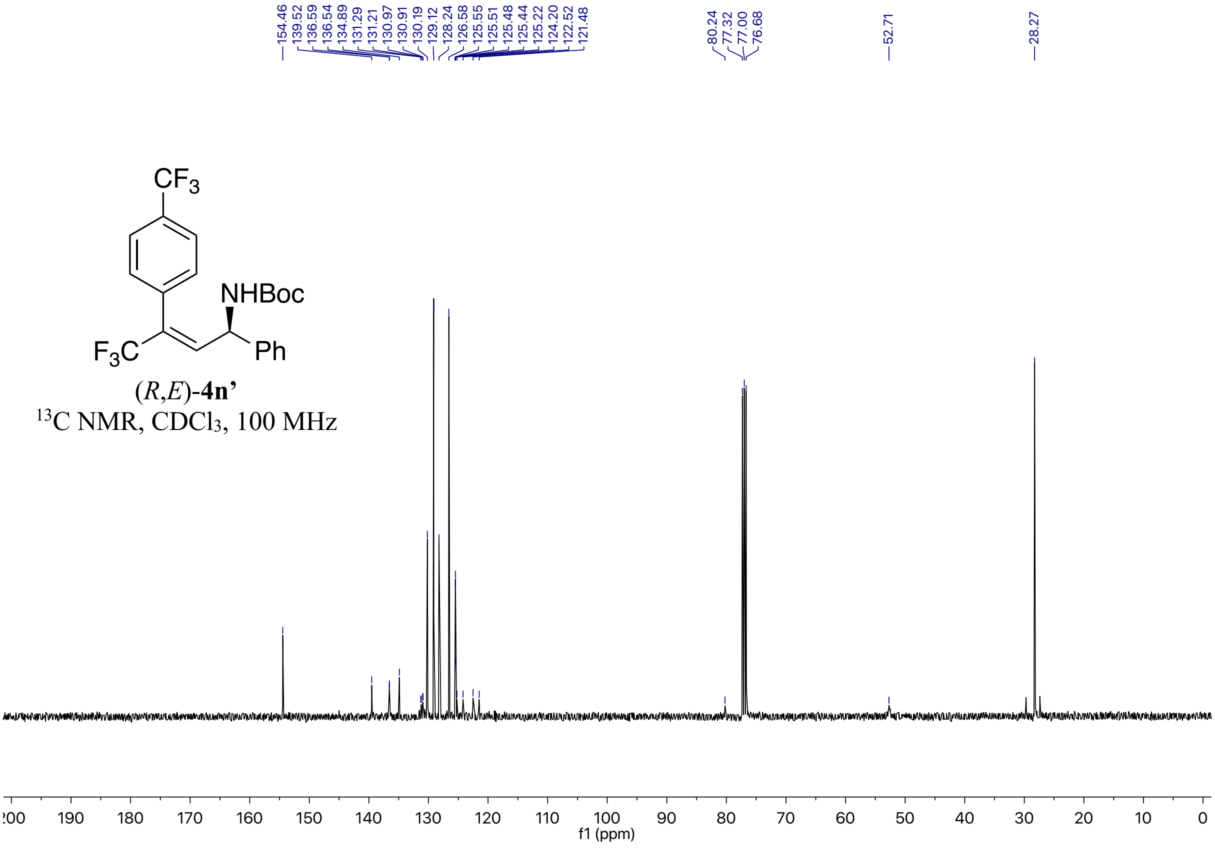

(_R_,_E_)-**4n'**
^{13}C NMR, $CDCl_3$, 100 MHz

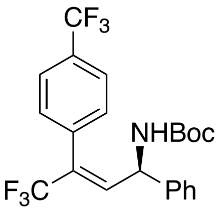

(*R*,*E*)-4n'

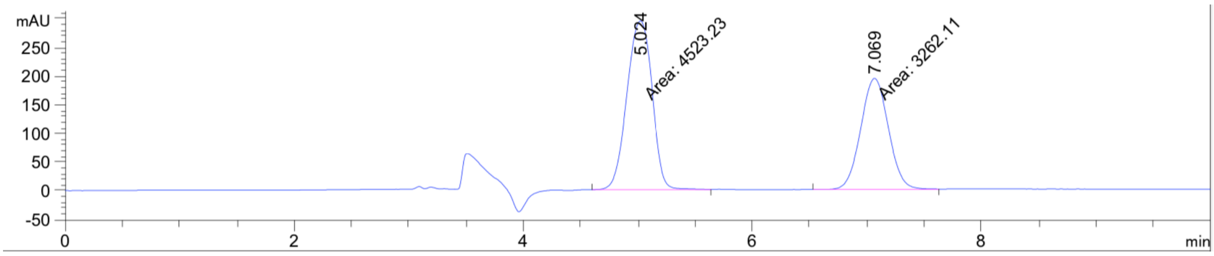

Peak #	RetTime [min]	Type	Width [min]	Area [mAU*s]	Height [mAU]	Area %
1	5.024	MM	0.2559	4523.22705	294.54364	58.0993
2	7.069	MM	0.2803	3262.11377	193.94978	41.9007

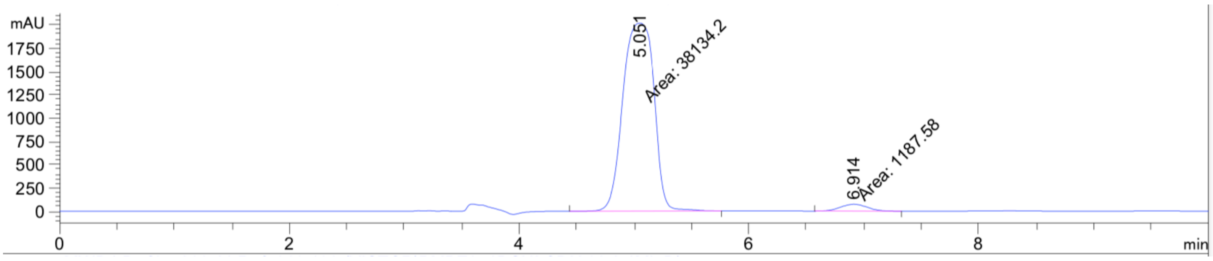

Peak #	RetTime [min]	Type	Width [min]	Area [mAU*s]	Height [mAU]	Area %
1	5.051	MM	0.3152	3.81342e4	2016.45679	96.9799
2	6.914	MM	0.2698	1187.57690	73.35021	3.0201

(*R*,*E*)-4,4,4-Trifluoro-1-phenyl-3-(*m*-tolyl)but-2-en-1-amine (4o)

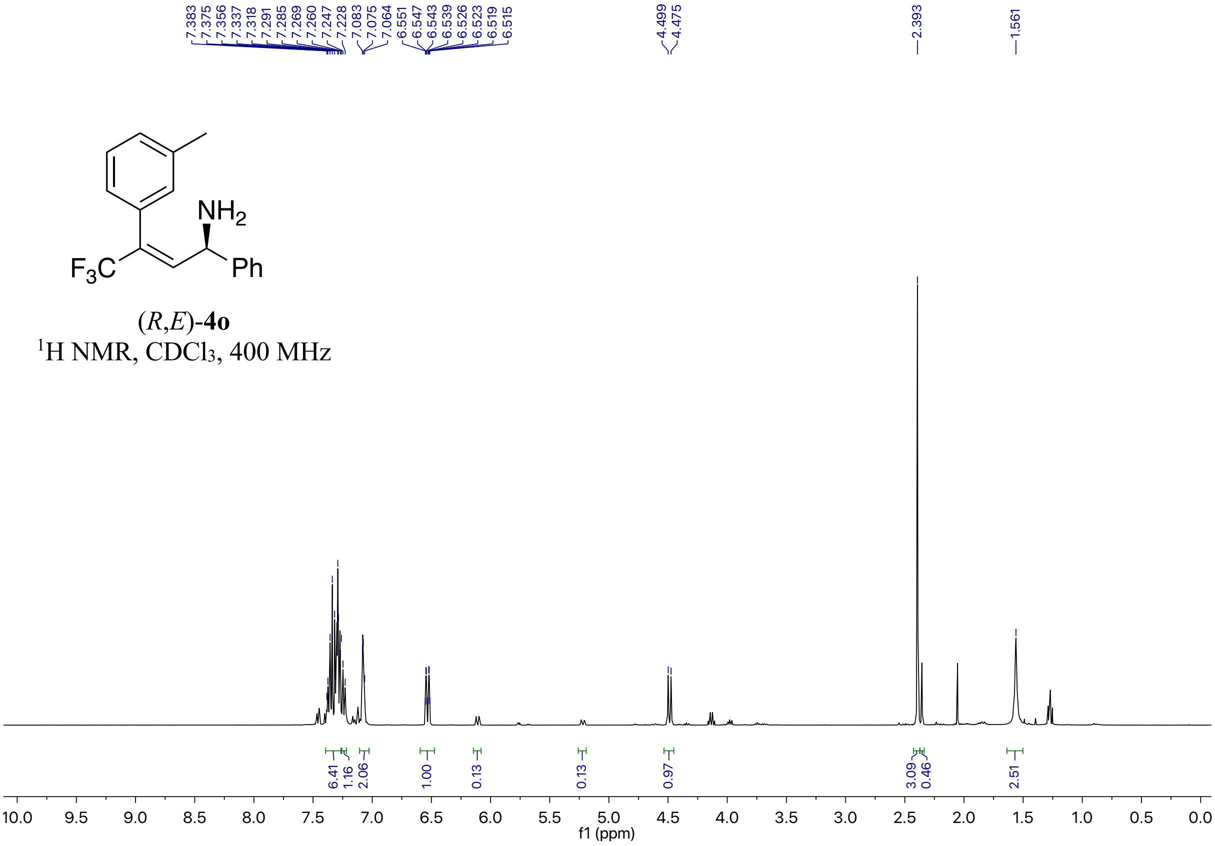

(*R*,*E*)-**4o**
^{1}H NMR, $CDCl_3$, 400 MHz

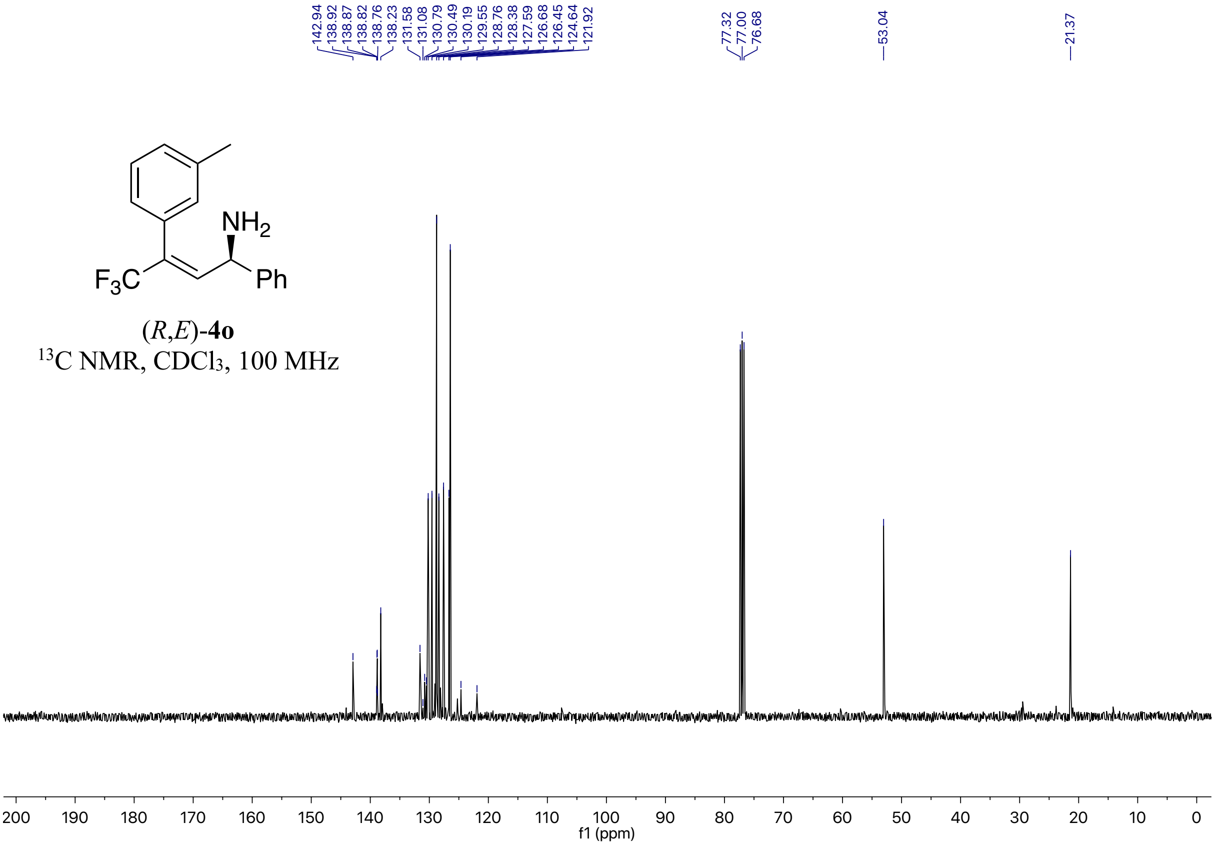

(*R*,*E*)-**4o**
^{13}C NMR, $CDCl_3$, 100 MHz

***tert*-Butyl (*R*,*E*)-(4,4,4-trifluoro-1-phenyl-3-(*m*-tolyl)but-2-en-1-yl)carbamate (4o')**

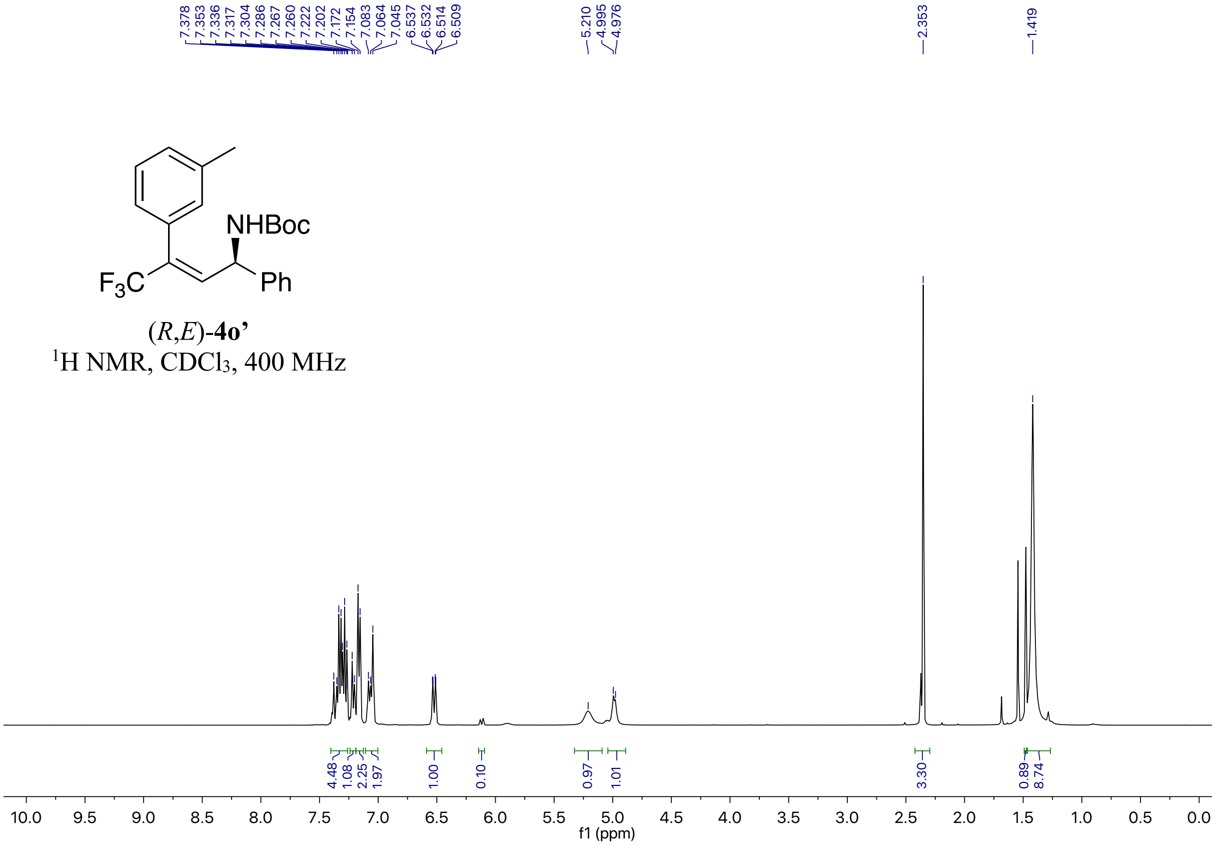

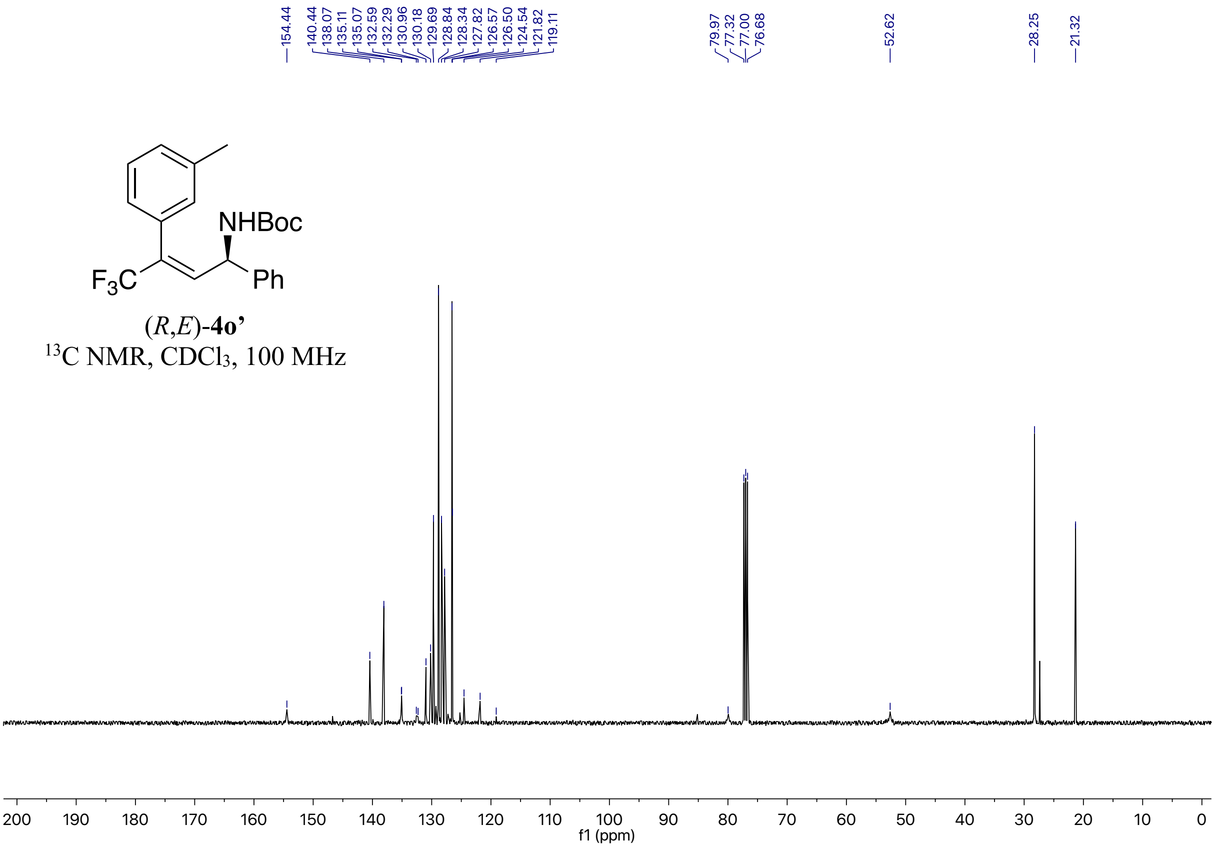

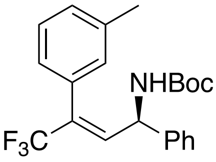

(*R*,*E*)-4o'

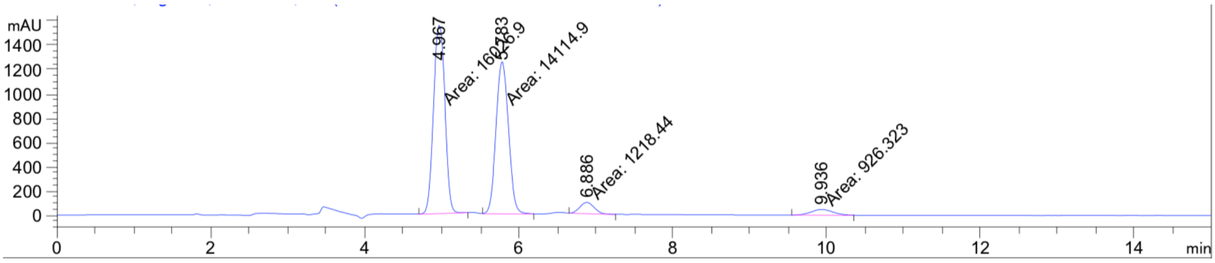

Peak #	RetTime [min]	Type	Width [min]	Area [mAU*s]	Height [mAU]	Area %
1	4.967	MM	0.1726	1.60269e4	1547.21997	49.6395
2	5.783	MM	0.1881	1.41149e4	1250.43481	43.7176
3	6.886	MM	0.2194	1218.43604	92.54398	3.7738
4	9.936	MM	0.3304	926.32281	46.72501	2.8691

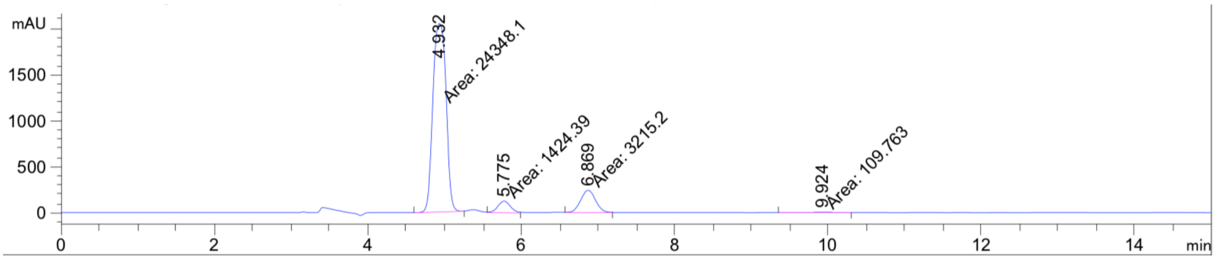

Peak #	RetTime [min]	Type	Width [min]	Area [mAU*s]	Height [mAU]	Area %
1	4.932	MM	0.1983	2.43481e4	2046.01196	83.6777
2	5.775	MM	0.1894	1424.39233	125.31697	4.8953
3	6.869	MM	0.2233	3215.20313	239.96571	11.0498
4	9.924	MM	0.4697	109.76331	3.89465	0.3772

(*R*,*Z*)-4,4,4-Trifluoro-1-phenyl-3-(thiophen-2-yl)but-2-en-1-amine (4p)

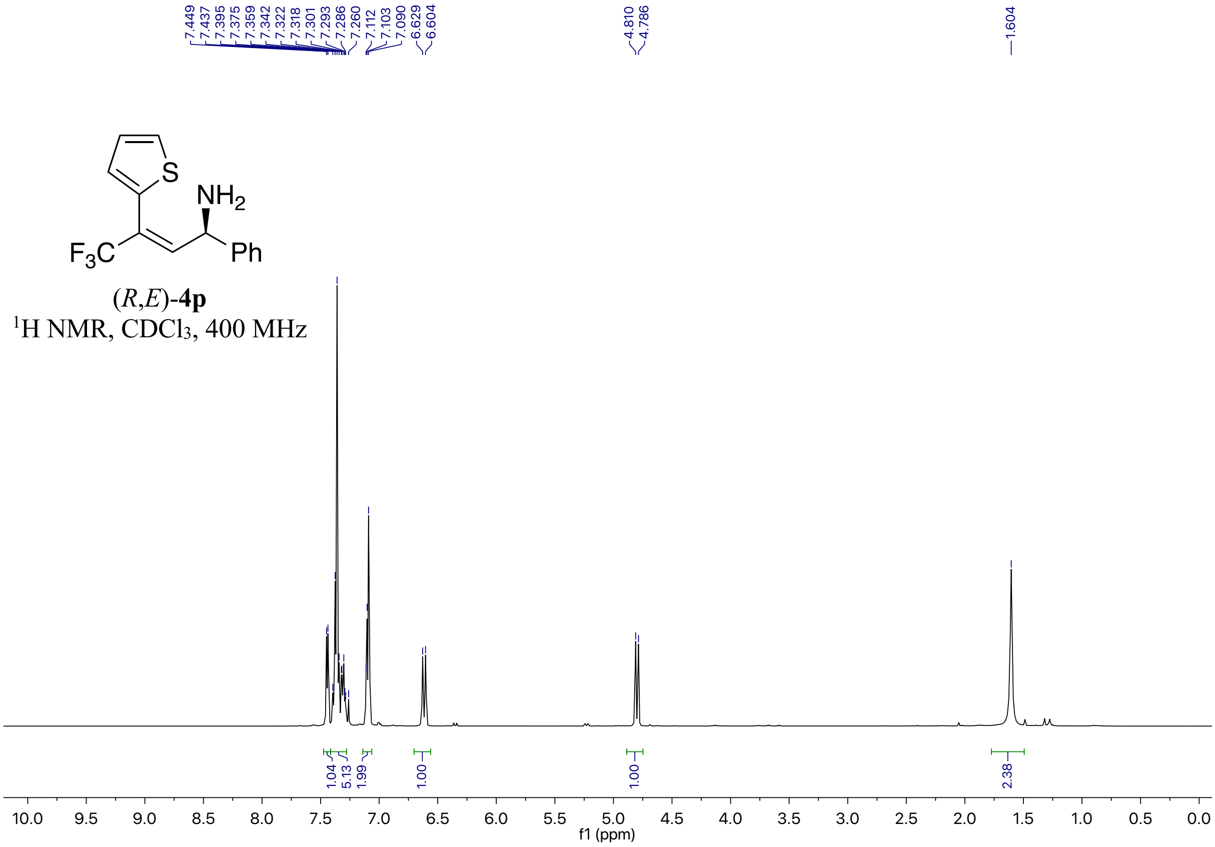

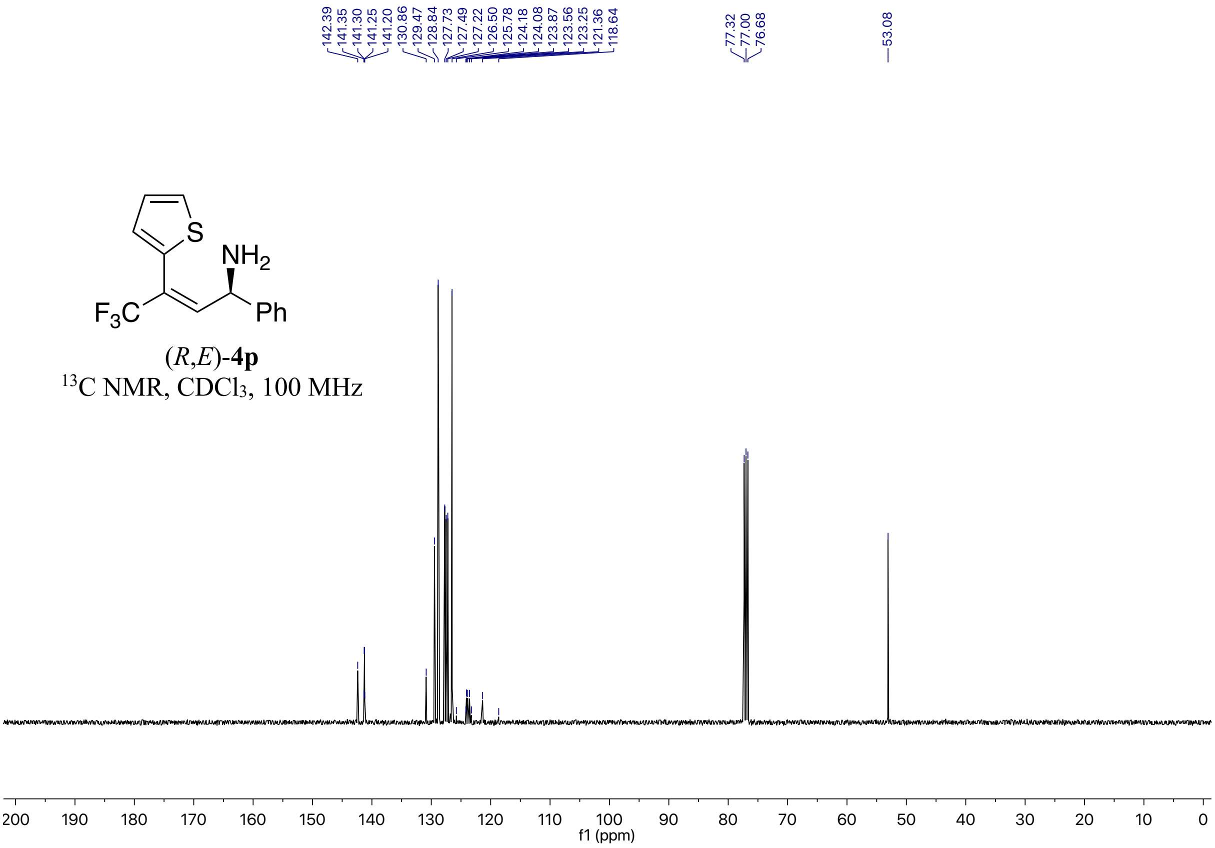

***tert*-Butyl (*R*,*Z*)-(4,4,4-trifluoro-1-phenyl-3-(thiophen-2-yl)but-2-en-1-yl)carbamate (4p')**

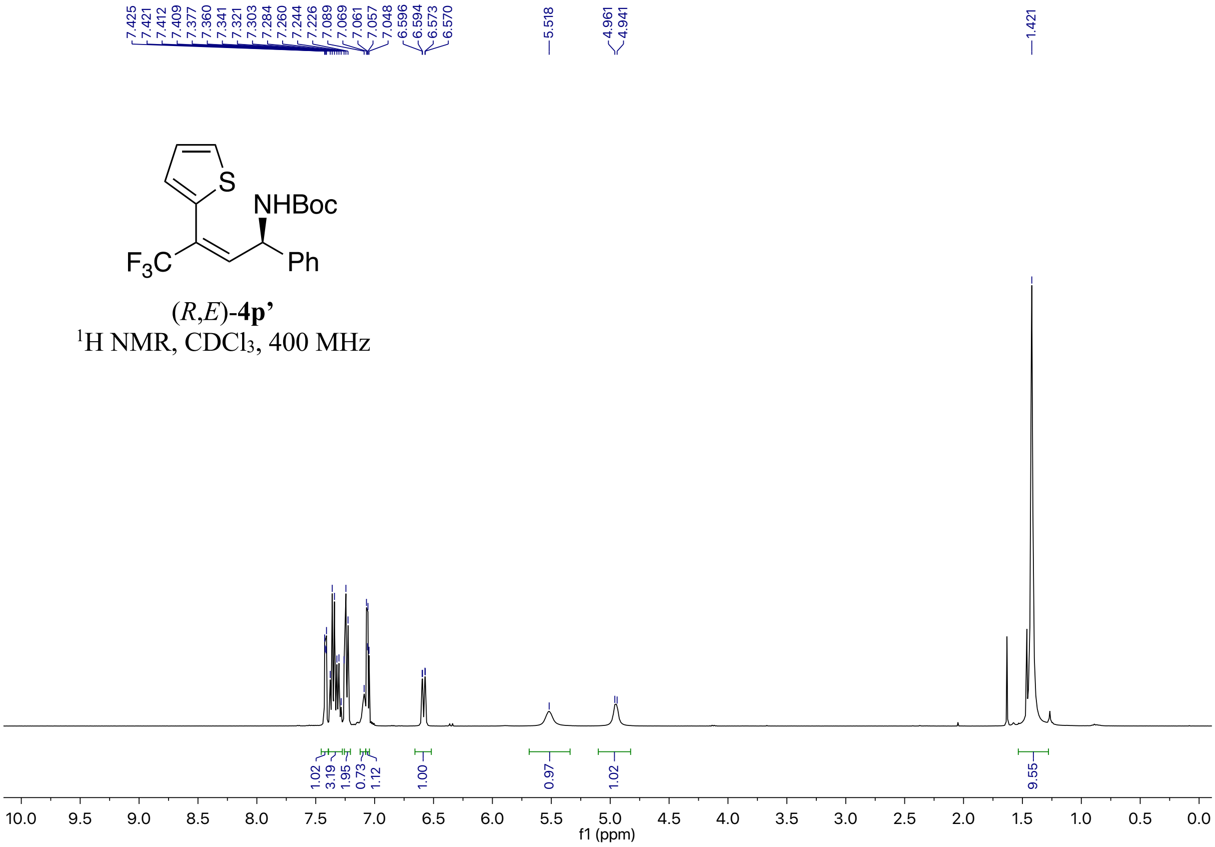

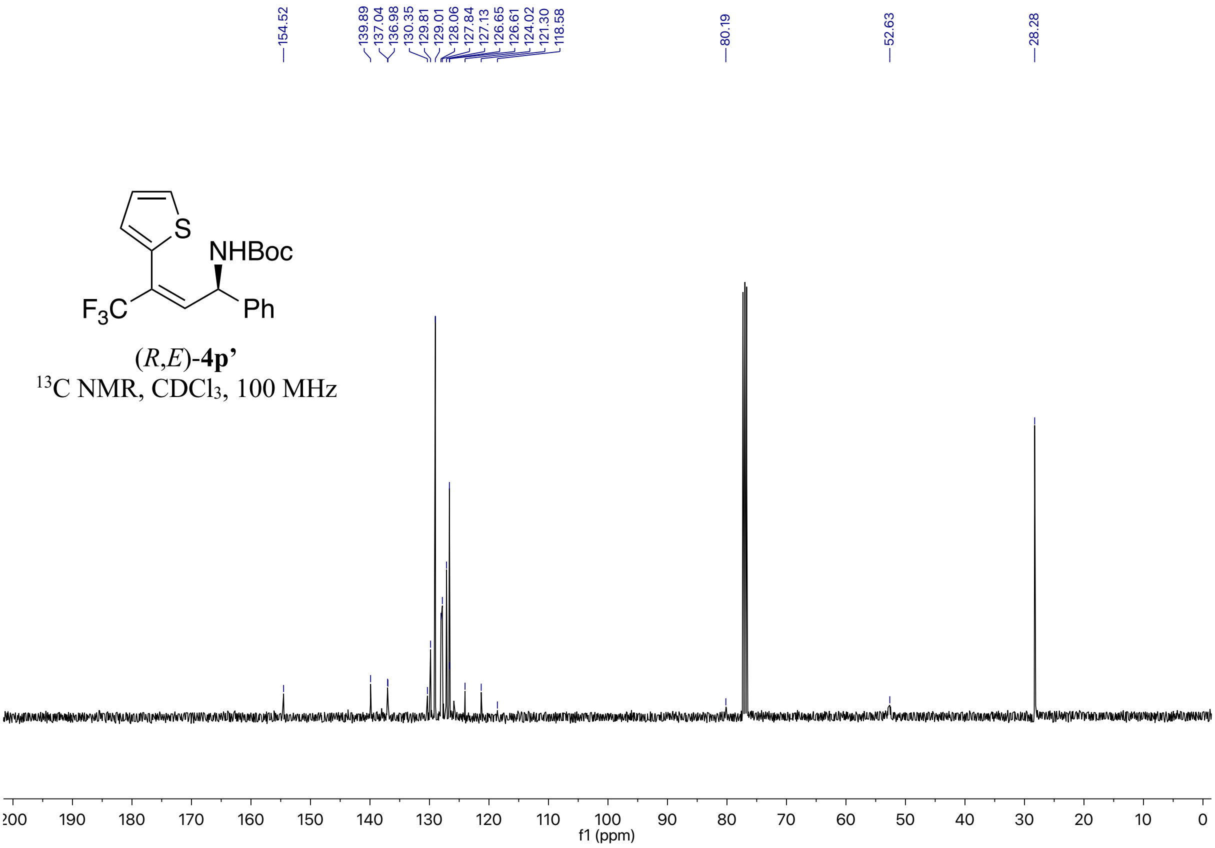

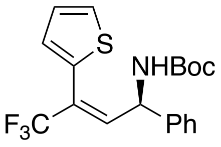

(*R*,*E*)-4p'

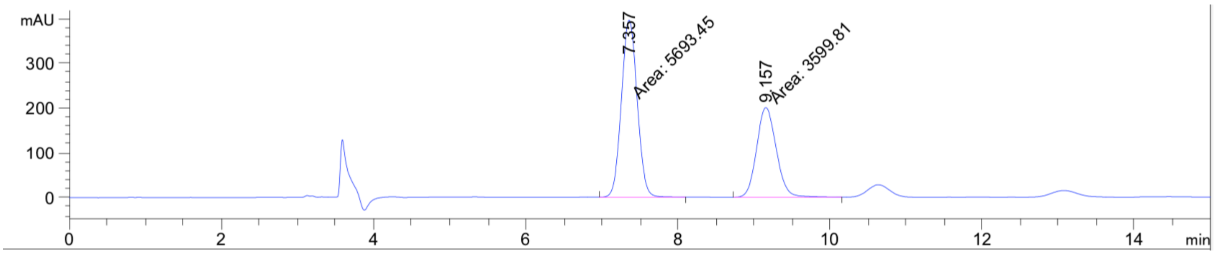

Peak #	RetTime [min]	Type	Width [min]	Area [mAU*s]	Height [mAU]	Area %
1	7.357	MM	0.2398	5693.45459	395.73523	61.2643
2	9.157	MM	0.2990	3599.80835	200.68520	38.7357

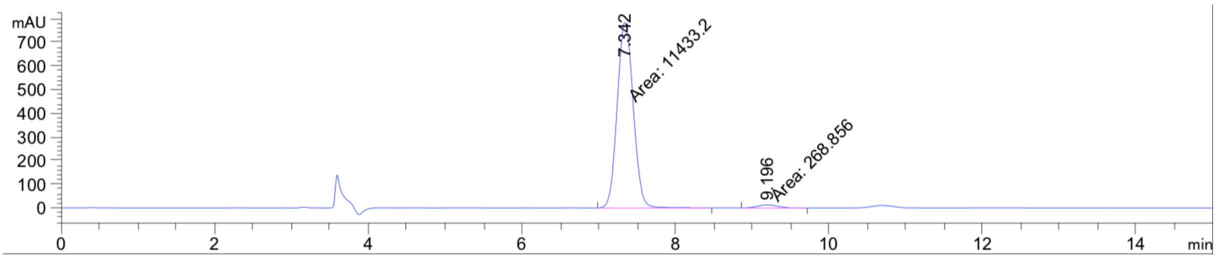

Peak #	RetTime [min]	Type	Width [min]	Area [mAU*s]	Height [mAU]	Area %
1	7.342	MM	0.2431	1.14332e4	783.90002	97.7025
2	9.196	MM	0.3239	268.85626	13.83280	2.2975

NMR of compounds 6 and 6’

(1*S*,3*R*)-4,4,4-Trifluoro-1,3-diphenylbutan-1-amine – minor diastereomer (6a)

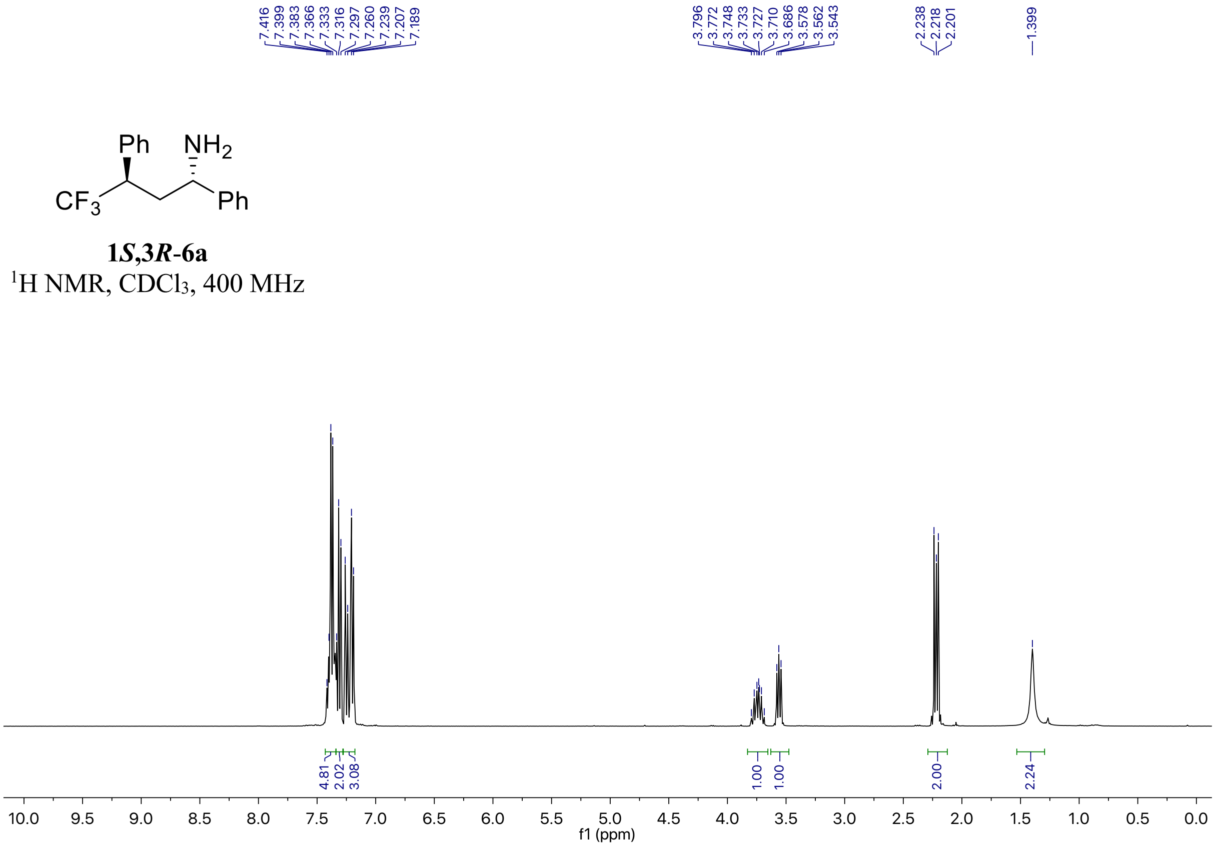

1*S*,3*R*-6a
^{1}H NMR, $CDCl_3$, 400 MHz

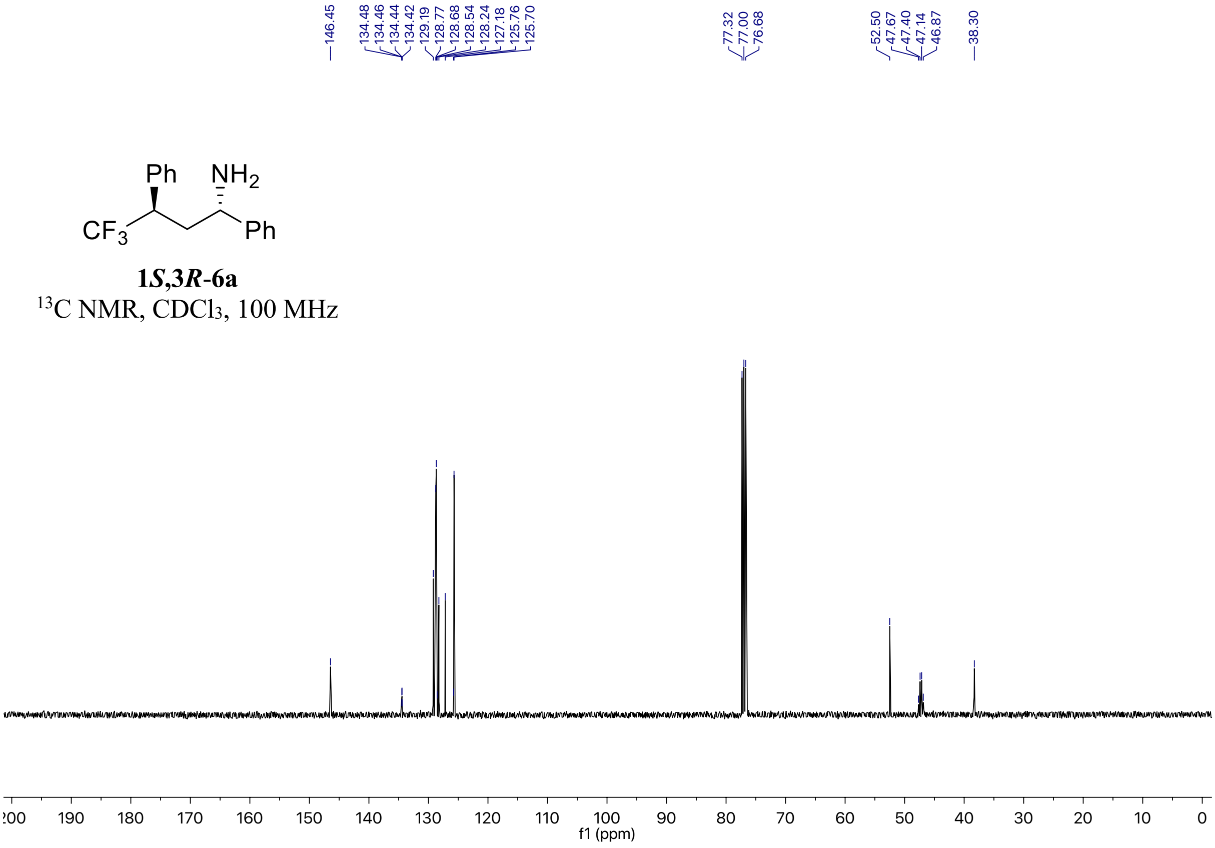

1*S*,3*R*-6a
^{13}C NMR, $CDCl_3$, 100 MHz

tert-Butyl ((1_S_,3_R_)-4,4,4-trifluoro-1,3-diphenylbutyl)carbamate – minor diastereomer (6a')

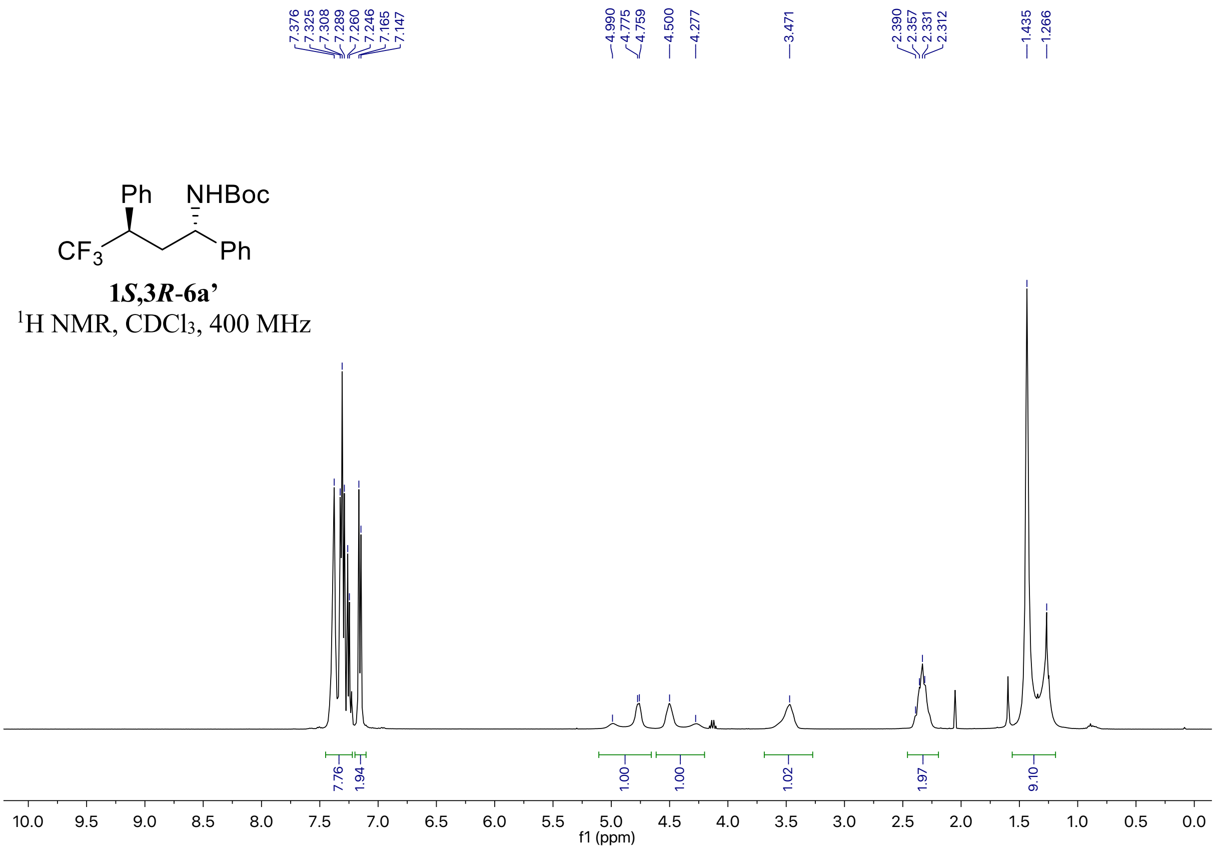

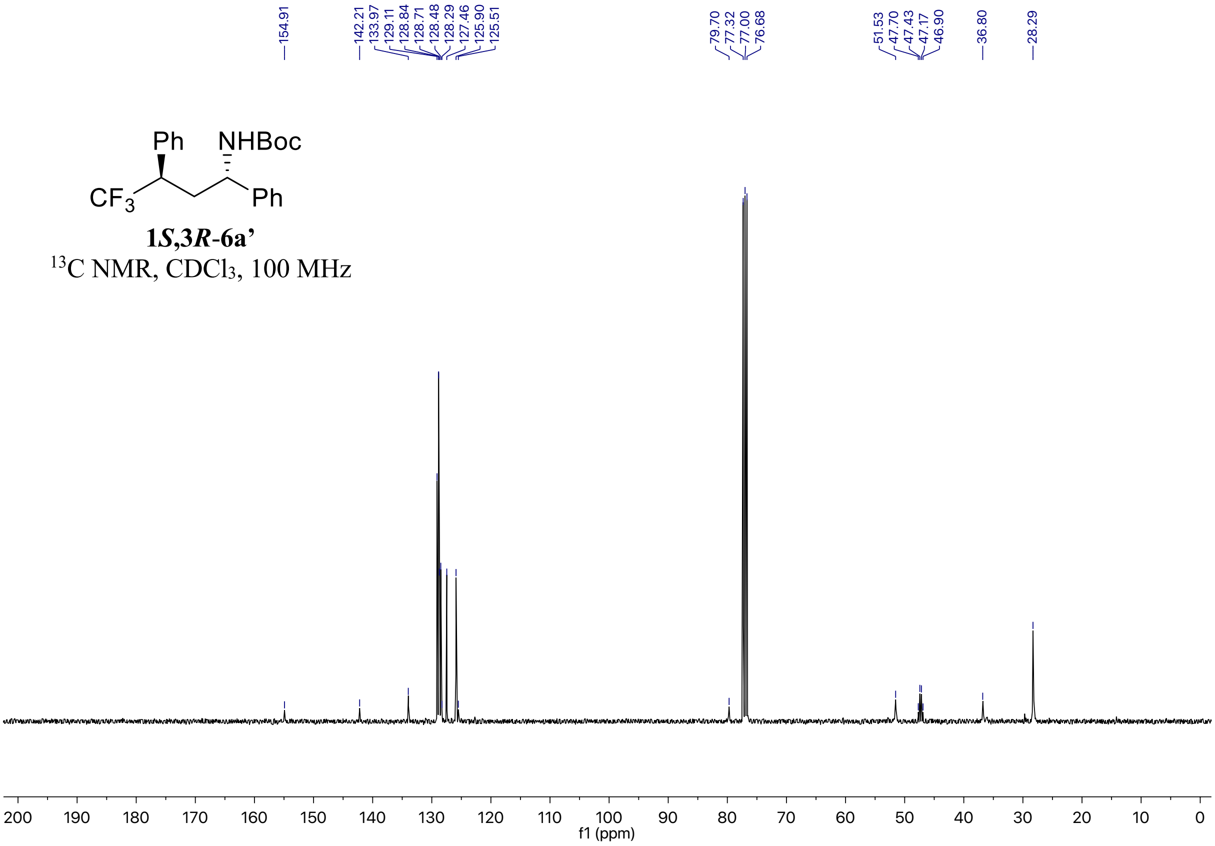

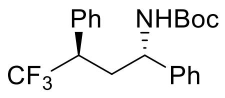

1*S*,3*R*-6a'

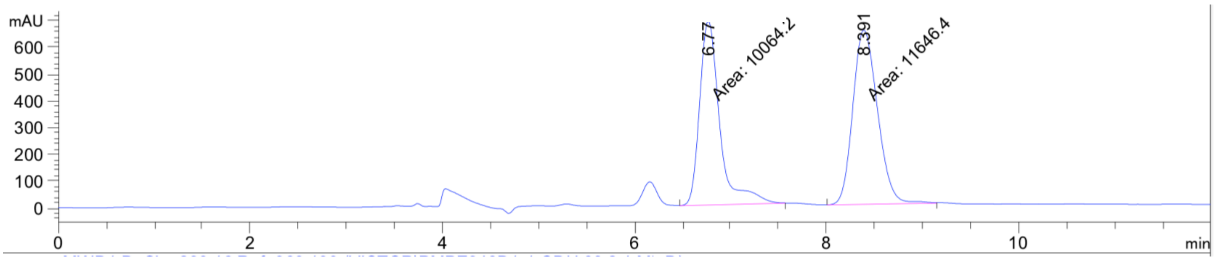

Peak #	RetTime [min]	Type	Width [min]	Area [mAU*s]	Height [mAU]	Area %
1	6.772	MM	0.2438	1.00642e4	687.94897	46.3561
2	8.391	MM	0.2981	1.16464e4	651.24738	53.6439

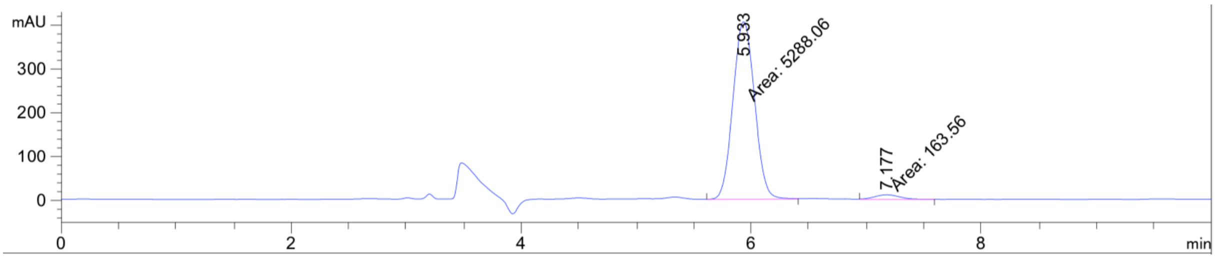

Peak #	RetTime [min]	Type	Width [min]	Area [mAU*s]	Height [mAU]	Area %
1	5.933	MM	0.2177	5288.05957	404.85703	96.9998
2	7.177	MM	0.2617	163.55989	10.41580	3.0002

(1*R*,3*R*)-4,4,4-Trifluoro-1,3-diphenylbutan-1-amine – major diastereomer (6a)

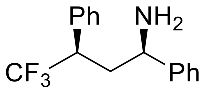

1*R*,3*R*-6a
^{1}H NMR, $CDCl_3$, 400 MHz

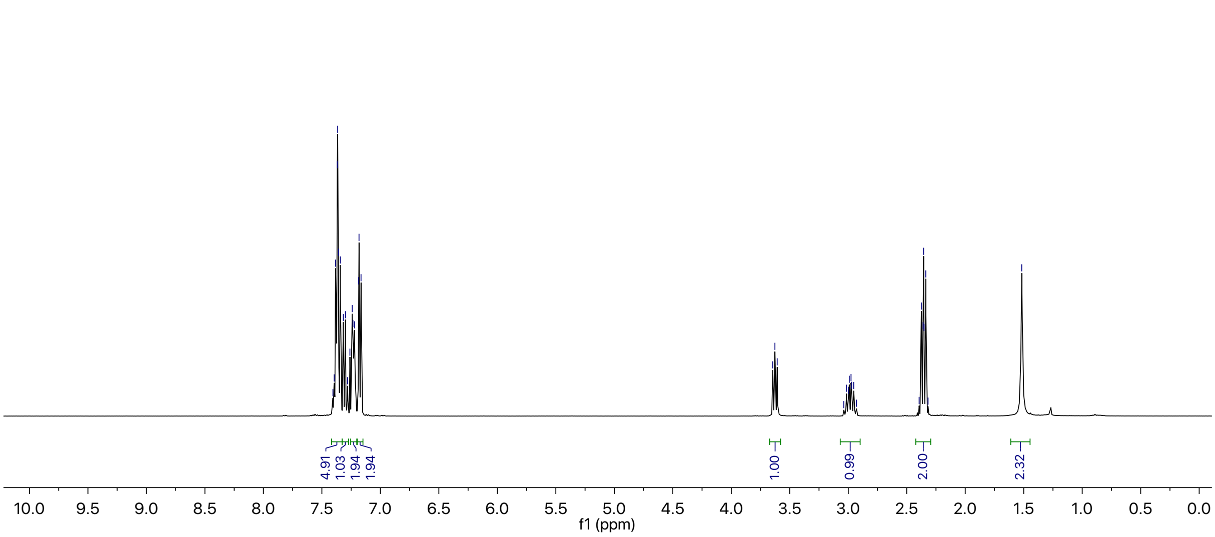

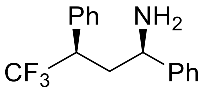

1*R*,3*R*-6a
^{13}C NMR, $CDCl_3$, 100 MHz

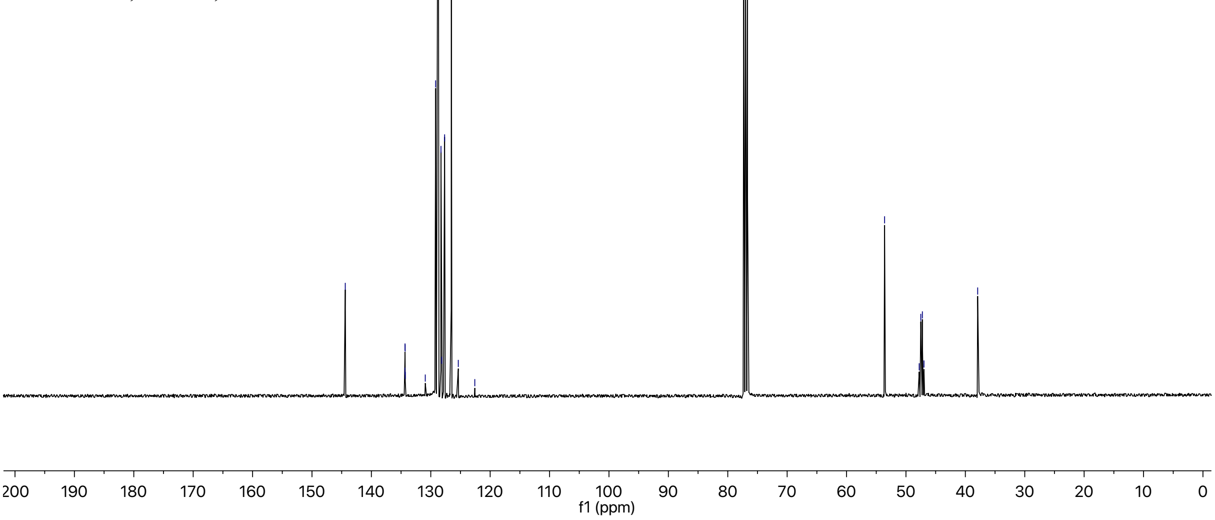

tert-Butyl ((1_R_,3_R_)-4,4,4-trifluoro-1,3-diphenylbutyl)carbamate – major diastereomer (6a')

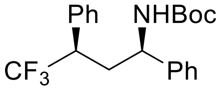

1_R_,3_R_-6a'
^{1}H NMR, $CDCl_3$, 400 MHz

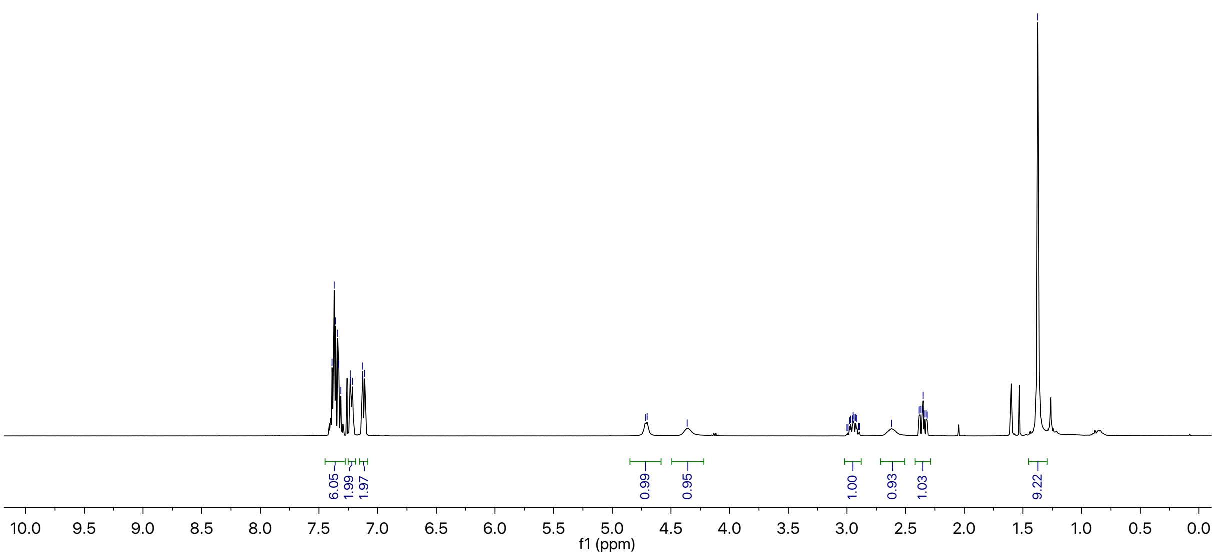

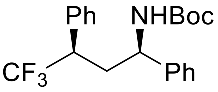

1_R_,3_R_-6a'
^{13}C NMR, $CDCl_3$, 100 MHz

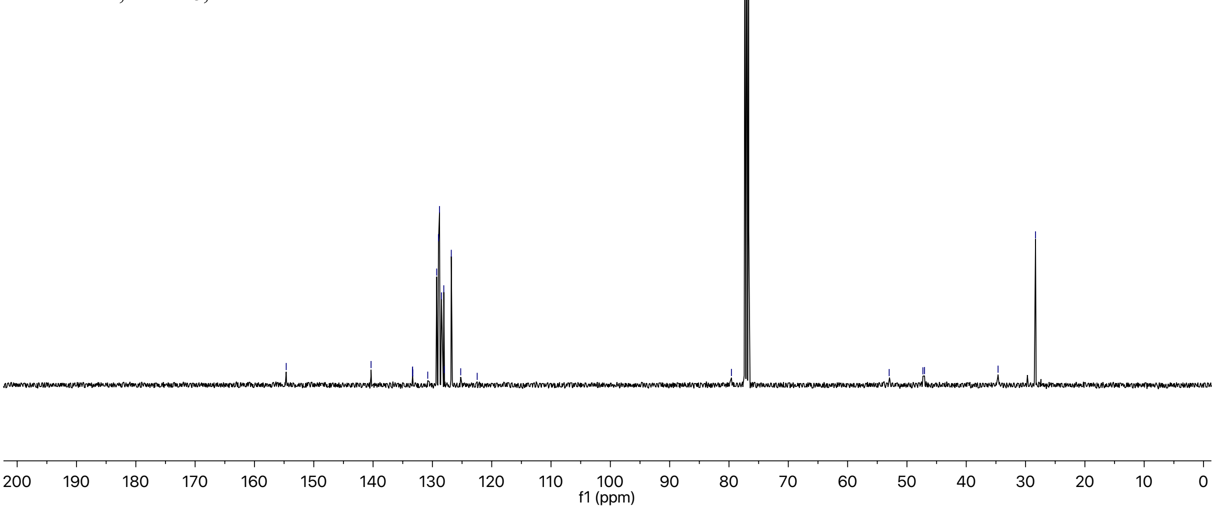

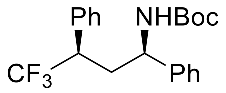

1*R*,3*R*-6a'

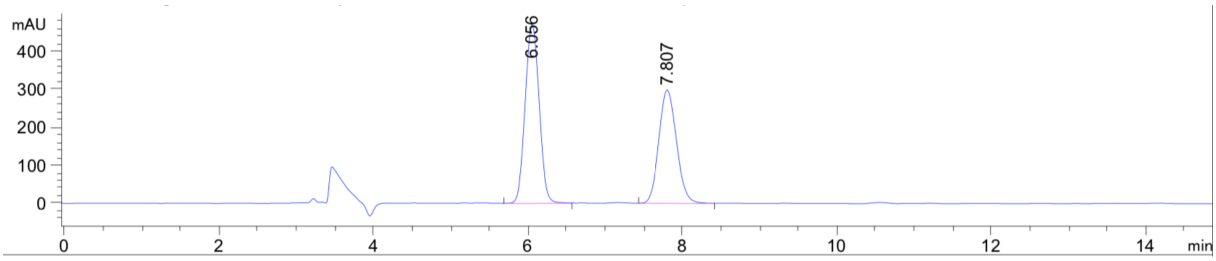

Peak #	RetTime [min]	Type	Width [min]	Area [mAU*s]	Height [mAU]	Area %
1	6.056	BV	0.1990	5949.37158	472.04483	55.5792
2	7.807	BV	0.2499	4754.93506	298.41345	44.4208

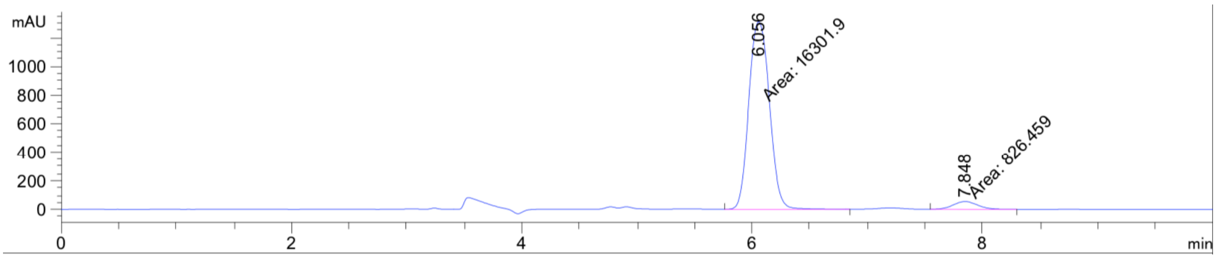

Peak #	RetTime [min]	Type	Width [min]	Area [mAU*s]	Height [mAU]	Area %
1	6.056	MM	0.2069	1.63019e4	1313.05225	95.1749
2	7.848	MM	0.2527	826.45880	54.50021	4.8251

(1*R*,3*R*)-4,4,4-Trifluoro-3-phenyl-1-(*p*-tolyl)butan-1-amine – major diastereomer (6b)

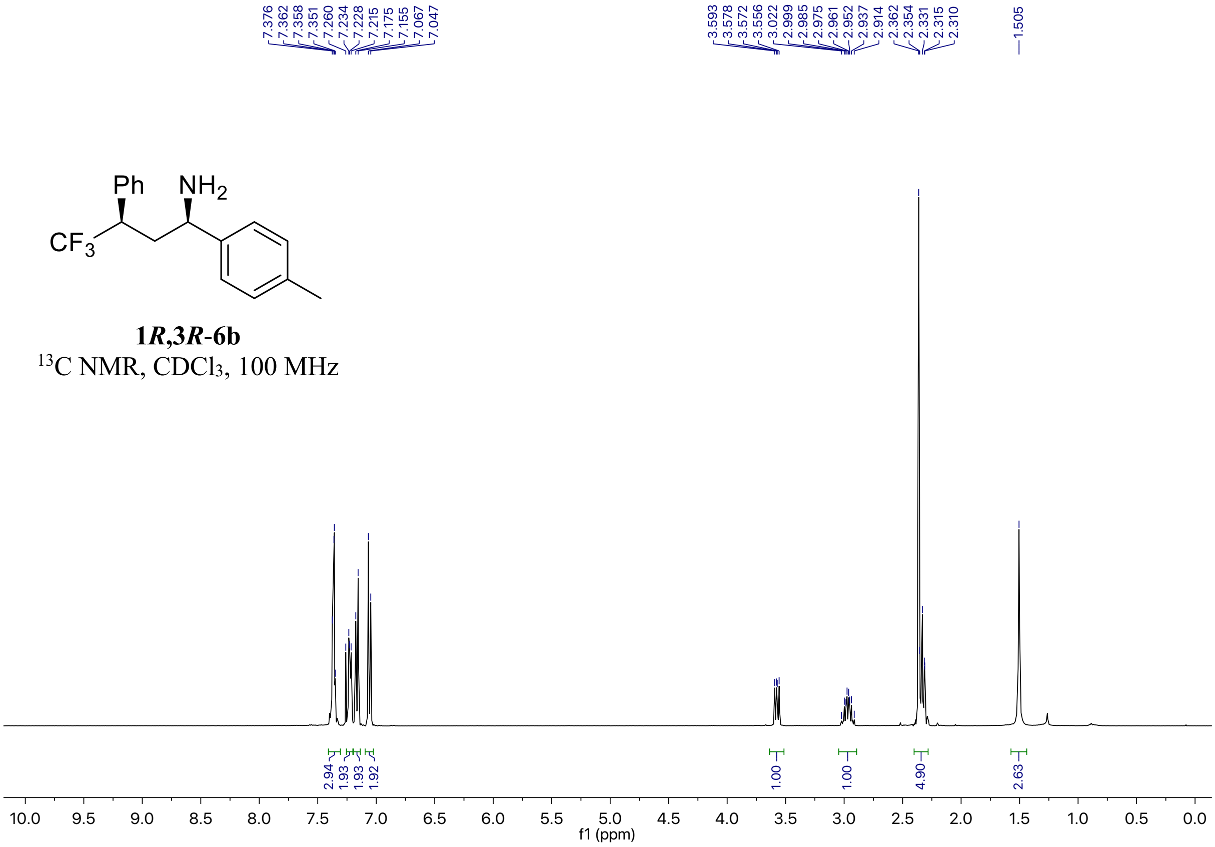

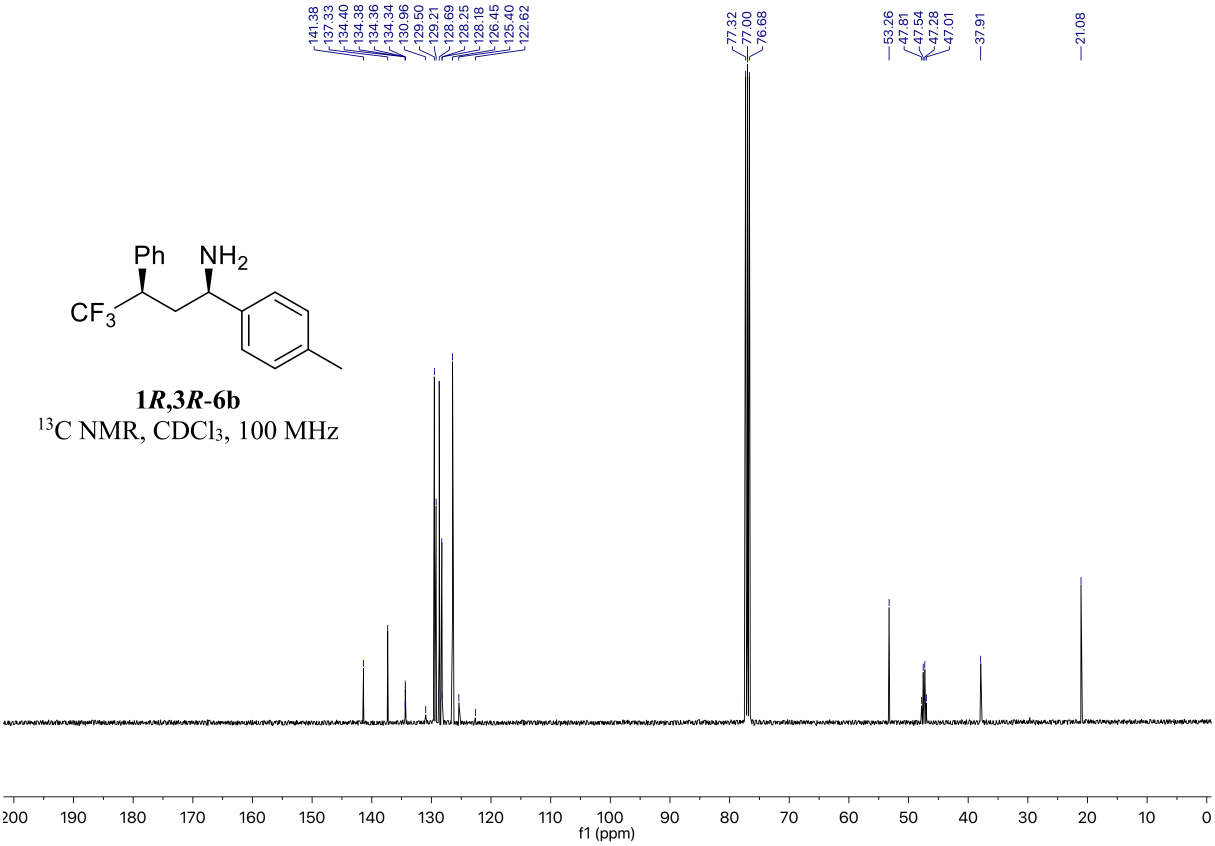

***tert*-Butyl ((1*R*,3*R*)-4,4,4-trifluoro-3-phenyl-1-(p-tolyl)butyl)carbamate – major diastereomer (6b')**

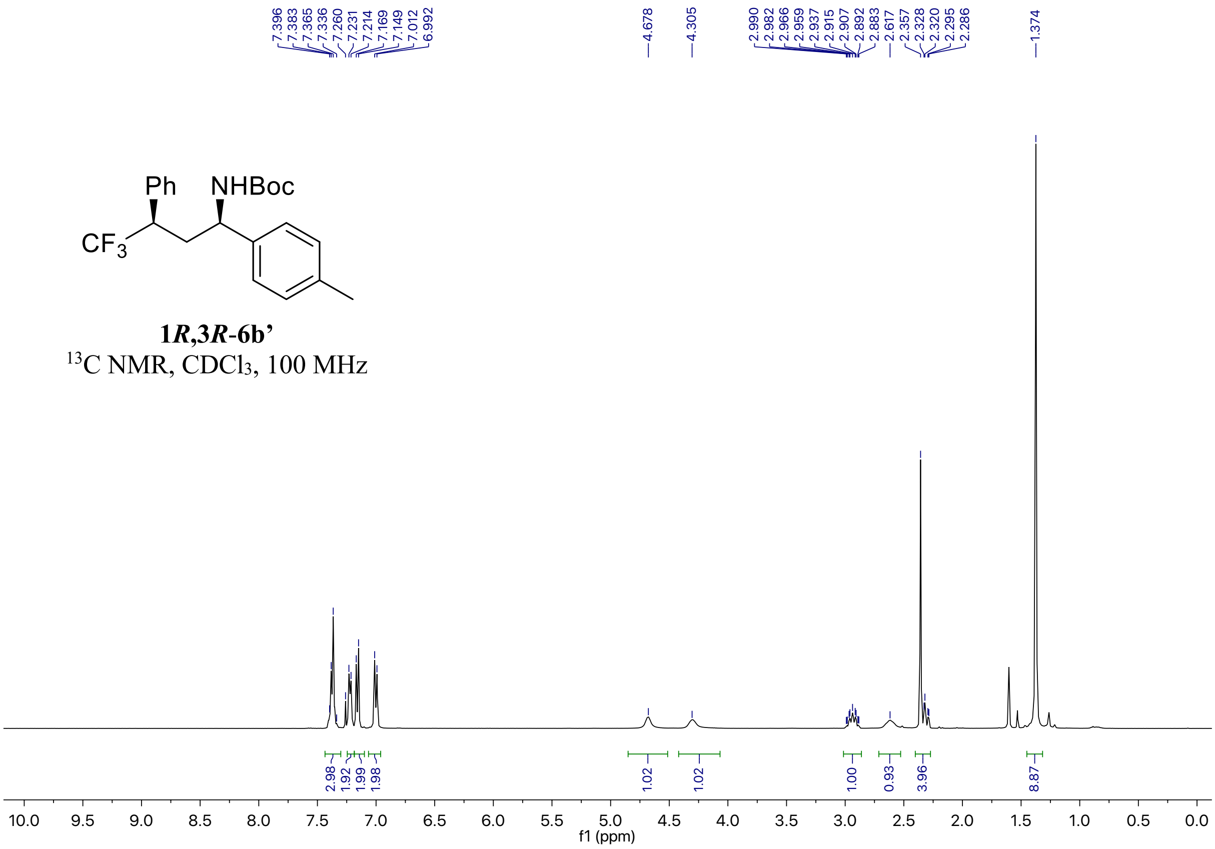

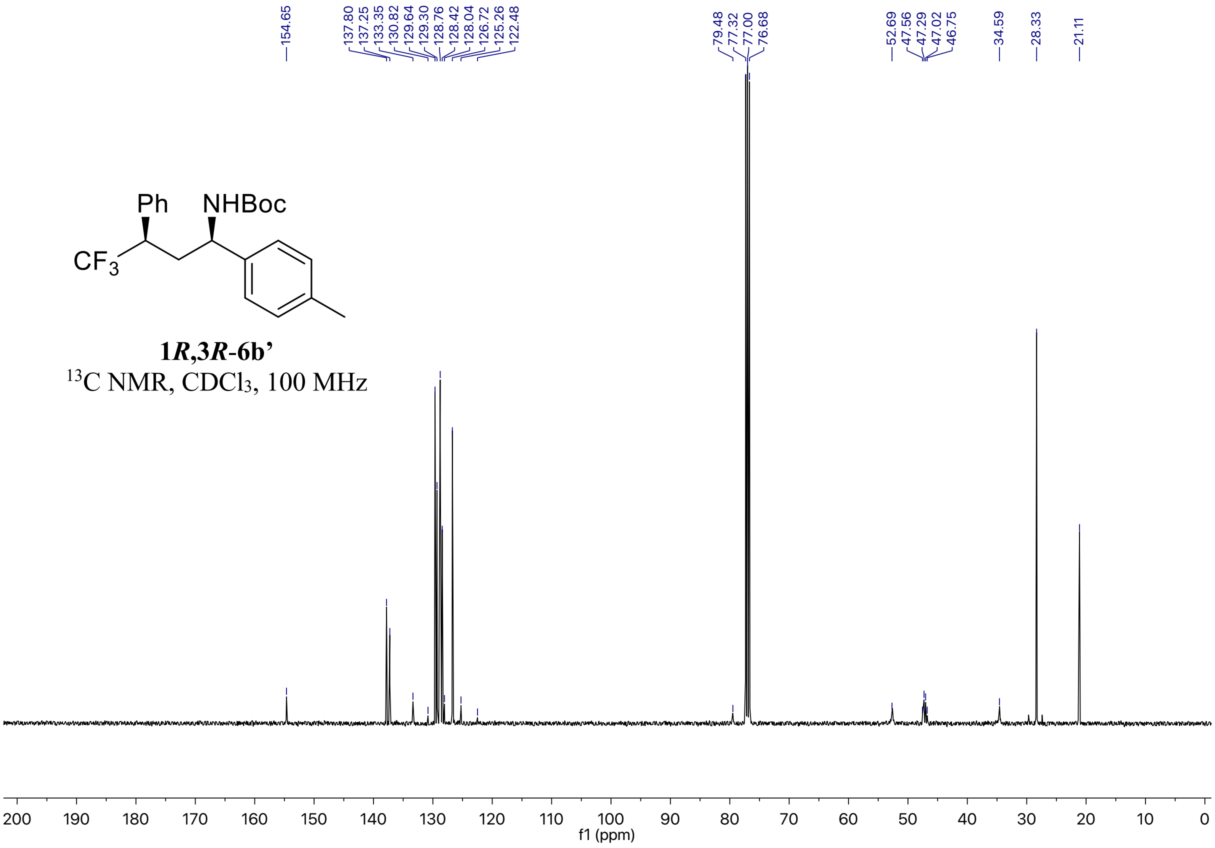

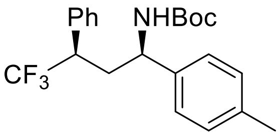

1*R*,3*R*-6b'

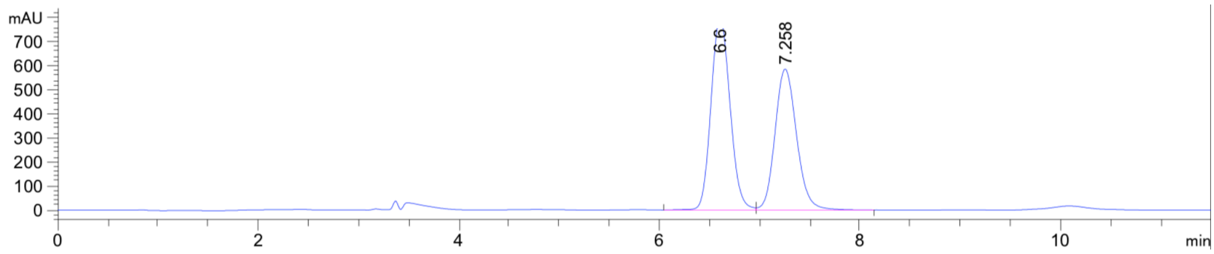

Peak #	RetTime [min]	Type	Width [min]	Area [mAU*s]	Height [mAU]	Area %
1	6.610	BV	0.2083	1.05567e4	798.36798	54.4745
2	7.258	VV	0.2353	8822.41699	587.30841	45.5255

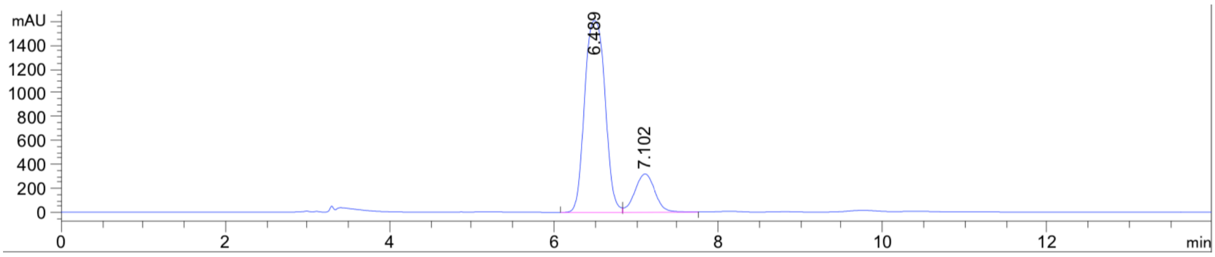

Peak #	RetTime [min]	Type	Width [min]	Area [mAU*s]	Height [mAU]	Area %
1	6.489	BV	0.2812	2.76891e4	1605.84692	83.4816
2	7.102	VV	0.2719	5478.82080	319.71326	16.5184

(1*R*,3*R*)-4,4,4-Trifluoro-1-(4-methoxyphenyl)-3-phenylbutan-1-amine – major diastereomer (6c)

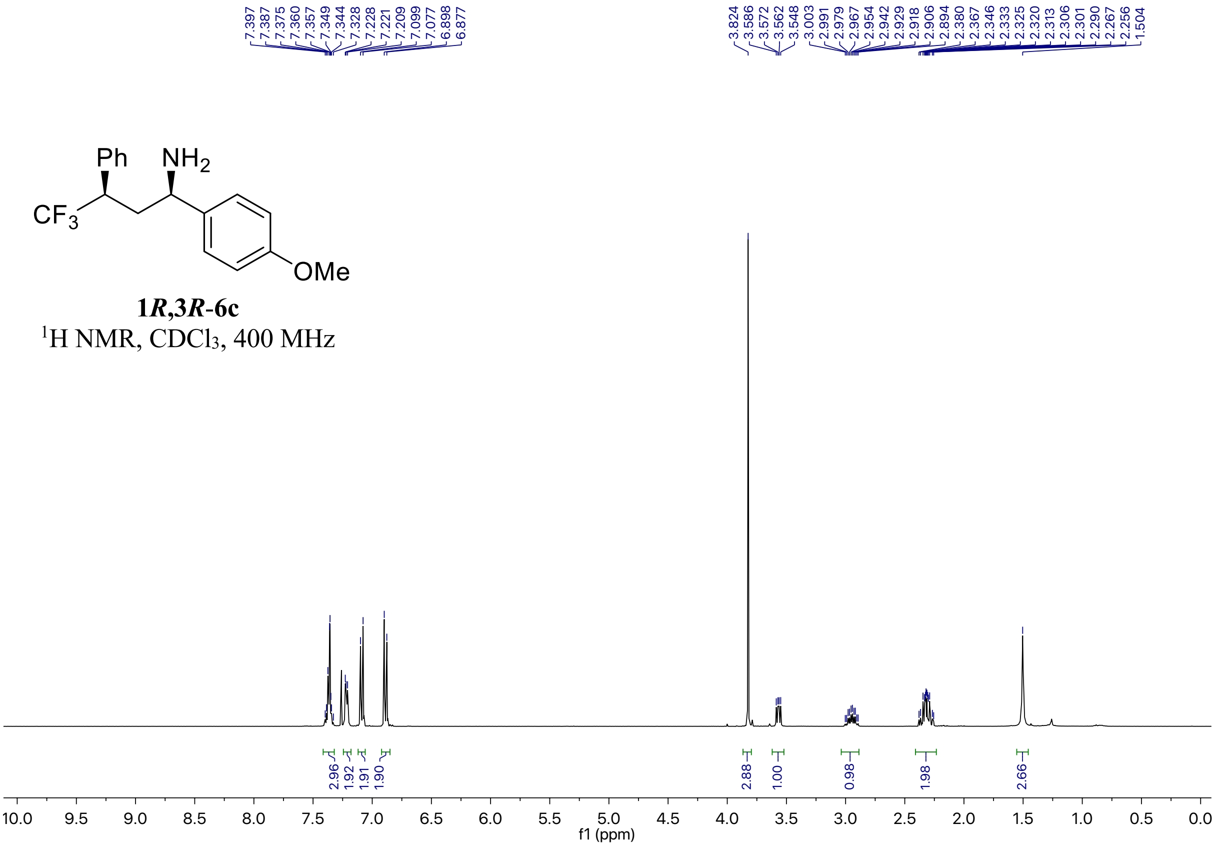

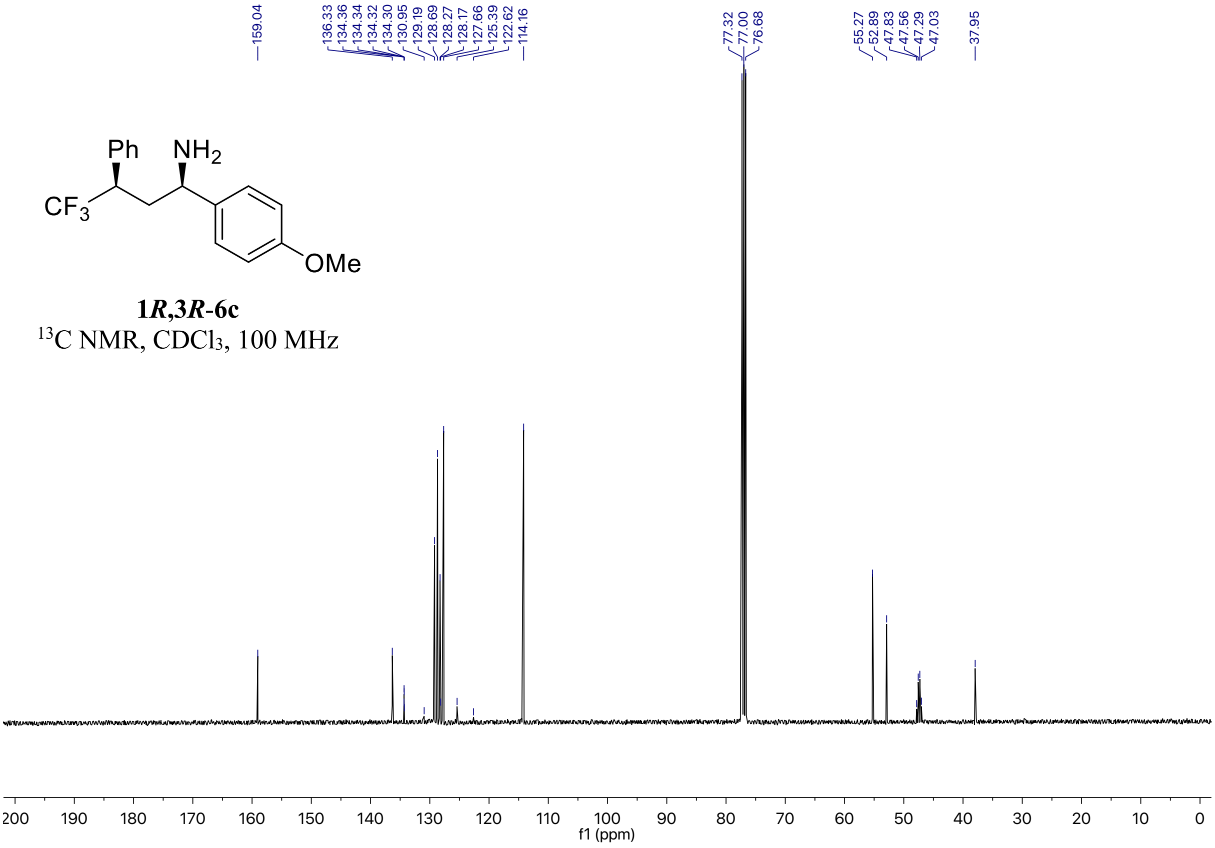

tert-Butyl ((1_R_,3_R_)-4,4,4-trifluoro-1-(4-methoxyphenyl)-3-phenylbutyl)carbamate – major diastereomer (6c')

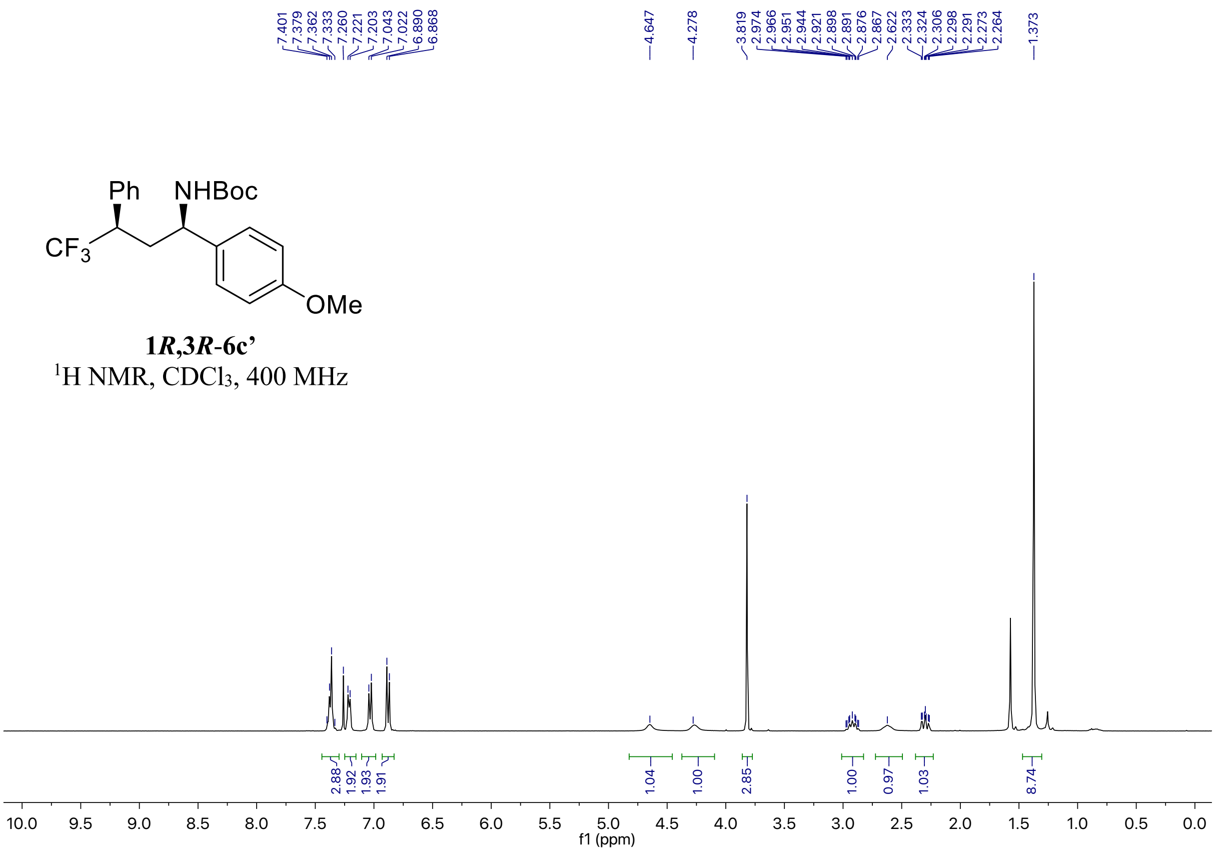

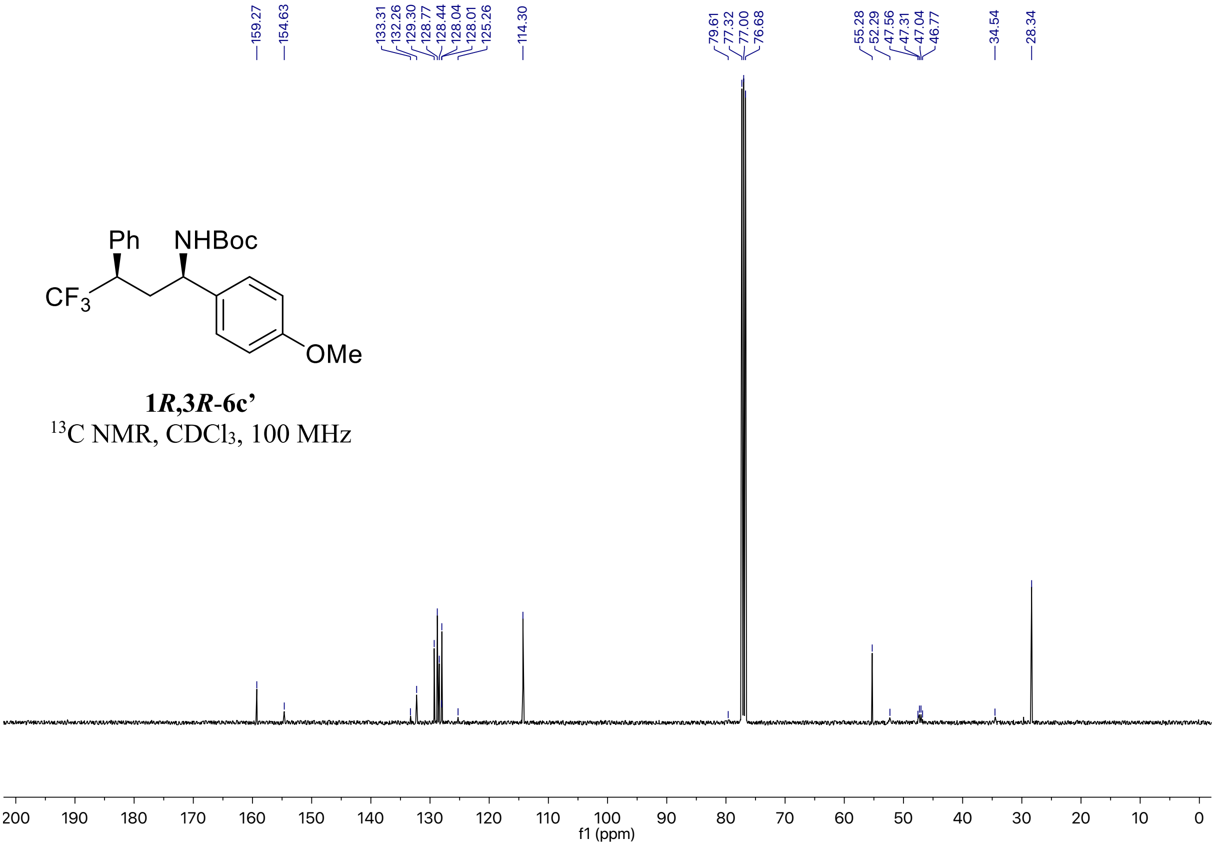

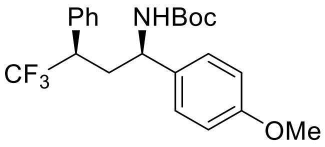

1*R*,3*R*-6c'

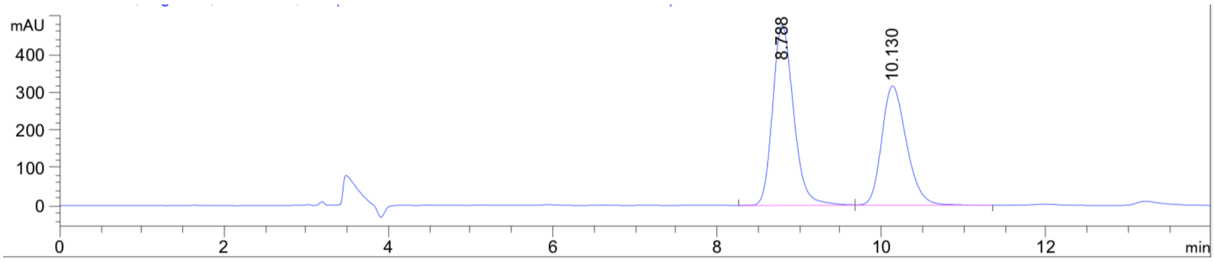

Peak #	RetTime [min]	Type	Width [min]	Area [mAU*s]	Height [mAU]	Area %
1	8.788	BV	0.2756	8483.00000	476.69098	56.0823
2	10.130	VB	0.3233	6642.98633	315.91843	43.9177

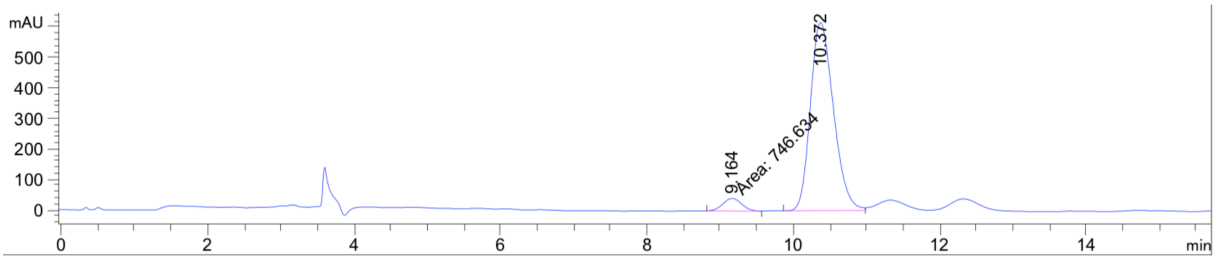

Peak #	RetTime [min]	Type	Width [min]	Area [mAU*s]	Height [mAU]	Area %
1	9.164	MM	0.2992	746.63446	41.58955	5.2305
2	10.372	BV	0.3397	1.35279e4	617.15686	94.7695

(1*R*,3*R*)-1-(4-Bromophenyl)-4,4,4-trifluoro-3-phenylbutan-1-amine – major diastereomer (6d)

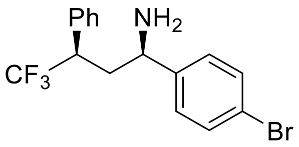

1*R*,3*R*-6d
^{1}H NMR, $CDCl_3$, 400 MHz

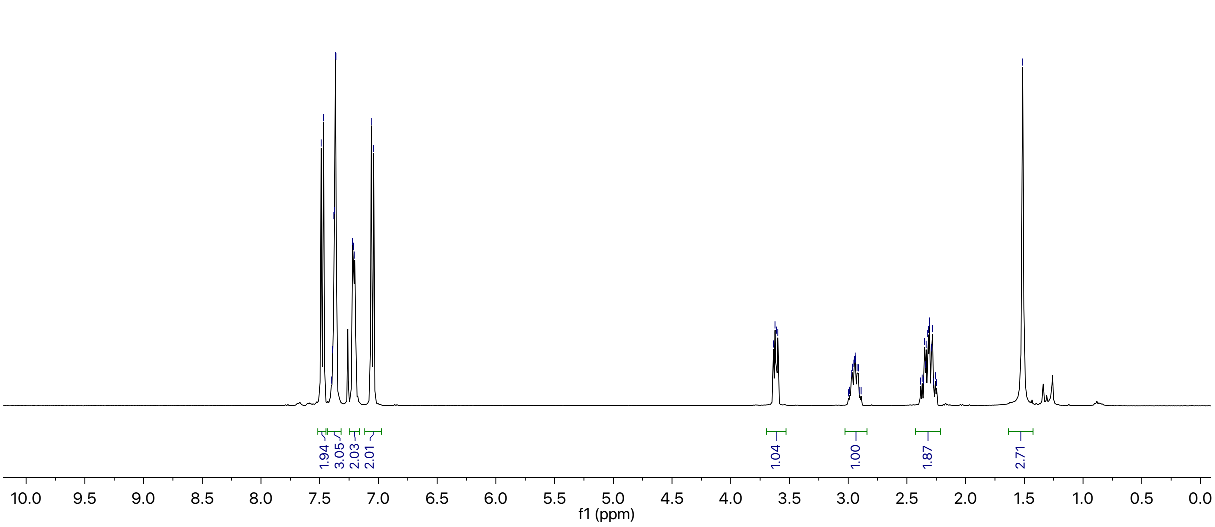

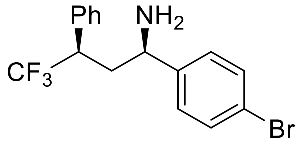

1*R*,3*R*-6d
^{13}C NMR, $CDCl_3$, 100 MHz

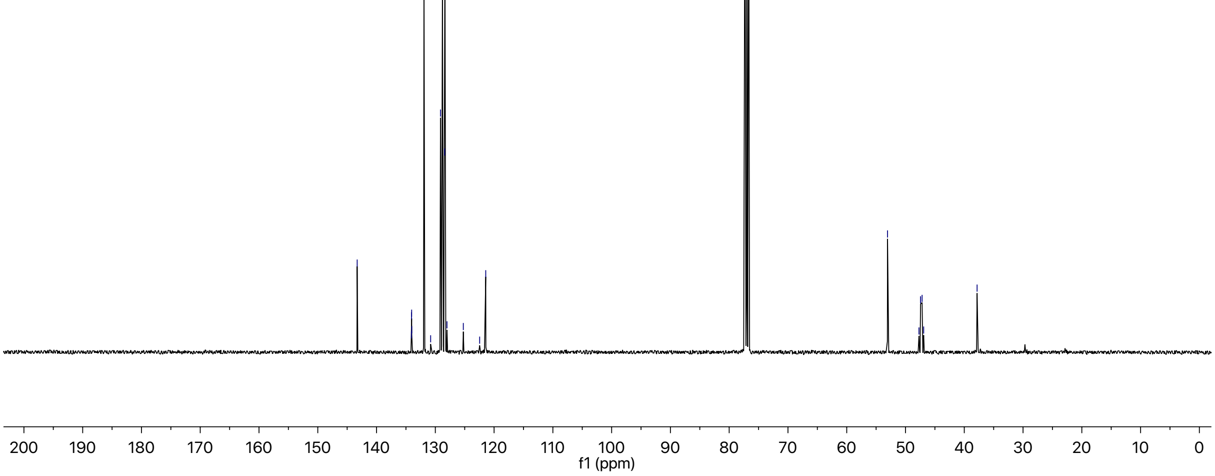

***tert*-Butyl ((1*R*,3*R*)-1-(4-bromophenyl)-4,4,4-trifluoro-3-phenylbutyl)carbamate – major diastereomer (6d')**

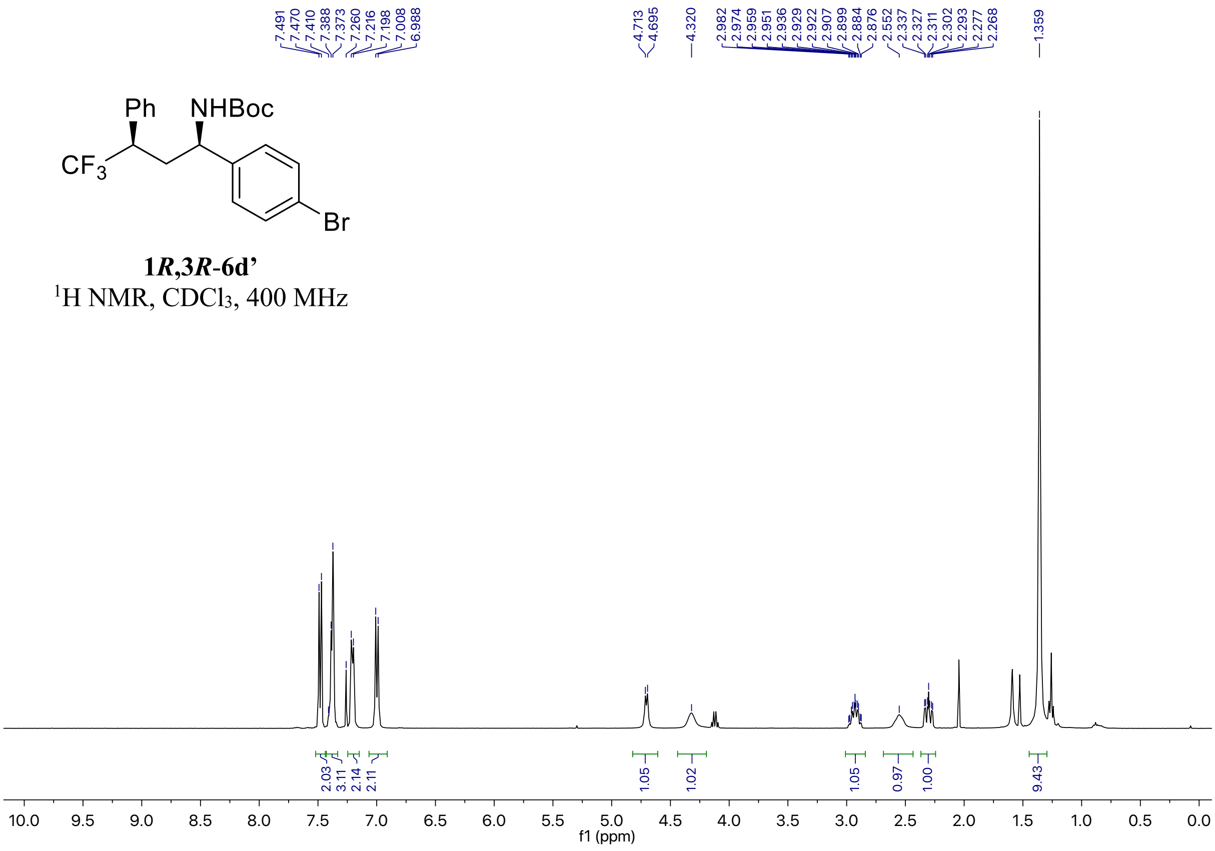

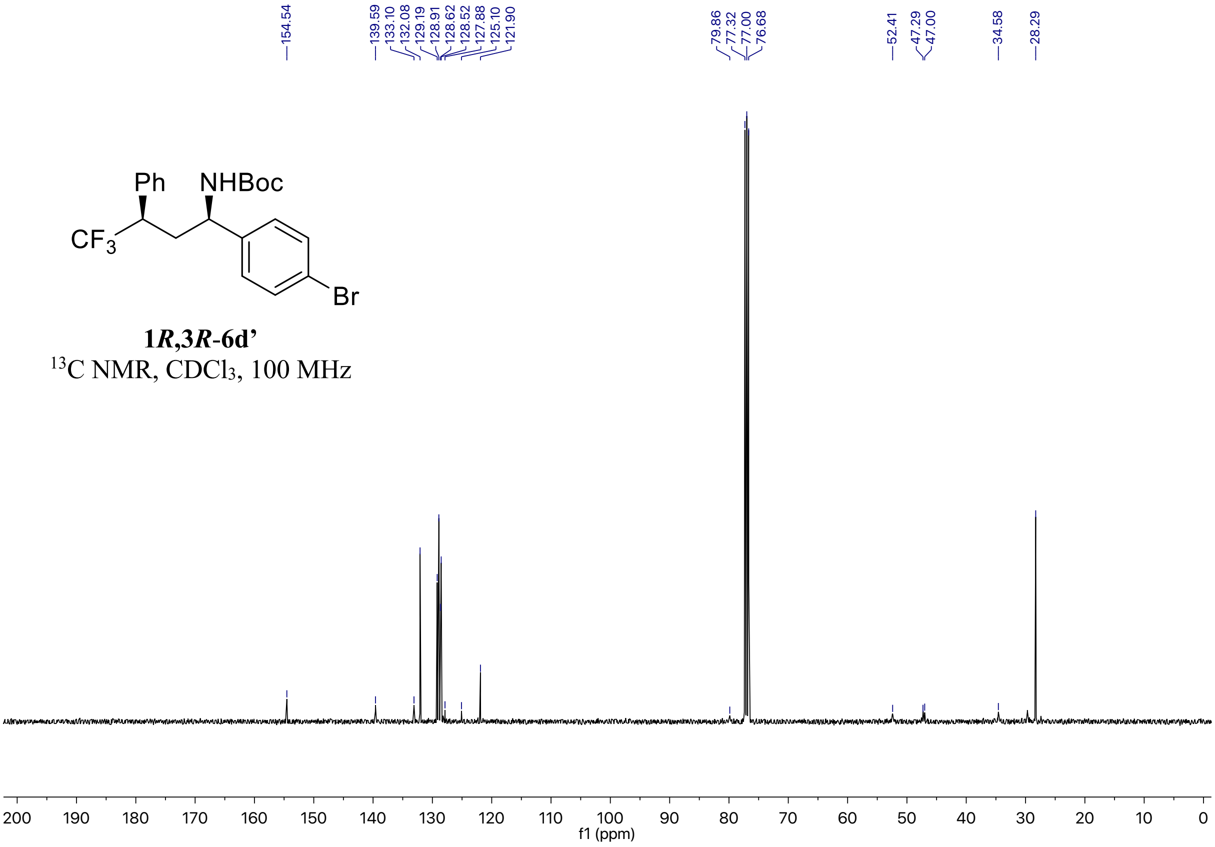

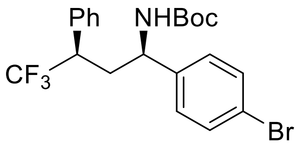

1*R*,3*R*-6d’

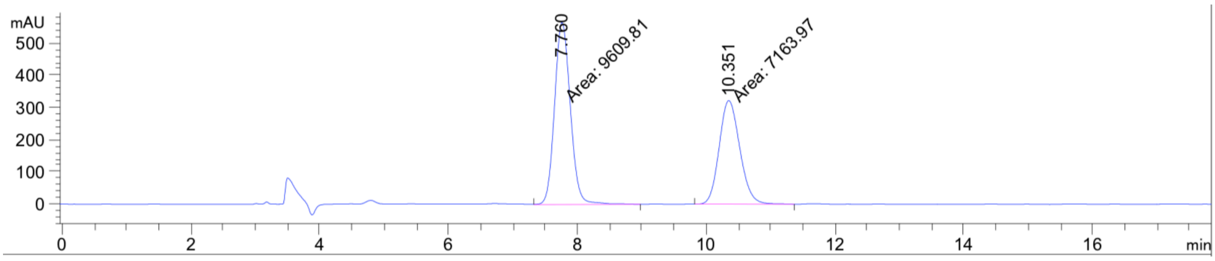

Peak #	RetTime [min]	Type	Width [min]	Area [mAU*s]	Height [mAU]	Area %
1	7.760	MM	0.2838	9609.80762	564.44696	57.2907
2	10.351	MM	0.3713	7163.97168	321.60458	42.7093

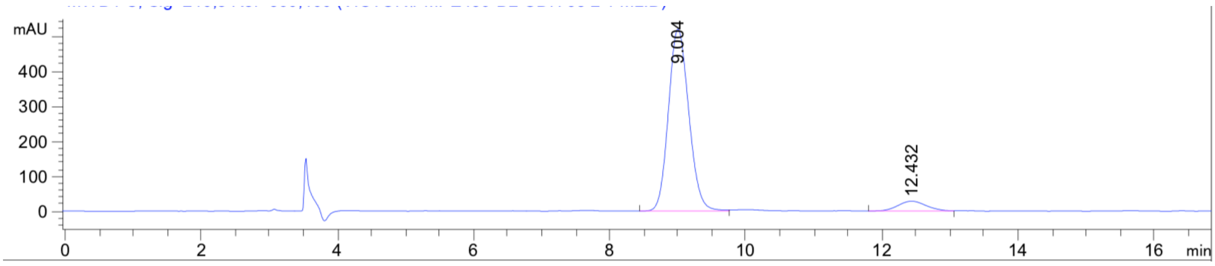

Peak #	RetTime [min]	Type	Width [min]	Area [mAU*s]	Height [mAU]	Area %
1	9.004	BV	0.3245	1.07869e4	518.95776	92.9500
2	12.432	BV	0.4319	818.16278	28.08563	7.0500

(1*S*,3*R*)-4,4,4-Trifluoro-3-phenyl-1-(4-(trifluoromethyl)phenyl)butan-1-amine – major diastereomer (6e)

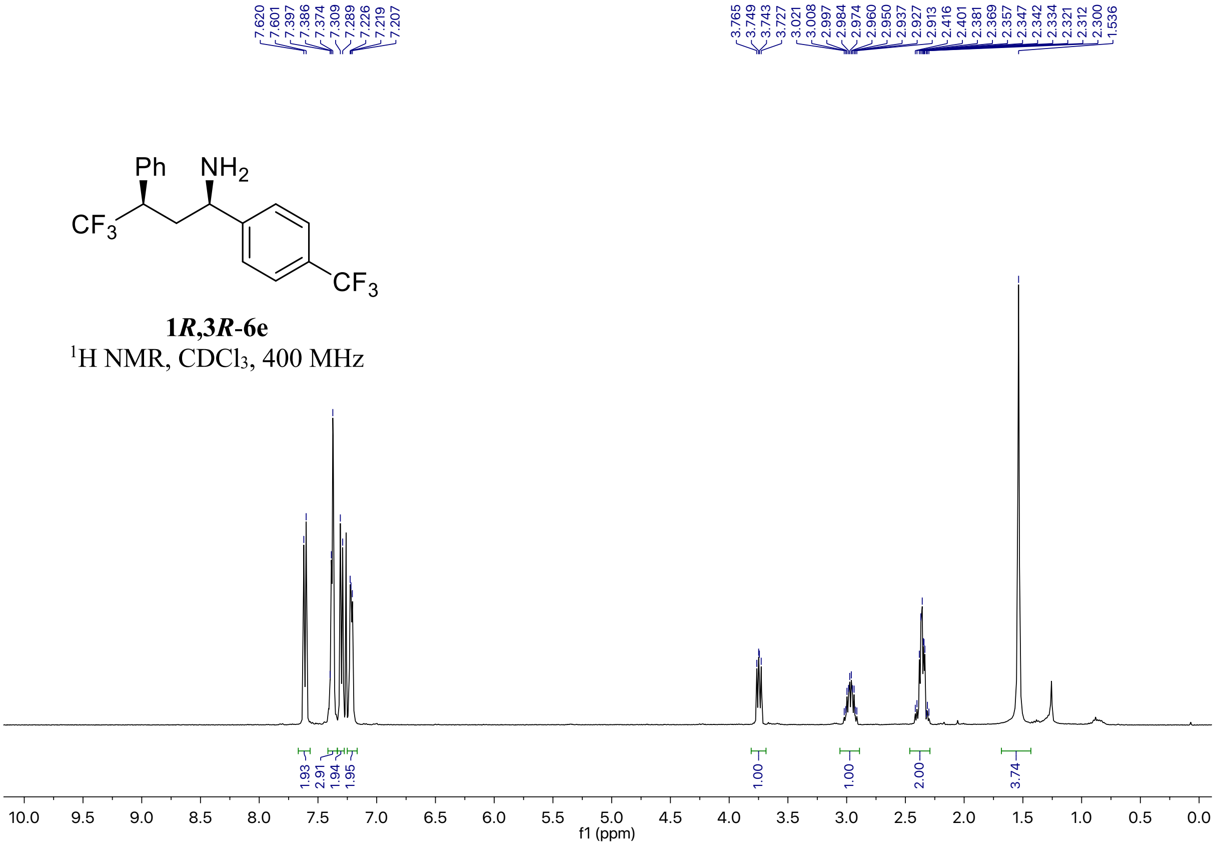

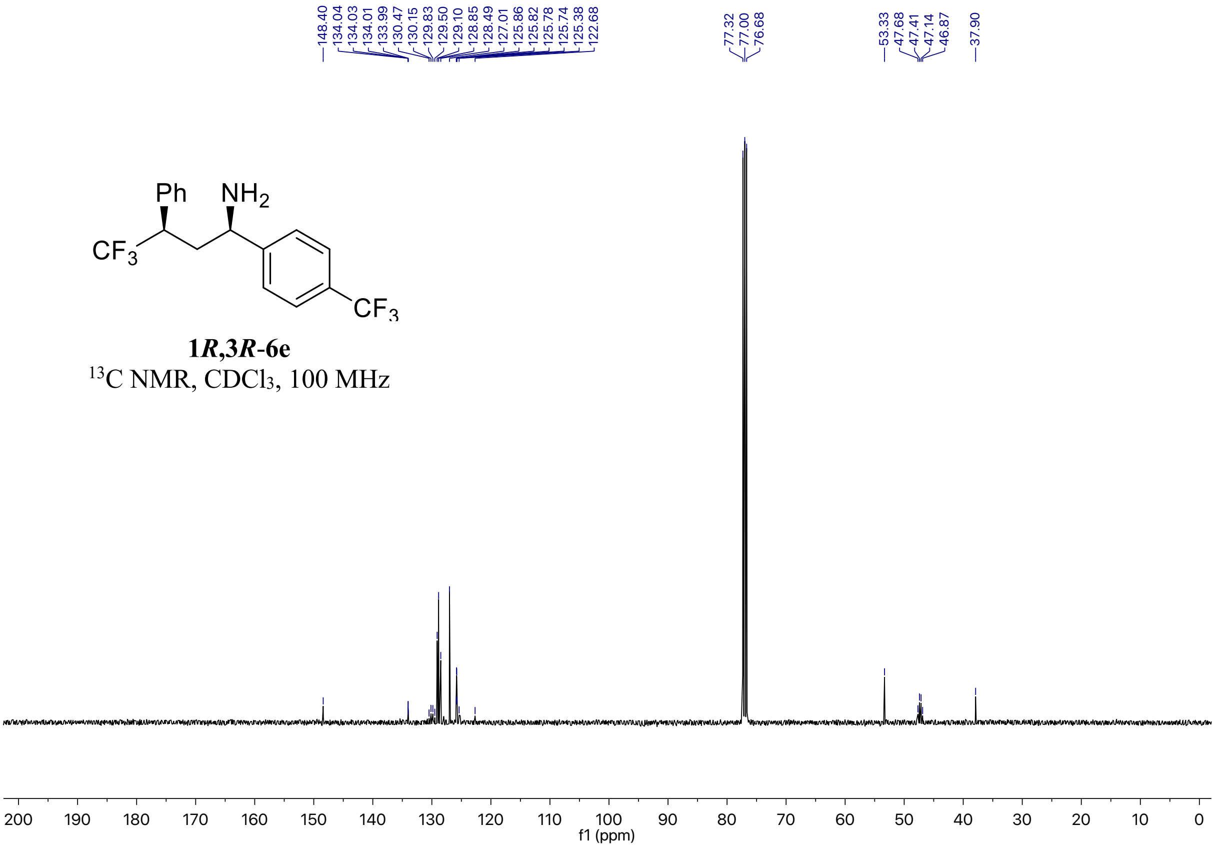

tert-Butyl ((1_R_,3_R_)-4,4,4-trifluoro-3-phenyl-1-(4-(trifluoromethyl)phenyl)butyl)carbamate – major diastereomer (6e’)

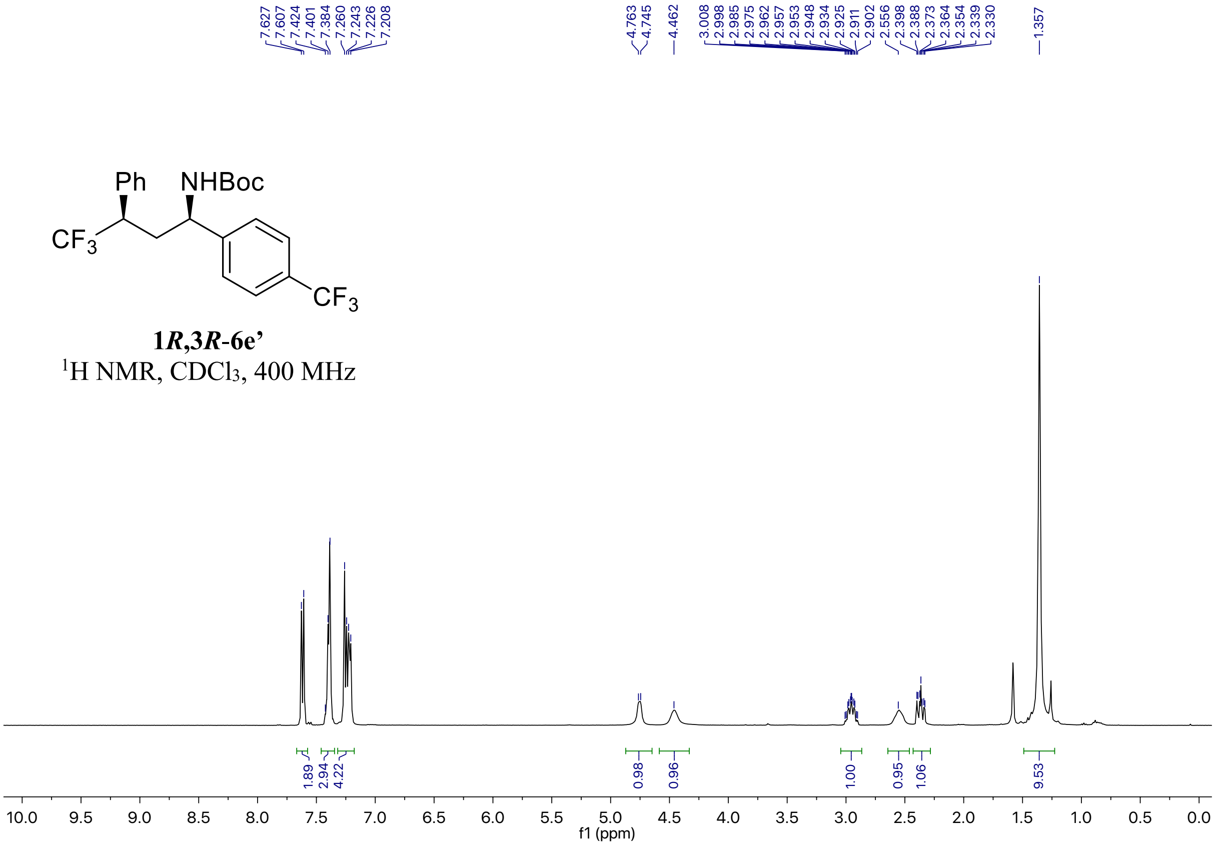

1_R_,3_R_-6e’
^{1}H NMR, $CDCl_3$, 400 MHz

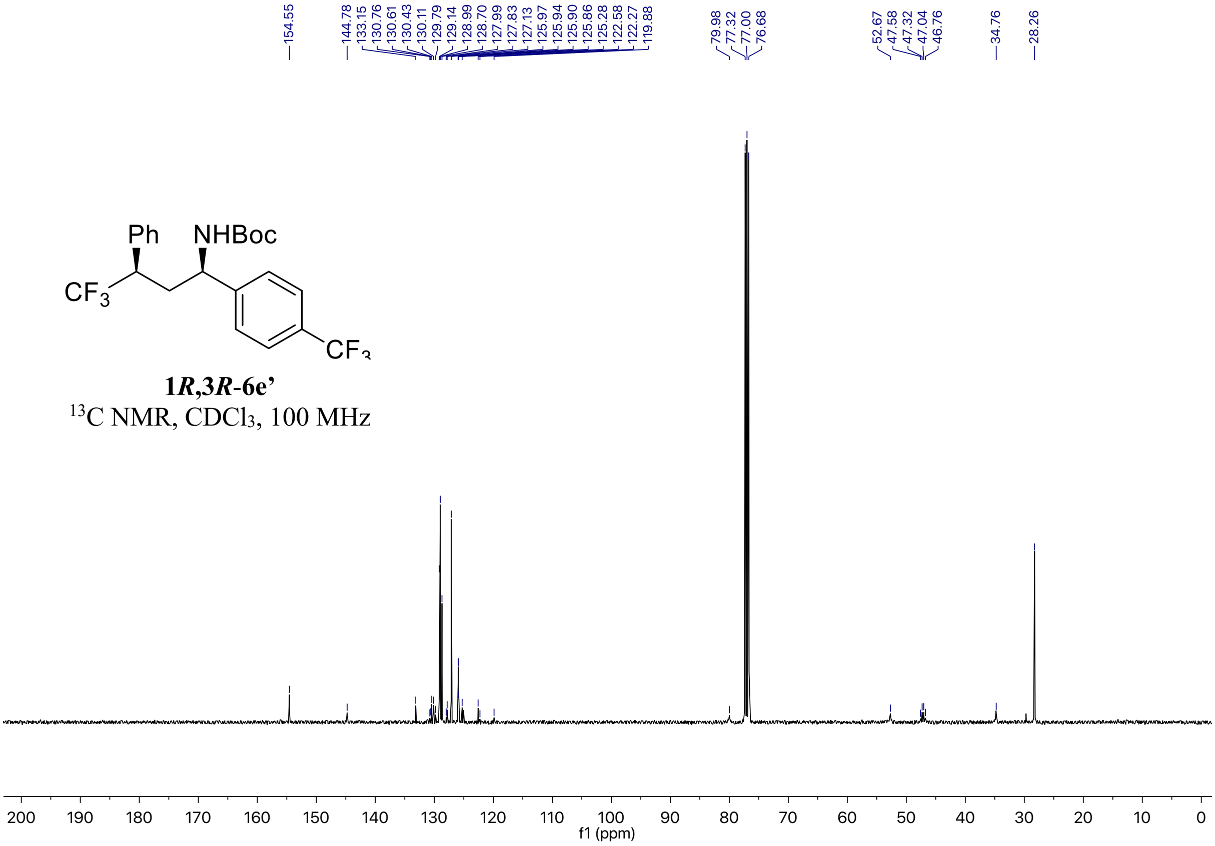

1_R_,3_R_-6e’
^{13}C NMR, $CDCl_3$, 100 MHz

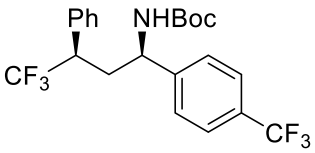

1*R*,3*R*-6e'

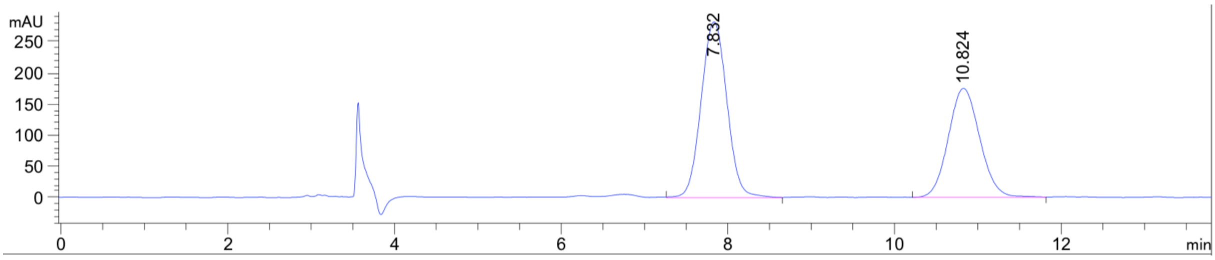

Peak #	RetTime [min]	Type	Width [min]	Area [mAU*s]	Height [mAU]	Area %
1	7.832	VB	0.3398	6039.44385	282.05258	56.7776
2	10.824	VV	0.4104	4597.56885	175.05698	43.2224

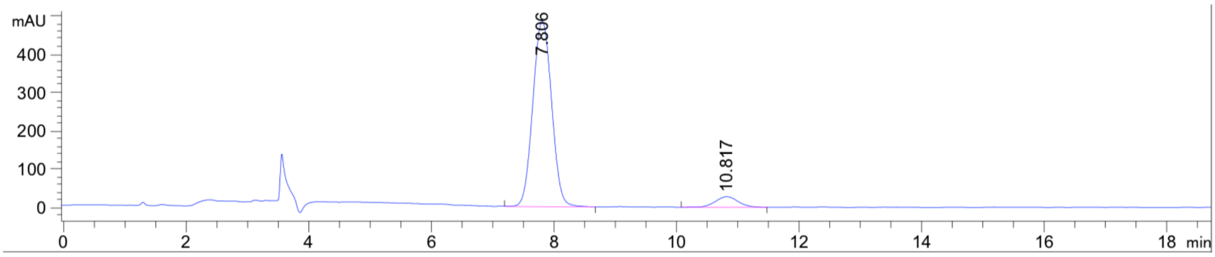

Peak #	RetTime [min]	Type	Width [min]	Area [mAU*s]	Height [mAU]	Area %
1	7.806	VB	0.3381	1.03017e4	484.37332	93.4268
2	10.817	BB	0.3918	724.79871	27.64863	6.5732

(1*S*,3*R*)-4,4,4-trifluoro-1-(naphthalen-2-yl)-3-phenylbutan-1-amine – minor diastereomer (6f)

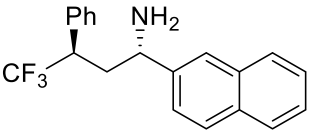

1*S*,3*R*-6f
^{1}H NMR, $CDCl_3$, 400 MHz

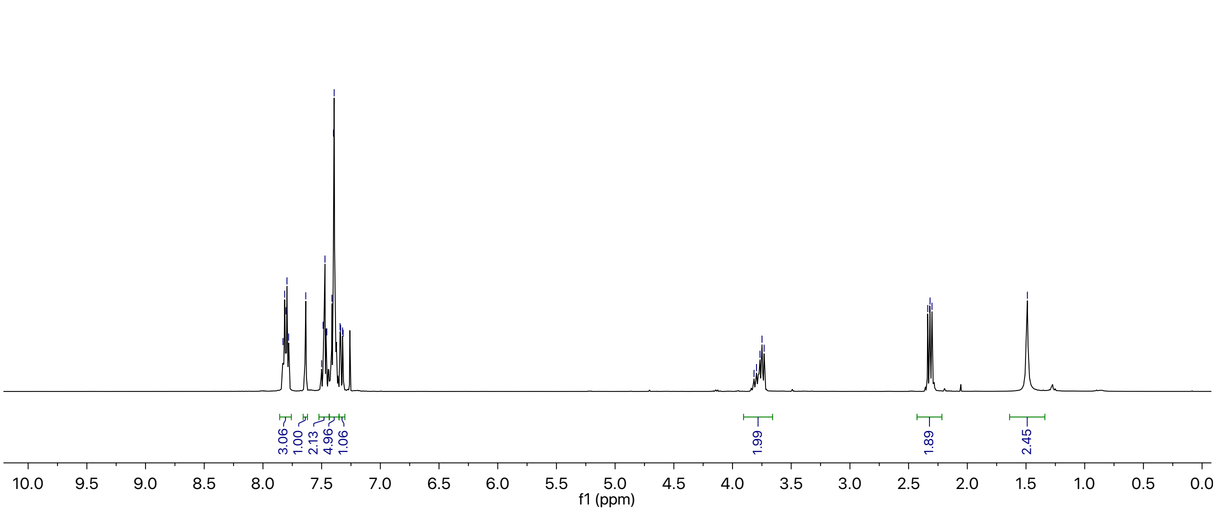

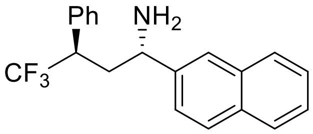

1*S*,3*R*-6f
^{13}C NMR, $CDCl_3$, 100 MHz

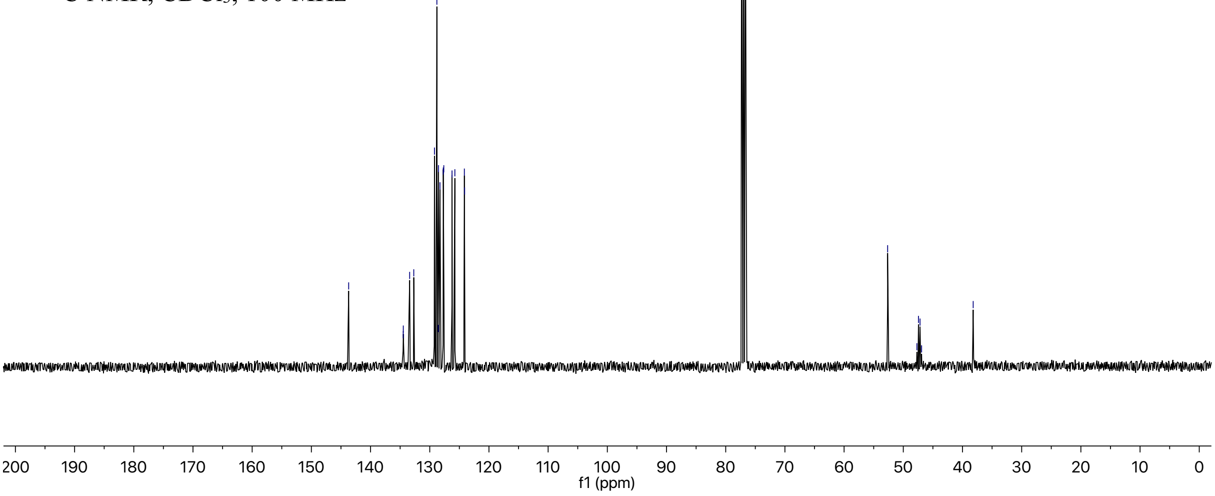

tert-Butyl ((1_S_,3_R_)-4,4,4-trifluoro-1-(naphthalen-2-yl)-3-phenylbutyl)carbamate – minor diastereomer (6f’)

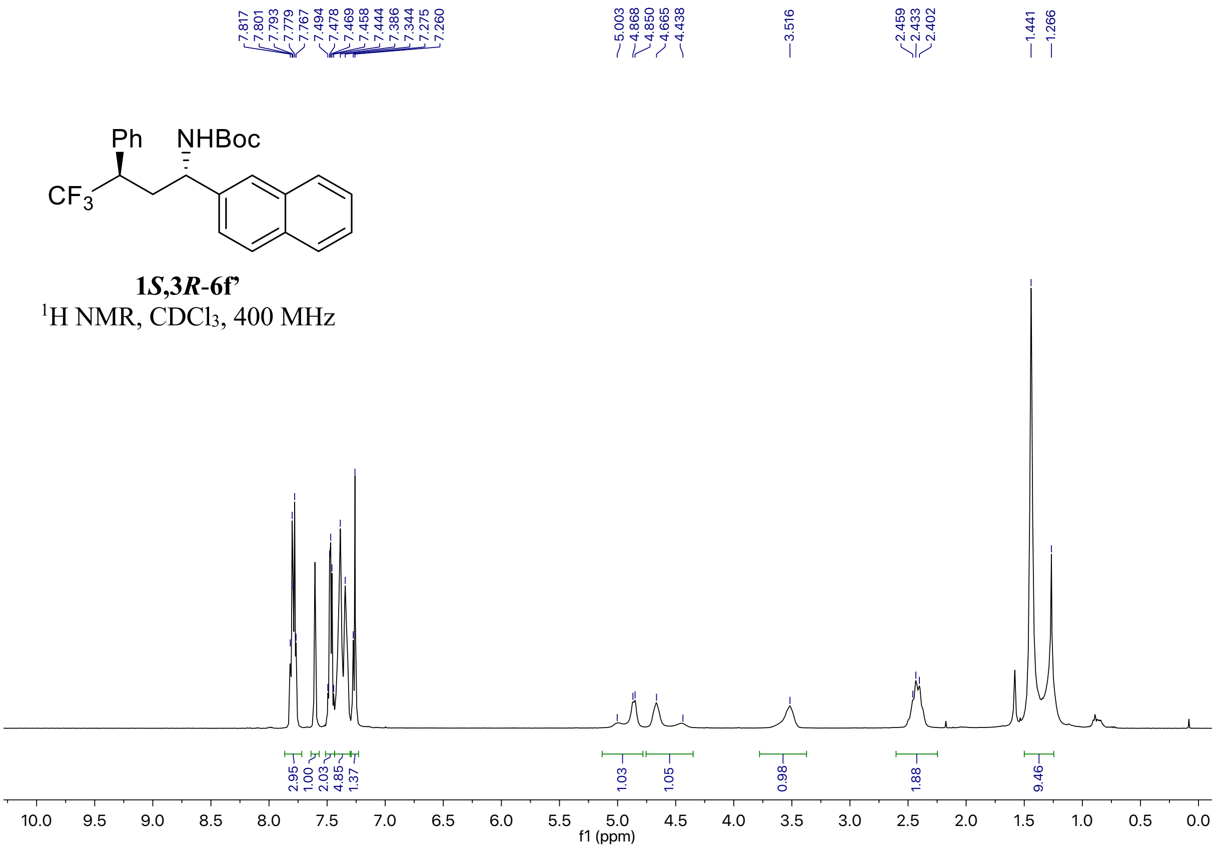

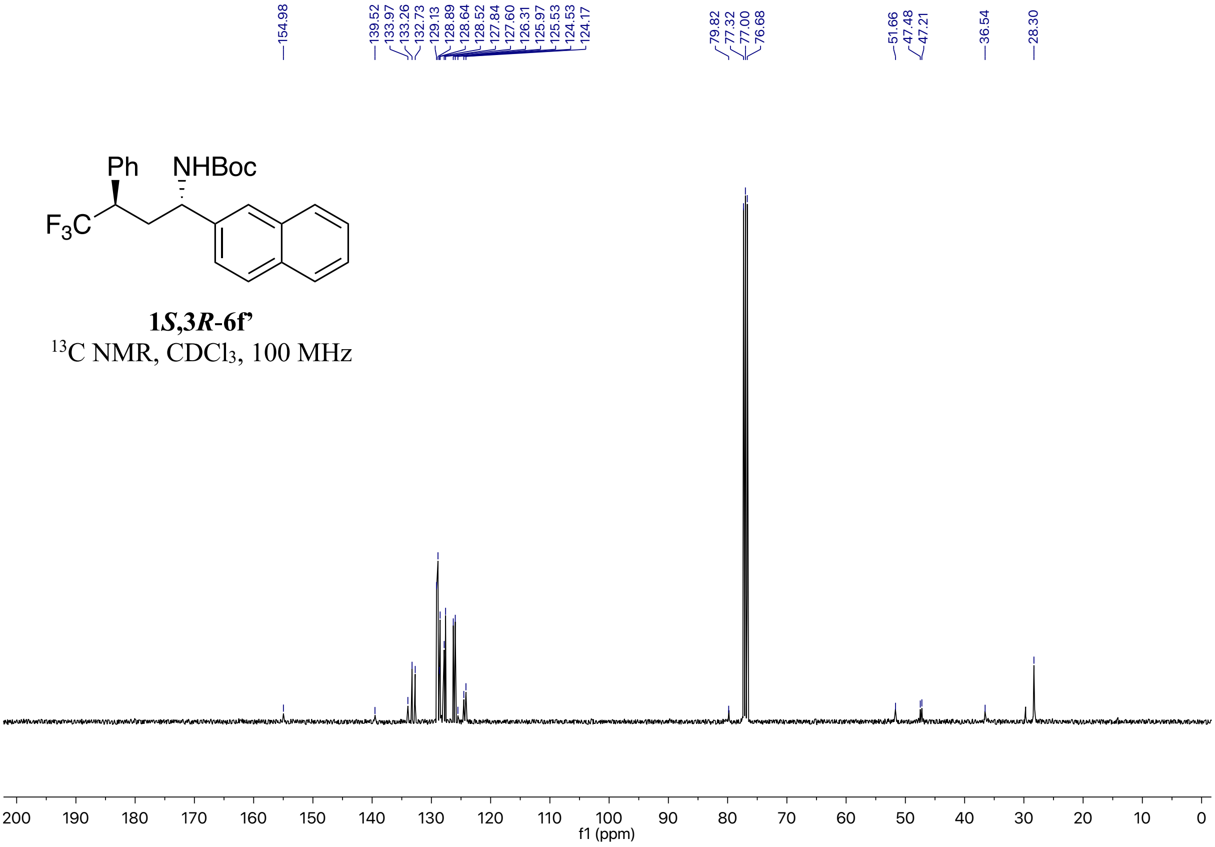

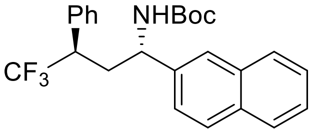

1*S*,3*R*-6f'

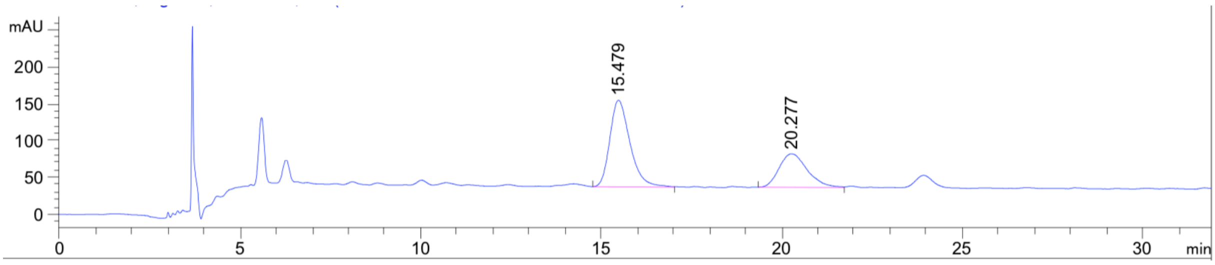

Peak #	RetTime [min]	Type	Width [min]	Area [mAU*s]	Height [mAU]	Area %
1	15.479	BV	0.5814	4568.28809	117.54218	64.8386
2	20.277	VV	0.6730	2477.34546	45.66288	35.1614

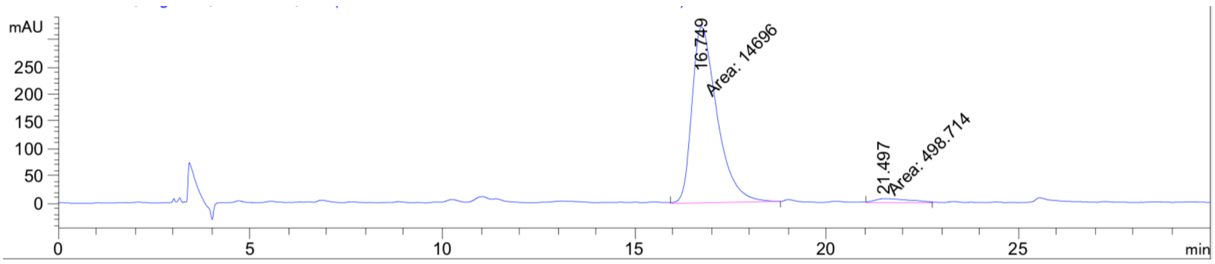

Peak #	RetTime [min]	Type	Width [min]	Area [mAU*s]	Height [mAU]	Area %
1	16.749	MM	0.7586	1.46960e4	322.86279	96.7179
2	21.497	MM	1.0989	498.71426	7.56412	3.2821

(1*R*,3*R*)-4,4,4-Trifluoro-1-(naphthalen-2-yl)-3-phenylbutan-1-amine – major diastereomer (6f)

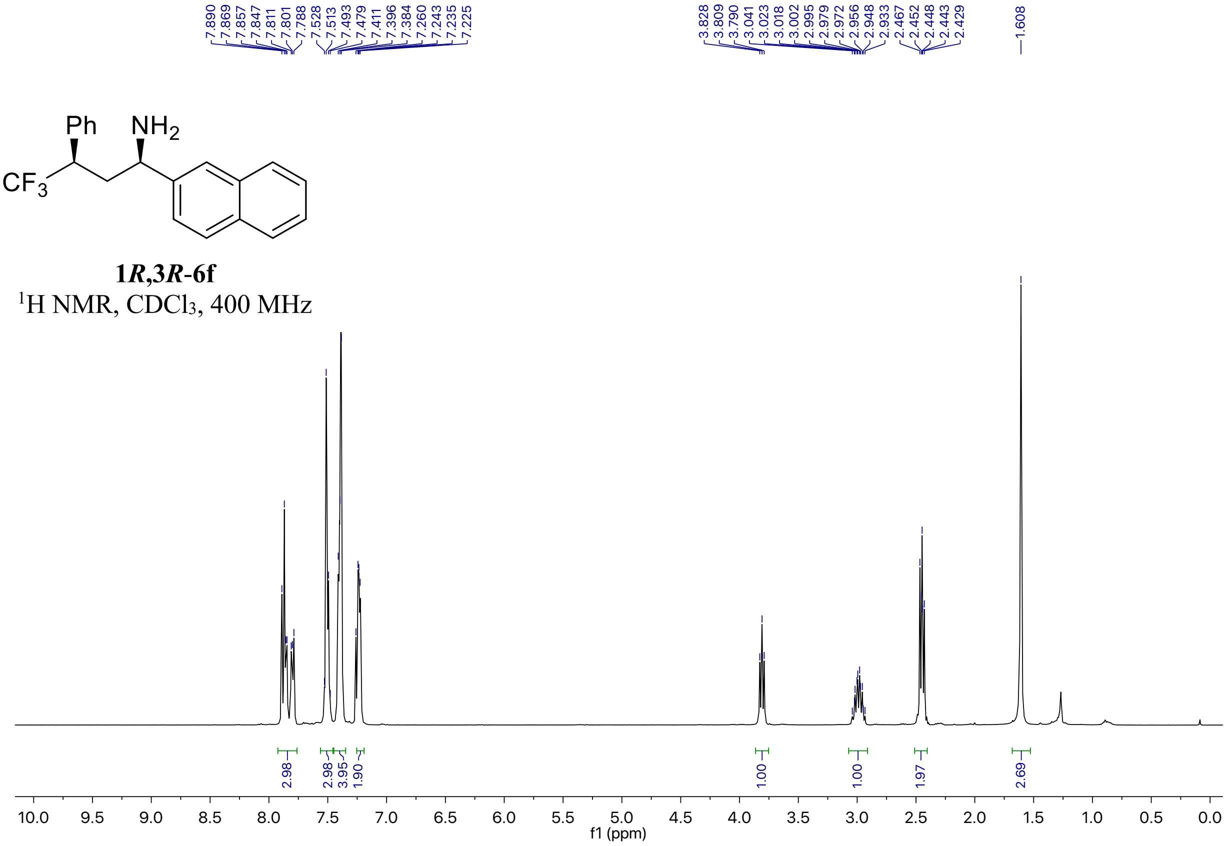

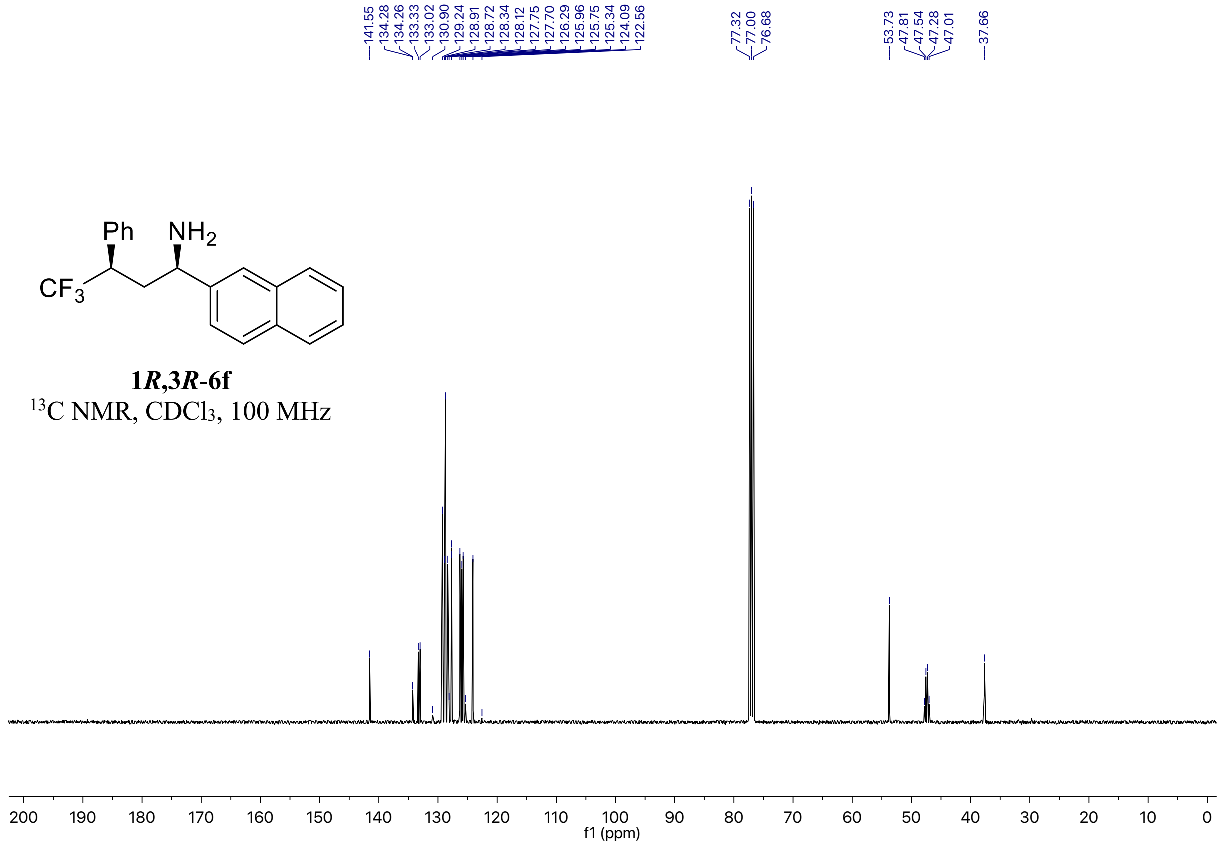

tert-Butyl ((1_R_,3_R_)-4,4,4-trifluoro-1-(naphthalen-2-yl)-3-phenylbutyl)carbamate – major diastereomer (6f')

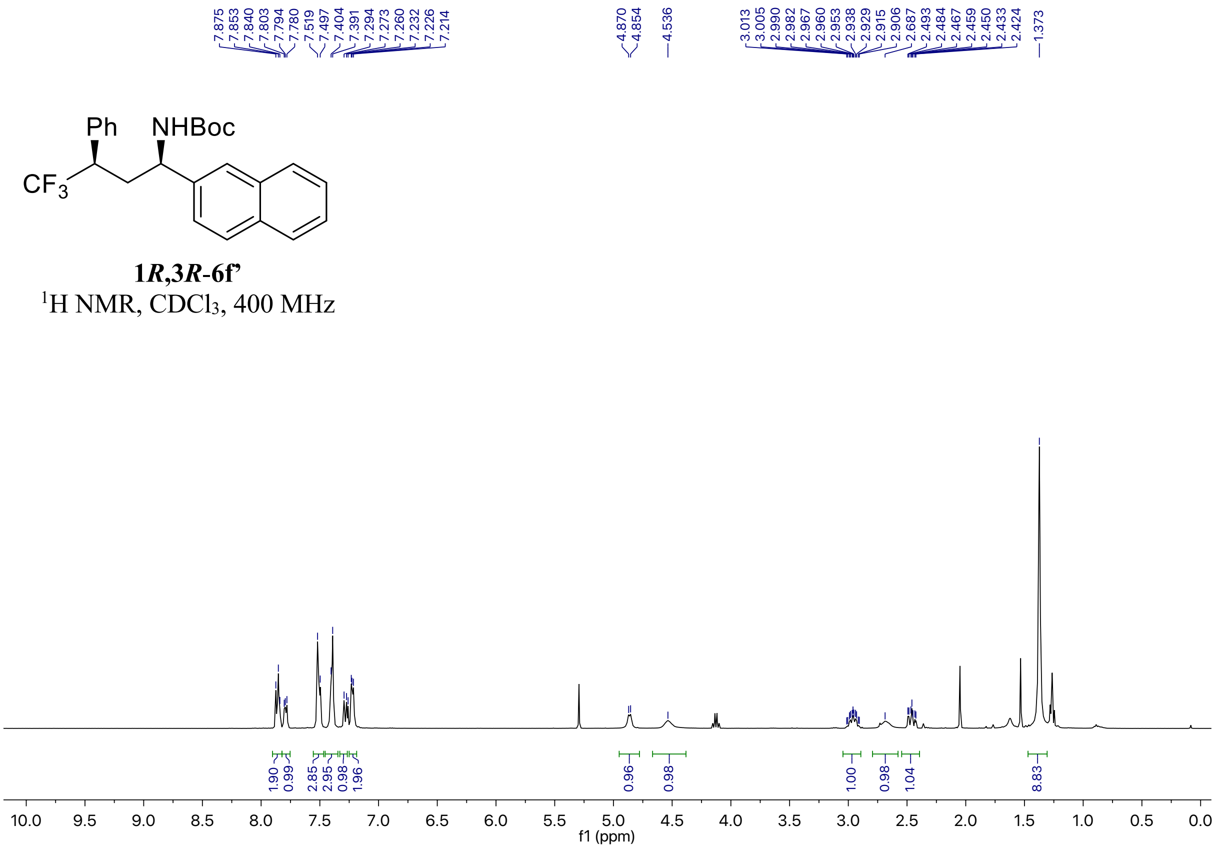

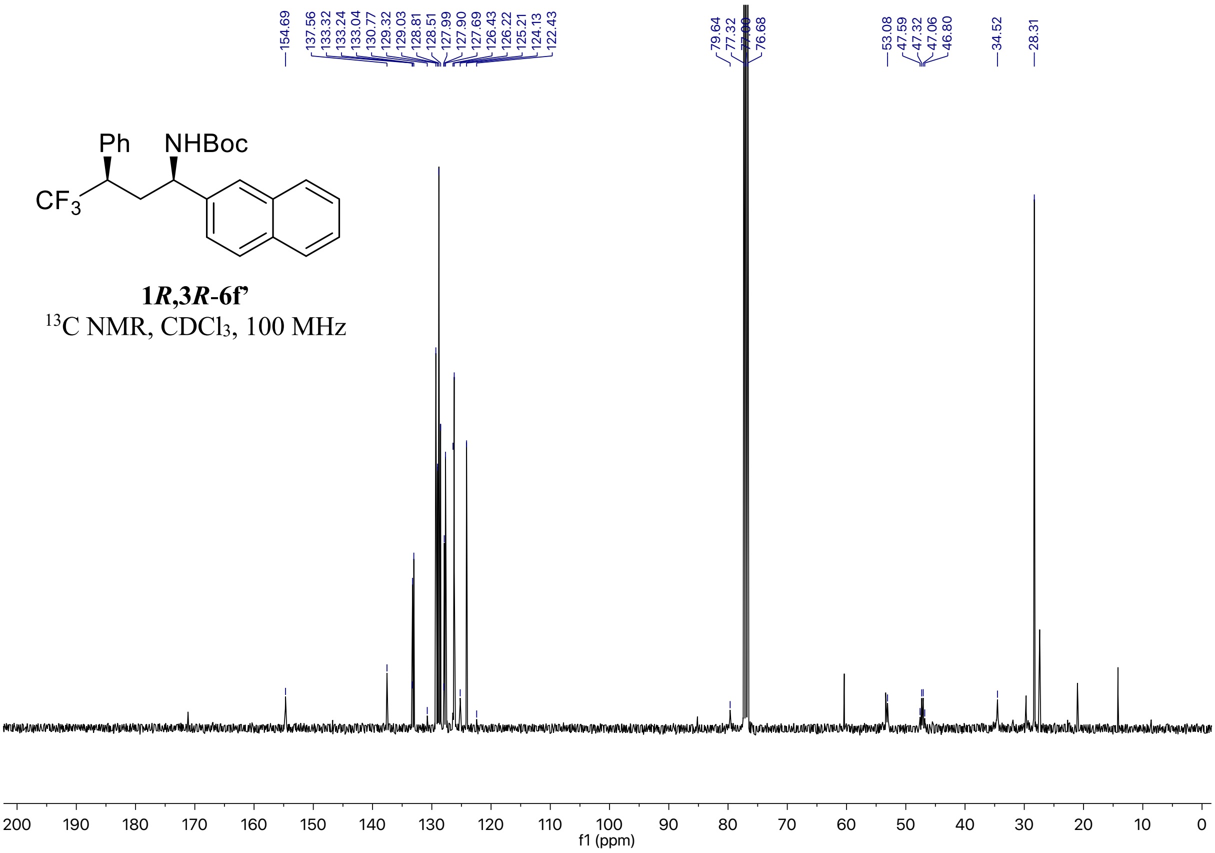

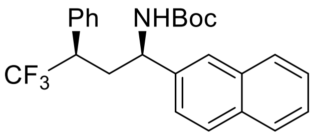

1*R*,3*R*-6f'

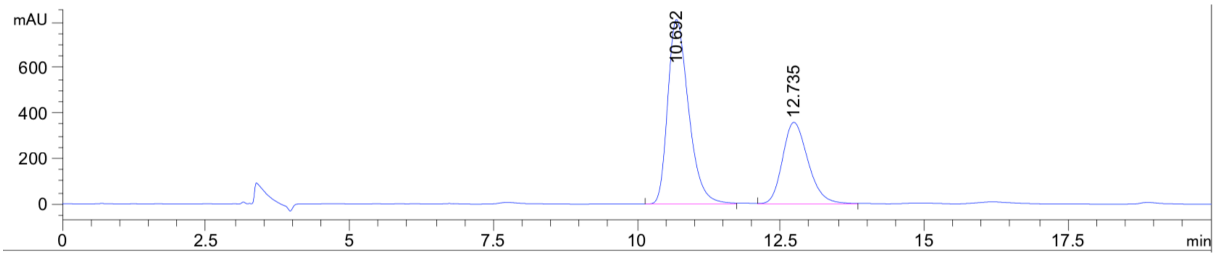

Peak #	RetTime [min]	Type	Width [min]	Area [mAU*s]	Height [mAU]	Area %
1	10.692	BV	0.3884	2.05084e4	812.80261	64.8635
2	12.735	VV	0.4781	1.11093e4	359.93011	35.1365

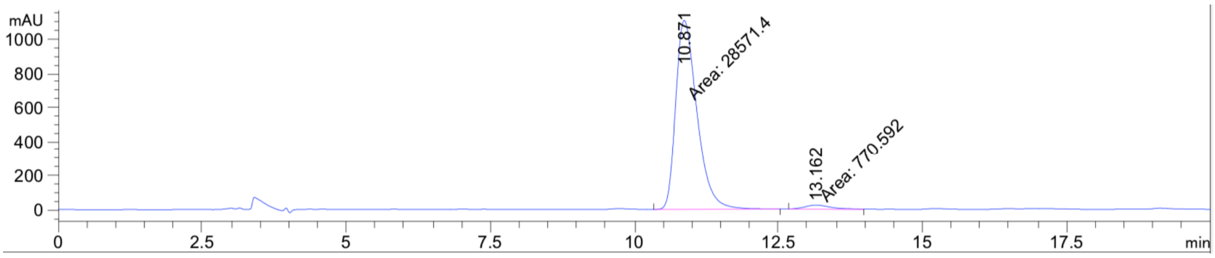

Peak #	RetTime [min]	Type	Width [min]	Area [mAU*s]	Height [mAU]	Area %
1	10.871	MM	0.4292	2.85714e4	1109.36584	97.3738
2	13.162	MM	0.5435	770.59174	23.63230	2.6262

(1*S*,3*R*)-4,4,4-Trifluoro-1-(3-methoxyphenyl)-3-phenylbutan-1-amine – minor diastereomer (6g)

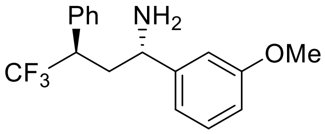

1*S*,3*R*-6g
^{1}H NMR, $CDCl_3$, 400 MHz

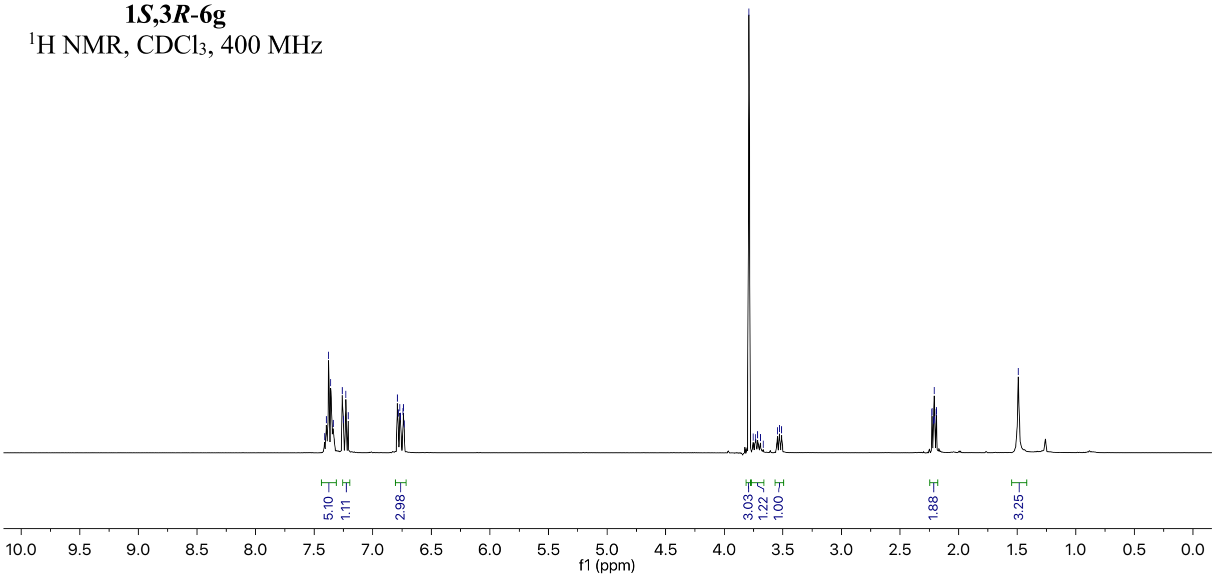

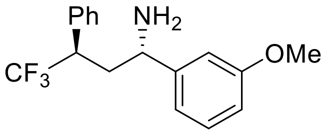

1*S*,3*R*-6g
^{13}C NMR, $CDCl_3$, 100 MHz

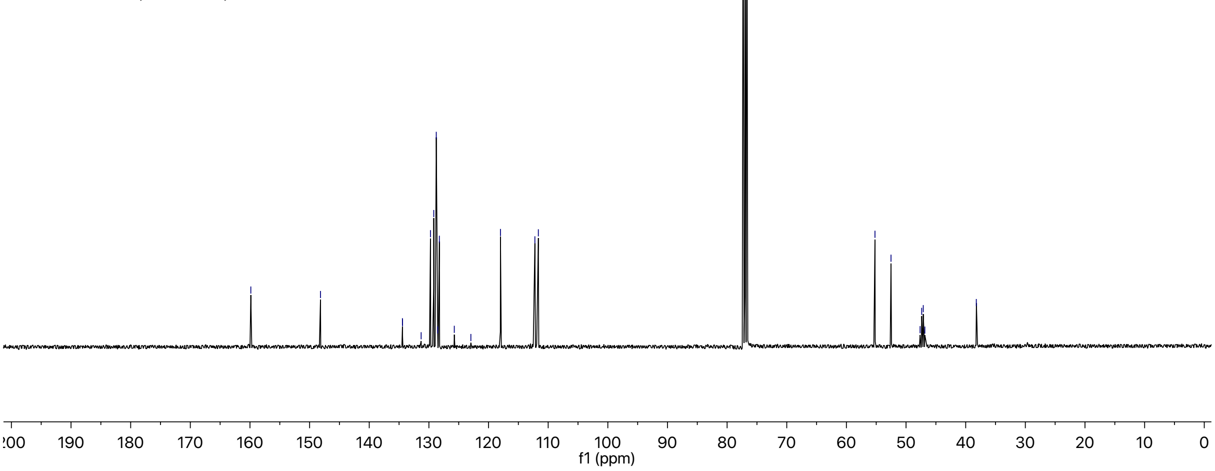

***tert*-Butyl ((1*S*,3*R*)-4,4,4-trifluoro-1-(3-methoxyphenyl)-3-phenylbutyl)carbamate– minor diastereomer (6g')**

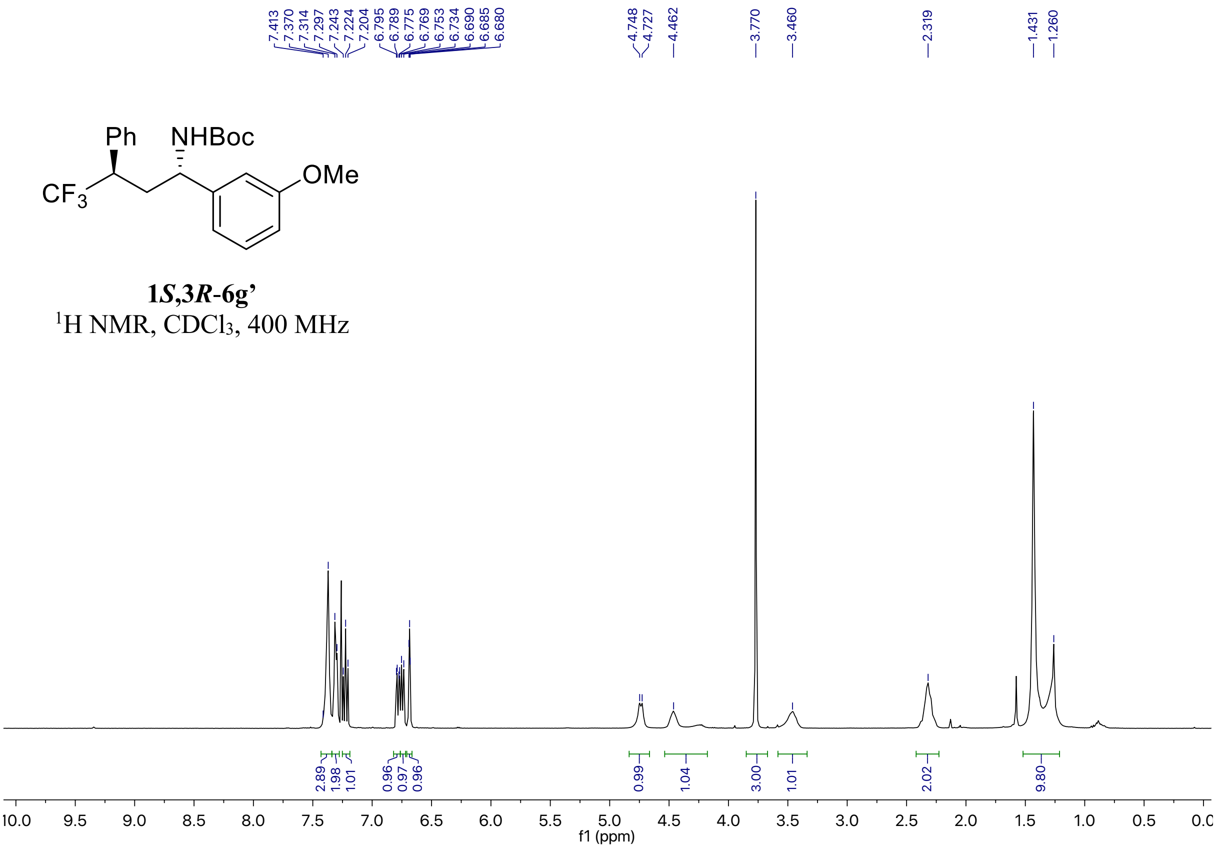

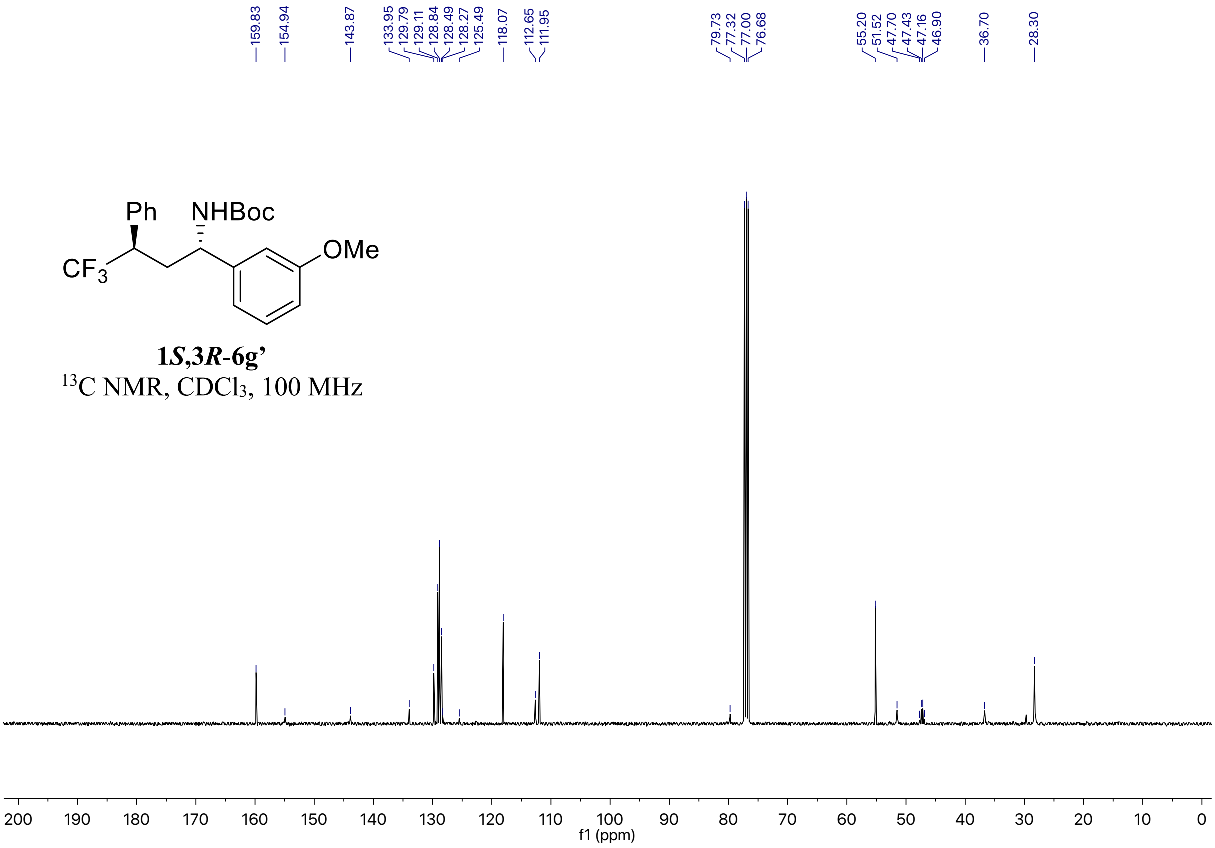

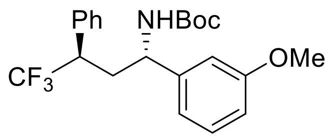

1*S*,3*R*-6f'

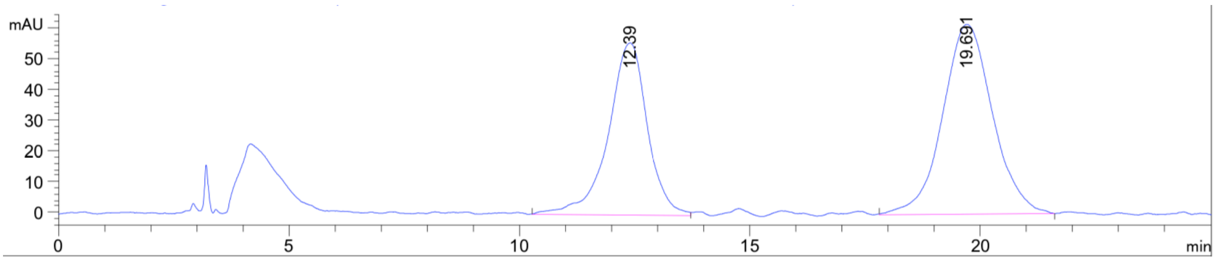

Peak #	RetTime [min]	Type	Width [min]	Area [mAU*s]	Height [mAU]	Area %
1	12.399	VV	0.8562	3308.19751	55.91677	41.6459
2	19.691	VB	0.9560	4635.42969	61.59345	58.3541

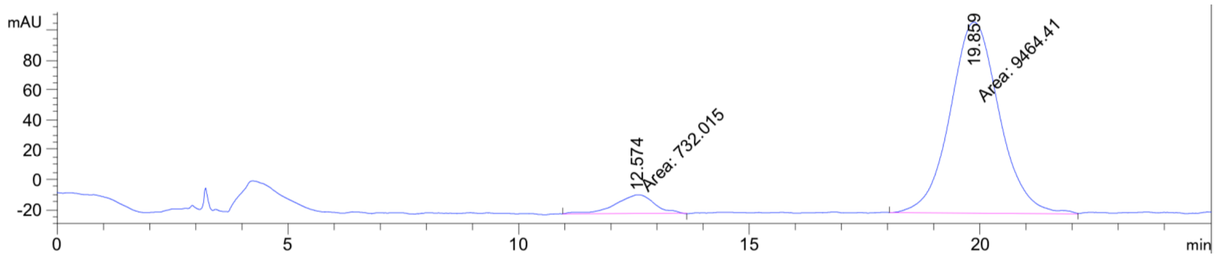

Peak #	RetTime [min]	Type	Width [min]	Area [mAU*s]	Height [mAU]	Area %
1	12.574	MM	0.9768	732.01520	12.49027	7.1791
2	19.859	MM	1.2317	9464.41211	128.07130	92.8209

(1*R*,3*R*)-4,4,4-Trifluoro-1-(3-methoxyphenyl)-3-phenylbutan-1-amine – major diastereomer (6g)

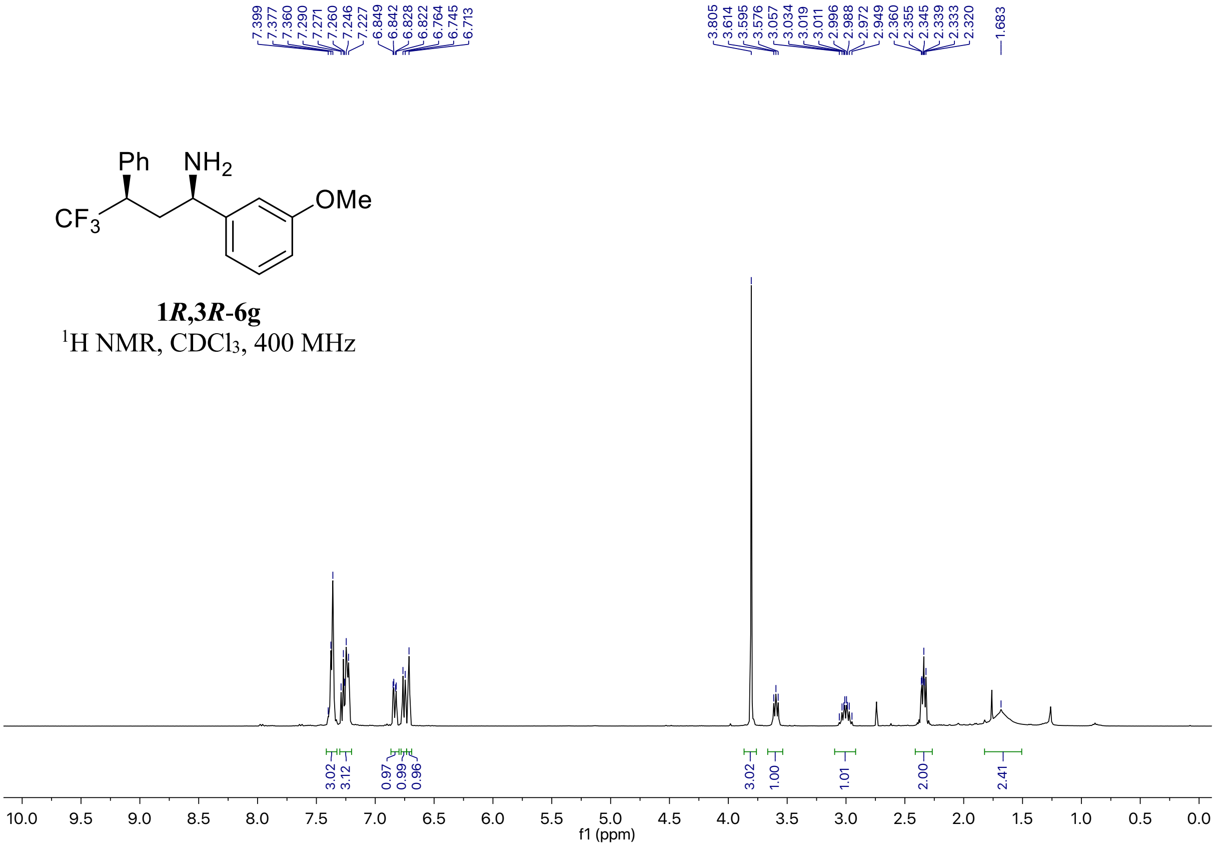

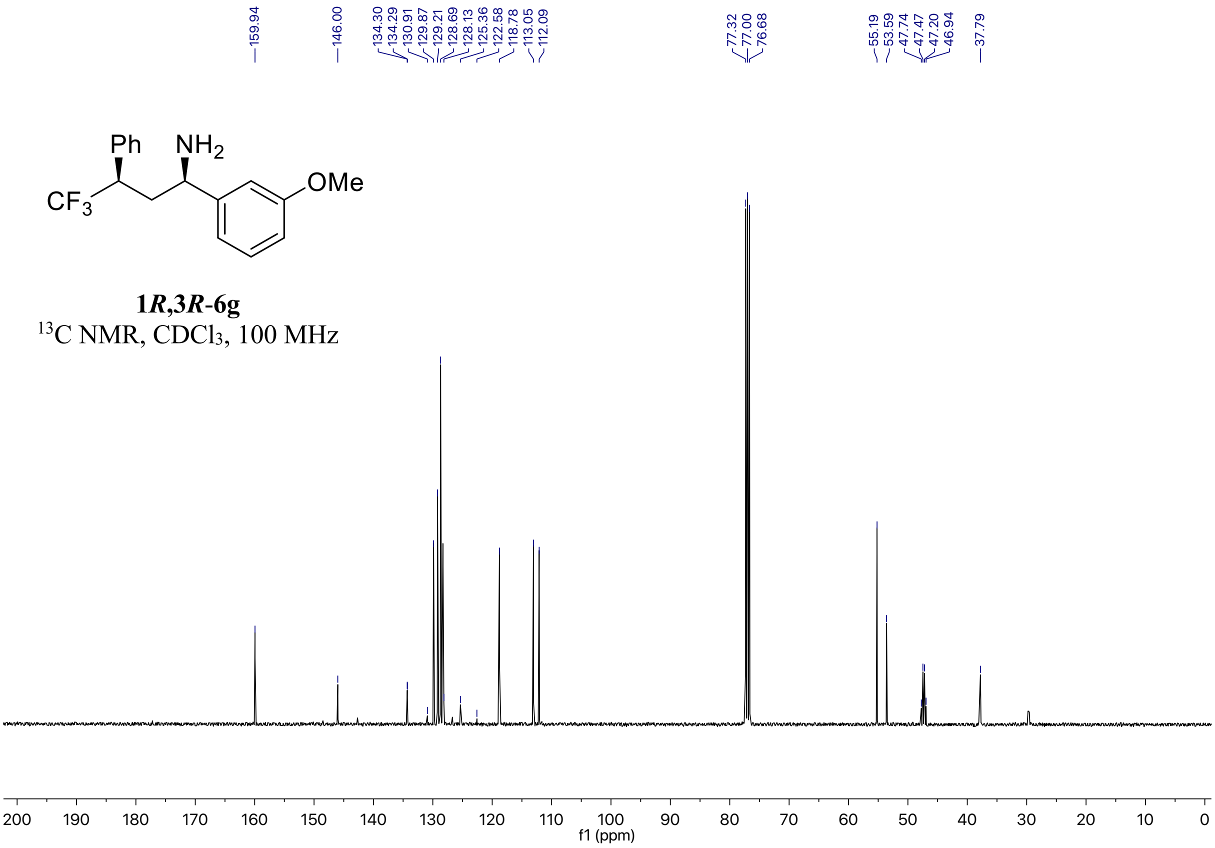

tert-Butyl ((1_R_,3_R_)-4,4,4-trifluoro-1-(3-methoxyphenyl)-3-phenylbutyl)carbamate – major diastereomer (6g')

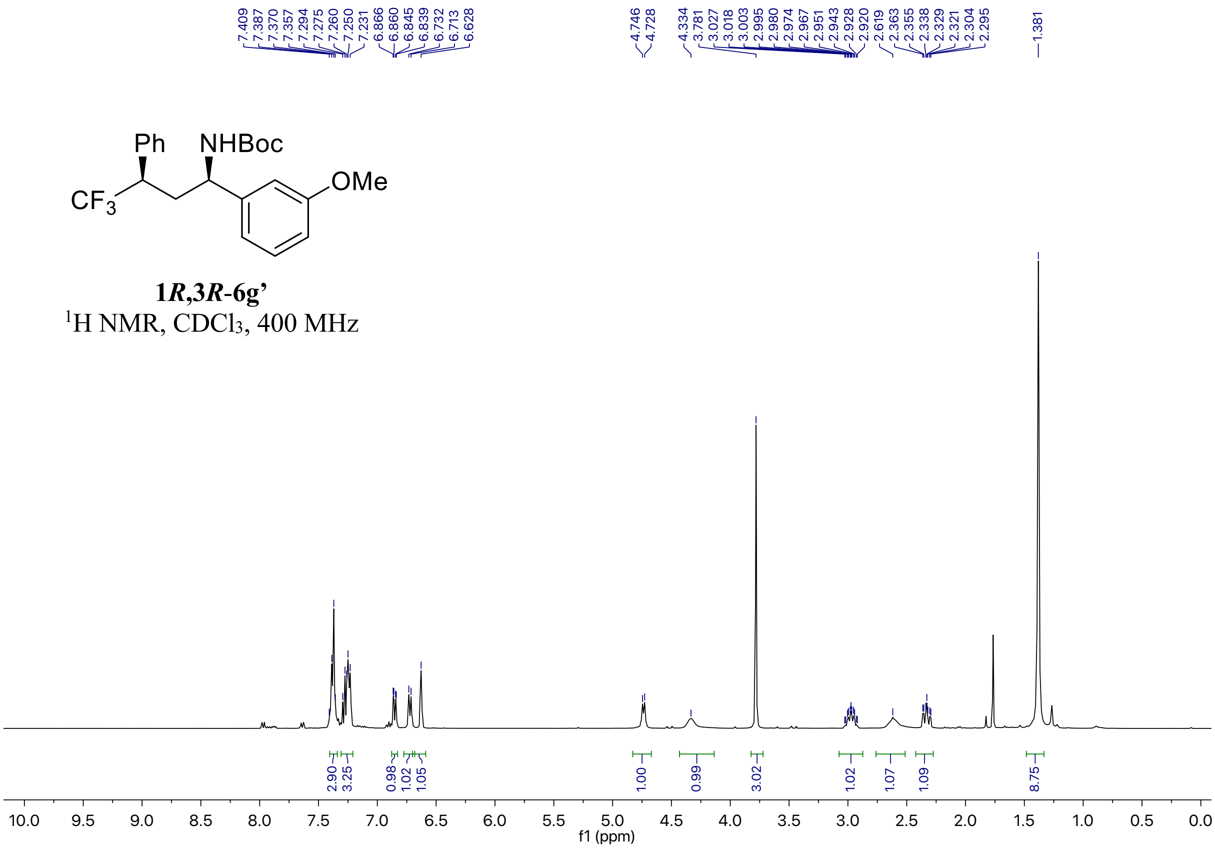

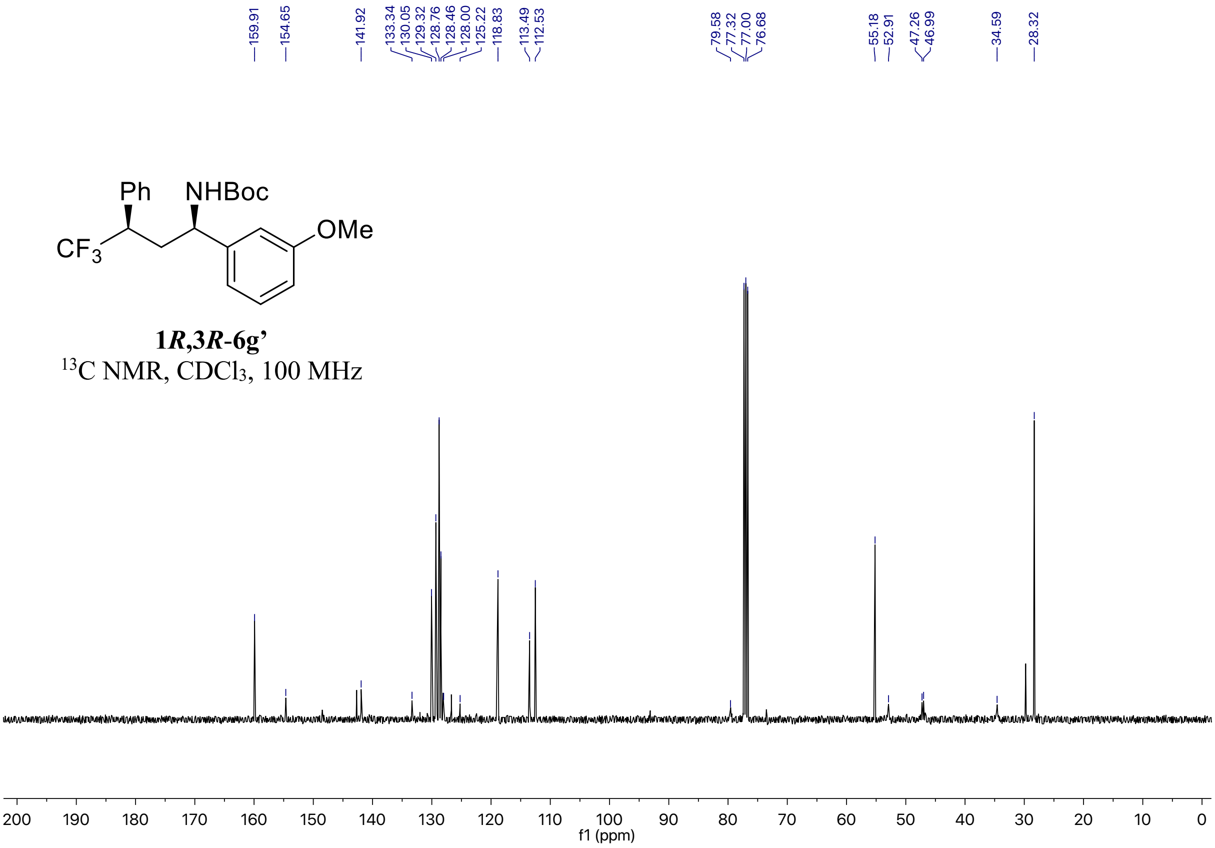

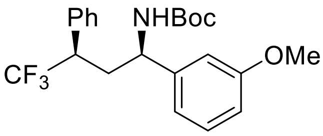

1*R*,3*R*-6g'

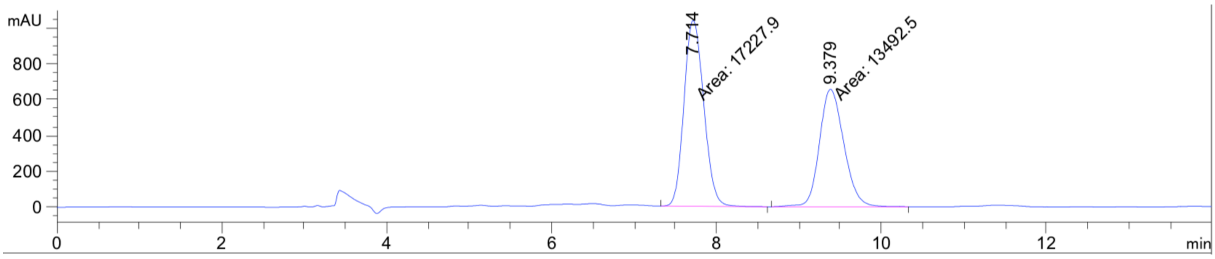

Peak #	RetTime [min]	Type	Width [min]	Area [mAU*s]	Height [mAU]	Area %
1	7.714	MM	0.2774	1.72279e4	1035.24622	56.0796
2	9.379	MM	0.3435	1.34925e4	654.59137	43.9204

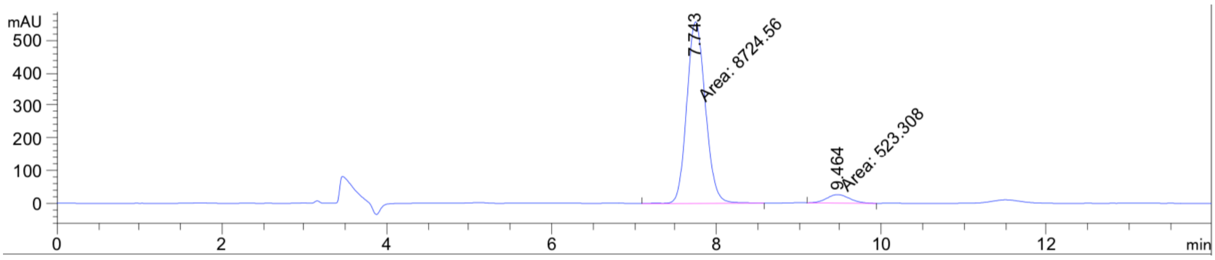

Peak #	RetTime [min]	Type	Width [min]	Area [mAU*s]	Height [mAU]	Area %
1	7.743	MM	0.2611	8724.56250	557.00623	94.3413
2	9.464	MM	0.3294	523.30811	26.47727	5.6587

(1*S*,3*R*)-4,4,4-Trifluoro-3-phenyl-1-(3-(trifluoromethyl)phenyl)butan-1-amine – minor diastereomer (6h)

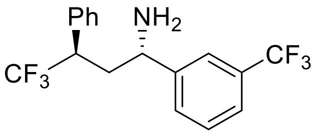

1*S*,3*R*-6h
^{1}H NMR, CDCl$_3$, 400 MHz

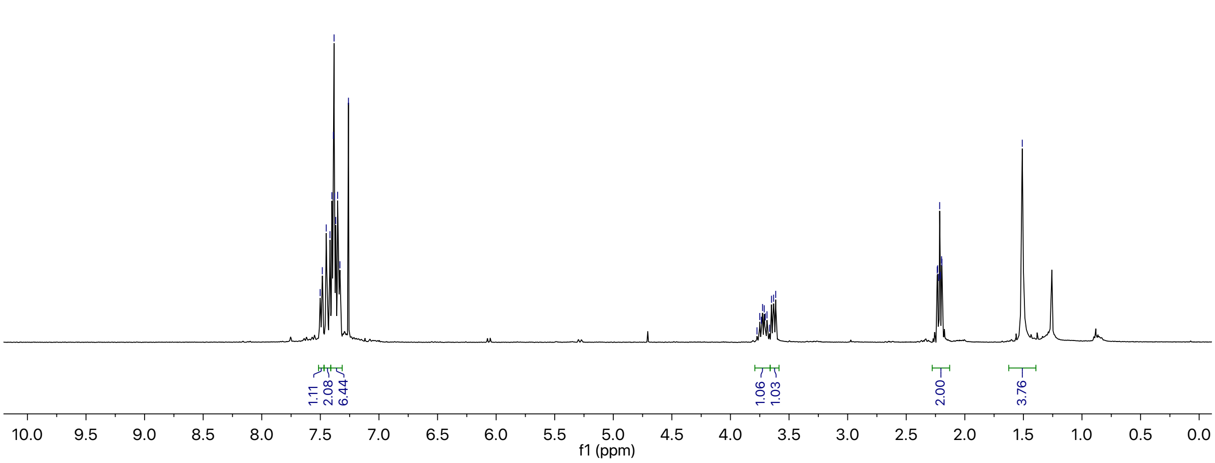

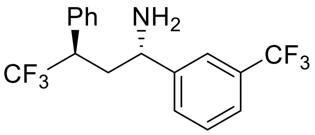

1*S*,3*R*-6h
^{13}C NMR, CDCl$_3$, 100 MHz

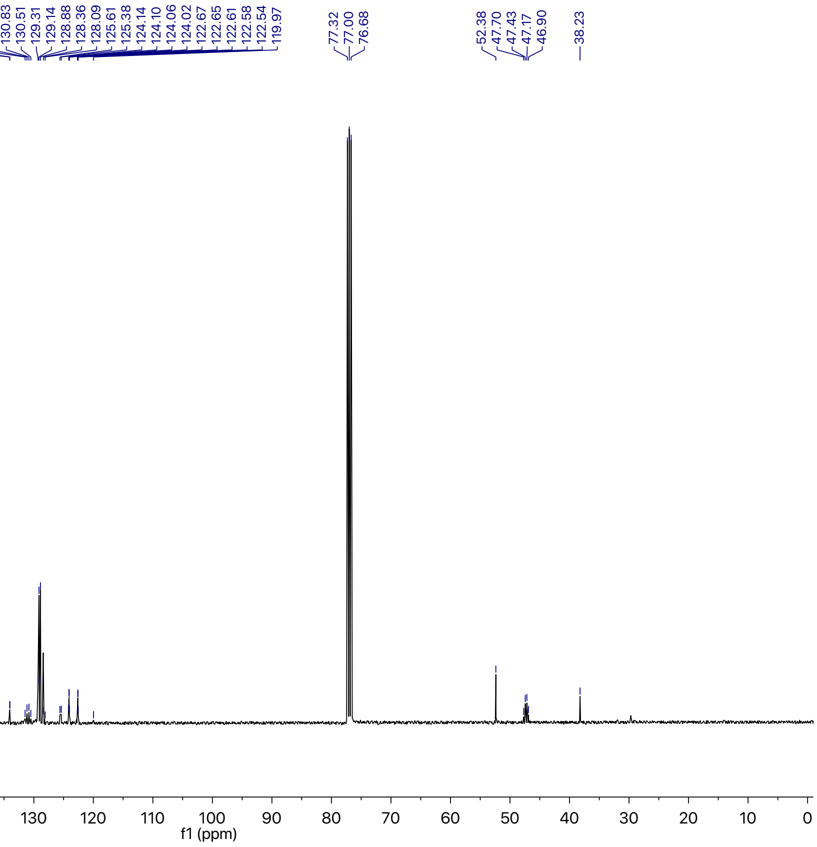

***tert*-Butyl ((1*S*,3*R*)-4,4,4-trifluoro-3-phenyl-1-(3-(trifluoromethyl)phenyl)butyl)carbamate – minor diastereomer (6h’)**

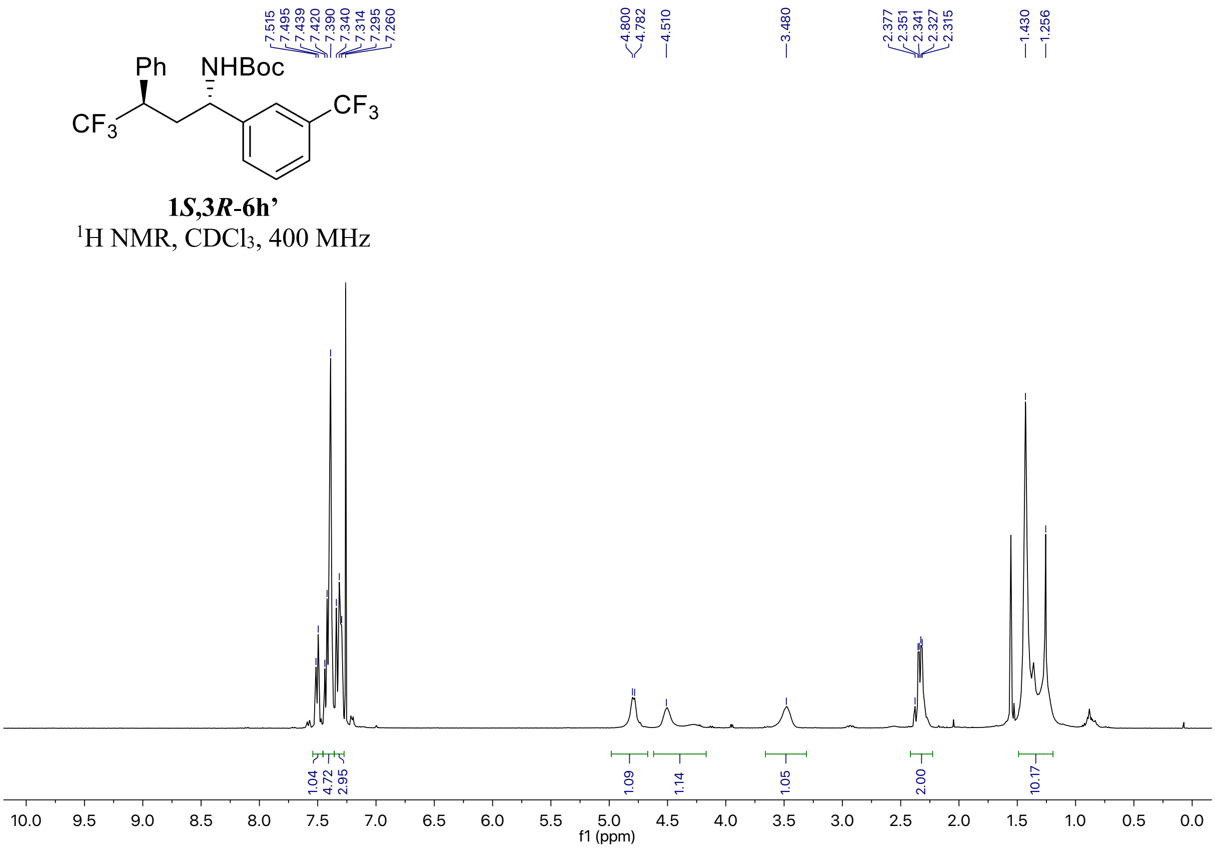

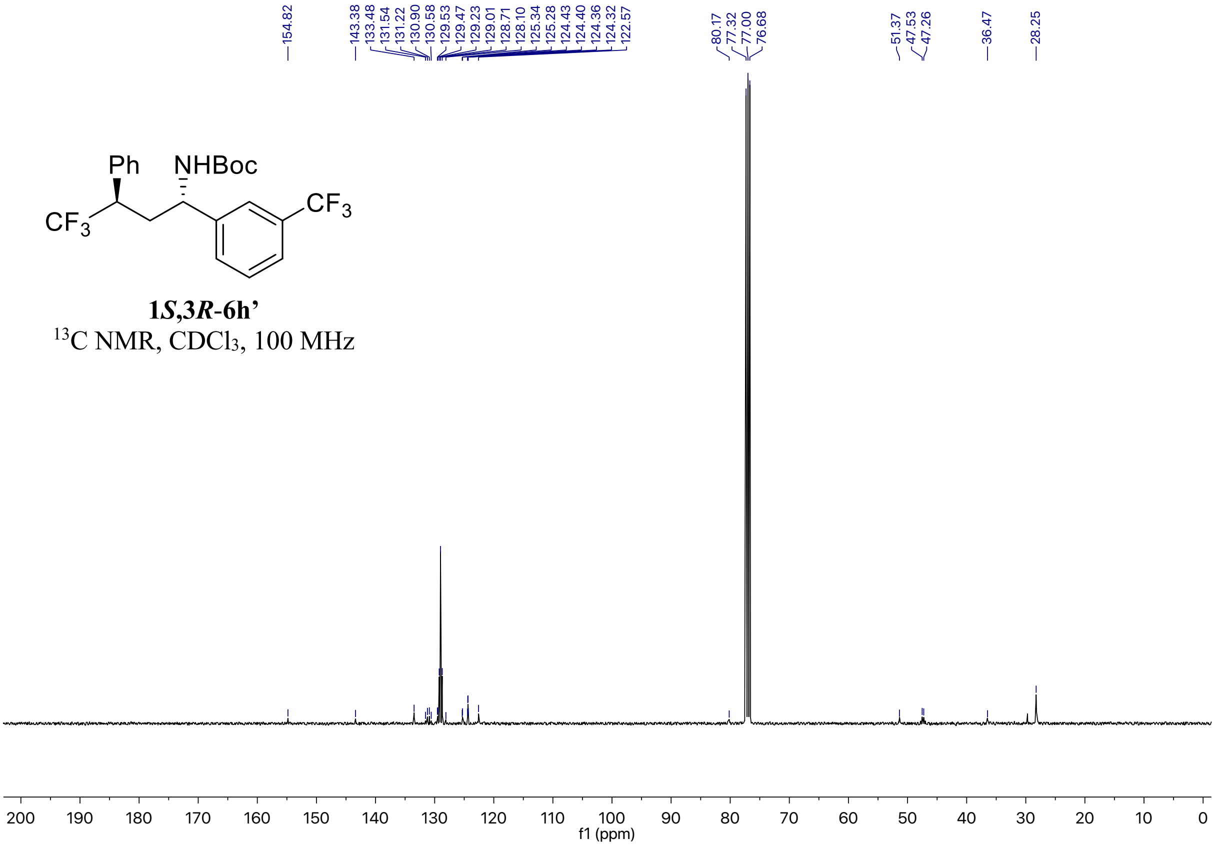

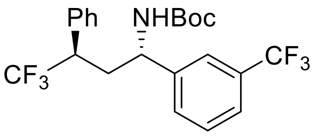

1*S*,3*R*-6h'

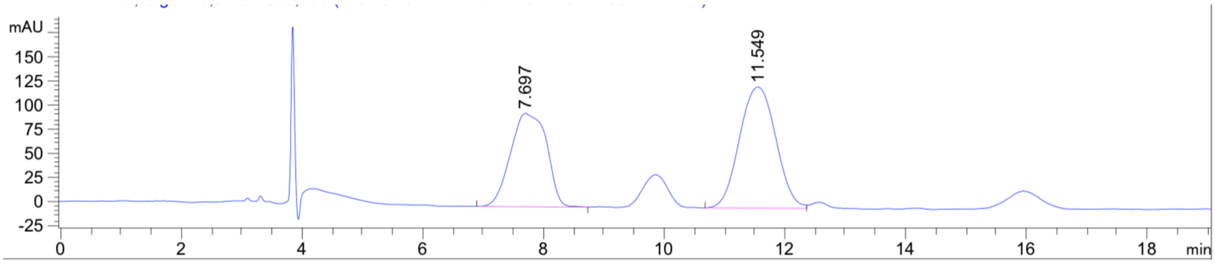

Peak #	RetTime [min]	Type	Width [min]	Area [mAU*s]	Height [mAU]	Area %
1	7.697	BV	0.5645	4053.77100	97.22186	43.4633
2	11.549	BV	0.6677	5273.12109	126.13394	56.5367

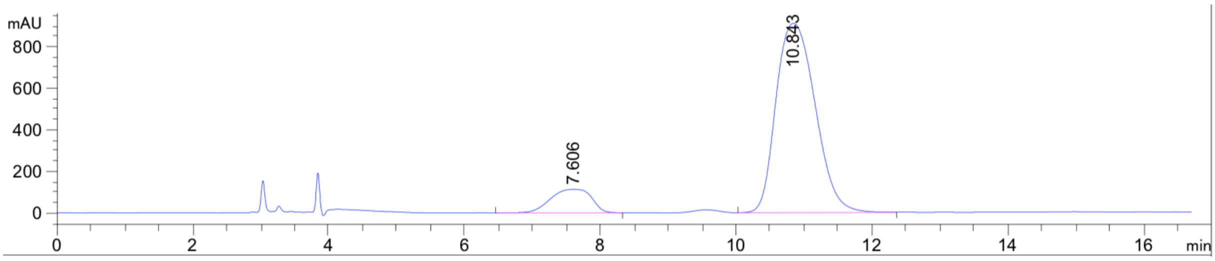

Peak #	RetTime [min]	Type	Width [min]	Area [mAU*s]	Height [mAU]	Area %
1	7.606	BV	0.6001	4758.38721	113.26289	11.5296
2	10.843	BV	0.6418	3.65128e4	910.68823	88.4704

(1*R*,3*R*)-4,4,4-Trifluoro-3-phenyl-1-(3-(trifluoromethyl)phenyl)butan-1-amine – major diastereomer (6h)

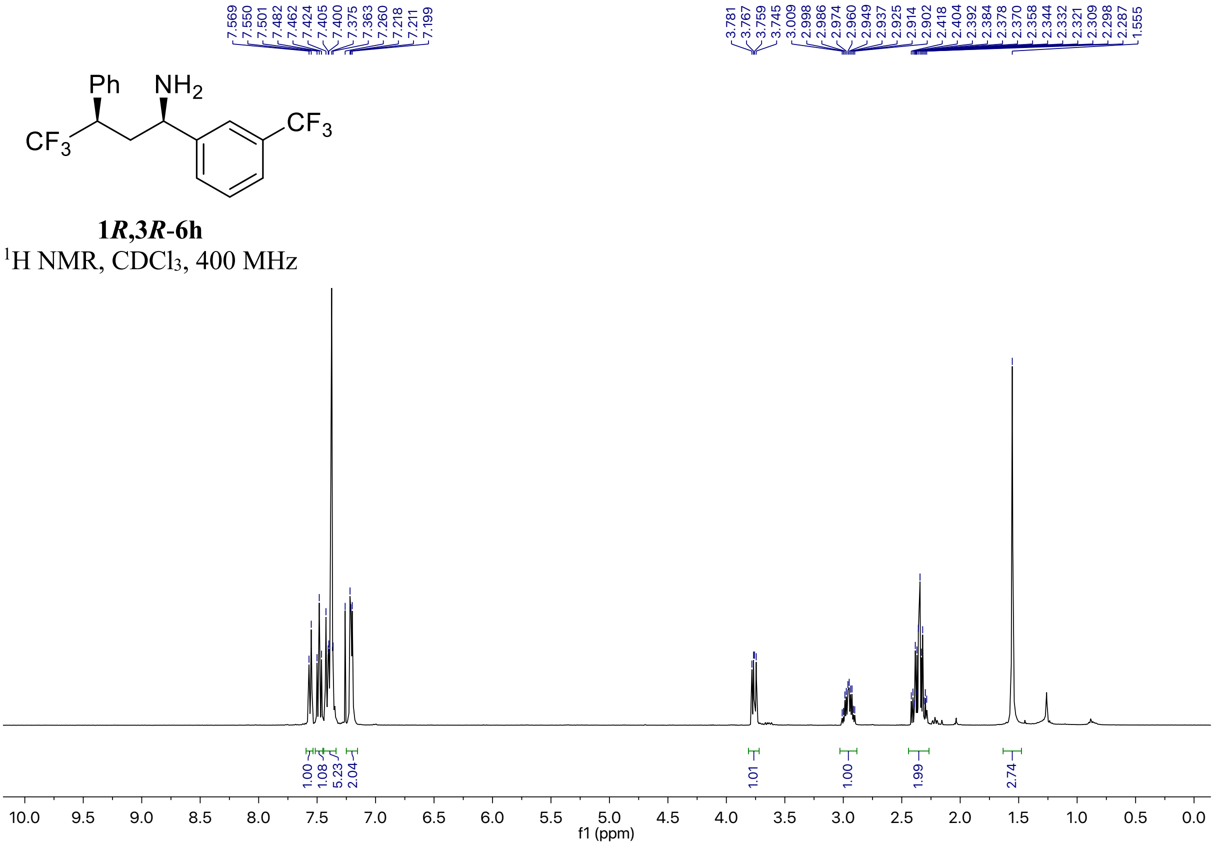

1*R*,3*R*-6h

^{1}H NMR, $CDCl_3$, 400 MHz

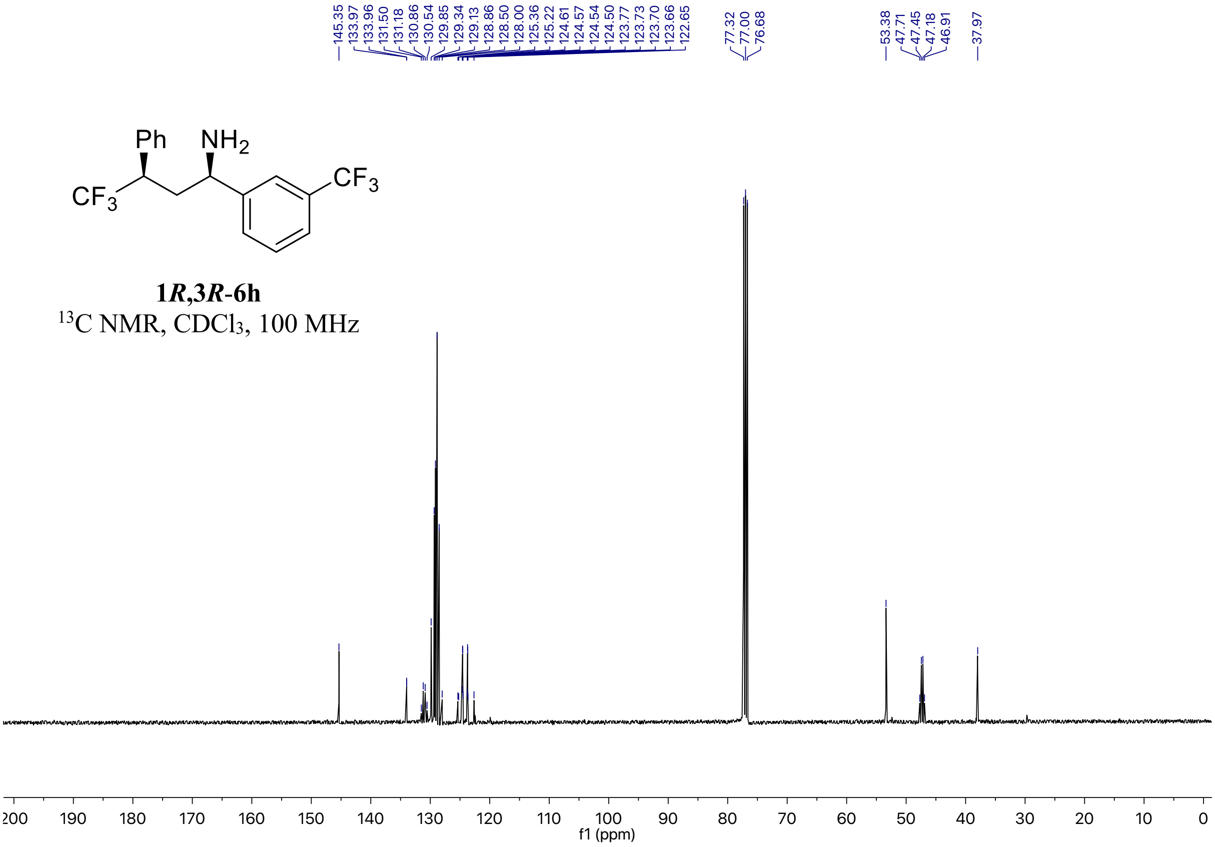

1*R*,3*R*-6h

^{13}C NMR, $CDCl_3$, 100 MHz

tert-Butyl ((1_R_,3_R_)-4,4,4-trifluoro-3-phenyl-1-(3-(trifluoromethyl)phenyl)butyl)carbamate – major diastereomer (6h')

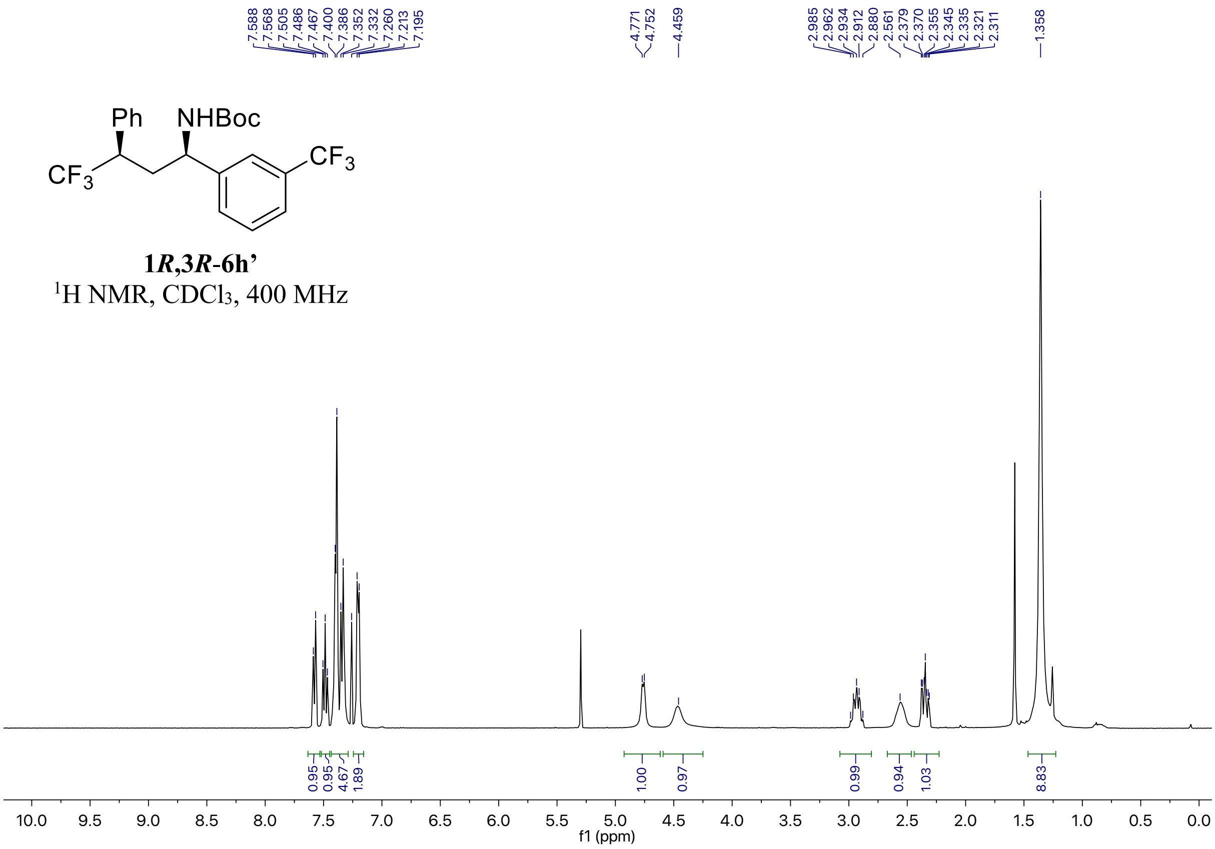

1_R_,3_R_-6h'
^{1}H NMR, $CDCl_3$, 400 MHz

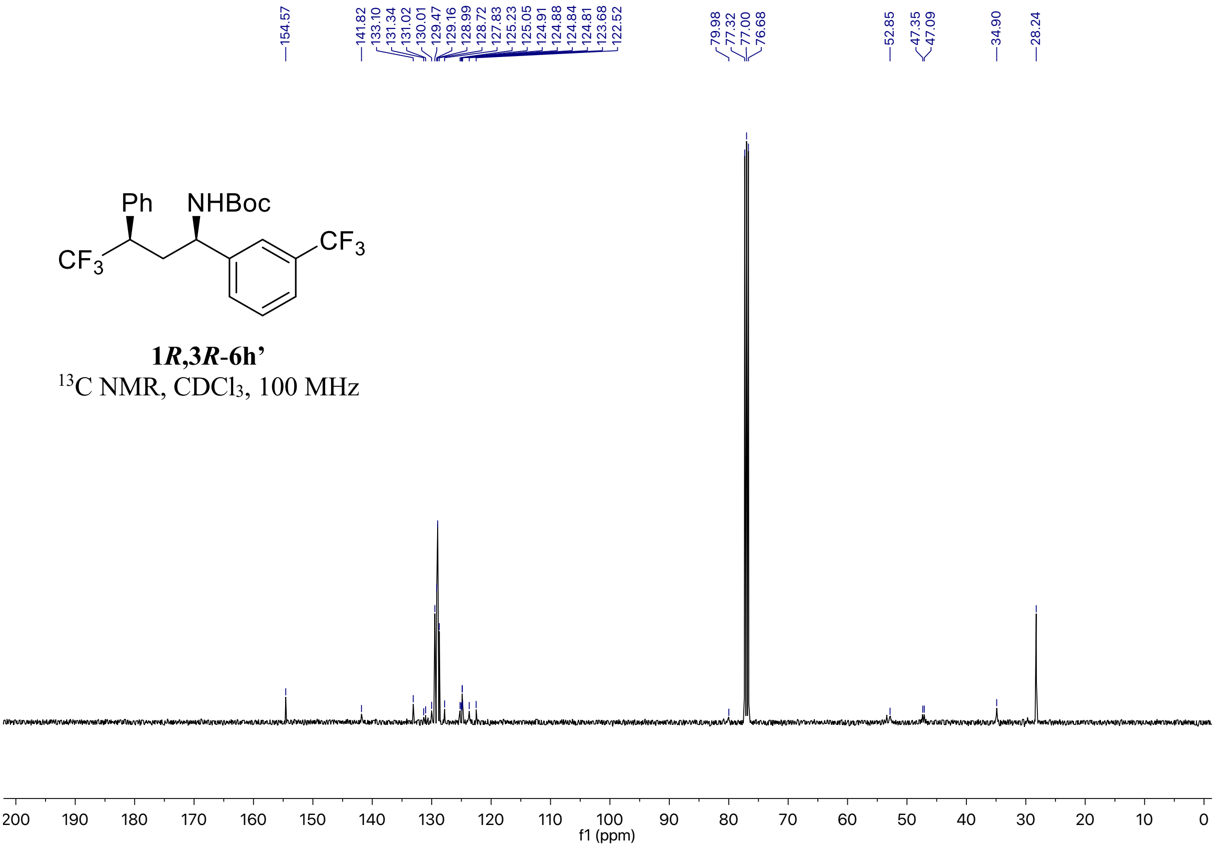

1_R_,3_R_-6h'
^{13}C NMR, $CDCl_3$, 100 MHz

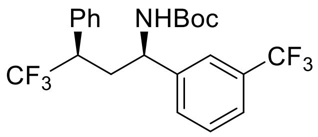

1*R*,3*R*-6h'

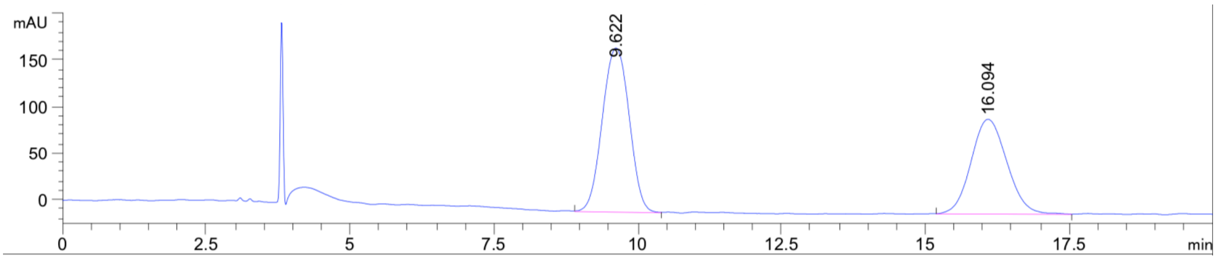

Peak #	RetTime [min]	Type	Width [min]	Area [mAU*s]	Height [mAU]	Area %
1	9.622	VV	0.5197	5738.37500	176.31398	56.4385
2	16.094	VB	0.6646	4429.10303	102.00723	43.5615

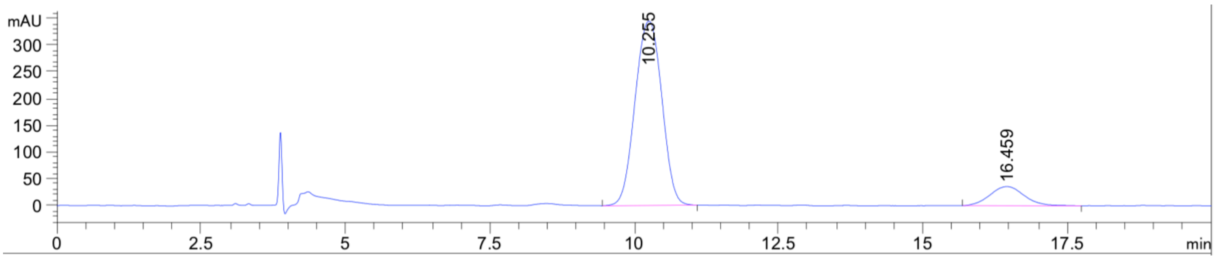

Peak #	RetTime [min]	Type	Width [min]	Area [mAU*s]	Height [mAU]	Area %
1	3.876	VB	0.0626	19.13806	4.59840	5.6458
2	10.256	BB	0.5126	319.83951	9.96070	94.3542

(1*S*,3*R*)-4,4,4-Trifluoro-1-(2-methoxyphenyl)-3-phenylbutan-1-amine - minor diastereomer (6i)

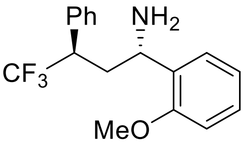

1*S*,3*R*-6i
^{1}H NMR, $CDCl_3$, 400 MHz

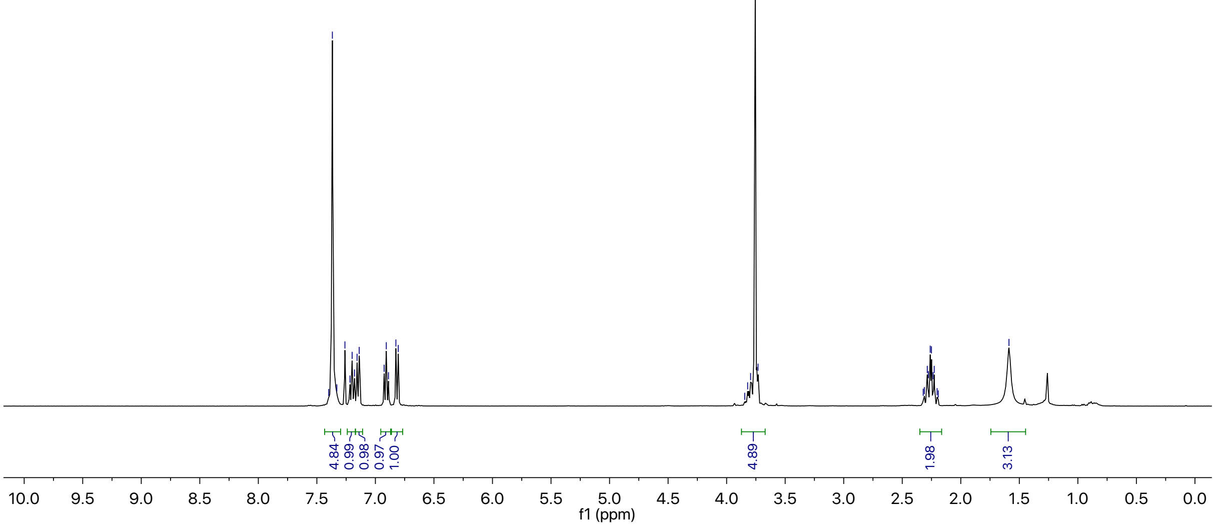

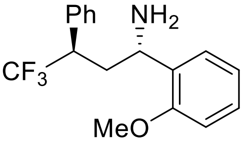

1*S*,3*R*-6i
^{13}C NMR, $CDCl_3$, 100 MHz

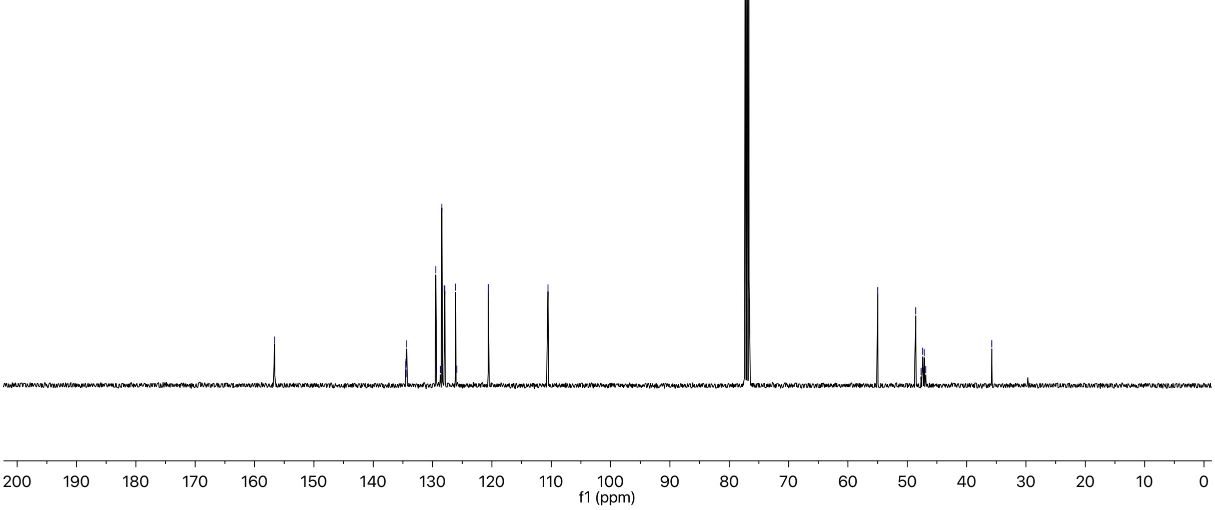

***tert*-Butyl ((1*S*,3*R*)-4,4,4-trifluoro-1-(2-methoxyphenyl)-3-phenylbutyl)carbamate – minor diastereomer (6i')**

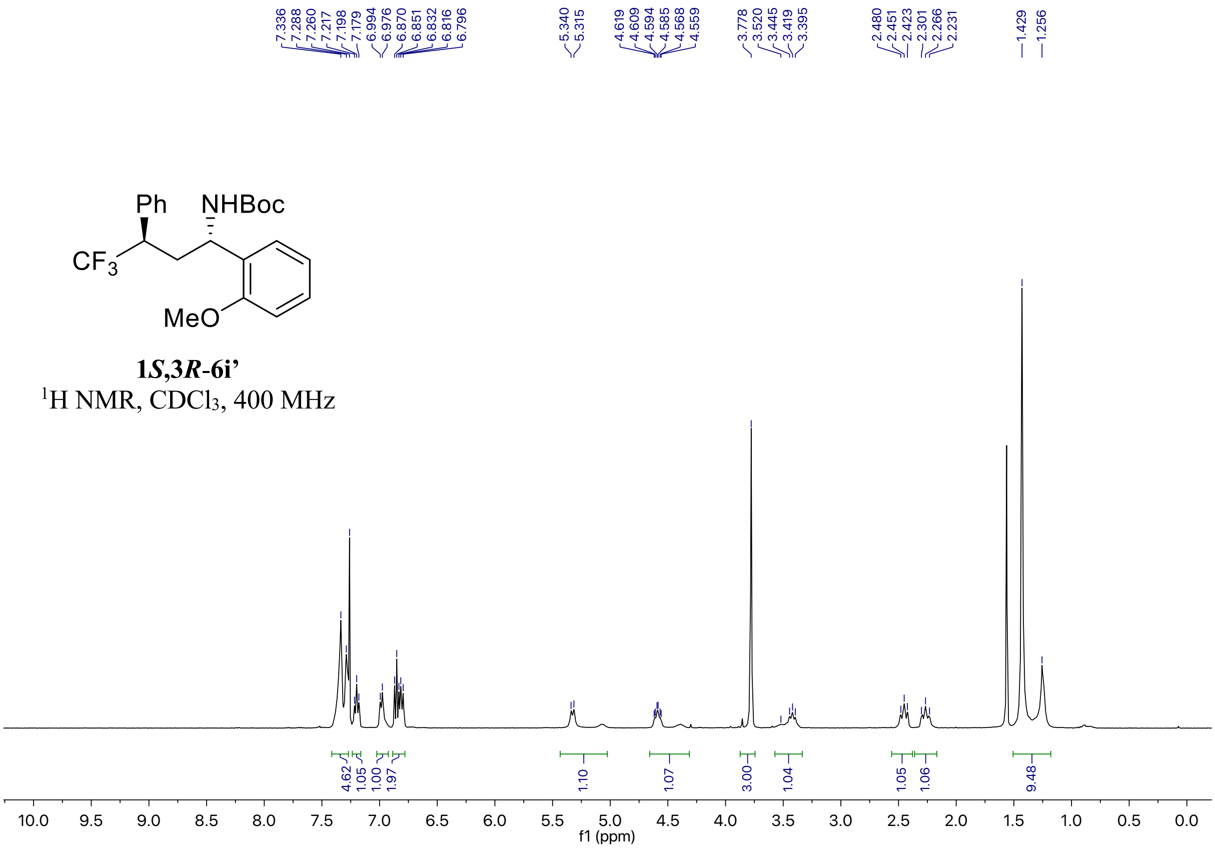

1*S*,3*R*-6i'
^{1}H NMR, $CDCl_3$, 400 MHz

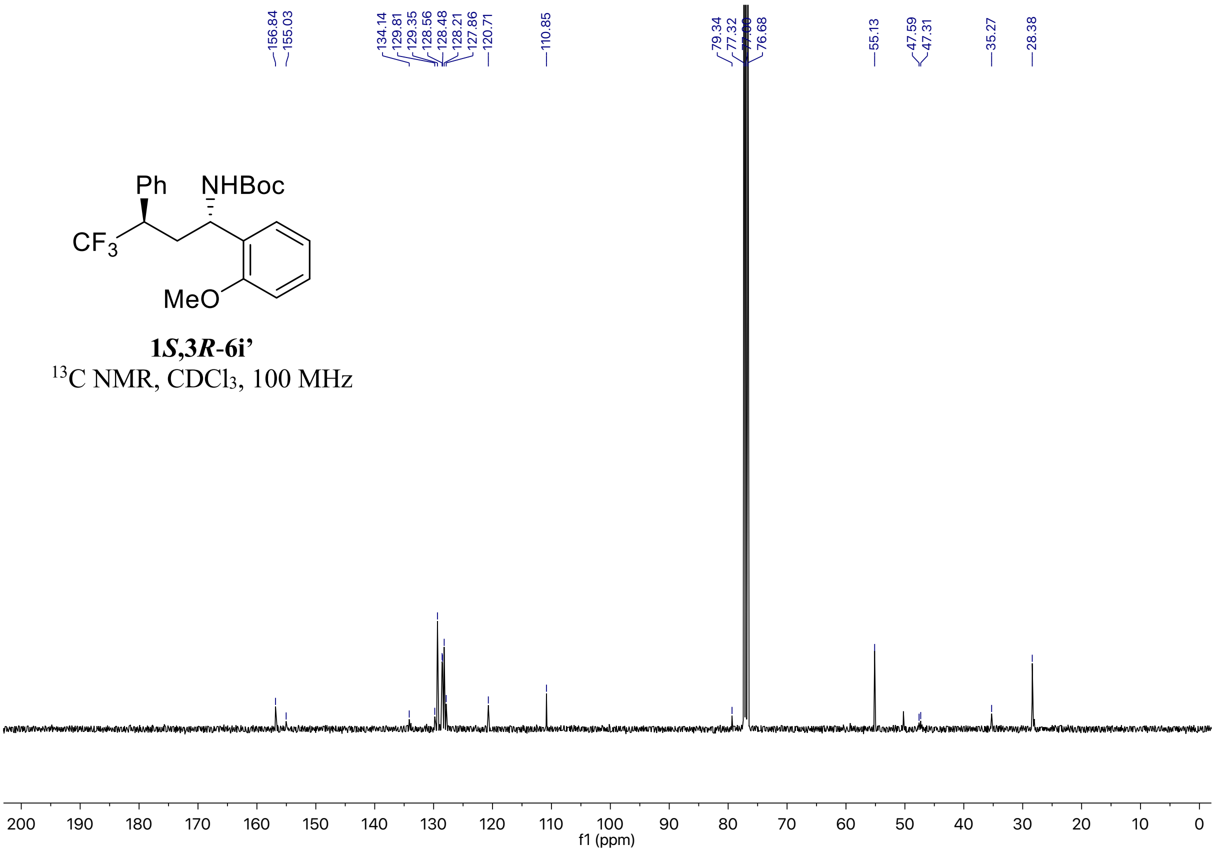

1*S*,3*R*-6i'
^{13}C NMR, $CDCl_3$, 100 MHz

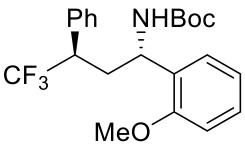

1*S*,3*R*-6i’

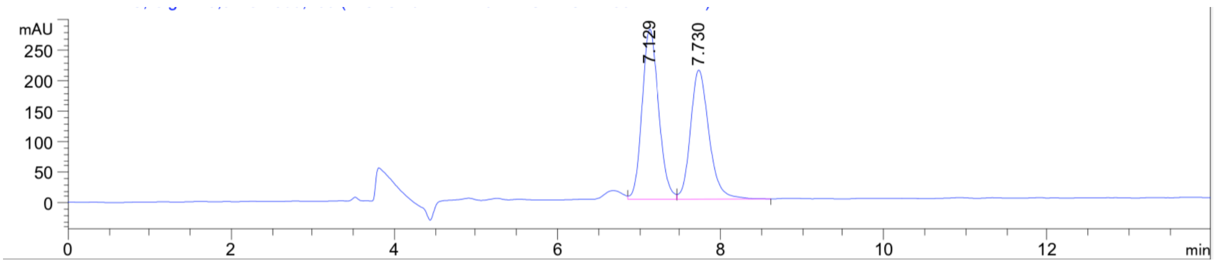

Peak #	RetTime [min]	Type	Width [min]	Area [mAU*s]	Height [mAU]	Area %
1	7.129	VV	0.2209	3949.51587	279.38400	54.1906
2	7.730	VB	0.2418	3338.67139	211.88257	45.8094

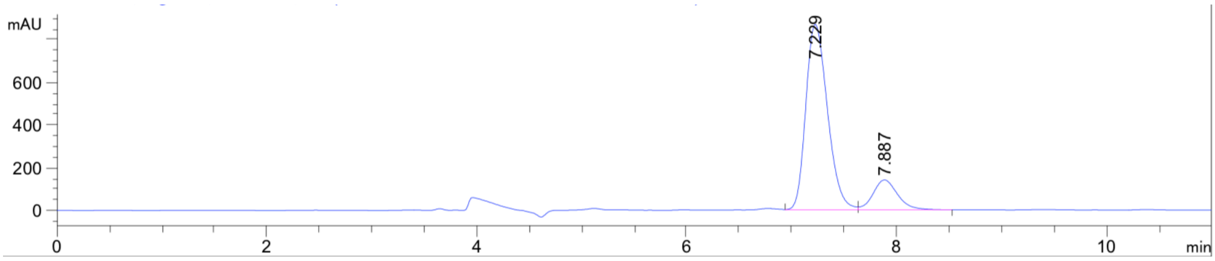

Peak #	RetTime [min]	Type	Width [min]	Area [mAU*s]	Height [mAU]	Area %
1	7.229	VV	0.2236	1.24736e4	868.37579	84.5006
2	7.887	VB	0.2486	2287.95801	140.07599	15.4994

(1*R*,3*R*)-4,4,4-Trifluoro-1-(2-methoxyphenyl)-3-phenylbutan-1-amine – major diastereomer (6i)

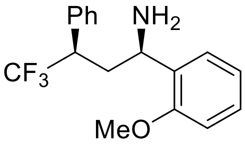

1*R*,3*R*-6i
^{1}H NMR, $CDCl_3$, 400 MHz

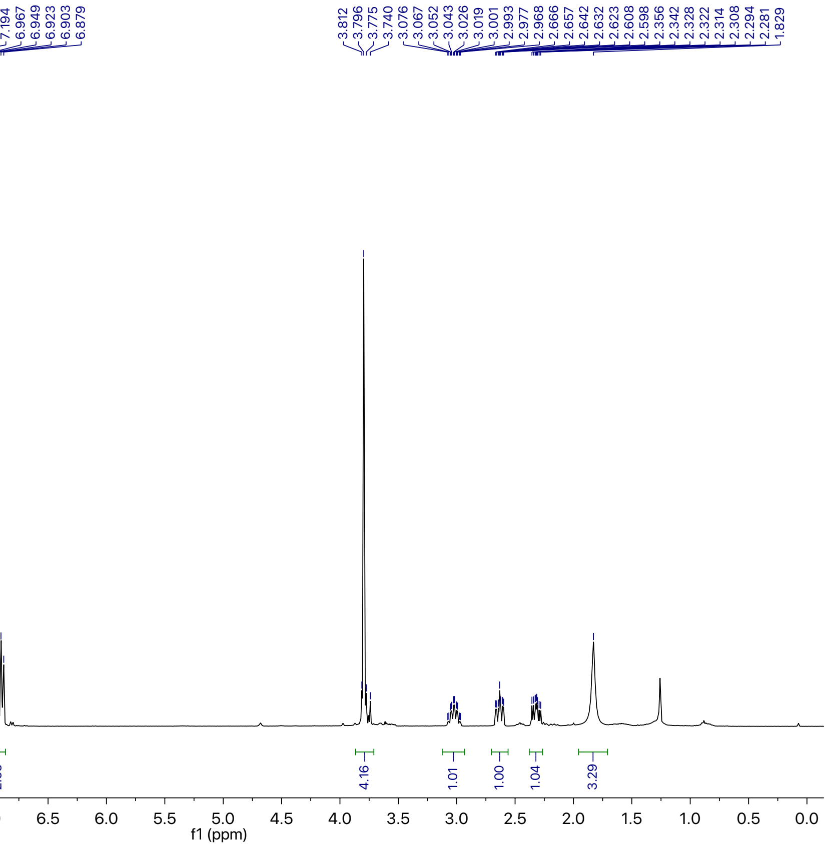

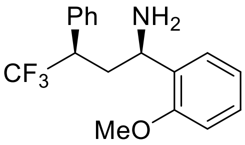

1*R*,3*R*-6i
^{13}C NMR, $CDCl_3$, 100 MHz

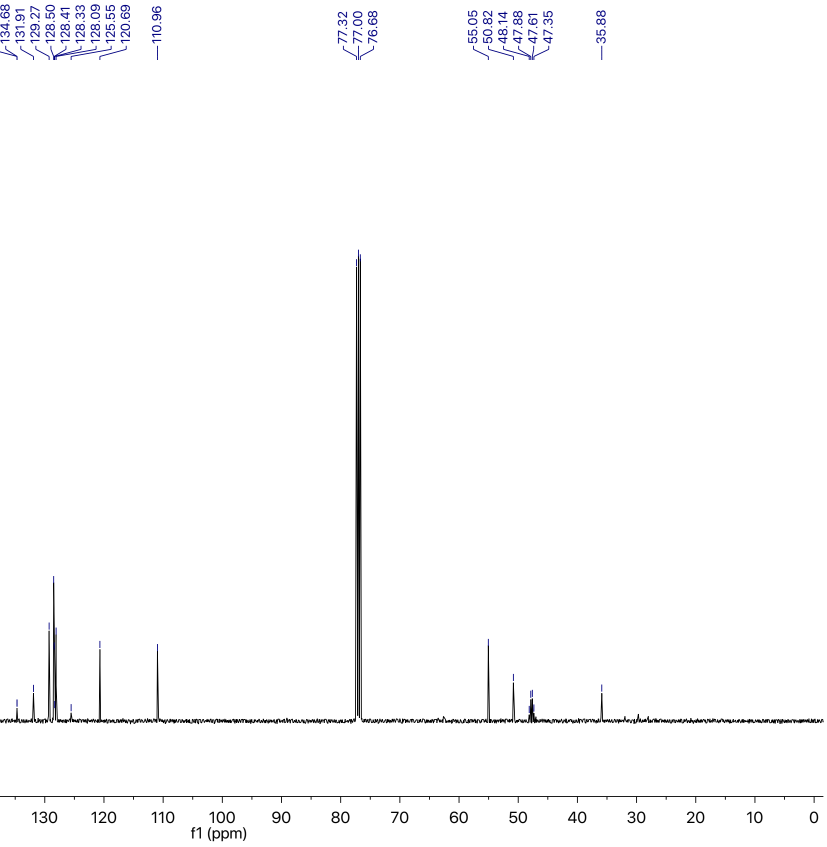

***tert*-Butyl ((1*R*,3*R*)-4,4,4-trifluoro-1-(2-methoxyphenyl)-3-phenylbutyl)carbamate – major diastereomer (6i')**

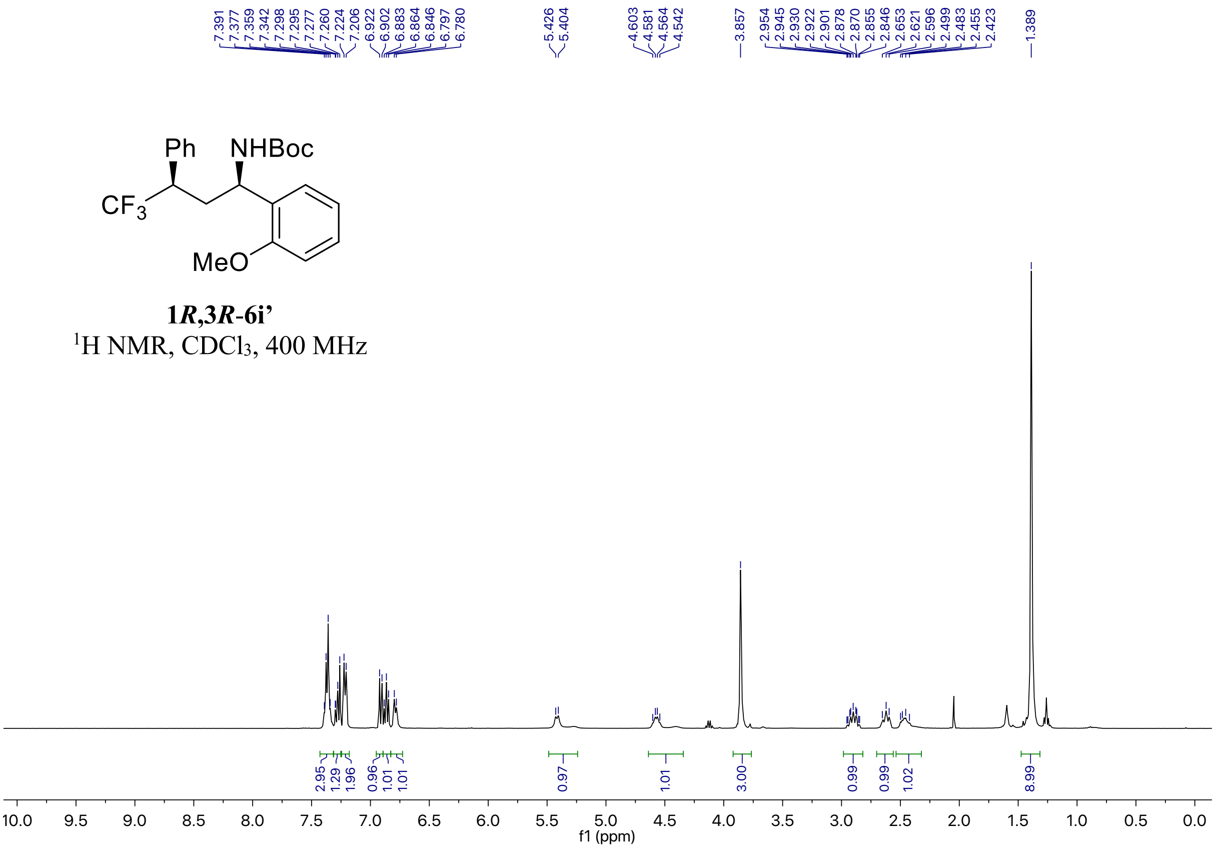

1*R*,3*R*-6i'
^{1}H NMR, $CDCl_3$, 400 MHz

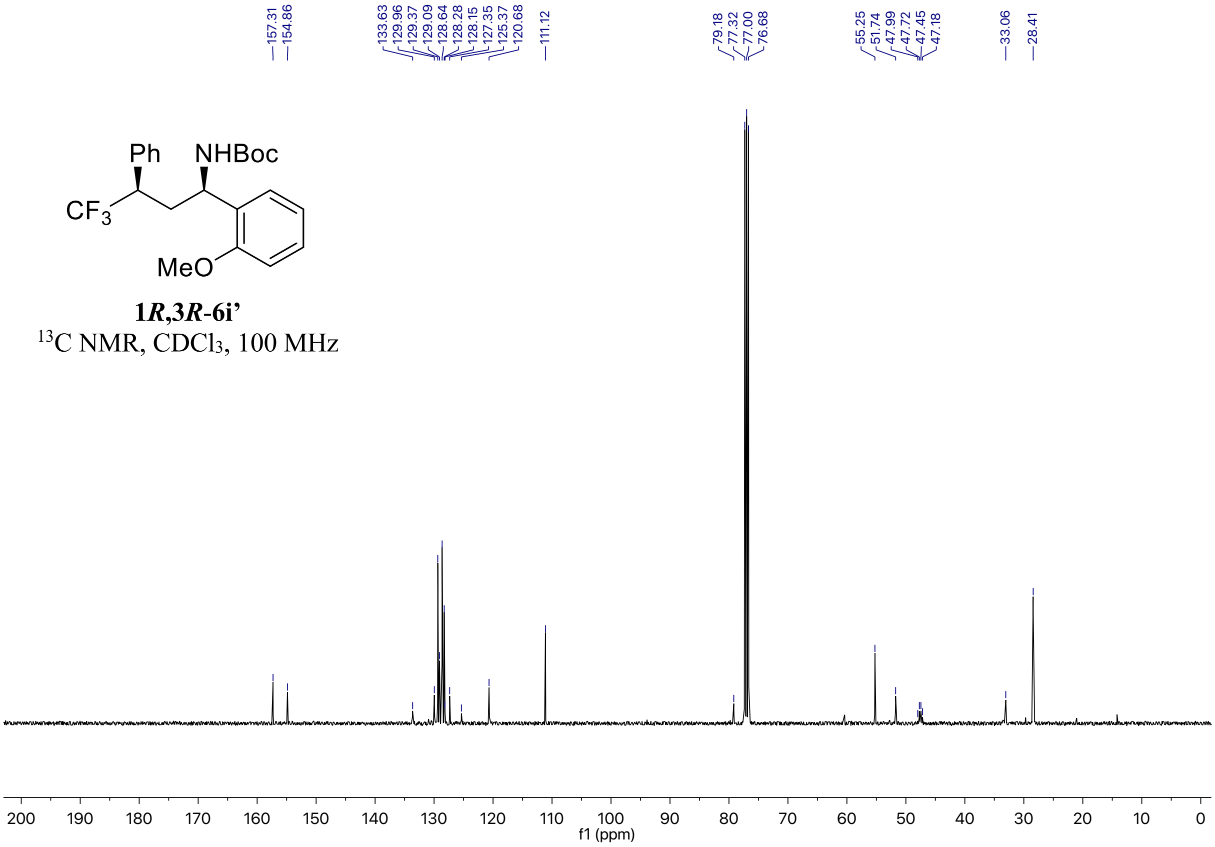

1*R*,3*R*-6i'
^{13}C NMR, $CDCl_3$, 100 MHz

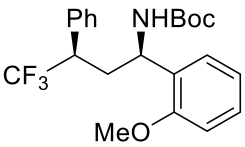

1*R*,3*R*-6i'

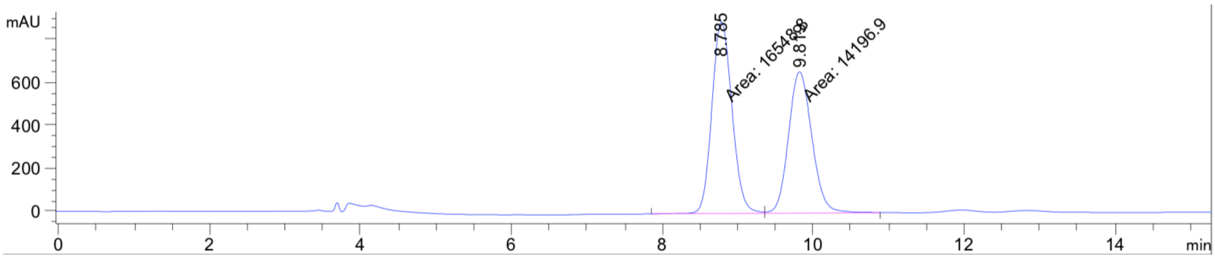

Peak #	RetTime [min]	Type	Width [min]	Area [mAU*s]	Height [mAU]	Area %
1	8.785	MF	0.3095	1.65488e4	891.15698	53.8248
2	9.819	FM	0.3600	1.41969e4	657.23694	46.1752

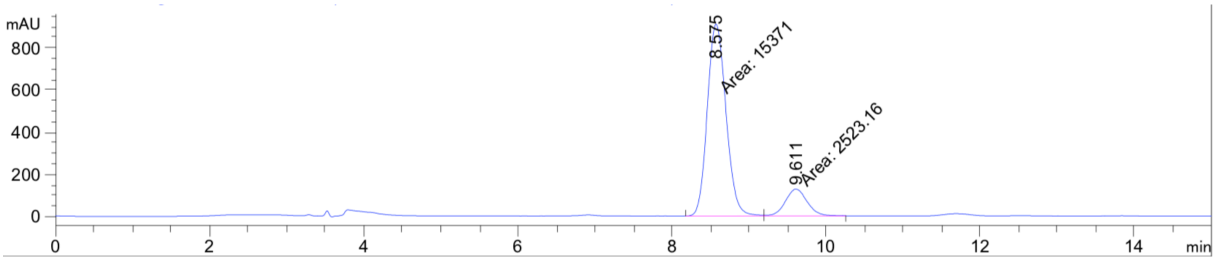

Peak #	RetTime [min]	Type	Width [min]	Area [mAU*s]	Height [mAU]	Area %
1	8.575	MF	0.2810	1.53710e4	911.81726	85.8995
2	9.611	FM	0.3308	2523.16089	127.14045	14.1005

(1*R*,3*R*)-4,4,4-Trifluoro-1-phenyl-3-(p-tolyl)butan-1-amine diastereomer 2 – major diastereomer (6l)

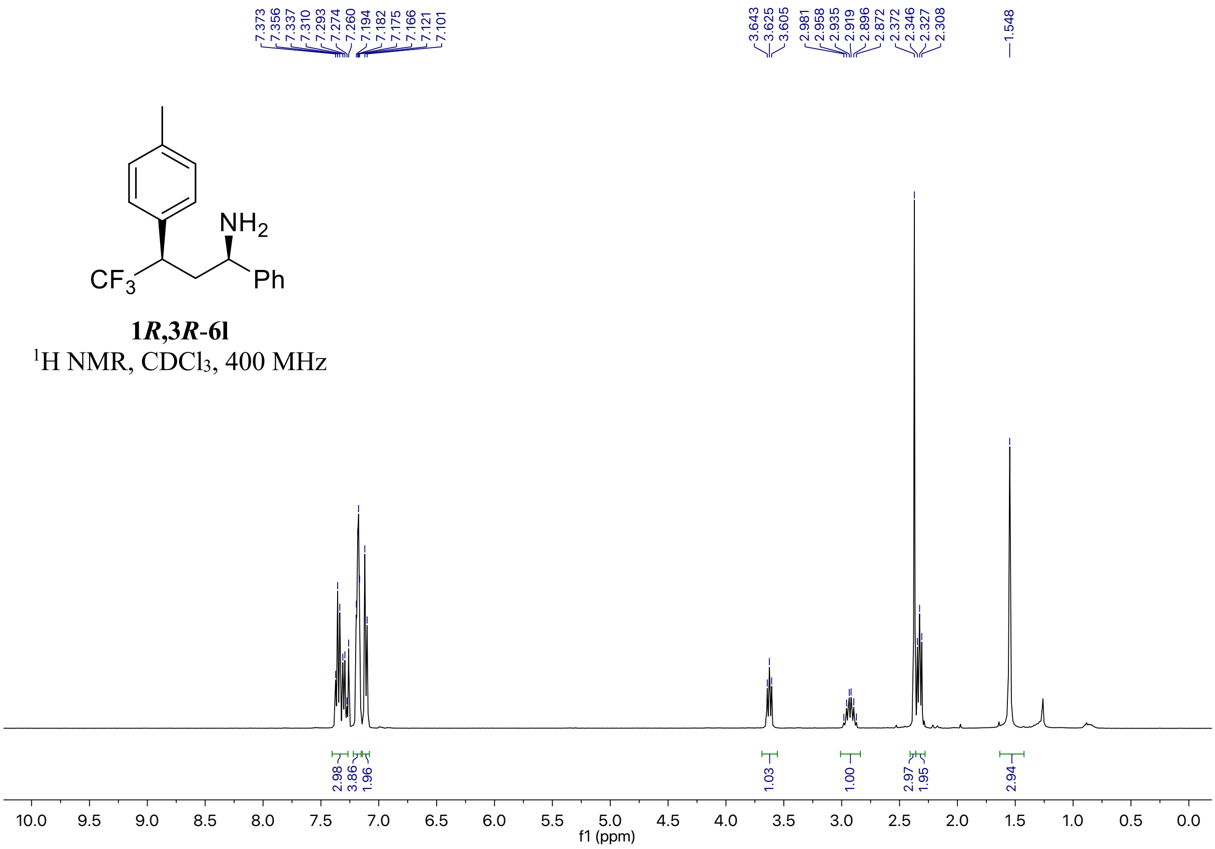

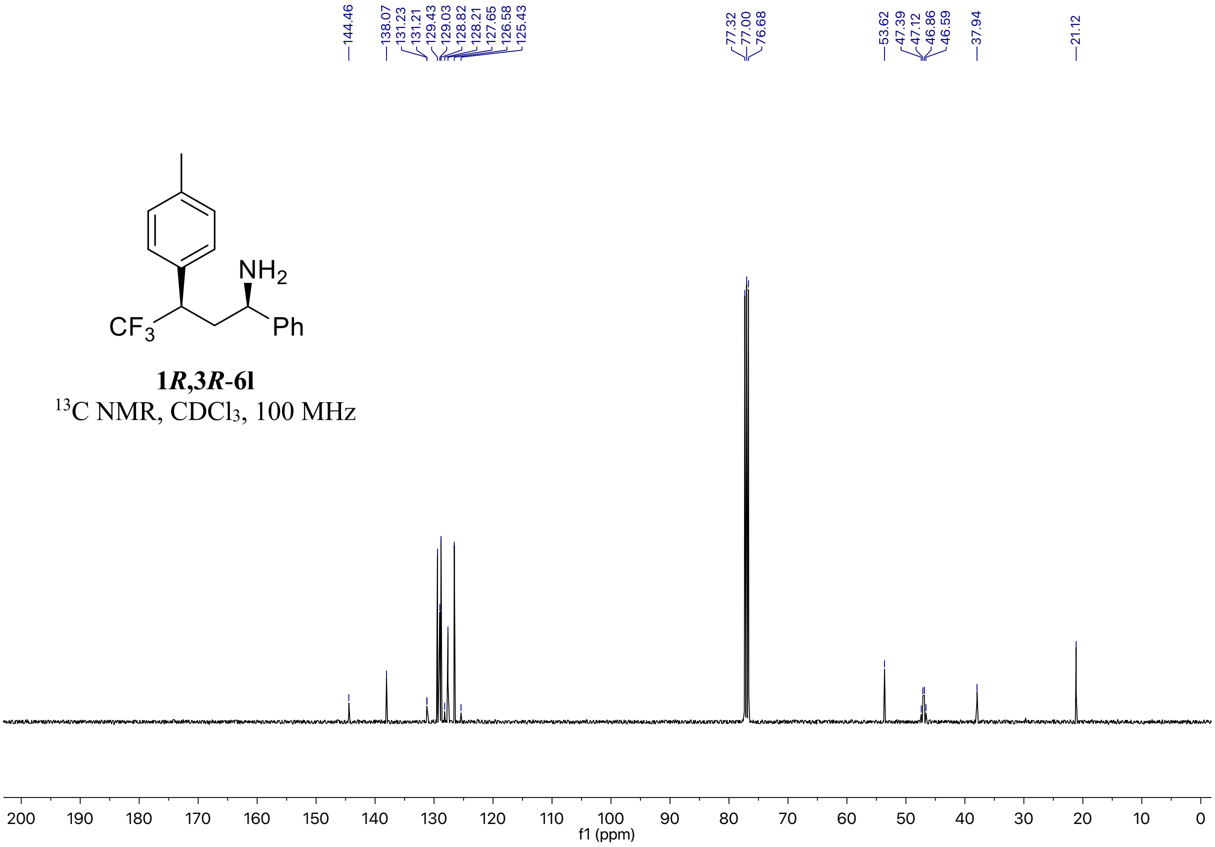

tert-Butyl ((1_R_,3_R_)-4,4,4-trifluoro-1-phenyl-3-(p-tolyl)butyl)carbamate – major diastereomer (6l’)

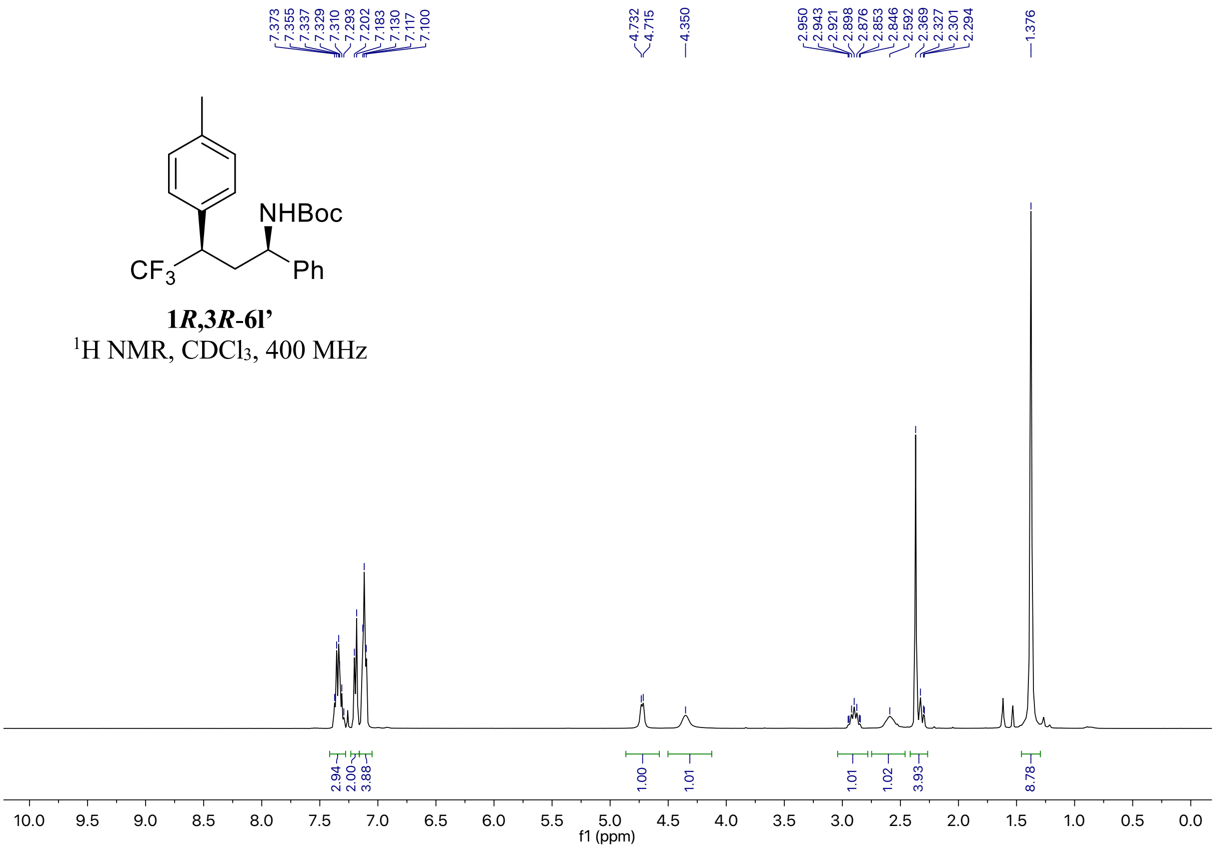

1_R_,3_R_-6l’
^{1}H NMR, $CDCl_3$, 400 MHz

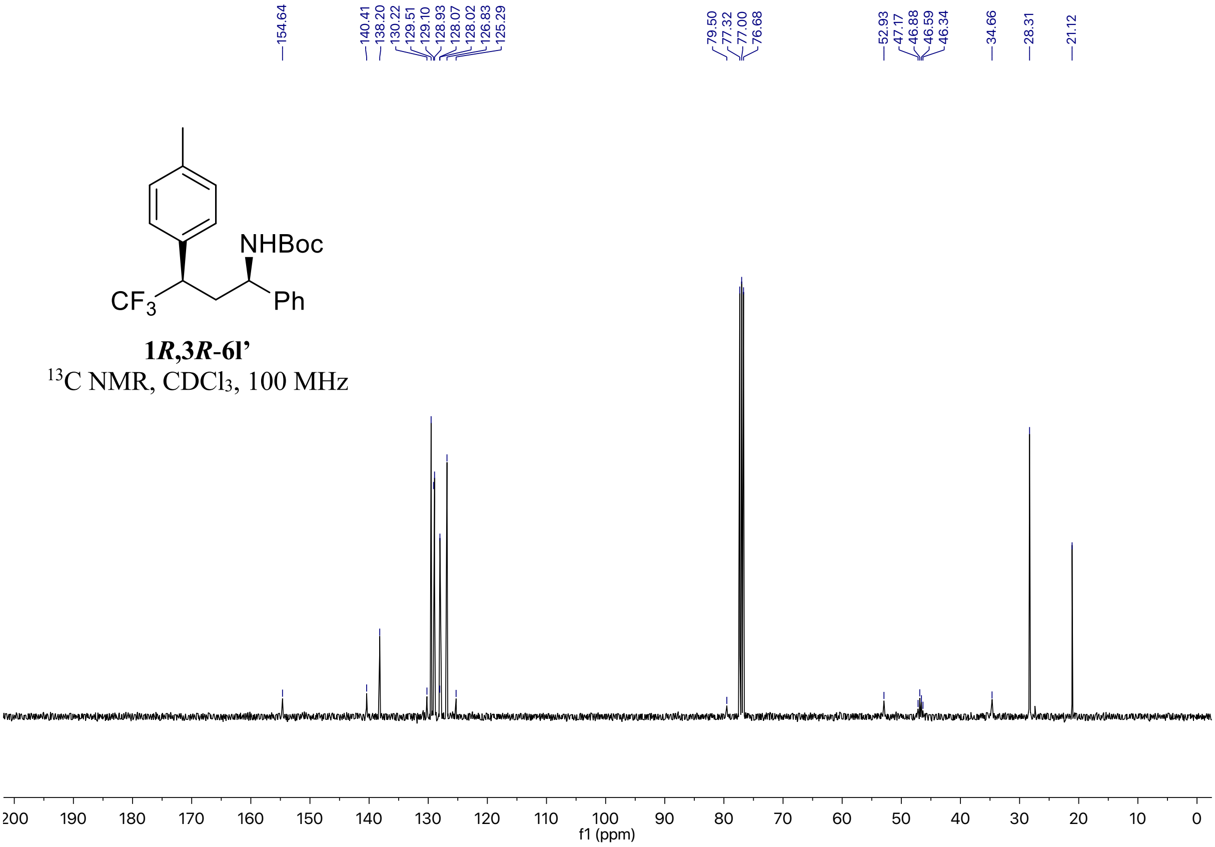

1_R_,3_R_-6l’
^{13}C NMR, $CDCl_3$, 100 MHz

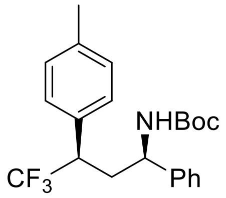

1*R*,3*R*-6l’

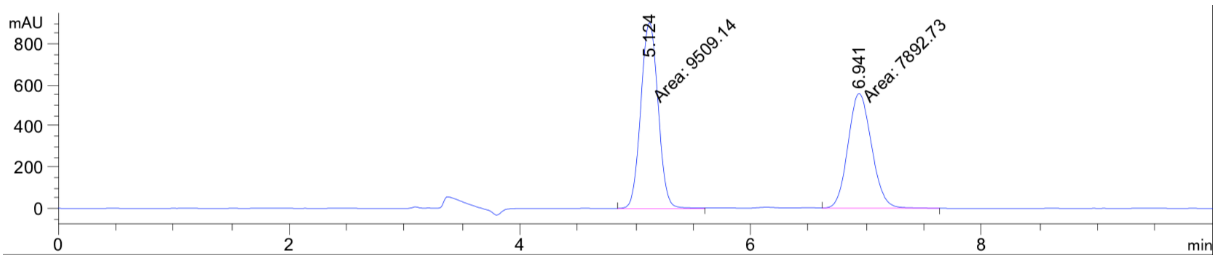

Peak #	RetTime [min]	Type	Width [min]	Area [mAU*s]	Height [mAU]	Area %
1	5.124	MM	0.1753	9509.13867	904.20941	54.6444
2	6.941	MM	0.2358	7892.72803	557.83124	45.3556

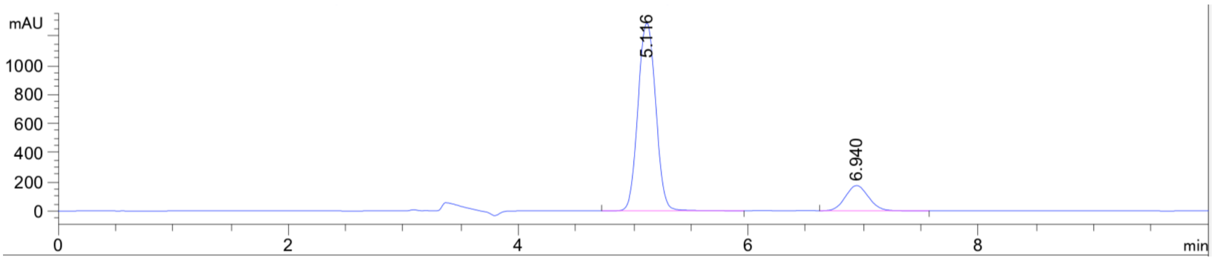

Peak #	RetTime [min]	Type	Width [min]	Area [mAU*s]	Height [mAU]	Area %
1	5.116	VV	0.1744	1.39353e4	1286.57983	85.0469
2	6.940	VB	0.2191	2450.12476	173.17271	14.9531

(1*R*,3*R*)-4,4,4-Trifluoro-3-(4-methoxyphenyl)-1-phenylbutan-1-amine – major diastereomer (6m)

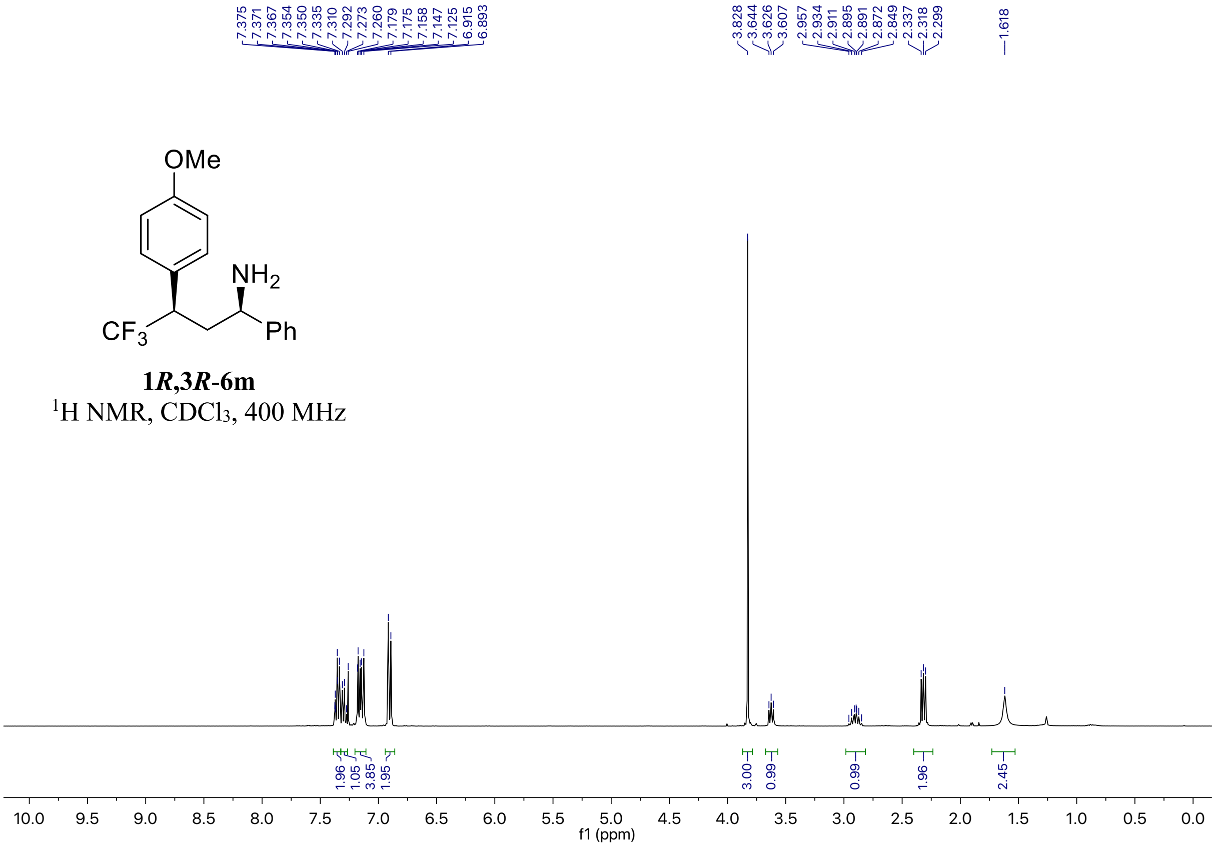

1*R*,3*R*-6m
^{1}H NMR, $CDCl_3$, 400 MHz

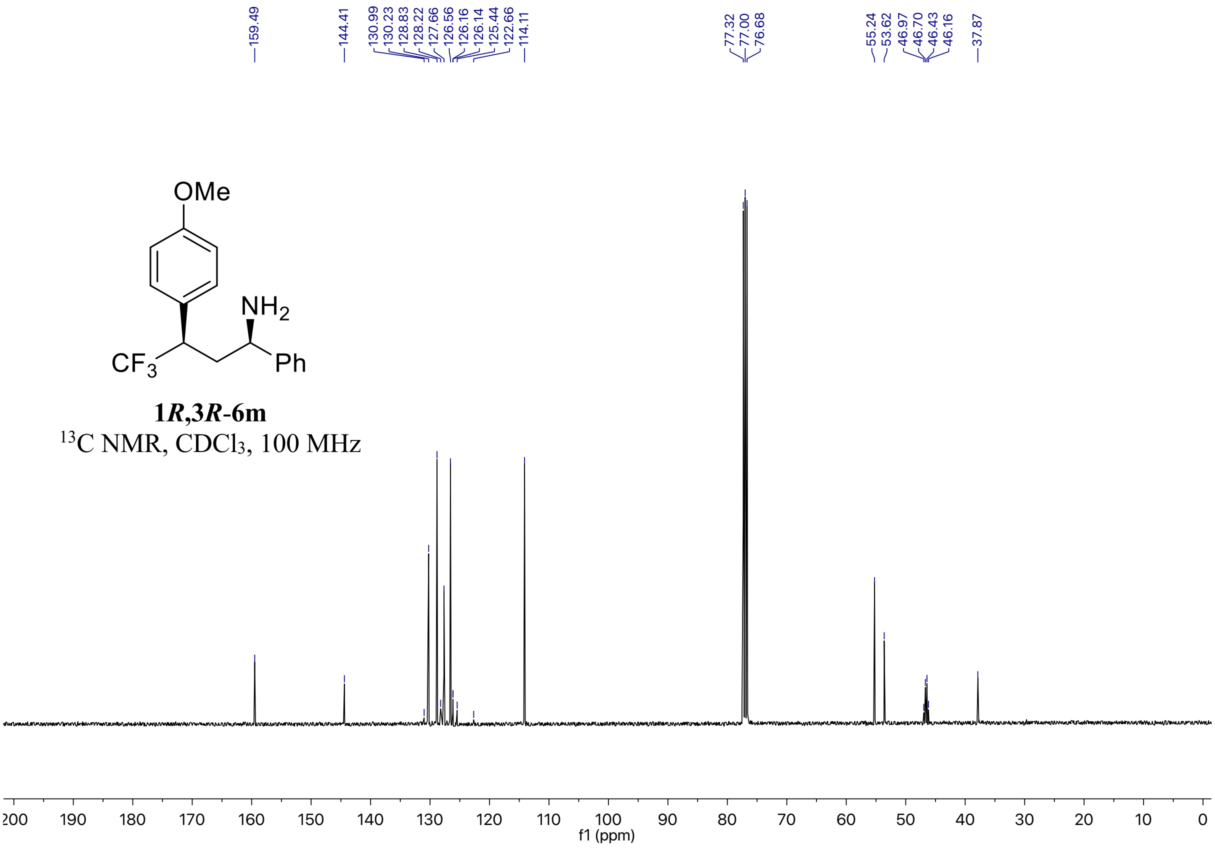

1*R*,3*R*-6m
^{13}C NMR, $CDCl_3$, 100 MHz

tert-Butyl ((1_R_,3_R_)-4,4,4-trifluoro-3-(4-methoxyphenyl)-1-phenylbutyl)carbamate – major diastereomer (6m’)

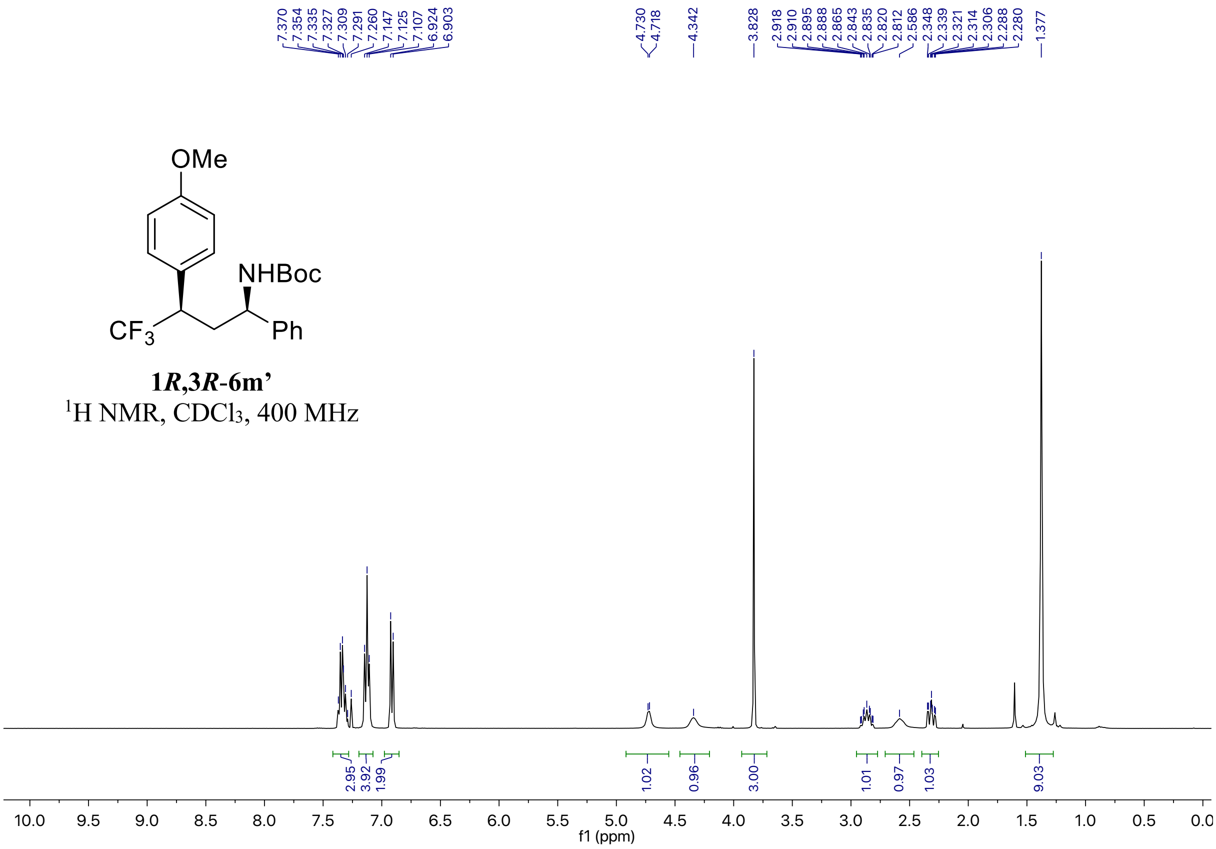

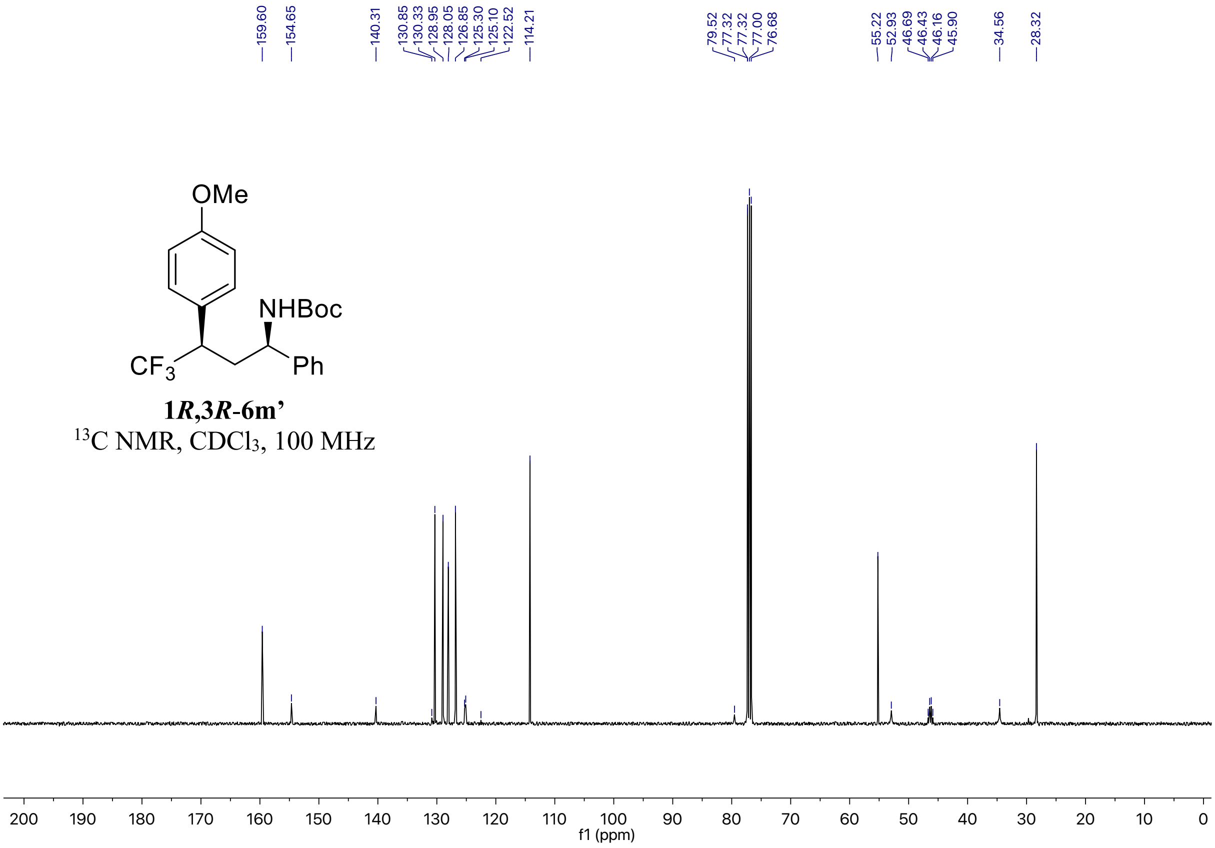

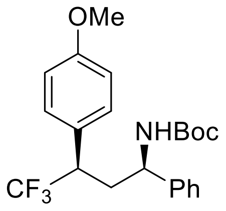

1*R*,3*R*-6m’

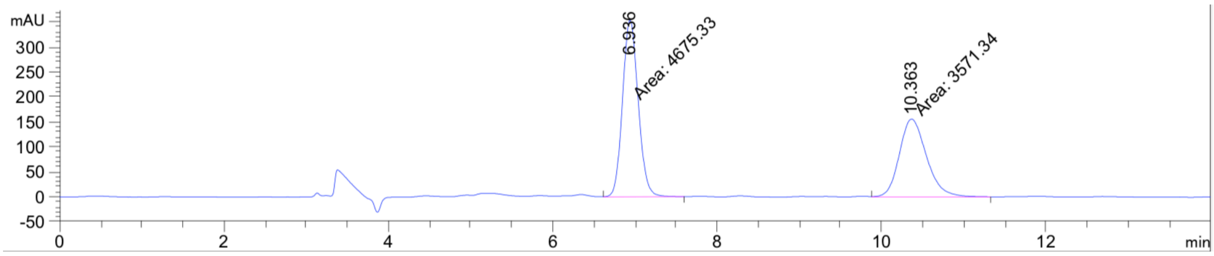

Peak #	RetTime [min]	Type	Width [min]	Area [mAU*s]	Height [mAU]	Area %
1	6.936	MM	0.2203	4675.32715	353.65256	56.6936
2	10.363	MM	0.3816	3571.33643	155.98499	43.3064

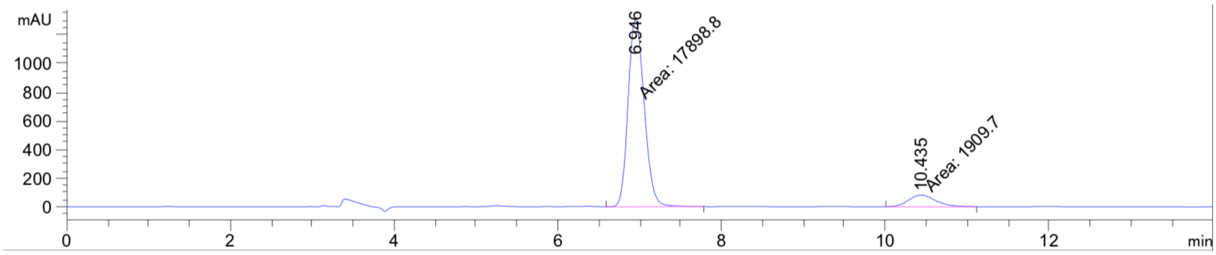

Peak #	RetTime [min]	Type	Width [min]	Area [mAU*s]	Height [mAU]	Area %
1	6.946	MM	0.2288	1.78988e4	1304.03430	90.3592
2	10.435	MM	0.3926	1909.70496	81.07506	9.6408

(1*R*,3*R*)-4,4,4-Trifluoro-1-phenyl-3-(4-(trifluoromethyl)phenyl)butan-1-amine – major diastereomer (6n)

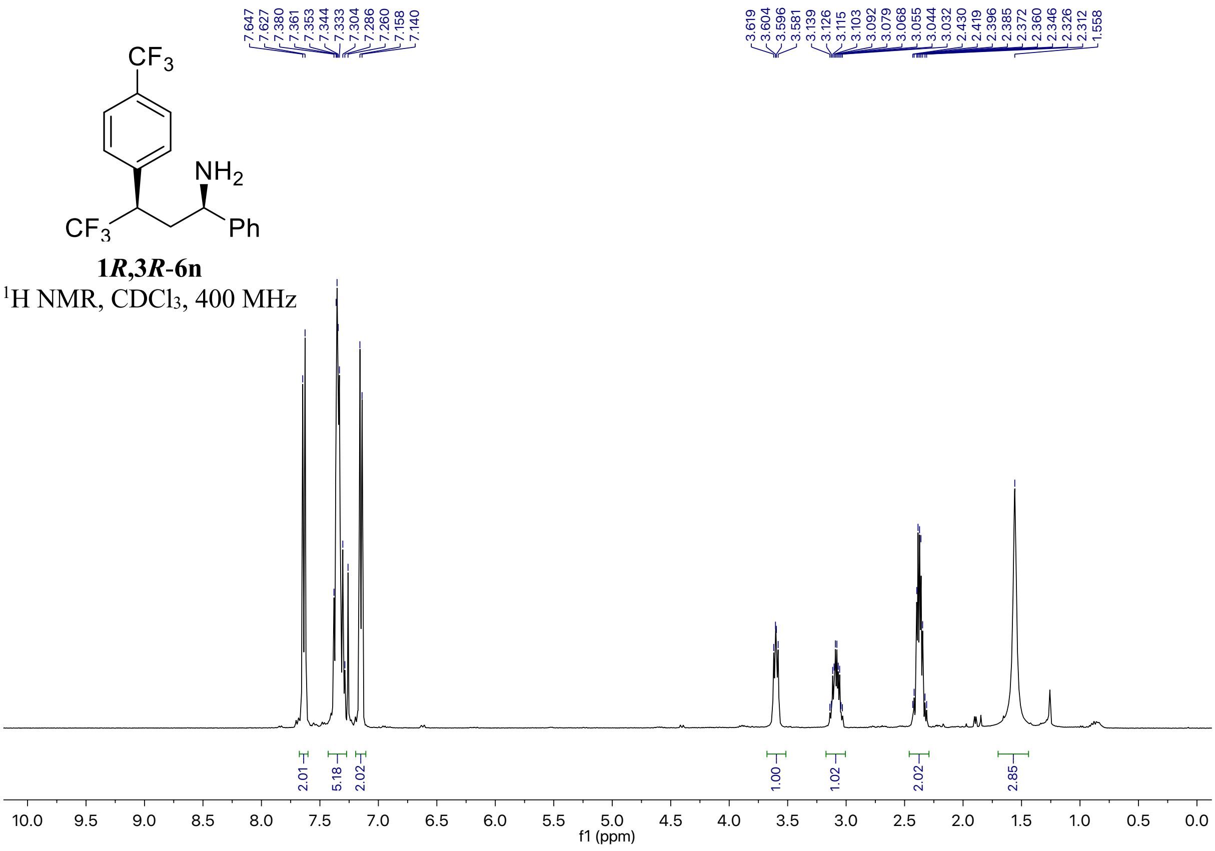

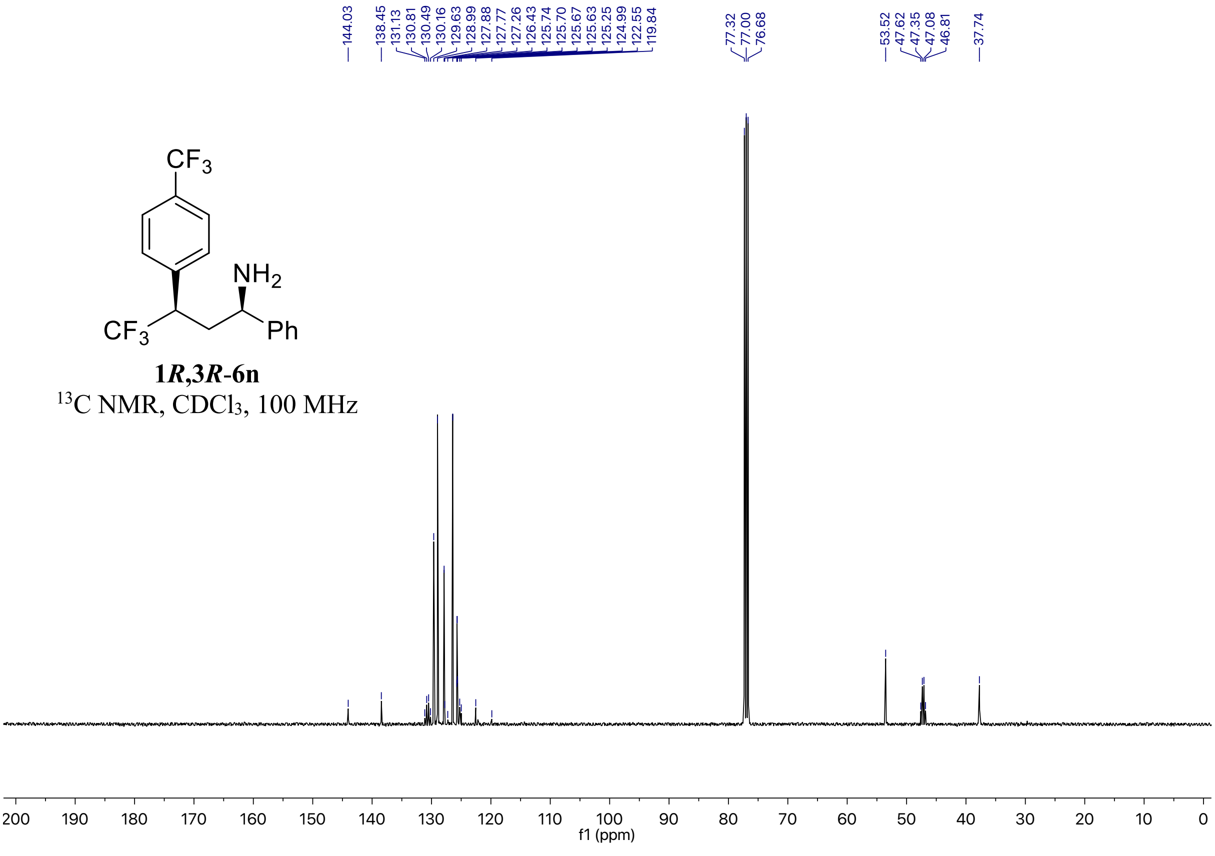

tert-Butyl ((1_R_,3_R_)-4,4,4-trifluoro-1-phenyl-3-(4-(trifluoromethyl)phenyl)butyl)carbamate – major diastereomer (6n')

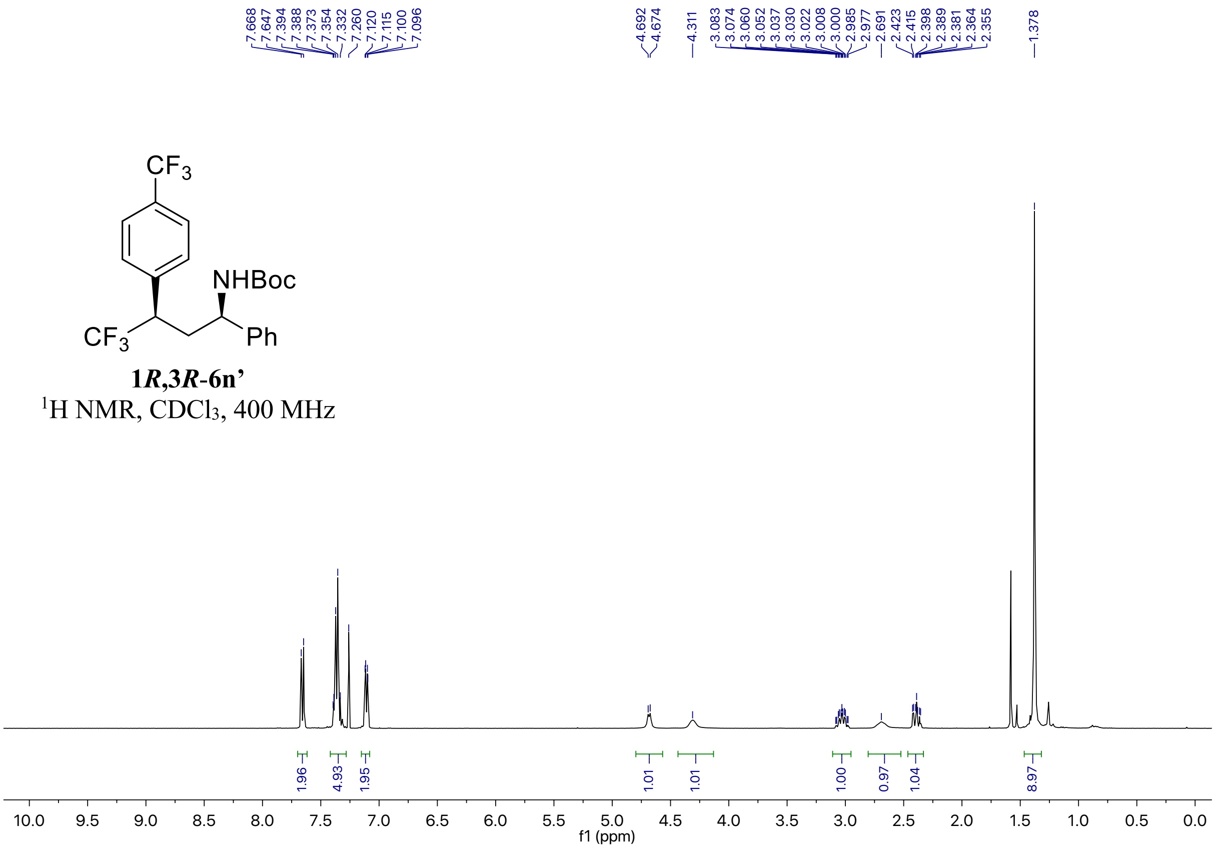

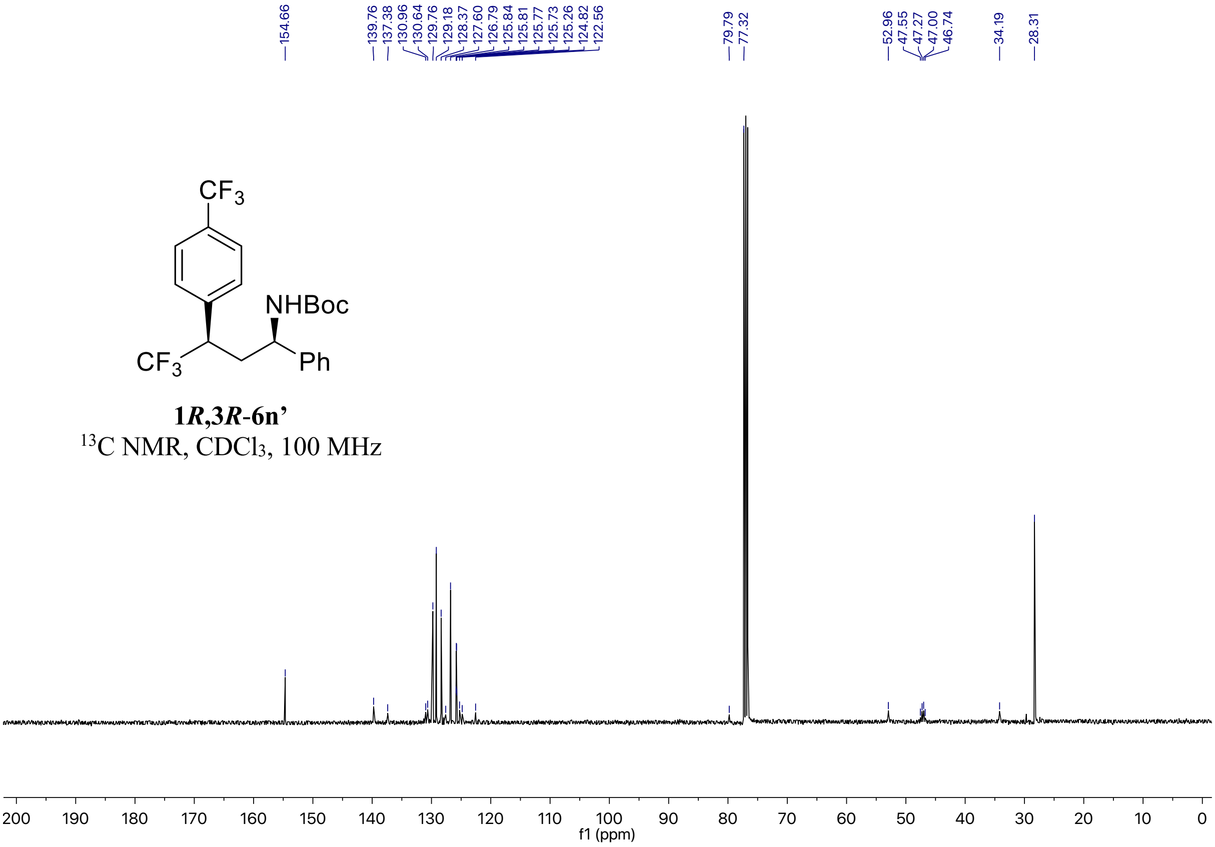

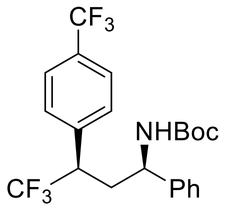

1*R*,3*R*-6n’

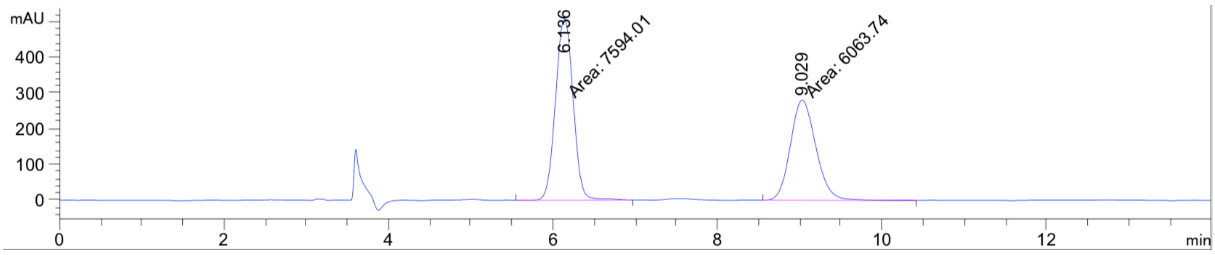

Peak #	RetTime [min]	Type	Width [min]	Area [mAU*s]	Height [mAU]	Area %
1	6.136	MM	0.2488	7594.01318	508.80359	55.6022
2	9.029	MM	0.3608	6063.74023	280.09622	44.3978

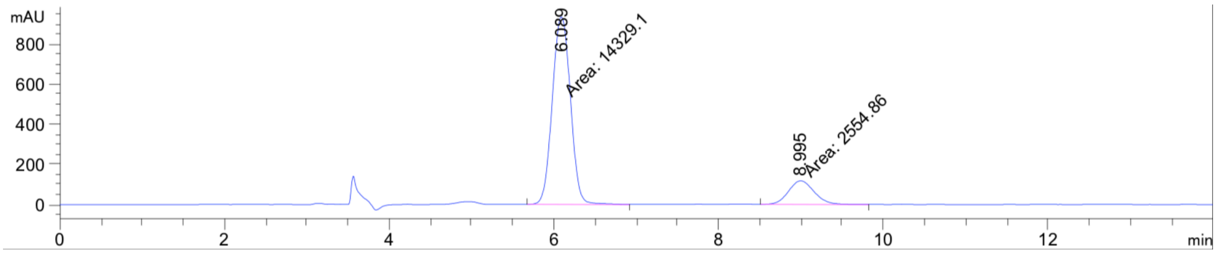

Peak #	RetTime [min]	Type	Width [min]	Area [mAU*s]	Height [mAU]	Area %
1	6.089	MM	0.2549	1.43291e4	936.79315	84.8681
2	8.995	MM	0.3620	2554.85742	117.61539	15.1319

(1*R*,3*R*)-4,4,4-Trifluoro-1-phenyl-3-(*m*-tolyl)butan-1-amine – major diastereomer (6o)

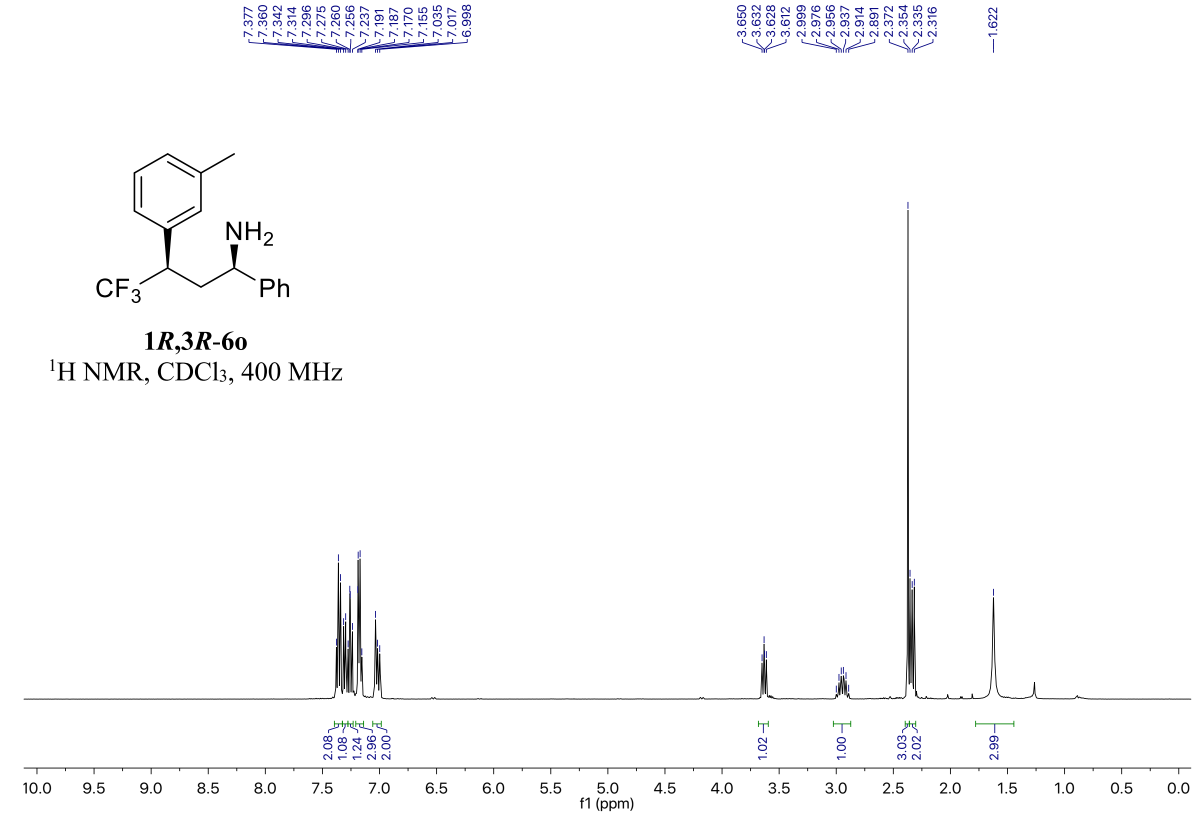

1*R*,3*R*-6o

^{1}H NMR, $CDCl_3$, 400 MHz

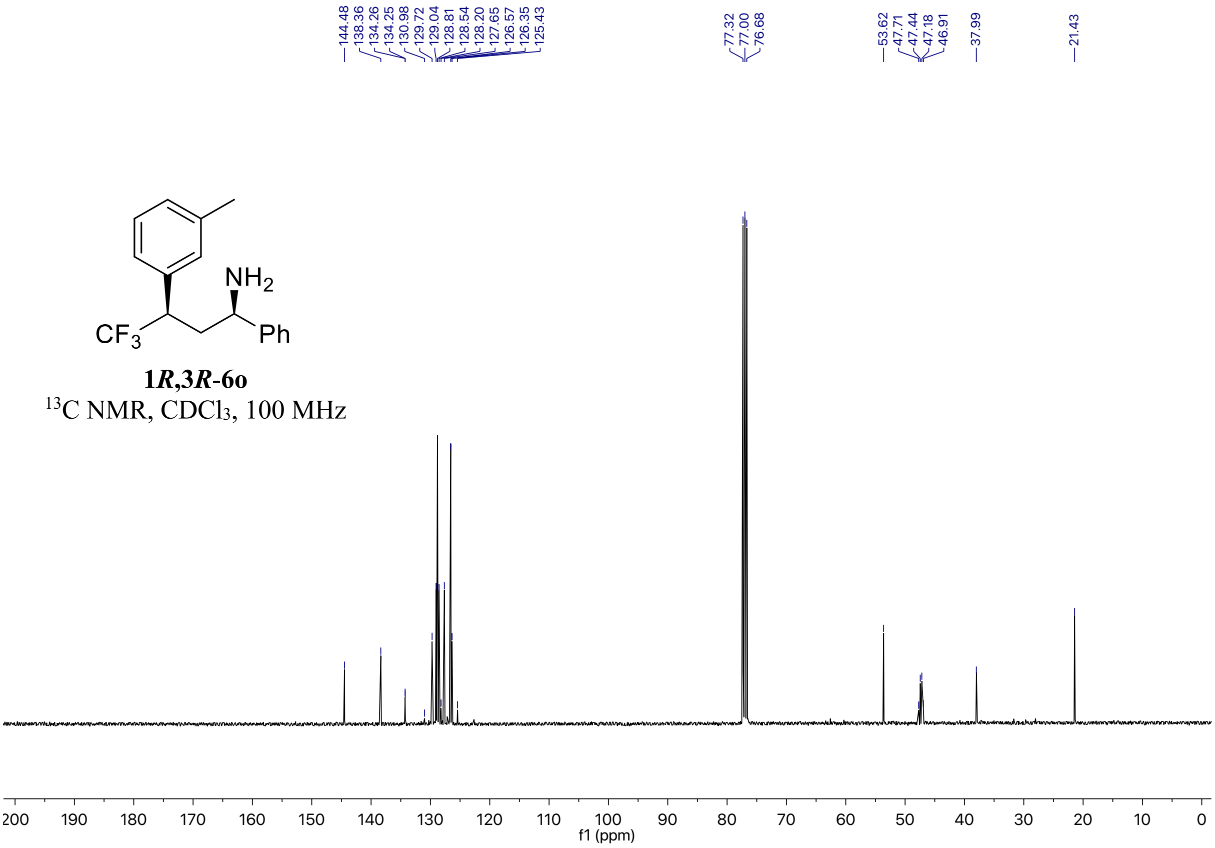

1*R*,3*R*-6o

^{13}C NMR, $CDCl_3$, 100 MHz

***tert*-Butyl ((1*R*,3*R*)-4,4,4-trifluoro-1-phenyl-3-(m-tolyl)butyl)carbamate – major diastereomer (6o')**

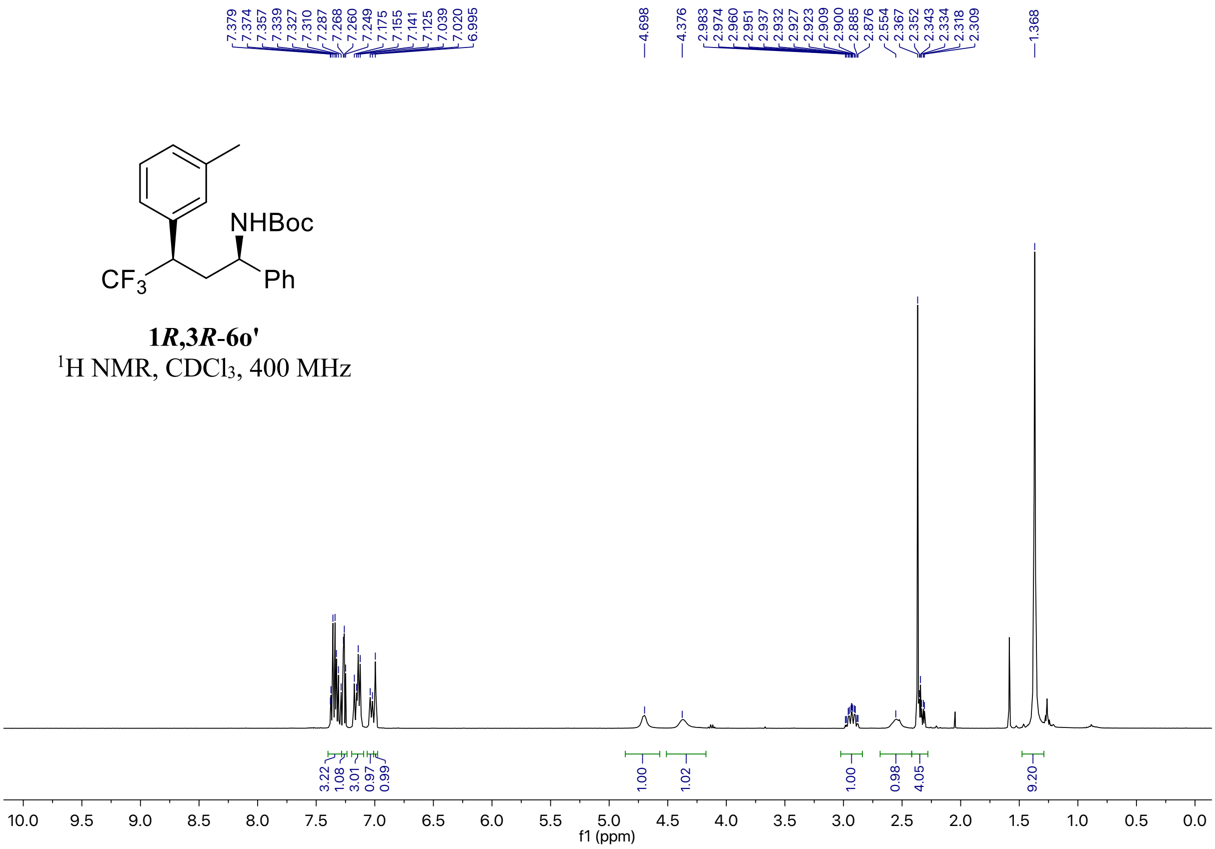

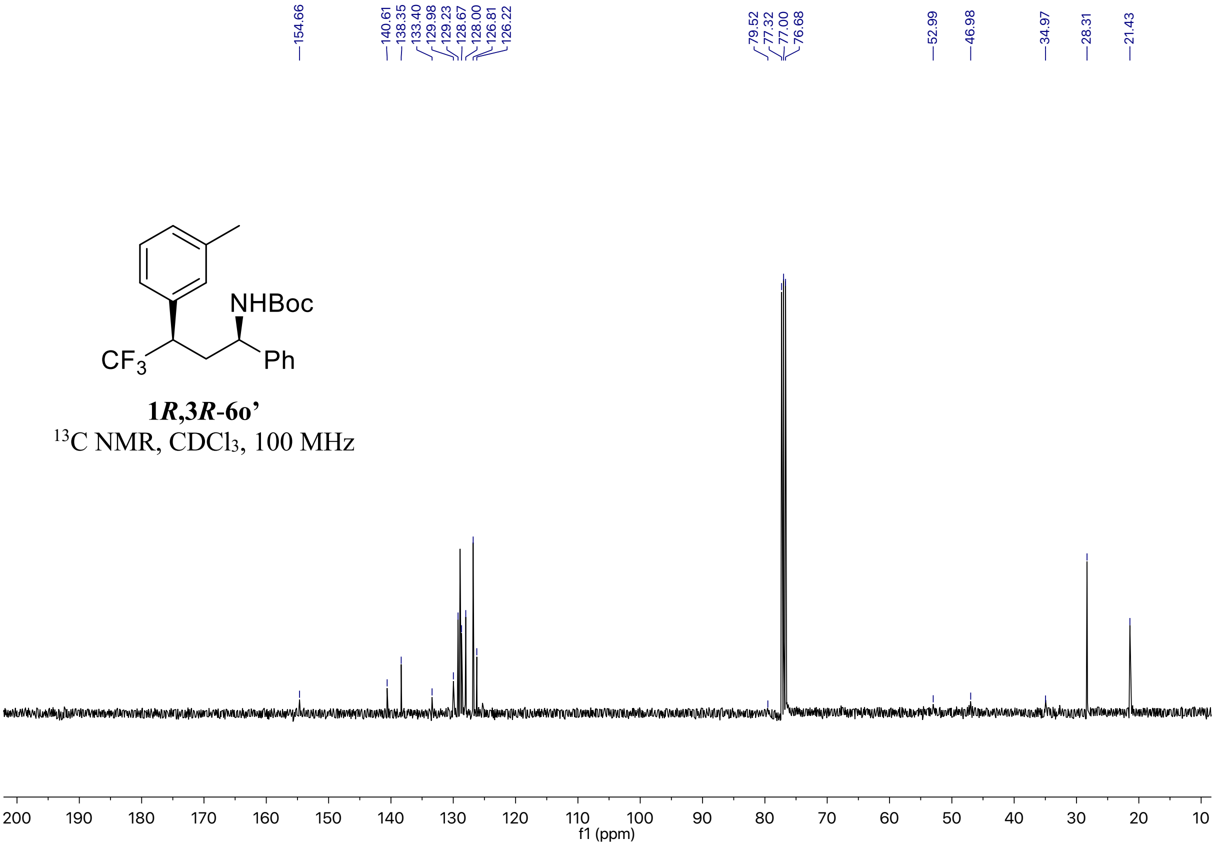

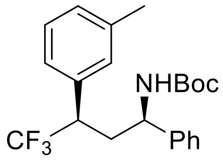

1*R*,3*R*-6o'

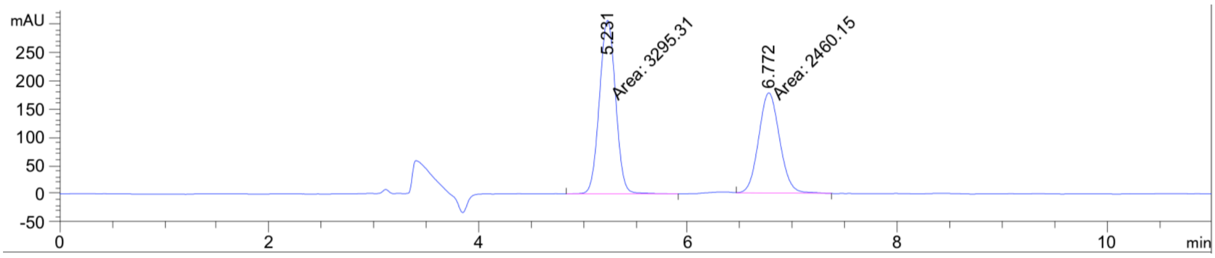

Peak #	RetTime [min]	Type	Width [min]	Area [mAU*s]	Height [mAU]	Area %
1	5.231	MM	0.1792	3295.30908	306.51895	57.2553
2	6.772	MM	0.2319	2460.15430	176.84816	42.7447

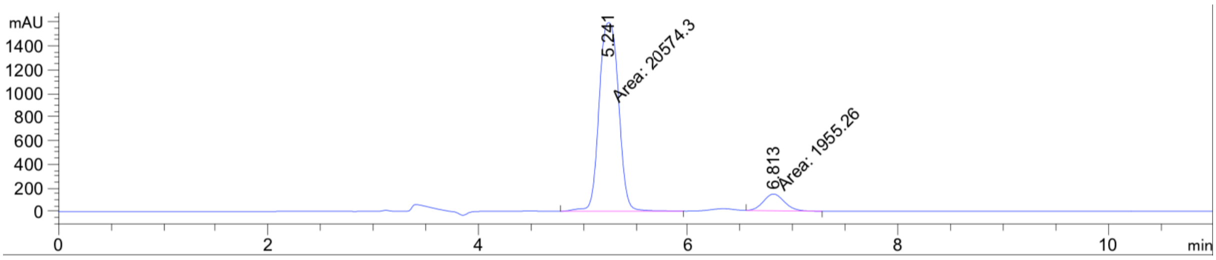

Peak #	RetTime [min]	Type	Width [min]	Area [mAU*s]	Height [mAU]	Area %
1	5.241	MM	0.2149	2.05743e4	1595.82104	91.3214
2	6.813	MM	0.2309	1955.25659	141.10260	8.6786

(1*S*,3*S*)-4,4,4-Trifluoro-1-phenyl-3-(thiophen-2-yl)butan-1-amine – minor diastereomer (6p)

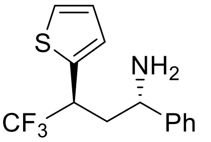

1*S*,3*S*-6p

^{1}H NMR, CDCl$_3$, 400 MHz

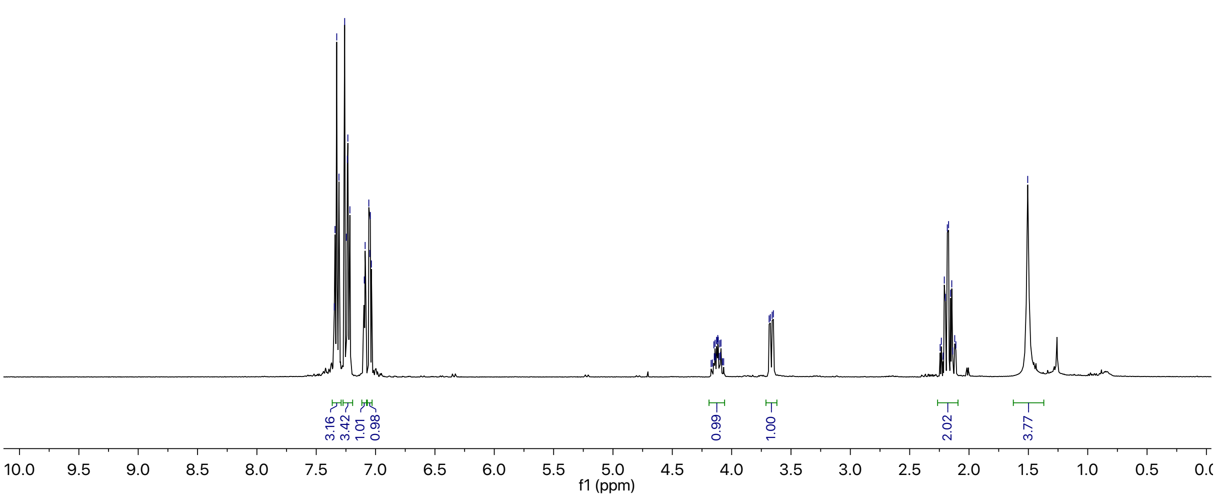

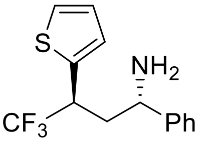

1*S*,3*S*-6p

^{13}C NMR, CDCl$_3$, 100 MHz

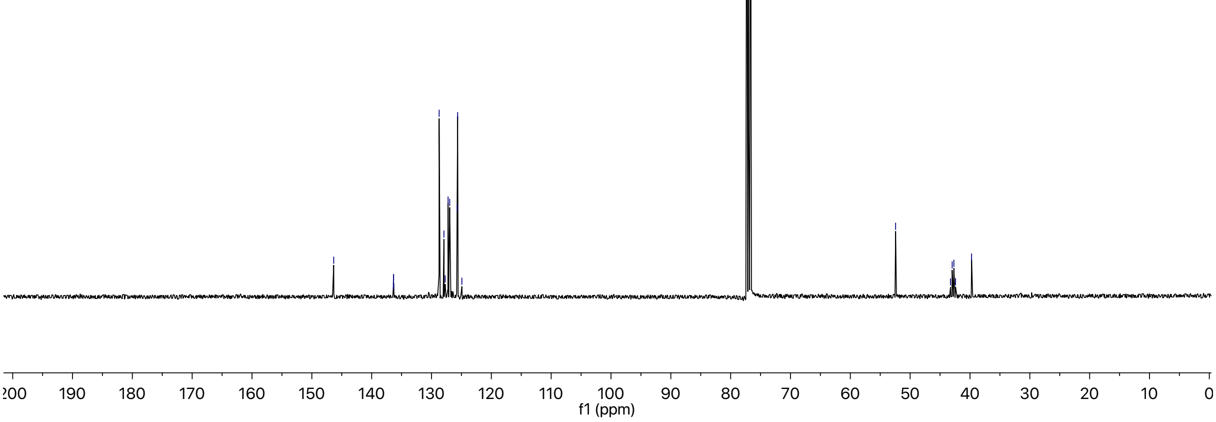

tert-Butyl ((1_S_,3_S_)-4,4,4-trifluoro-1-phenyl-3-(m-tolyl)butyl)carbamate – minor diastereomer (6p')

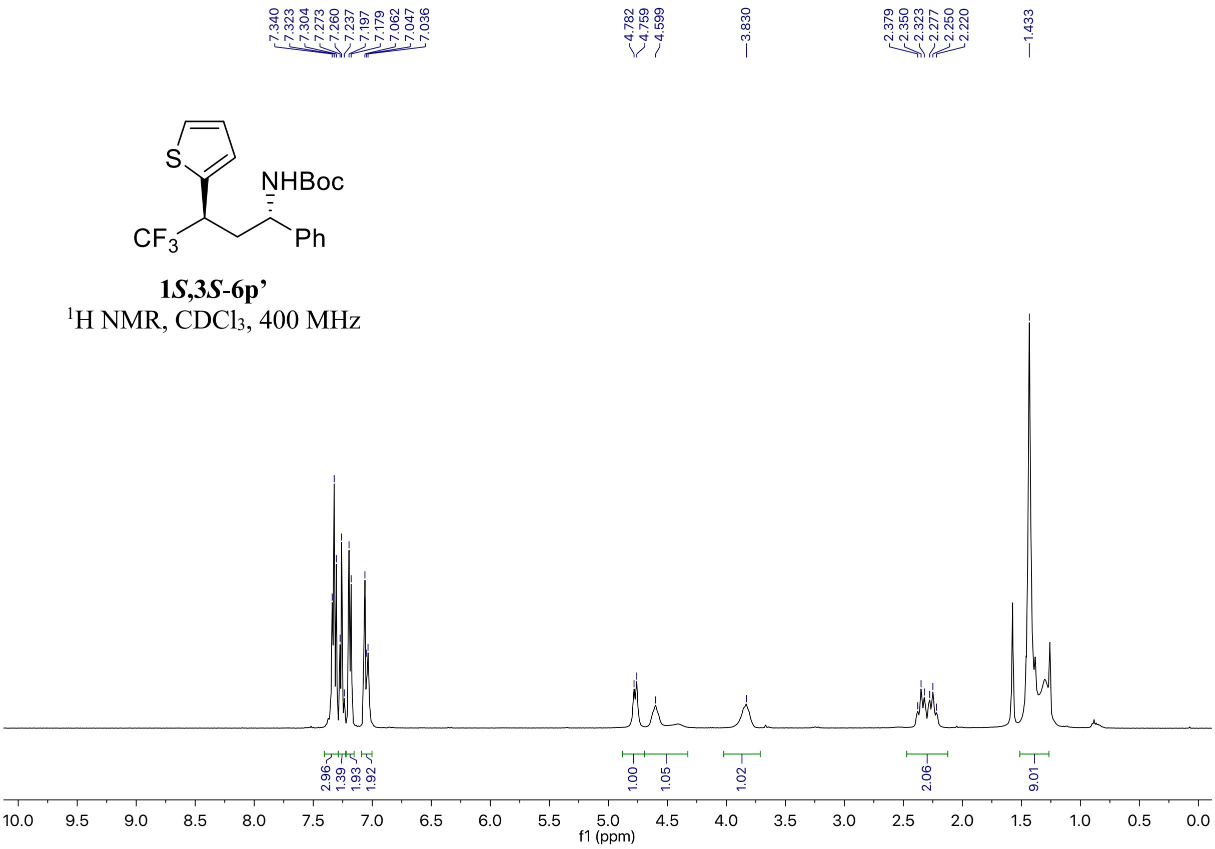

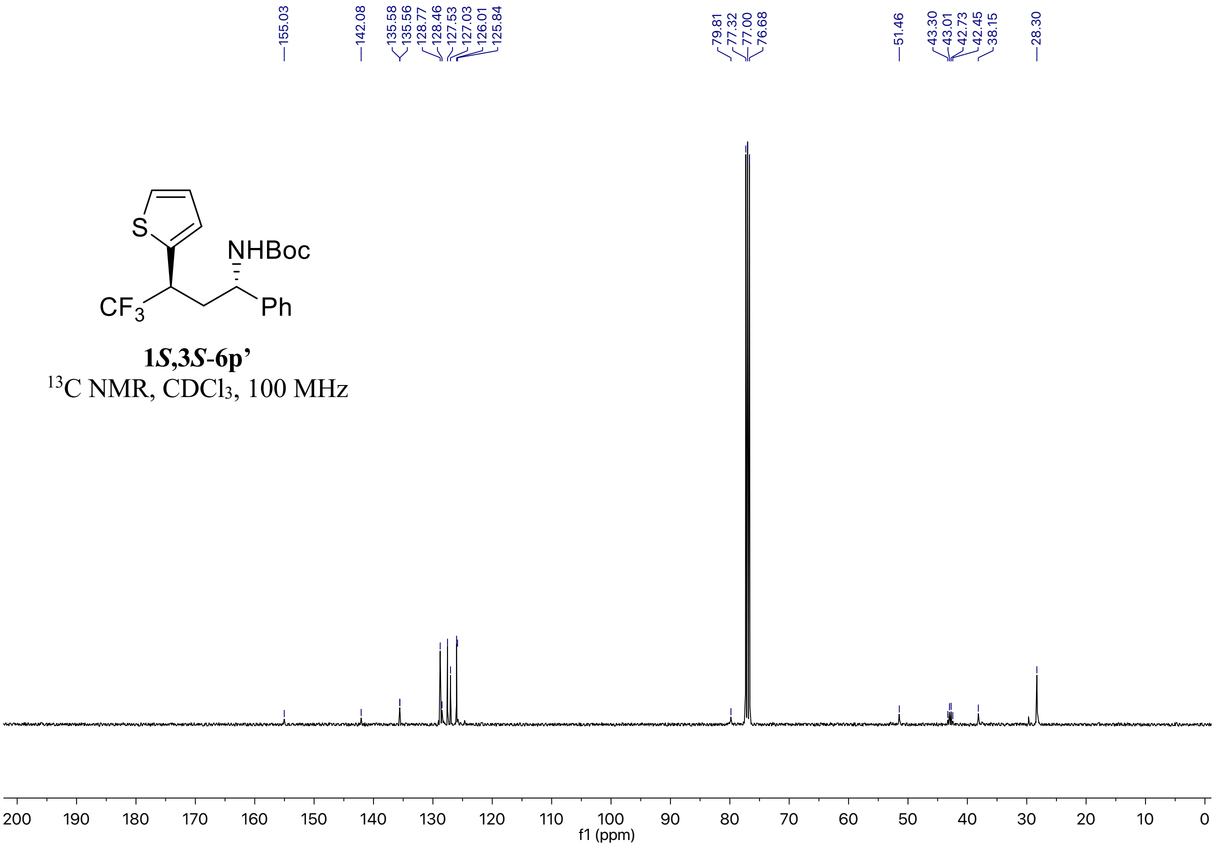

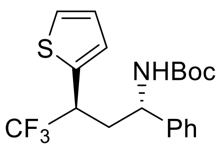

1*S*,3*S*-6p'

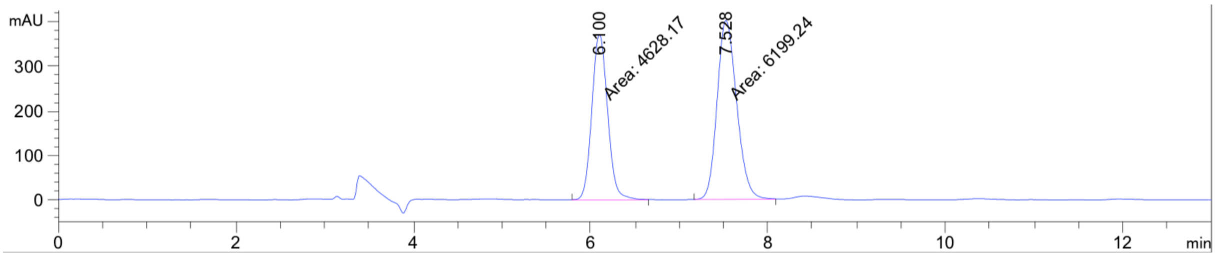

Peak #	RetTime [min]	Type	Width [min]	Area [mAU*s]	Height [mAU]	Area %
1	6.100	MM	0.2064	4628.16504	373.68042	42.7449
2	7.528	MM	0.2559	6199.23682	403.77646	57.2551

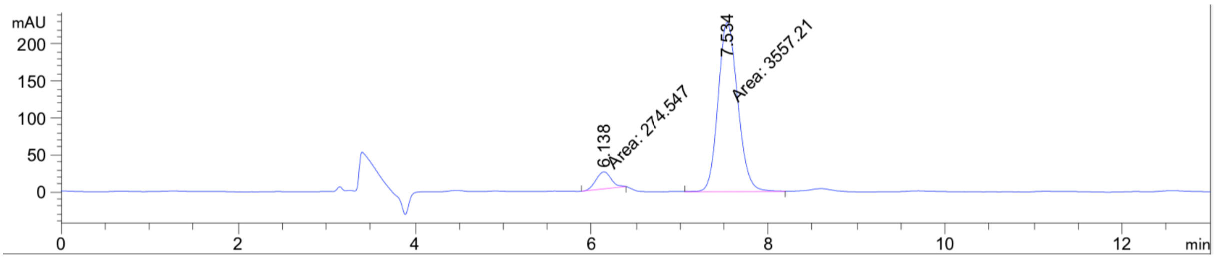

Peak #	RetTime [min]	Type	Width [min]	Area [mAU*s]	Height [mAU]	Area %
1	6.138	MM	0.1984	274.54715	23.06816	7.1650
2	7.534	MM	0.2583	3557.21191	229.51276	92.8350

(1*R*,3*S*)-4,4,4-Trifluoro-1-phenyl-3-(thiophen-2-yl)butan-1-amine – major diastereomer (6p)

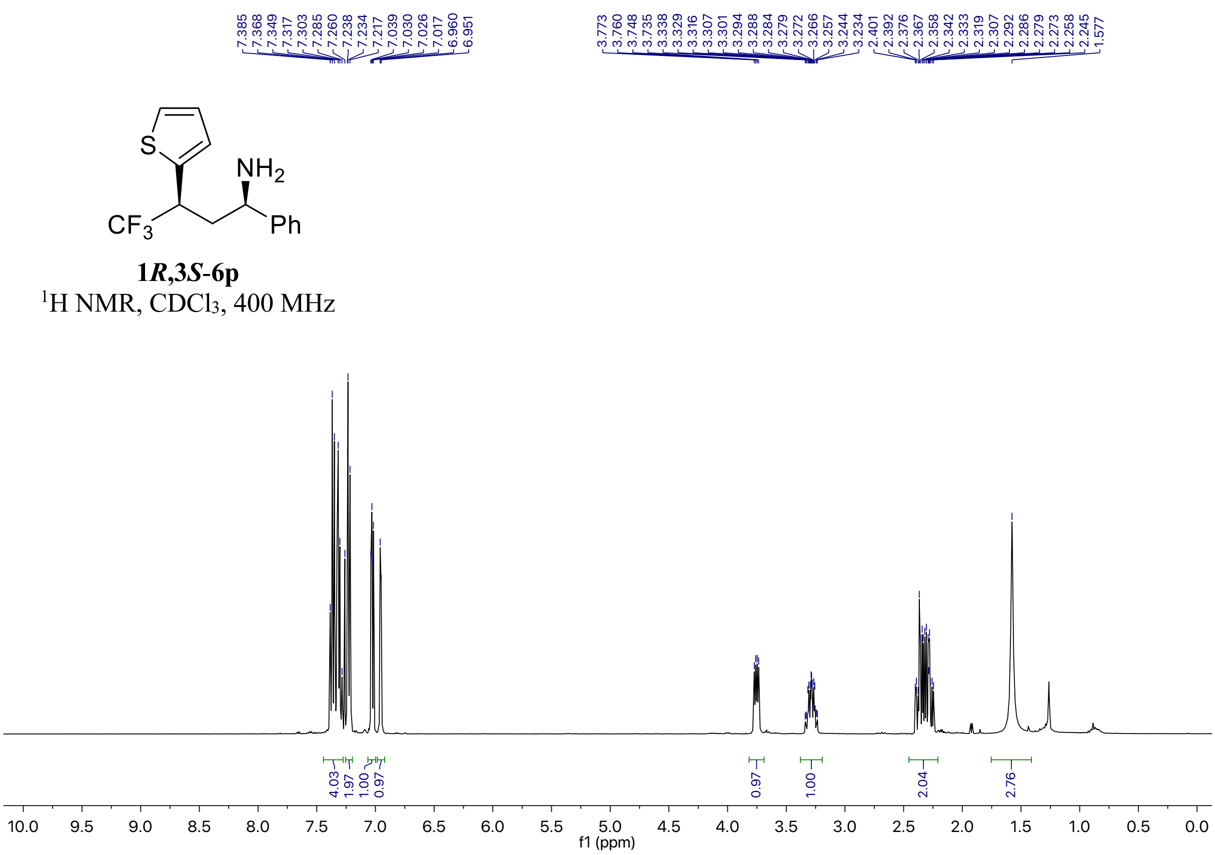

1*R*,3*S*-6p
^{1}H NMR, $CDCl_3$, 400 MHz

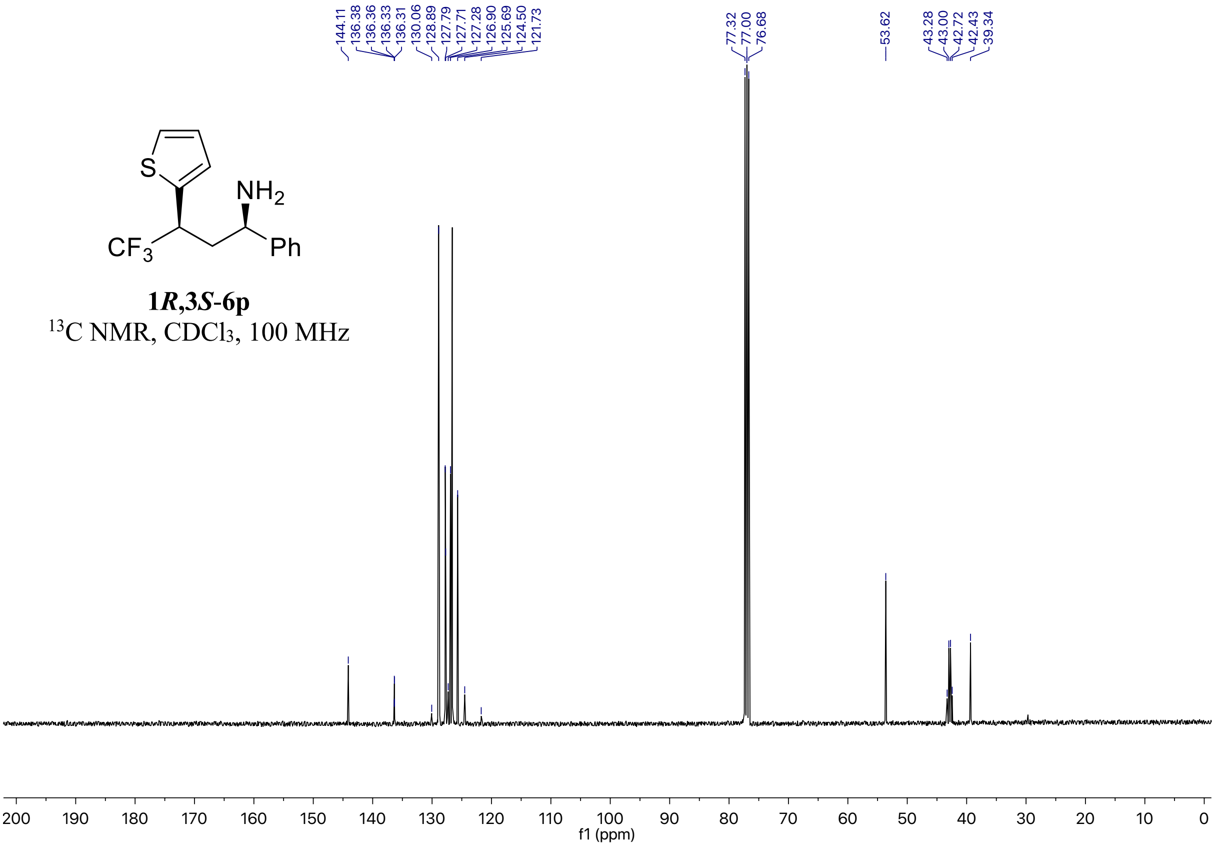

1*R*,3*S*-6p
^{13}C NMR, $CDCl_3$, 100 MHz

tert-Butyl ((1_R_,3_S_)-4,4,4-trifluoro-1-phenyl-3-(thiophen-2-yl)butyl)carbamate – major diastereomer (6p’)

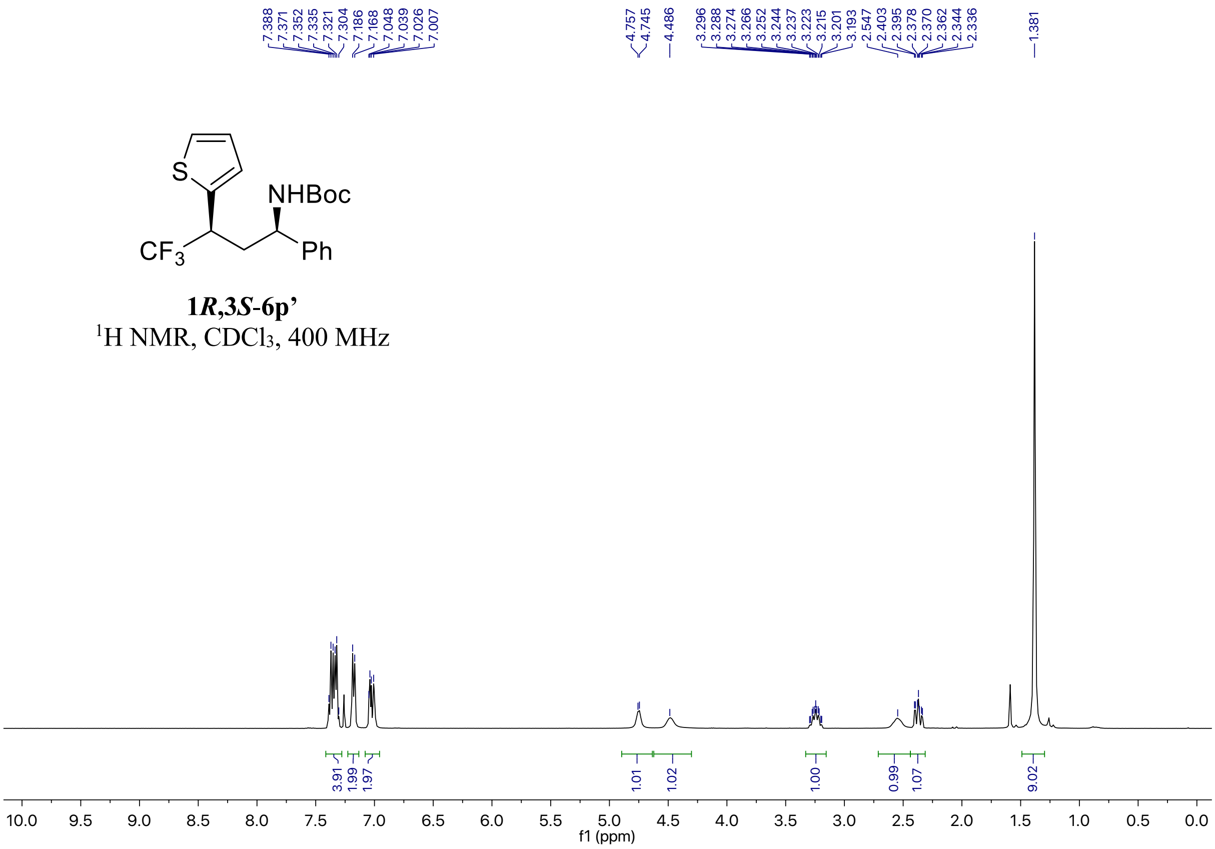

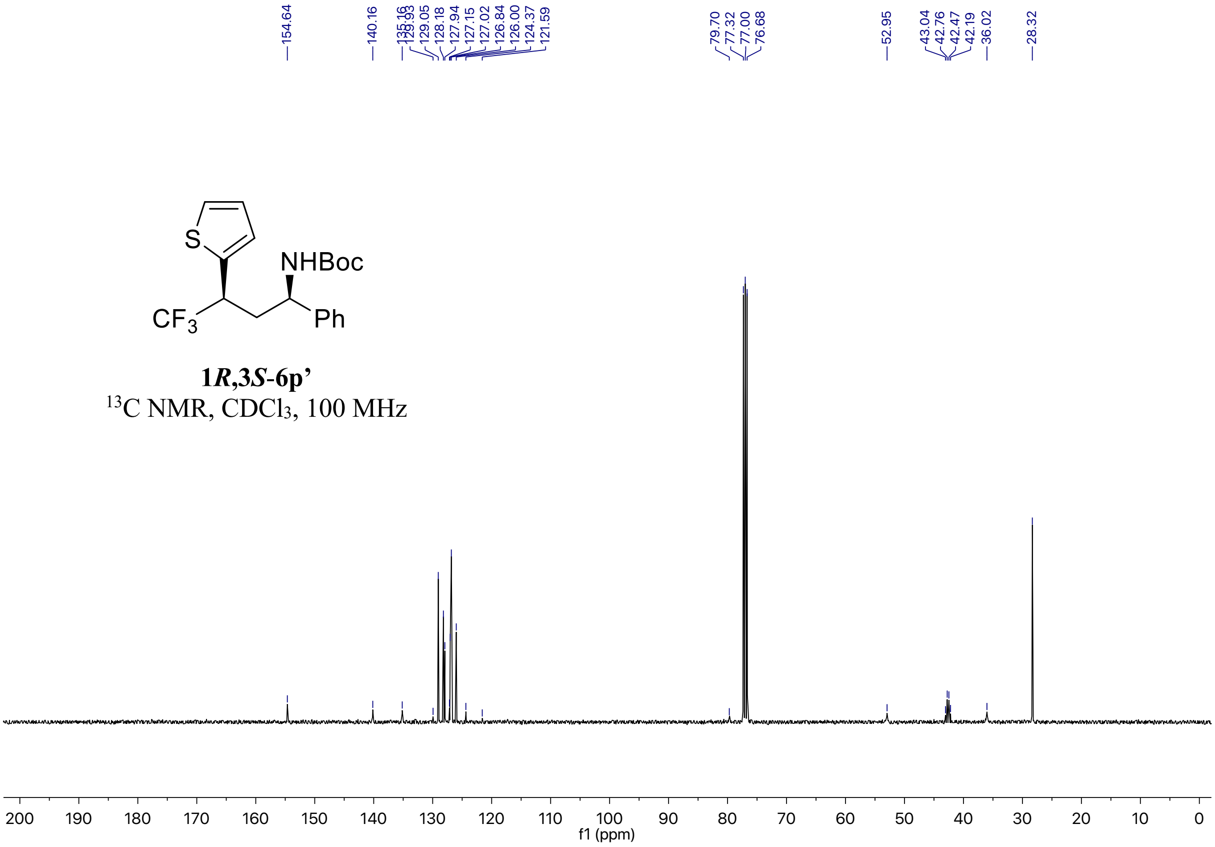

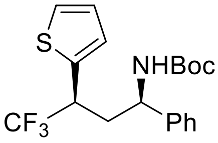

1*R*,3*S*-6p'

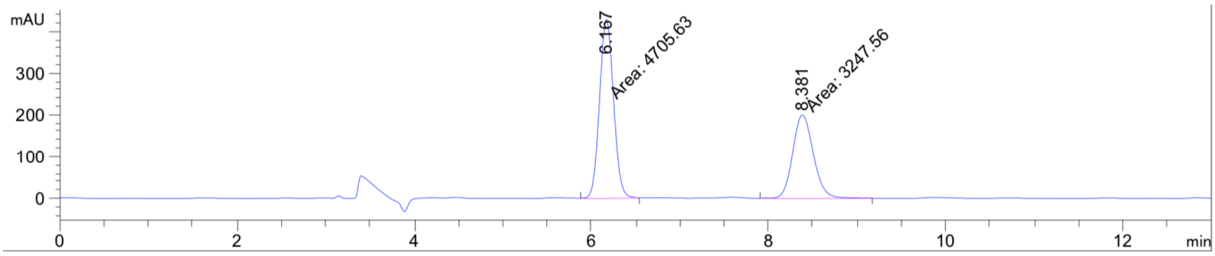

Peak #	RetTime [min]	Type	Width [min]	Area [mAU*s]	Height [mAU]	Area %
1	6.167	MM	0.1830	4705.63184	428.47260	59.1665
2	8.381	MM	0.2706	3247.56494	199.99536	40.8335

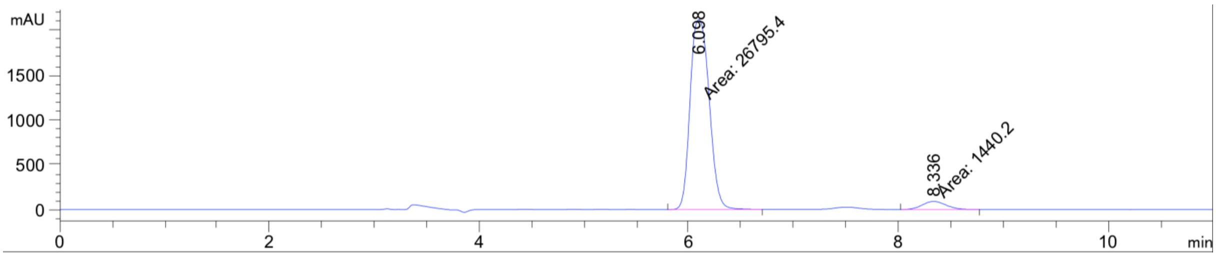

Peak #	RetTime [min]	Type	Width [min]	Area [mAU*s]	Height [mAU]	Area %
1	6.098	MM	0.2109	2.67954e4	2117.18213	94.8994
2	8.336	MM	0.2660	1440.19580	90.22694	5.1006

4,4,4-Trifluoro-1-phenylbutan-1-amine (6q)

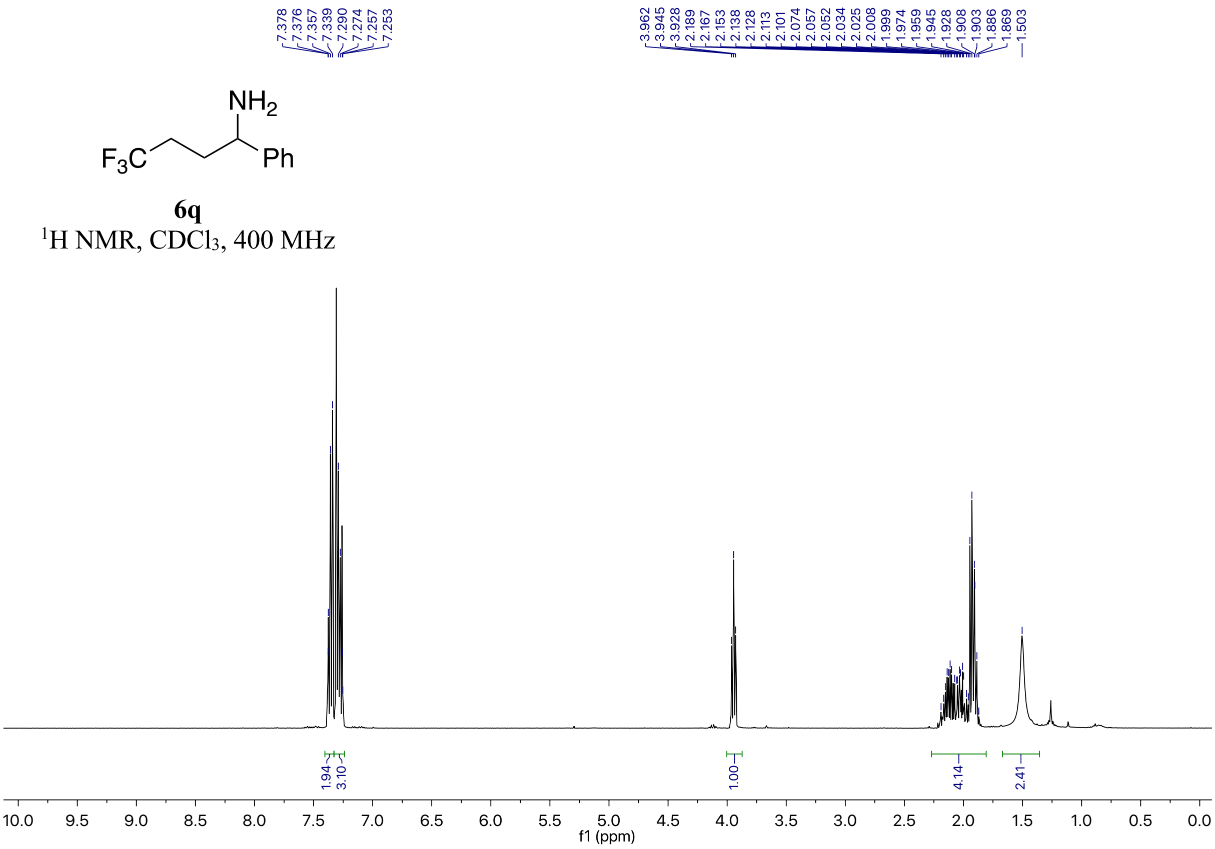

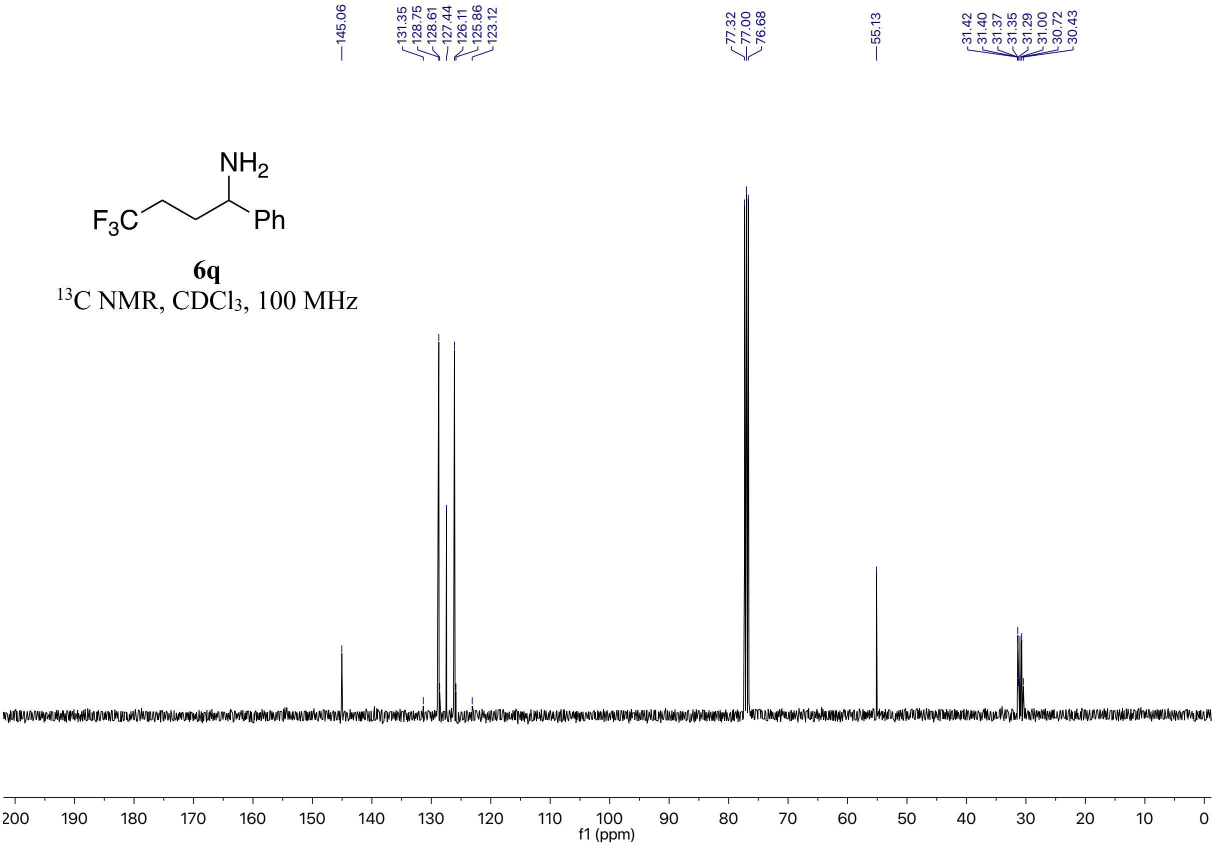

Crystal structure determination

Single crystal X-ray diffraction data on suitable crystals of compounds **4d’** and (**1*R*,3*R***)-**6d’** were collected using Cu Kα radiation on a Bruker D8 VENTURE diffractometer equipped with a PHOTON 100 detector. The dataset was reduced and absorption correction was applied using by the APEX3 suite. The crystal structures were solved and refined by SHELXT and SHELXL respectively.[1] The crystal structures were refined using full-matrix least-squares based on F^2 with all non-hydrogen atoms anisotropically defined. All hydrogen atoms were either located in the difference Fourier maps or placed using a riding model. A summary of the crystallographic data and refinement parameters are provided in Tables S2 and S3.
CCDC 2129456-2129457 contain the supplementary crystallographic data for this paper. These data can be obtained free of charge from The Cambridge Crystallographic Data Center via http://www.ccdc.cam.ac.uk/structures.
Compound **4d’** crystallizes in the Sohncke space group $P2_12_12_1$ with four molecules in the asymmetric unit and 16 molecules per unit cell. All molecules in the crystal had the same right-handed stereocenters (R configuration) and enantiopurity was confimed by the low Flack parameter. Hydrogen-bonding occurs between the amine and carbonate groups on neighbouring molecules forming a 1D hydrogen-bonded network along the *c*-axis.
Compound (**1*R*,3*R***)-**6d’** also crystallizes in the same Sohncke space group $P2_12_12_1$ but with only one molecule per asymmetric unit and four per unit cell. All molecules in the crystal had the same right-handed stereocenters (*R*,*R* configuration) and enantiopurity was confirmed by the low Flack parameter.

Both crystals **4d’** and **6d’** were grown by diffusion crystallization using a mixture pentane:CH_2Cl_2 (ca 97:3) as solvent system.

[1] G. Sheldrick, *Acta Cryst.*, **2008**, *A64*, 112-122

X-Ray - 4d’

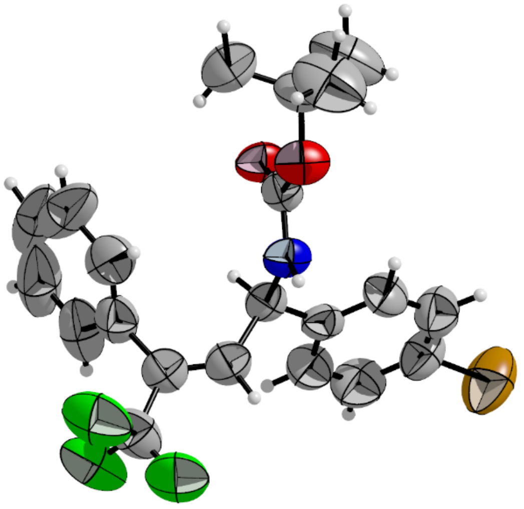

Figure S1. Structure of **4d’** (CCDC 2129456) as determined by single crystal X-ray diffraction. Thermal ellipsoids are displayed with 50% probability. Atom are colored as the following: carbon (grey), oxygen (red), nitrogen (blue), fluorine (green), bromine (brown), hydrogen (white spheres).

Table S2: Crystallographic data and refinement details for compound **4d'**

Empirical formula	C21 H21 Br F3 N O2
formula weight	456.30
temperature	296 K
wavelength	1.54178 Å
crystal system	orthorhombic
space group	$P2_12_12_1$ (No. 19)
unit cell dimensions	a = 13.2581 (3)Å
	b = 19.2734 (5) Å
	c = 34.5735 (9) Å
volume	8834.5 (4) $Å^3$
Z	16
density (calculated)	1.372 g/cm^3
absorption coefficient	2.893 mm^{-1}
F(000)	3712
θ range for data collection	2.556° to 70.344°
index ranges	$-16\leq h\leq 16$, $-22\leq k\leq 23$, $-33\leq l\leq 41$
reflections collected	16766
independent reflections	8392 [R(int) = 0.1144]
absorption correction	multi-scan
data / restraints / parameters	16766 / 0 / 1038
goodness-of-fit on F^2	1.014
final R indices [$I>2\sigma(I)$]	$R1$ = 0.0575, w$R2$ = 0.1270
largest diff. peak and hole	0.244 and –0.261 e/$Å^3$
Flack parameter	0.126 (13)

X-Ray - (1*R*,3*R*)-6d'

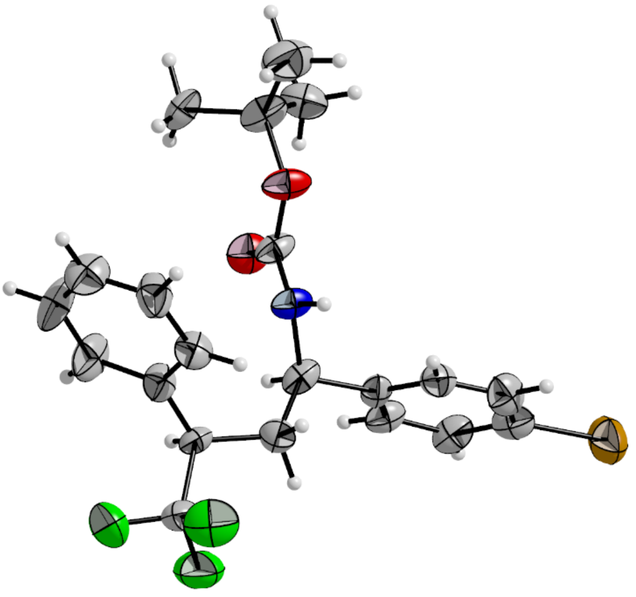

Figure S2. Structure of (**1*R*,3*R***)-**6d'** (CCDC 2129457) as determined by single crystal X-ray diffraction. Thermal ellipsoids are displayed with 50% probability. Atom are colored as the following: carbon (grey), oxygen (red), nitrogen (blue), fluorine (green), bromine (brown), hydrogen (white spheres).

Table S3: Crystallographic data and refinement details for compound (**1*R*,3*R***)-**6d'**

Empirical formula	C21 H22 Br F3 N O2
formula weight	457.30
temperature	296 K
wavelength	1.54178 Å
crystal system	orthorhombic
space group	$P2_12_12_1$ (No. 19)
unit cell dimensions	a = 5.4631 (5) Å
	b = 17.5350 (13) Å
	c = 22.3359 (19) Å
volume	2139.7 (3) Å^3
Z	4
density (calculated)	1.420 g/cm^3
absorption coefficient	2.987 mm^{-1}
F(000)	932
θ range for data collection	3.204° to 68.471°
index ranges	$-6 \leq h \leq 5$, $-20 \leq k \leq 21$, $-17 \leq l \leq 26$
reflections collected	3795
independent reflections	1813 [R(int) = 0.2085]
absorption correction	multi-scan
data / restraints / parameters	3795 / 0 / 261
goodness-of-fit on F^2	0.983
final R indices [I>2σ(I)]	R1 = 0.1083, wR2 = 0.2572
largest diff. peak and hole	0.961 and –0.912 e/Å^3
Flack parameter	0.09 (6)
